# Identification of DNA methyltransferases and demethylases in *Solanum melongena* L., and their transcription dynamics during fruit development and after salt and drought stresses

**DOI:** 10.1371/journal.pone.0223581

**Published:** 2019-10-09

**Authors:** Andrea Moglia, Silvia Gianoglio, Alberto Acquadro, Danila Valentino, Anna Maria Milani, Sergio Lanteri, Cinzia Comino

**Affiliations:** Department of Agricultural, Forest and Food Sciences, Plant Genetics and Breeding, University of Torino, Grugliasco, Italy; National Taiwan University, TAIWAN

## Abstract

DNA methylation through the activity of cytosine-5-methyltransferases (C5-MTases) and DNA demethylases plays important roles in genome protection as well as in regulating gene expression during plant development and plant response to environmental stresses. In this study, we report on a genome-wide identification of six C5-MTases (*Smel*MET1, *Smel*CMT2, *Smel*CMT3a, *Smel*CMT3b, *Smel*DRM2, *Smel*DRM3) and five demethylases (*Smel*Demethylase_1, *Smel*Demethylase_2, *Smel*Demethylase_3, *Smel*Demethylase_4, *Smel*Demethylase_5) in eggplant. Gene structural characteristics, chromosomal localization and phylogenetic analyses are also described. The transcript profiling of both C5-MTases and demethylases was assessed at three stages of fruit development in three eggplant commercial F_1_ hybrids: i.e. ‘Clara’, ‘Nite Lady’ and ‘Bella Roma’, representative of the eggplant berry phenotypic variation. The trend of activation of C5-MTases and demethylase genes varied in function of the stage of fruit development and was genotype dependent. The transcription pattern of C5MTAses and demethylases was also assessed in leaves of the F1 hybrid ‘Nite Lady’ subjected to salt and drought stresses. A marked up-regulation and down-regulation of some C5-MTases and demethylases was detected, while others did not vary in their expression profile. Our results suggest a role for both C5-MTases and demethylases during fruit development, as well as in response to abiotic stresses in eggplant, and provide a starting framework for supporting future epigenetic studies in the species.

## Introduction

DNA methylation of the fifth carbon of a cytosine residue is an epigenetic modification that strongly impacts chromatin structure and plays an essential role in gene regulation and imprinting, as well as in the defence against the invasion of mobile DNA elements, such as transposons, viruses and retroelements.

In plants, the cytosine methylation can occur in the three contexts: CG, CHG and CHH (where H stands for A, C, or T). CG and CHG are defined as ‘symmetrical’ contexts, since they are palindromic and during DNA replication both daughter strands are hemi-methylated and serve as template for specific classes of methyltransferases called ‘maintenance methyltransferases’. The maintenance of CG is operated by MET1, a homologue of mammalian Dnmt1 [DNA (cytosine-5-)-methyltransferase 1], whose role has been validated through mutant analyses [1] and methylome mapping [2,3]. CHG methylation context is maintained by CHROMOMETHYLASE 3 (CMT3), which mainly acts in centromeric and transposon regions [4,5] and to a much lesser extent by CHROMOMETHYLASE 2 (CMT2). CMT family is plant specific, and its members are characterized by two distinctive domains in their N-terminal region: CHROMO (chromatin organization modifier) and BAH (bromo-adjacent-homology). In *Arabidopsis thaliana*, there is evidence for a positive feedback between H3K9me2 histone modifications and CHG methylation as the chromo-domain of CMT3 recognizes this chromatin modification to reinforce silencing at these regions [6].CHH context, instead, is defined as ‘non-symmetrical’, since during DNA replication the asymmetric methylation lacks a methylated cytosine on the opposite strand, thus the methylation in this context needs to be established *de novo* after each cycle of DNA replication. Through the RNA directed DNA methylation (RdDM) pathway [7,8], DOMAINS REARRANGED METHYLTRANSFERASE2 (DRM2, an ortholog of mammalian Dnmt3) maintains CHH methylation at target regions (*i*.*e*: young and short transposons and other repeat sequences) in euchromatin, whereas CMT2 catalyses CHH methylation (with a self-reinforcing model with the H3K9me2 methylation [9]) at histone H1-containing heterochromatin, where RdDM is inhibited. Finally, *de novo* methylation in all contexts is also catalysed by DRM2 through the RdDM.

Recently, a fourth type of C5-MTase (*e*.*g*.: DNMT3,[10]), has been characterized in *Physcomitrella patens*, where two DNMT3s are present. In this basal plant, the DNMT3b mediates CG and CHH *de novo* methylation, independently of DRMs. The DNMT3 class has not been detected in any available angiosperm genomes or transcriptomes, supporting the hypothesis of its loss during evolution.

DNA demethylation may occur passively during DNA replication, due to a lack of DNA methyltransferase activity, or actively through the removal of 5-methylcytosines operated by DNA demethylases, which act as glycosilases/lyases through the base excision repair (BER) pathway.

In plants, the majority of methylated sequences occur in heterochromatin regions, enriched with transposable elements and repetitive sequences [11]. Methylation associated to genes can occur in the promoters as well as within the transcribed gene body (gene body methylation, gbM); while promoter DNA methylation usually imposes a repressive effect on gene expression, gbM genes are typically longer than unmethylated ones and are often constitutively active housekeeping [12,13]. Choi and colleagues [14], recently demonstrated that H1 and gbM are cooperatively involved in the repression of aberrant intragenic transcripts in Arabidopsis a gbM function already proposed when gbM was first discovered [15]

DNA methylation and demethylation are dynamic and strongly associated with plant development [8,16–18], by regulating key biological processes, such as leaf growth [19], seed development [20,21], heterosis of hybrids [22,23], fruit ripening [18,21,24] as well as synthesis of secondary metabolites [25]. Moreover, DNA methylation plays an essential role in response to biotic and abiotic stresses through modifications in the (de)methylation pattern at coding regions in some stress responsive genes [26–29].

The isolation and characterization of C5-MTase and demethylases have been carried out in several plant species, such as Arabidopsis [30], rice [31], tomato [24,32], soybean [33], maize [34], peanut [35], globe artichoke [36], carrot [37], peach [38], strawberry [39], oil palm [40], ricinus [41] and wheat [42], but not in eggplant.

Thanks to the recent availability of a high quality, annotated and anchored eggplant genome sequence [43] (www.eggplantgenome.org), we report on the identification and characterization of C5-MTases and demethylases in this species on the basis of sequence homology, functional domain identification and phylogenetic analyses. In addition, the expression dynamics of C5-MTases and demethylases were assessed at three stages of fruit development as well as in leaves of plants subjected to salt and drought stresses.

## Materials and methods

### Characterization of eggplant C5-MTase and demethylase sequences

Protein sequences of C5-MTases and demethylases of *Solanum lycopersicum* (collected from Sol Genomics Network, https://solgenomics.net) and *Arabidopsis thaliana* (collected form TAIR, www.arabidopsis.org), were used as query to search against the annotated proteome of eggplant (www.eggplantgenome.org) through a BLASTp search. Hits were filtered using the e-value cutoff of 1e^-5^ and the corresponding mRNA sequences were retrieved. Moreover, reverse translated C5-MTases and demethylases as well as the methyltransferase domains of MET1, CMT, and DRMs have been used as query to search in the eggplant genome using tblastn tool.

### Structure and chromosomal location of genes encoding C5-MTase and demethylase

The domain structure of the eggplant C5-MTases and demethylases was established by implementing hmmer software (hmmer.org/) along with the Pfam database (pfam.xfam.org/). Using the eggplant genome structural annotation (www.eggplantgenome.org), the graphical gene structure (exon/intron) was obtained by applying the script available at http://wormweb.org/exonintron. The genome localization of methyltransferase/demethylase sequences on chromosomes was obtained making use of the CIRCOS software (circos.ca). The presence and location of nuclear localization signals (NLS) were predicted via the cNLS Mapper software (http://nls-mapper.iab.keio.ac.jp/cgi-bin/NLS_Mapper_form.cgi).

### Phylogenetic analysis

The multiple sequence alignment of identified C5-MTases in *Solanum melongena*, *Arabidopsis thaliana*, *Cynara cardunculus*, *Glycine max*, *Oryza sativa*, *Solanum lycopersicum*, *Zea mays*, *Fragaria x ananassa*, *Solanum tuberosum*, *Salvia miltiorrhiza*, *Sorghum bicolor*, *Brachypodium distachyon*, *Ricinus communis* and *Populus trichocarpa* was obtained through Clustal Omega online software (www.ebi.ac.uk/tools/msa/clustalo/). Multiple sequence alignment was also performed for demethylases isolated from *Solanum melongena*, *Solanum lycopersicum*, *Arabidopsis thaliana*, *Cynara cardunculus*, *Ricinus communis*, *Brassica rapa*, *Capsella rubella*, *Cucumis sativus*, *Cucumis melo*, *Fragaria vesca Malus domestica*, *Zea mays and Oryza sativa*. A phylogenetic tree was generated by the Neighbor joining (NJ) method in conjunction with the *p*-distance, and pairwise deletion of gaps for the computation of evolutionary distances. In order to obtain a support value for each branch, bootstrap value was performed with 1,000 replicates. Sequences used for the construction of the C5-MTase and demethylase phylogenetic trees are listed in S1 and S2 Files, respectively.

### Protein modelling

RaptorX (http://raptorx.uchicago.edu/) was used to predict secondary and tertiary protein structures based on aminoacidic sequences; these structures were then visualized using the Chimera software (https://www.cgl.ucsf.edu/chimera/). Protein domains as identified by Prosite, hmmer and blastp were highlighted on each structure. Each eggplant protein was compared to its tomato or Arabidopsis orthologs when a tomato homolog was not available (as is the case of *Smel*DRM3 and *Smel*Demethylase_5). The Chimera MatchMaker tool was used to superimpose the structures of related eggplant and tomato or *A*. *thaliana* proteins to reveal the extent of structural conservation/divergence. Global alignments were obtained using the Needleman-Wunsch algorithm with default settings; alignments restricted to a single catalytic or regulatory domain, where structures couldn't be superposed globally, were obtained using the Smith-Waterman algorithm with default settings. Alignments were refined by iterated pruning.

### Plant material

#### Fruit development analysis

Plantlets of three commercial hybrids representative of the eggplant berry phenotypic variation: i.e. ‘Clara’ producing white and ovoid fruits, ‘Nite Lady’ producing black and elongated fruits and ‘Bella Roma’ producing pale violet and round fruits, were grown, in a greenhouse, in pots containing topsoil and until they developed six true leaves. Afterwards, they were transplanted in field at the agricultural experimental farm of DISAFA in Carmagnola (Turin, Italy), during the summer of 2017. Standard cultivation practices were applied. Fruits were harvested at ~5 (stage 1, corresponding to small fruits enclosed in the calyx), ~14 (stage 2, immature fruit) and ~25 (stage 3, ripe fruits) days after anthesis (S1 Fig). Collected samples were immediately frozen in liquid nitrogen and stored at −80°C. RNA was extracted from three biological replicates for each fruit developmental stage.

#### Salt stress analysis

Six plants of the hybrid ‘Nite Lady’ were also grown in a climate room at 25°C, with 60% relative humidity and a long-day photoperiod (16 h light/8 h dark cycle) at 300 μmol m^-2^s^-1^ light intensity. At the stage of six fully developed leaves, they were transferred into plastic pots containing a mixture of peat and sand. Three plants were irrigated with water (control plants), while three with a water solution supplemented with 150 mM NaCl. Three weeks after treatment, leaf samples were immediately frozen in liquid nitrogen and stored at −80°C. RNA was extracted from leaves collected from each of the treated and control plants.

#### Drought stress analysis

Six plants of the hybrid ‘Nite Lady’ were grown in Jiffy-7 growing media in a climate room at 25°C, with 60% relative humidity and a long-day photoperiod (16 h light/8 h dark cycle) at 300 μmol m^-2^s^-1^ light intensity. Drought treatment was applied at the same plant growth stage (3 week-old plants). Three plants were irrigated with water (control plants), while for three plants water was withheld for 2 days. Two days after treatment, leaf samples were immediately frozen in liquid nitrogen and stored at −80°C. RNA was extracted from leaves collected from each of the treated and control plants.

### RNA extraction and quantitative Real-time PCR analysis

Frozen tissues were ground in liquid nitrogen to a fine powder, from which RNA was extracted using the Spectrum Plant Total RNA kit (Sigma Aldrich). The single strand cDNA was synthesized from 1 μg of RNA using a High Capacity RNA-to-cDNA kit (Applied Biosystems, Foster City, USA) as directed by the manufacturer. Primers (S3 File) were designed on the basis of CDS sequence using the Primer3 software (http://bioinfo.ut.ee/primer3). PCR reactions were carried out using the StepOnePlus Real-Time PCR System (Applied Biosystems). The following PCR protocol was applied 95°C/10min, followed by 40 cycles of 95°C/15s and 60°C/1min. Data were quantified using the 2^- ΔΔCt^ method based on Ct values of C5MTases/demethylases and actin (as housekeeping gene)[44].

## Results

### Gene identification, structure and chromosomal localization

Based on a Blastp survey of the eggplant proteome set and on a tBlastn search on the eggplant genome, six *loci* (SMEL_000g005920.1.01, SMEL_008g299520.1.01, SMEL_001g152860.1.01, SMEL_005g241610.1.01, SMEL_002g159210.1.01 and SMEL_005g227660.1.01; Table 1) were identified as C5-MTases, and five (SMEL_009g320250.1.01, SMEL_010g357340.1.01, SMEL_011g366300.1.01, SMEL_003g197280.1 and SMEL_011g367960.1.01; Table 1) as demethylases. One locus (SMEL_008g310230.1.01) was identified as SmelDnmt2.

**Table 1:** Eggplant *loci* encoding C5-MTases and demethylases, their location and annotation of their various protein domains.

*Locus*	Gene name	Chr	Chromosome location	ORF length (bp)	Exon num	Prot size (aa)	kDa	pI	Domains	Pfam Dom.
*C5-MTases*										
**SMEL_000g005920.1.01**	*Smel*MET1	0	1183618..1195028	11411	12	1552	174,56	5,89	DNMT1-RFD (2)–BAH DCM I–BAH Plant DCM II–DNA methylase	Pfam 12047 (2)– 01426 (2)– 00145
**SMEL_008g299520.1.01**	*Smel*CMT2	8	2762317..2771058	8742	21	1062	119,25	8,02	SWIRM-assoc_2 –BAH–CHROMO—DNA methylase	Pfam 16496–01426–00385–00145
**SMEL_001g152860.1.01**	*Smel*CMT3a	1	135745005..135754602	9598	20	873	98,71	4,8	BAH–CHROMO–DNA methylase	Pfam 01426–00385–00145
**SMEL_005g241610.1.01**	*Smel*CMT3b	5	43316293..43325201	8909	21	934	104,1	5,39	BAH–CHROMO–DNA methylase	Pfam 01426–00385–00145
**SMEL_002g159210.1.01**	*Smel*DRM2	2	57486097..57520113	34017	9	602	67,79	4,82	UBA (2)–SAM-dependent MTase DRM-type	Pfam 00627 (2) - 00145
**SMEL_005g227660.1.01**	*Smel*DRM3	5	4965885..4983559	17675	11	699	78,64	5,37	UBA (2)–SAM-dependent MTase DRM-type	Pfam 00627 (2) - 00145
***DNMT2***										
**SMEL_008g310230.1.01**	SmelDnmt2	8	90090255..90097200	6946	7	334	37,55	5,22	DNA methylase	Pfam 00145
*Demethylases*										
**SMEL_009g320250.1.01**	*Sme*lDemethylase_1	9	919477..929609	10133	19	1848	207,28	6,3	HhH-GPD—Perm-CXXC—RRM-DME	Pfam 00730–15629–15628
**SMEL_010g357340.1.01**	*Smel*Demethylase_2	10	102148465..102159304	10840	20	1840	203,69	6,3	HhH-GPD—Perm-CXXC—RRM-DME	Pfam 00730–15629–15628
**SMEL_011g366300.1.01**	*Smel*Demethylase_3	11	8127481..8138610	11130	21	1876	209,45	8,24	HhH-GPD—Perm-CXXC—RRM-DME	Pfam 00730–15629–15628
**SMEL_003g197280.1**	*Smel*Demethylase_4	3	94446469..94458094	11626	21	1482	167,31	8,74	HhH-GPD—RRM-DME	Pfam 00730–15628
**SMEL_011g367960.1.01**	*Smel*Demethylase_5	11	13028970..13032877	3908	15	566	63,78	8,35	HhH-GPD—RRM-DME	Pfam 00730–15628

**Fig 1:**
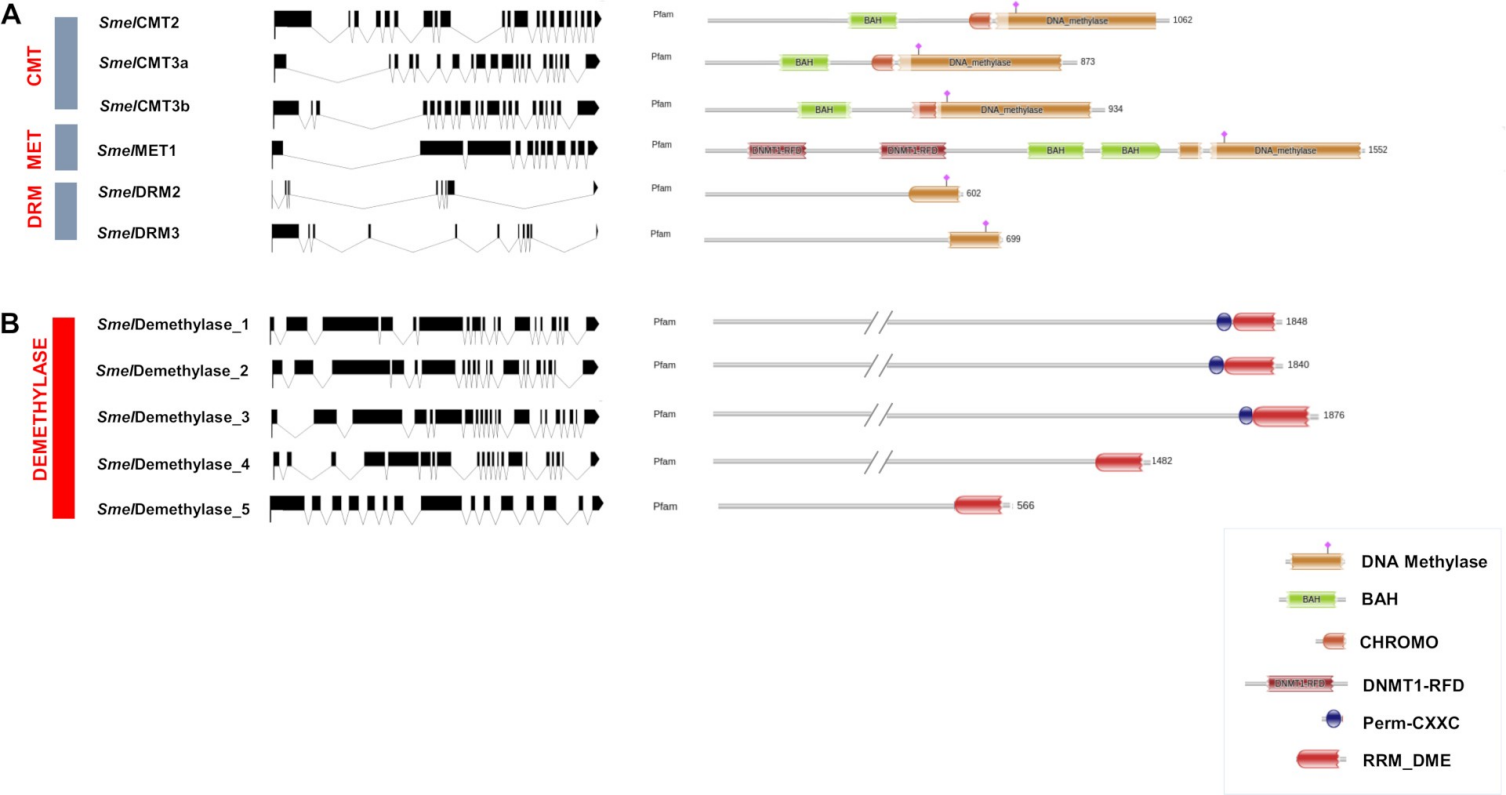
**Gene and protein structure of the eggplant set of (A) C5-MTases, (B) demethylases.** On the left hand panel the exon/intron structures are showed, with exons displayed as black boxes and introns as lines (generated using the tool provided at http://wormweb.org/exonintron). Name of the motifs/domains are shown inside the figure. Acronym explanations: BAH = Bromo Adjacent Homology domain; CHROMO = CHRromatin Organisation MOdifier domain; DNMT1-RFD = Cytosine specific DNA methyltransferase Replication Foci Domain; Perm-CXXC = Permuted single zf-CXXC unit; RRM_DME = RNA-recognition motif in Demeter.

**Fig 2:**
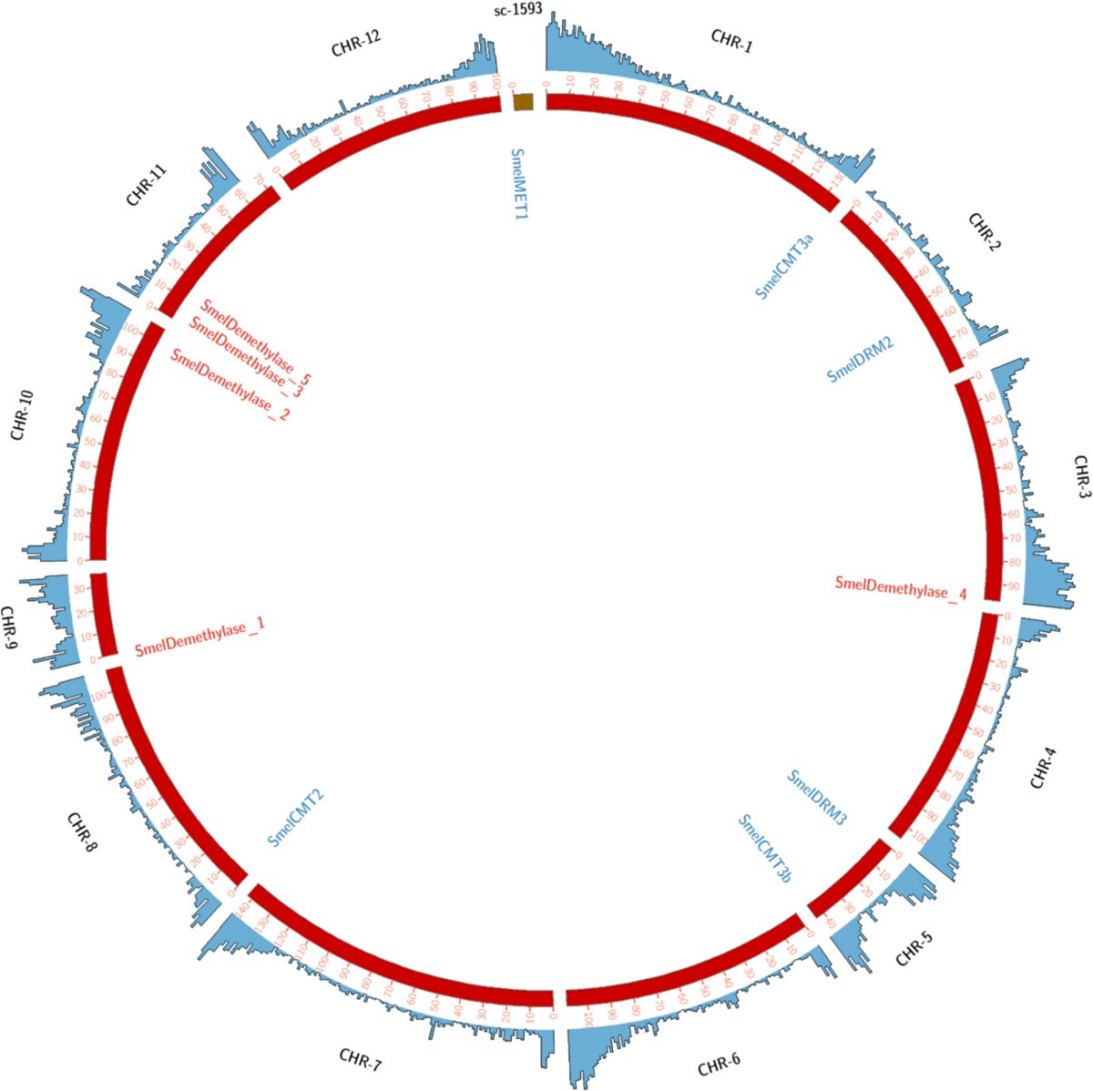
Chromosomal location of genes encoding C5-MTase and demethylase. The 12 pseudomolecules (chromosomes) are depicted by the set of red bars in the center, and gene density (1 Mbp windows) by the outer blue track. Location of each gene is showed.

The six C5-MTases encode proteins ranging in size from 602 to 1552 amino acids (aa) and the five demethylases encode proteins ranging from 566 to 1876 aa (Table 1). The gene model of 11 *loci* were derived (Fig 1) and were located on eggplant chromosomes (Fig 2). The number of exons ranged from 9 to 21 in the C5-MTase group and from 15 to 21 among demethylases.

All the eggplant C5-MTases are characterized by the DNA methylation domain PF00145 at their C-terminus. Based on the presence of specific domains, ubiquitin-associated (UBA) domain, BAH (bromo adjacent homology domain), chromodomain and replication foci domain (RFD), the C5-MTases were attributed to different sub-classes. The SMEL_000g005920.1.01 *locus* was assigned to Methyltransferase1 sub-class and named *Smel*MET1, because of its sequence similarity and the presence of two BAH and two DNMT1-RFD domains. Three *loci* (SMEL_008g299520.1.01, SMEL_001g152860.1.01 and SMEL_005g241610.1.01) belong to the chromomethylase sub-class, since they harbour both CHROMO and BAH domains, and were named *Smel*CMT2, *Smel*CMT3a and *Smel*CMT3b, respectively. Two UBA domains (PF00627) are present in SMEL_002g159210.1.01 and SMEL_005g227660.1.01 which are named *Smel*DRM2 and *Smel*DRM3, respectively.

All the 5 demethylases harbour the RRM DME (RNA recognition motif demethylase—PF15628) and HhH-GPD (helix-hairpin-helix Gly/Pro, PF00730) domains. However, only *Smel*Demethylase_1, *Smel*Demethylase_2 and *Smel*Demethylase_3 harbour the Perm-CXXC (permuted single zf-CXXC, PF15629) domain.

The presence of nuclear localization signals (NLSs) in C5-MTases and demethylases is reported in Table 2. All the protein members showed the simultaneous occurrence of mono and bipartite NLS, with the exception of *Smel*CMTs and *Smel*Demethylase_5 characterized only by bipartite NLS.

**Table 2:** The mono- and bipartite nuclear localization signals (NLSs) (cut-off score = 5) identification in eggplant C5-MTases and demethylases. Score higher than 8 indicates an exclusive protein localization in the nucleus.

	Monopartite NLSs	Starting monopartite NLS	Score monopartite NLS	Bipartite NLSs	Starting position of bipartite NLS	Score bipartite NLS
***Smel*MET1**	3	50-649-1088	9-8-5	9	29-35-54-626-630-951-957-1086-1304	5–6,5–5,7–5,7–5,3–5,7–5,1–5,9–6,2
***Smel*CMT2**	-	-	-	5	53-126-133-178-195	6,4–10,2–7,2-6-5,3
***Smel*CMT3a**	-	-	-	2	2–417	9,5–5,3
***Smel*CMT3b**	-	-	-	4	2-53-187-510	8,3–7,1–5,1–5,1
***Smel*DRM2**	1	224	10	1	324	5,3
***Smel*DRM3**	1	308	5,5	1	293	10,1
***Smel*Demethylase_1**	2	281–535	8–10,5	4	181-196-488-797	5,2–5,5–5,6–6,0
***Smel*Demethylase_2**	1	388	5.0	3	262-277-860	5-4-5,4–6,1
***Smel*Demethylase_3**	4	351-386-423-1274	6-5-5-8	5	348-351-354-365-369	5,1–6,6–6,6–6,3–10,7
***Smel*Demethylase_4**	3	143-158-611	10–8,5–7,0	3	144-157-480	11,3–5,7–5,8
***Smel*Demethylase_5**	-	-	-	2	531–537	5,6–5,5

### Phylogenetic analysis

**Fig 3:**
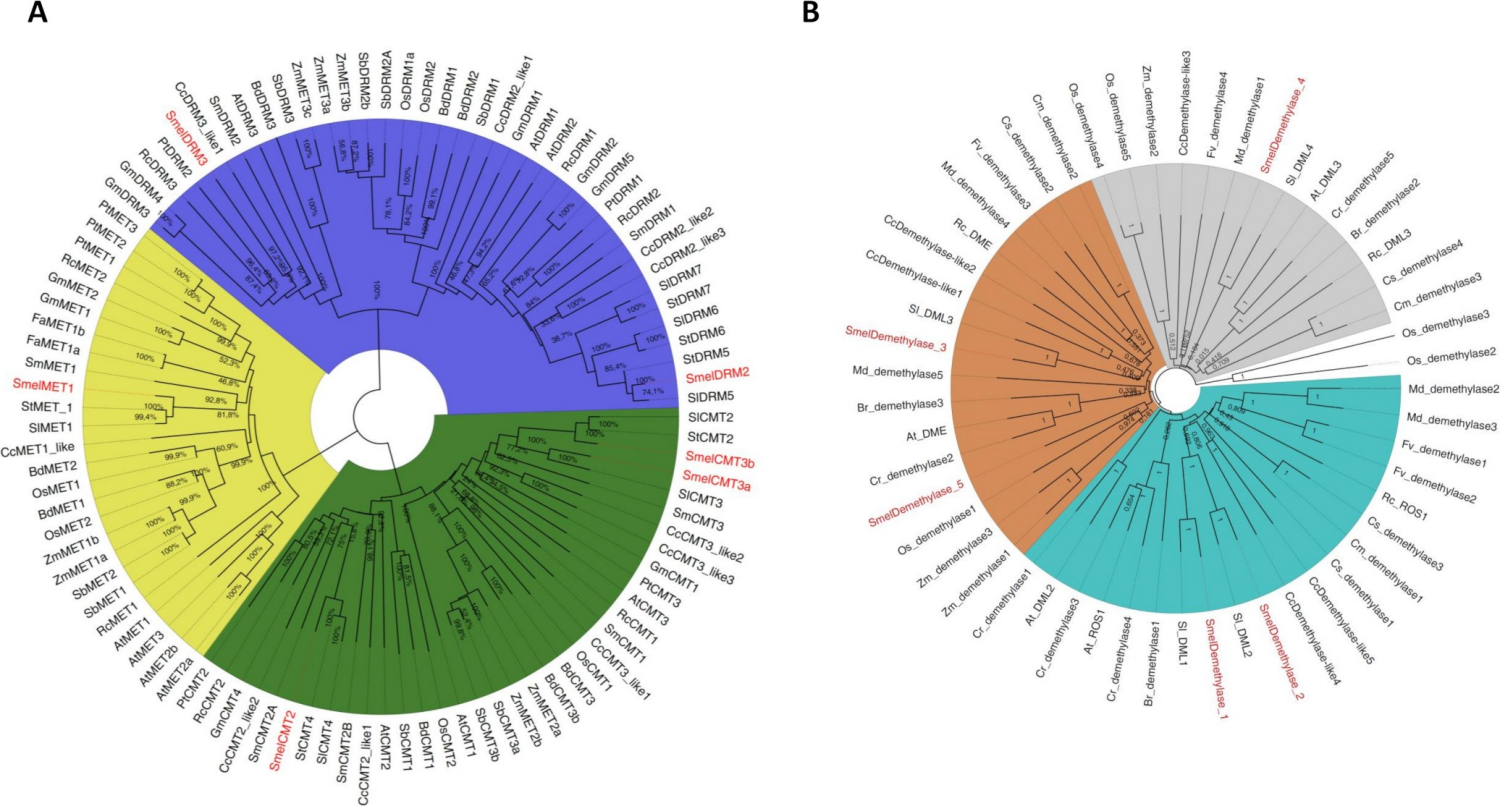
**Phylogenetic analysis of the genes encoding (A) C5-MTase and (B) DNA demethylases.** The two unrooted, neighbour-joining trees were constructed by aligning the C5-MTase and DNA demethylases protein sequences contained in S1 and S2 Files, respectively. Colours (yellow, green and violet) in the tree A indicate the main three clades obtained for the C5-MTases corresponding to the three subclasses: MET1, CMT and DRM, respectively. Colours (pale blue, grey and orange) in the tree B indicate the main three clades obtained for the DNA demethylase corresponding to the three subclasses present in Arabidopsis: ROS, DML and DME, respectively. The number at each node represents the bootstrap percentage value from 1,000 replicates. *Smel = Solanum melongena*, *At = Arabidopsis thaliana*, *Cc = Cynara cardunculus*, *Gm = Glycine max*, *Os = Oryza sativa*, *Sl = Solanum lycopersicum*, *Zm = Zea mays*, *Fa = Fragaria x ananassa*, *St = Solanum tuberosum*, *Sm = Salvia miltiorrhiza*, *Sb = Sorghum bicolor*, *Bd = Brachypodium distachyon*, *Rc = Ricinus communis*, *Pt = Populus trichocarpa*, *Br = Brassica rapa*, *Cr = Capsella rubella*, *Cs = Cucumis sativus*, *Cm = Cucumis melo*, *Fv = Fragaria vesca*, *Md = Malus domestica*.

Full-length protein sequences of eggplant C5-MTase, along with others of 13 species (S1 File), were used for phylogenetic tree construction. The resulting unrooted, neighbour-joining tree is presented in Fig 3A. The C5-MTase grouped in three clades (highlighted in pale yellow, green and violet colours) and corresponding to the three plant DNA C5-MTase families: MET, CMT and DRM, respectively, with support values close to 100 in accordance with classification based on domain composition. Within each clade, members of the *Solanum* taxon (tomato, potato and eggplant) were clearly separated from other plant species. The phylogenetic tree highlighted a close evolutionary relationship between MET and CMT members containing the BAH domain. The MET protein from *A*. *thaliana* formed a separate sub-clade with respect to the ones of monocotyledonous and dicotyledonous species (including *Smel*MET1). The CMT clade was divided in two main sub-clades, one including mainly CMT2 proteins, while the other groups the CMT1/CMT3 proteins (containing *Smel*CMT3a and *Smel*CMT3b). The DRM clade included two main sub-clades, one containing *Smel*DRM2, and the other *Smel*DRM3. In all the C5-MTase clades, dicots and monocots formed distinct groups, supporting the hypothesis that an independent evolution occurred for these genes. An analysis based solely on the methyltransferase domain (MTD) was also performed and the resulting phylogenetic tree (S2 Fig) confirmed what obtained on the basis of full protein sequences (Fig 3A).

The same phylogenetic analysis was performed for the eggplant demethylases together with the ones from *S*. *lycopersicum*, *A*. *thaliana*, *C*. *cardunculus*, *R*. *communis*, *B*. *rapa*, *C*. *rubella*, *C*. *sativus*, *C*. *melo*, *F*. *vesca*, *M*. *domestica*, *Z*.*mays and O*. *sativa* (Fig 3B; S2 File). Results indicated that all 53 proteins were mainly clustered into three groups (*i*.*e*.: ROS, DML and DME), consistent with what obtained in Arabidopsis. Specifically, *Smel*Demethylase_1/ Demethylase_2 resulted closer to each other than to *Smel*Demethylase*_*5 and *Smel*Demethylase_3, while *Smel*Demethylase_4 showed to be the most phylogenetically distant. Within each clade, eggplant proteins showed a strong phylogenetic relatedness with tomato homologs, with the exception of *Smel*Demethylase_5, for which no tomato ortholog was highlighted.

### Protein modelling

By comparing proteins both within and between species, in MET and CMT families, a high degree of conservation was observed in the methylases, BAH and CHROMO domains (S3 Fig). In the DRM family, a high degree of conservation was observed between *Smel*DRM2 and *Sl*DRM5. Since *Smel*DRM3 does not own a close tomato ortholog, the Arabidopsis *At*DRM3 protein was used for comparison; here structural homology appears mostly restricted to the methylase and UBA domain located in the N-terminal portion of the protein.

Structural conservation in the eggplant demethylases appeared to be restricted to the three RRM DME, Perm-CXXC and HhH GPD domains (S4 Fig), characteristic of base excision DNA repair proteins. *Smel*Demethylase_1, *Smel*Demethylase_2, *Smel*Demethylase_3 and *Smel*Demethylase_4 displayed an overall good degree of structural conservation with respect to their tomato homologs (*Sl*DML1-4, respectively). *Smel*Demethylase_5 was compared to its Arabidopsis ortholog AtDME.

### Transcriptional profiling during fruit development

**Fig 4:**
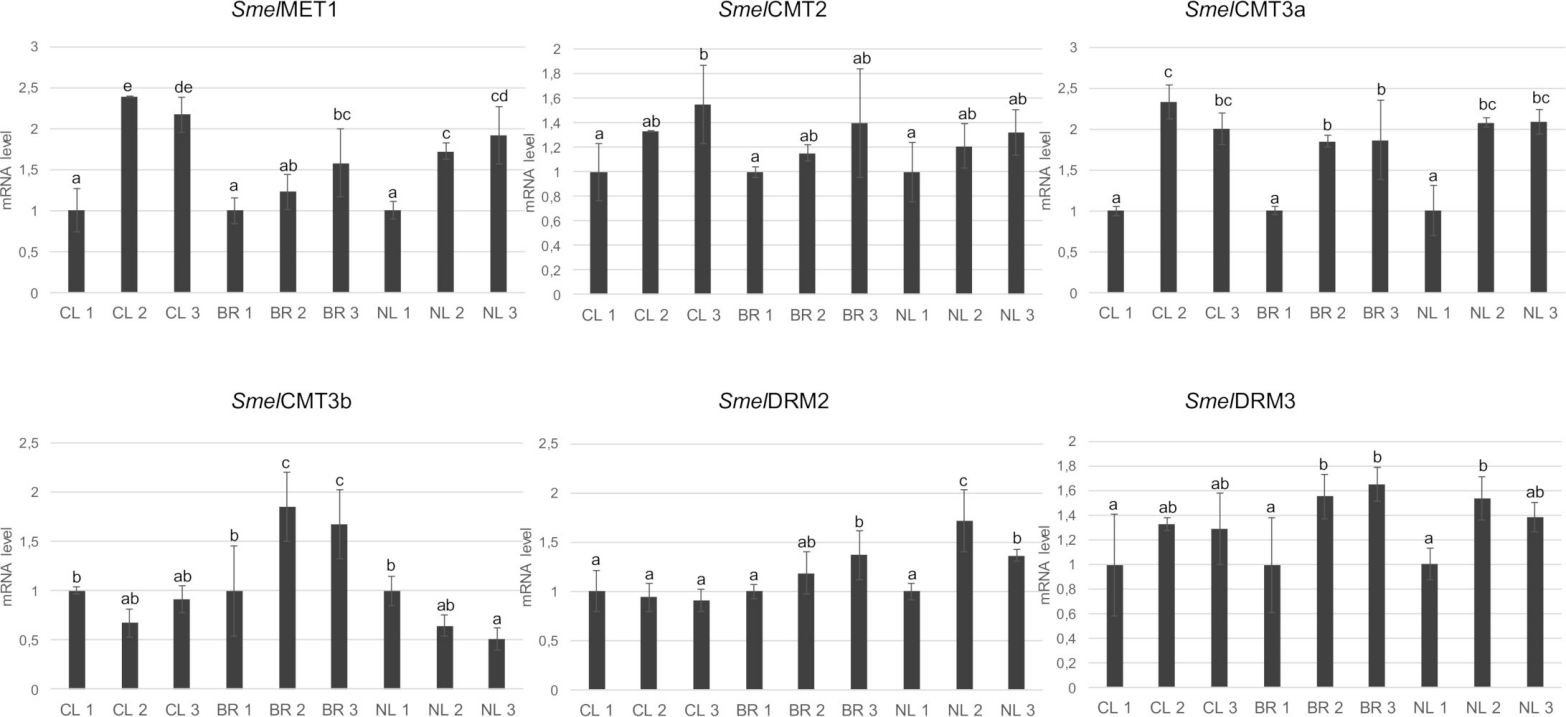
qRT-PCR based transcription profiling of eggplant C5-MTase during fruit development in the three eggplant F1 hybrids. On “X” axis, the three eggplant F1 hybrids (CL = Clara, BR = Bella Roma and NL = Nite Lady) are represented at three fruit stages (5, 14 and 25 days after anthesis) indicated with the numbers as 1, 2 and 3, respectively. The actin eggplant gene was used as the reference sequence. Error bars represent SD (*n* = 3). Different letters associated with the set of means indicate a significant difference based on Tukey's HSD test (P≤0.05).

**Fig 5:**
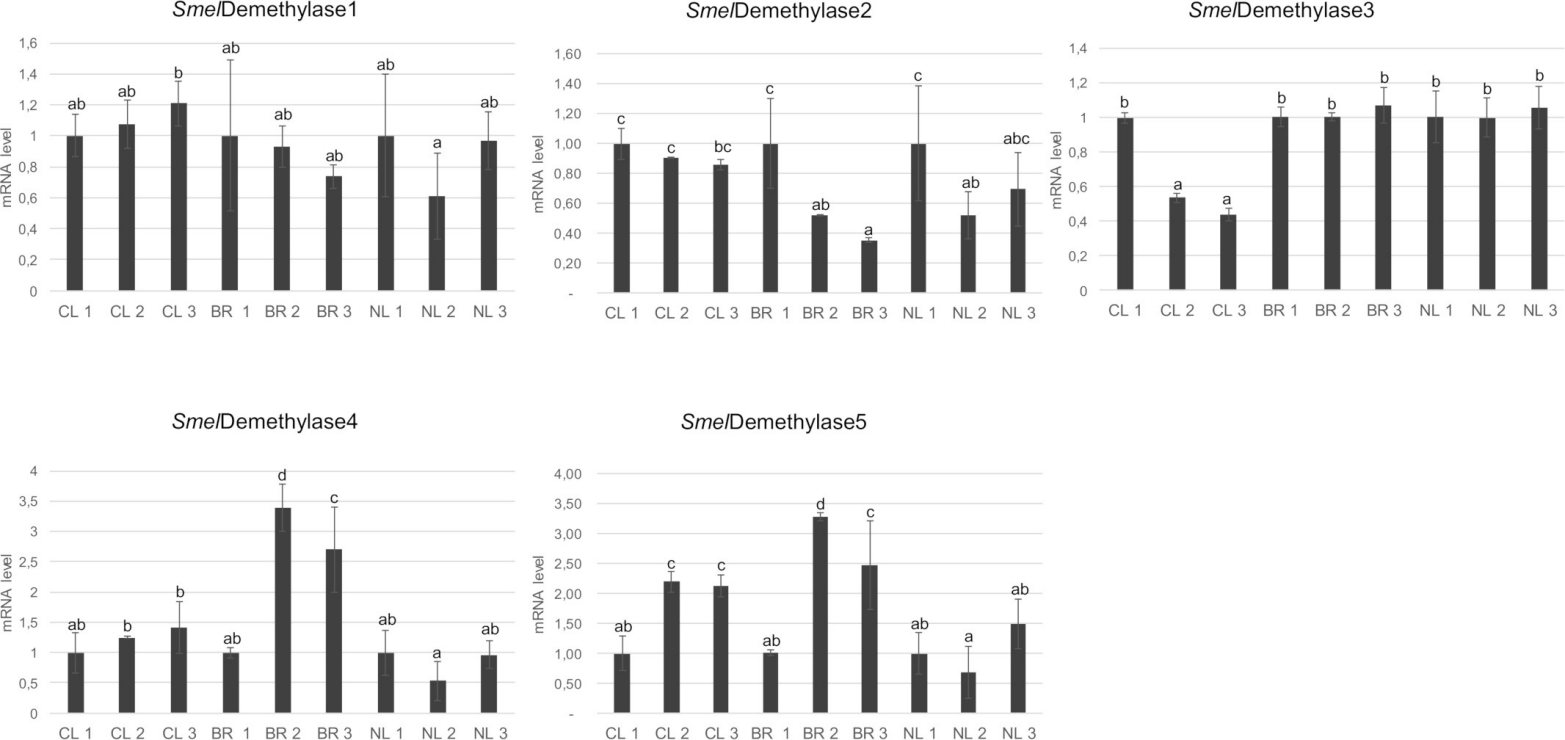
qRT-PCR based transcription profiling of eggplant DNA demethylase during fruit development in the three eggplant F1 hybrids. On “X” axis, the three eggplant F1 hybrids (CL = Clara, BR = Bella Roma and NL = Nite Lady) are represented at three fruit stages (5, 14 and 25 days after anthesis) indicated with the numbers as 1, 2 and 3, respectively. The actin eggplant gene was used as the reference sequence. Error bars represent SD (*n* = 3). Different letters associated with the set of means indicate a significant difference based on Tukey's HSD test (P≤0.05).

Transcript abundances were estimated for the C5-MTase (Fig 4) and demethylase (Fig 5) genes in eggplant berries sampled at three stages of fruit development. *Smel*MET1 transcription was strongest in the 2^nd^ and 3^rd^ development stages in all hybrid plants under study. *Smel*CMT2 showed an analogous trend in the three cultivars, and its transcription slightly but not significantly increased during fruit development. *Smel*CMT3 transcript level increased during development, with transcript level ~2 fold higher in the 3^rd^ development stage. Significant changes in the transcript level of *Smel*CMT3b were highlighted only in 'Bella Roma' with transcript levels respectively around 1.9 and 1.7 fold higher in the 2^nd^ and 3^rd^ stages. The transcription of *Smel*DRM2 didn’t show any conserved trends between developmental stages or genotypes. The transcript level of *Smel*DRM3 increased with the progress of fruit development in ‘Clara’ and ‘Bella Roma’, with transcript levels respectively around 1.3 and 1.7 fold higher.

The transcription of *Smel*Demethylase_1 was at a similar level across the various developmental stages, while *Smel*Demethylase_2 decreased from 1^st^ to 3^rd^ stage in all three hybrids (with a reduction going from 15 to 65%). *Smel*Demethylase_3 transcript level showed a conserved trend in 'Bella Roma' and 'Nite Lady' hybrids and a reduction during fruit development in 'Clara'. Significant changes in *Smel*Demethylase_4 transcription were highlighted only in the 'Bella Roma' hybrid with transcript levels respectively around 3.4 and 2.7 fold higher in the 2^nd^ and 3^rd^ development stages. The transcript level of *Smel*Demethylase_5 increased with the progress of fruit development in ‘Clara’ and ‘Bella Roma’, with transcript levels respectively around 2.1 and 2.5 fold higher in the 3^rd^ development stage.

### Transcriptional profiling in response to salt and drought stresses

**Fig 6:**
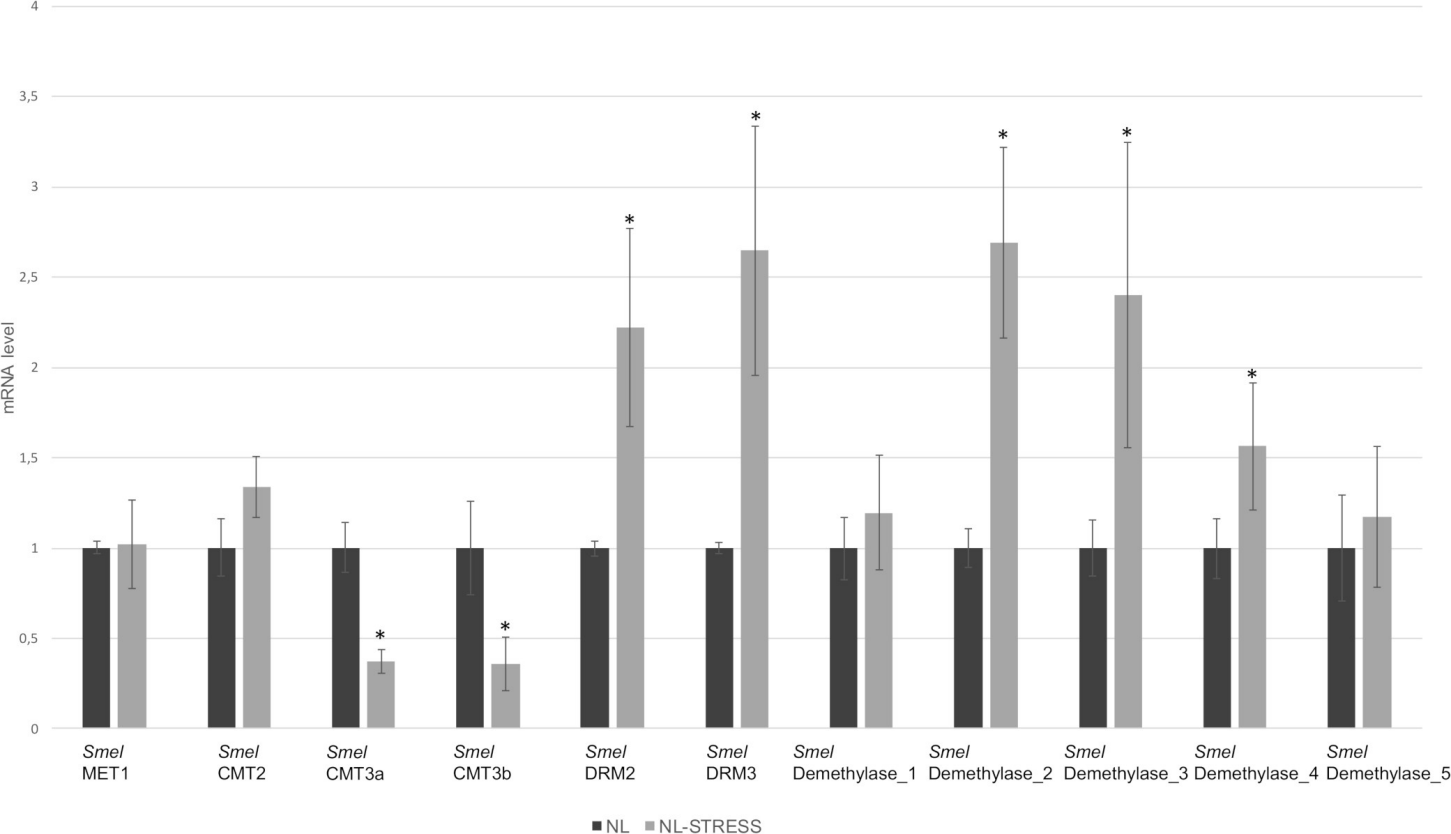
The patterns of expression of C5-MTase and DNA demethylase in leaf tissues of the F1 hybrid ‘Nite Lady’ in response to salt stress. The actin eggplant gene was used as the reference sequence. Error bars represent SD (*n* = 3). Asterisk indicates a significant difference based on Tukey's HSD (P≤0.05).

The transcription pattern of C5-MTases and demethylases under salt stress (150 mM NaCl for three weeks) was assessed in the F_1_ hybrid ‘Nite Lady’ (Fig 6). The transcription of *Smel*MET1 remained unchanged under salt stress. A slight but not significant increase in transcript level in response to salt stress was highlighted for *Smel*CMT2 (1.33 X). *Smel*CMT3a and *Smel*CMT3b were down-regulated, with transcript levels respectively 2.7 and 2.8 fold lower than control plants. *Smel*DRM2 and *Smel*DRM3 were strongly up-regulated in stressed samples, where transcript levels were respectively 2.2 and 2.6 fold higher. Among demethylases, no significant changes in transcription level were highlighted for *Smel*Demethylase_1 and *Smel*Demethylase_5. On the other hand, *Smel*Demethylase_2, *Smel*Demethylase_3 and *Smel*Demethylase_4 were strongly activated in salt-stressed plants, reaching an abundance 2.7, 2.5 and 1.6 fold higher than in controls.

**Fig 7:**
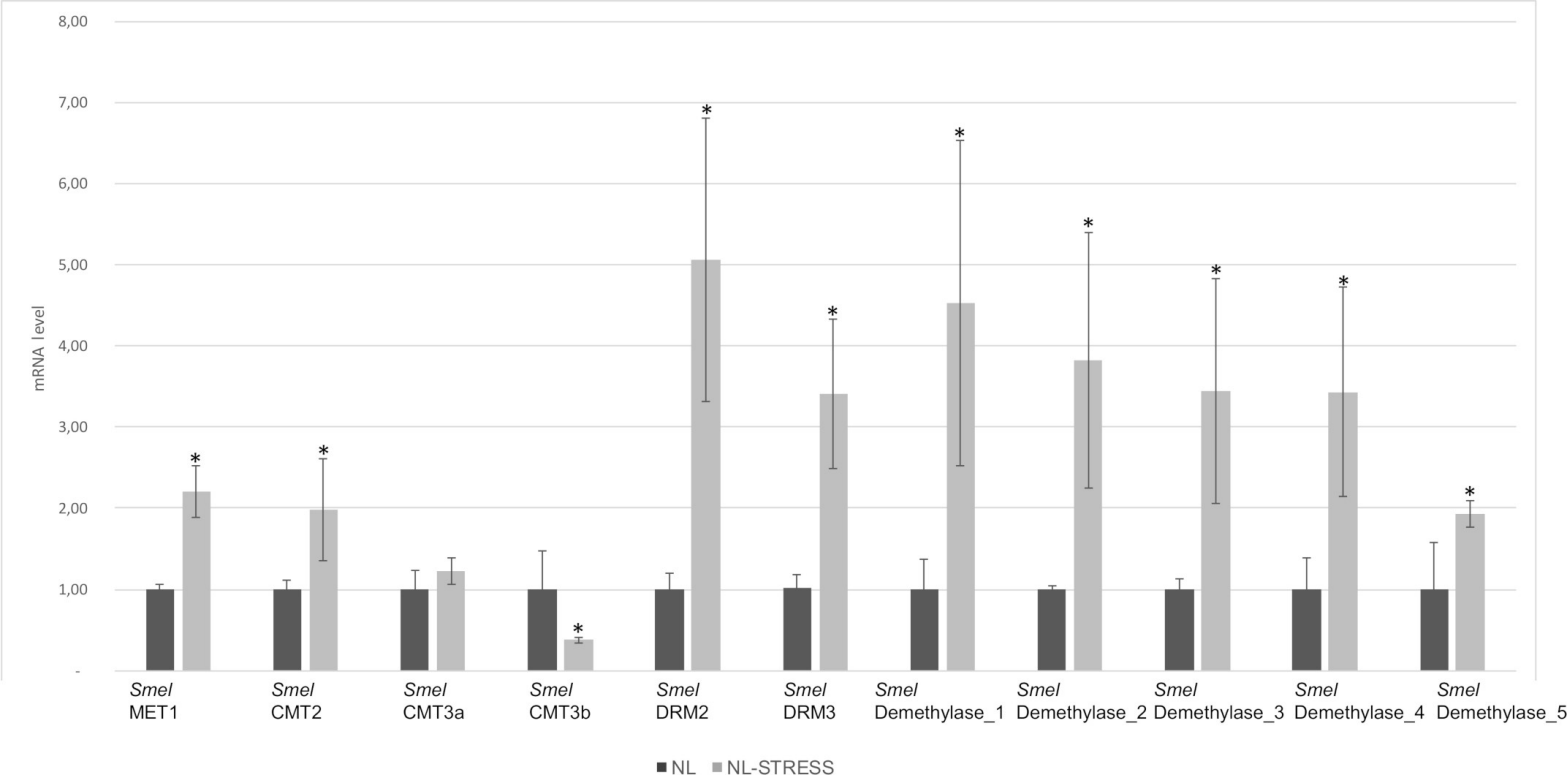
The patterns of expression of C5-MTase and DNA demethylase in leaf tissues of the F1 hybrid ‘Nite Lady’ in response to drought stress. The actin eggplant gene was used as the reference sequence. Error bars represent SD (*n* = 3). Asterisk indicates a significant difference based on Tukey's HSD (P≤0.05).

The transcription pattern of C5-MTase and demethylases under drought stress was also assessed in the F_1_ hybrid ‘Nite Lady’ (Fig 7). *Smel*MET1 and *Smel*CMT2 were up-regulated in treated samples, where transcript levels were respectively 2.2 and 2 fold higher. The transcription of *Smel*CMT3a remained unchanged under drought stress. *Smel*CMT3b was down-regulated in response to drought, with transcript levels respectively 2.7 fold lower than unstressed plants. *Smel*DRM2 and *Smel*DRM3 were strongly activated in drought-stressed plants, reaching an abundance 5.1 and 3.4 fold higher than control sample. Among demethylases, *Smel*Demethylase_1, *Smel*Demethylase_2, *Smel*Demethylase_3, *Smel*Demethylase_4 and *Smel*Demethylase_5 were strongly activated in drought-stressed plants, reaching an abundance 4.5, 3.8, 3.4, 3.4 and 1.9 fold higher than control sample.

## Discussion

The first eggplant leaf methylome map displayed 90% methylation in CG, 83% in CHG and 16% in CHH contexts [45]; these values are higher than those reported in tomato (75% CG, 50% CHG and 10% CHH) [18]. DNA methylation levels can be positively correlated with the density of transposable element, and negatively with gene density [46,47]. Indeed, in spite of the very similar number of genes, the eggplant genome is about 1.3-fold larger than the one of tomato, mainly due to the amplification of Gypsy and Copia elements [43] (www.eggplantgenome.org).

The pattern of DNA methylation is regulated by maintenance and *de novo* methylation as well as by demethylation, operated by C5-MTases and DNA demethylases, respectively. Thanks to the recent availability of a high quality eggplant genome sequence [43], we got access and characterized the full repertoire of genes encoding these two classes of enzymes. We identified six C5-MTases grouped into the three known subfamilies, MET, CMT, DRM, on the basis of their domain organization and phylogenetic relationship (Fig 3A). The range in size of the predicted C5-MTase is consistent with the one reported in *A*. *thaliana* and tomato [32] (Table 1). The number of isolated C5-MTases corresponds to that identified in potato, but it is lower than the one reported in pepper (11) and tomato (8) [48]. Eggplant displays one MET1 gene, like tomato and potato, and 3 CMTs, like potato, and only 2 DRMs. However, the identification of an analogous number of C5-MTase genes in eggplant, potato and tomato may suggest a similar function within the three phylogenetically closer Solanaceae species. No CMT1-like homologs were revealed in the eggplant genome, as previously observed in other Solanaceae species [48]. This gene, when present, was found to be almost silent in *A*. *thaliana*, and it is defective in many ecotypes of the species [30].

The phylogenetic tree highlights a strong relatedness of eggplant C5-MTases with homologs from other Solanaceae [47] (Fig 3A). The domain pattern shows conservation across C5-MTase families in eggplant and mirrors the one of other plant species, strengthening the hypothesis of a common evolutionary origin and a conserved function (Fig 1 and S3 Fig). Differently from MET1 of other Solanaceae [48], *Smel*MET1 is characterized by the presence of two RFD domains instead of one, and by two BAH domain. RFD domains functions non-catalytically to target the protein towards replication foci allowing MET protein to methylate the correct residues. As previously clarified, one BAH domain (BAH1) is similar to the BAH domains of CMT, and might be involved in MET interaction with histone tails, while the other (BAH2) participates in molecular interaction with other proteins [33].

Tomato contains four putative DNA demethylases (called *Sl*DML1 to 4) [24], two of which (*Sl*DML1 and *Sl*DML2) are closely related to the Arabidopsis ROS1 (*At*ROS1)[49]. In our study, we identified 5 demethylases harbouring three recognizable domains: Perm-CXXC, RRM DME and HhH GPD (Fig 1, S4 Fig). Based on the phylogenetic analysis, homologs of tomato DML1, DML2, DML3 and DML4 could be clearly spotted in the eggplant genome (Fig 3B).

DNA methylation profiles play a key role in the regulation of fruit developmental processes, such as fruit ripening and size change [16–18,24,49–51]. It has been reported that both tomato and strawberry undergo a loss of DNA methylation during ripening [18,51,52], while in sweet orange an increase of methylation was observed [53]. The variations of DNA methylation levels are closely related to the expression of cytosine DNA methyltransferases/demethylases, which act dynamically during plant development.

Recently it has been also observed that duplicated transcription factors involved in tomato fruit ripening can be regulated through DNA methylation mechanisms [54]. In strawberry, genes involved in RNA-directed DNA methylation are downregulated during ripening, contributing to DNA hypometylation [52]. DNA methylation variations in the promoter of the *Malus domestica* MYB10 gene, a key transcription factor regulating anthocyanin biosynthesis in apple, are likely epigenetic factors causing the fruit colour variation [55].

Our results highlight that the abundance of *Smel*MET1, *Smel*CMT2, *Smel*CMT3a and *Smel*DRM3 rises during eggplant fruit development, while that of *Smel*CMT3b and *Smel*DRM2 remains unchanged (Fig 4). These data are in accordance with the one previously reported in tomato, in which a differential expression of MET1, DRM6, CMT2 (homolog of *Smel*CMT3b) and CMT4 (homolog of *Smel*CMT2) during fruit ripening was observed [32,51]. Analogies are also detectable with the trend of expression of DNA methyltransferase and demethylase genes in strawberry, in which the expression peaks at either pre-turning or turning stage were detected [39]. The current model of tomato ripening suggests that active demethylation, operated by the Demeter-like demethylase *Sl*DML2, is needed to trigger fruit ripening [24,49]; in loss of function mutants of *Sl*DML2 fruits no ripening occurs [49]. Demethylation induces a gradual decrease in promoter methylation of fruit ripening-induced genes, such as COLOURESS NON-RIPENING (CNR), whose epi-mutation has been demonstrated to inhibit ripening. Surprisingly, as compared to *Sl*DML2, we observed a similar trend for *Smel*Demethylase_5, while an opposite one for *Smel*Demethylase_2, showing a sharp decrease of transcription during fruit development (Fig 5).

DNA methylation plays essential roles in regulating gene expression of plants exposed to biotic and abiotic stresses [5] by hindering/suppressing transcription. In particular, it has been observed that the level of DNA methylation is dynamically regulated and often enhanced in plants exposed to salt stress conditions [56–58]. In *A*. *thaliana* the changes in DNA methylation induced by high salinity were found to be transmitted to the next generation, while if the progeny is not stressed the epigenetic status can be reset [59]. In the present study, the transcription changes of C5-MTases and demethylases under drought and soil salinity stresses, the major abiotic stresses affecting eggplant productivity and quality, were evaluated. The up-regulation of *Smel*DRM2 and *Smel*DRM3 following NaCl treatment suggests that these genes may be involved in salt stress response (Fig 6). This also seems confirmed by the fact that up-regulation of the DRM family in response to salt stress has been generally reported in tomato [48], rice [31] and soybean [33]. Among demethylases, we observed an up-regulation in response to salt stress of *Smel*Demethylase_2, *Smel*Demethylase_3 and *Smel*Demethylase_4, while no response was noticed for *Smel*Demethylase_1 and *Smel*Demethylase_5.

In a previous study in *Fragaria vesca* and *Pyrus betulaefolia* [39,60], the expression of demethylase genes was found to be altered in response to salinity, supporting its potential role in the plant response to this abiotic stress.

The up-regulation of the DRMs and Demethylases was also observed after drought stress induced on plants of the F1 ‘Nite Lady’. These data are in accordance with the ones previously reported in tomato and soybean [33,48].

Our results would confirm that the expression of DRMs and Demethylases is regulated by multiple abiotic stress conditions in eggplant, suggesting a common regulatory mechanism(s) that may exert influence on sensing and signalling cascade governing both drought and salt stress.

In conclusion, we report on the identification of six C5-MTases and five DNA demethylases in eggplant, whose genomic structure and genomic localization have also been achieved. Differential transcript abundance of C5-MTase and DNA demethylase genes highlights their involvement in regulating fruit ripening and salt and drought stress response, providing a starting framework for supporting future epigenetic studies in the species. Thanks to the ongoing development of the CRISPR/Cas9 system in eggplant, our future goal will be to perform the functional characterization of key isolated C5-MTases and DNA demethylases.

## Supporting information

S1 File Methyltransferase sequences used for tree construction. *Solanum melongena (Smel)*, *Arabidopsis thaliana (At)*, *Cynara cardunculus (Cc)*, *Glycine max (Gm)*, *Oryza sativa (Os)*, *Solanum lycopersicum (Sl)*, *Zea mays (Zm)*, *Fragaria x ananassa (Fa)*, *Solanum tuberosum (St)*, *Salvia miltiorrhiza (Sm)*, *Sorghum bicolor (Sb)*, *Brachypodium distachyon (Bd)*, *Ricinus communis (Rc)* and *Populus trichocarpa (Pt)*.
(DOCX)

S2 File Demethylase sequences used for tree construction. *Solanum melongena (Smel)*, *Solanum lycopersicum (Sl)*, *Arabidopsis thaliana (At)*, *Cynara cardunculus (Cc)*, *Ricinus communis (Rc)*, *Brassica rapa (Br)*, *Capsella rubella (Cr)*, *Cucumis sativus (Cs)*, *Cucumis melo (Cm)*, *Fragaria vesca (Fv)*, *Malus domestica (Md)*, *Zea mays (Zm) and Oryza sativa (Os)*.
(DOCX)

S3 File Primer sequences used in the qRT-PCR assays. (DOCX)

S1 Fig Fruits of Clara (a), Bella Roma (B) and Nite Lady (c) ~25 (stage 3, ripe fruits) days after anthesis.
(TIFF)

S2 Fig Phylogenetic analysis of the C5-MTases. The unrooted, neighbour-joining tree was constructed by aligning the methylase domain of C5-MTase protein sequences contained in S1 File. The number at each node represents the bootstrap percentage value from 1,000 replicates.
(TIFF)

S3 Fig Protein models of eggplant C5-MTases. The three-dimensional structures of eggplant C5-MTases have been compared to those of *S*. *lycopersicum*, when possible, and of *A*. *thaliana* when a tomato ortholog was not available. The methylase domain is highlighted in green, the BAH domain in blue, the CHROMO domain in red and the UBA domain in magenta.
(TIFF)

S4 Fig Protein models of eggplant DNA demethylases. The three-dimensional structures of eggplant demethylases have been compared to those of *S*. *lycopersicum*, when possible, and of *A*. *thaliana* when a tomato ortholog was not available. The HhH-GPD domain is highlighted in orange, the Perm-CXXC domain in purple, and the RRM DME domain in green.
(TIFF)
