# Supplementary material for: Identification of DNA methyltransferases and demethylases in Solanum melongena L., and their transcription dynamics during fruit development and after salt and drought stresses
Source: PLoS One. 2019 Oct 9;14(10):e0223581. doi: 10.1371/journal.pone.0223581 (PMC6785084; doi:10.1371/journal.pone.0223581)
Supplement: S1 File — Solanum melongena (Smel), Arabidopsis thaliana (At), Cynara cardunculus (Cc), Glycine max (Gm), Oryza sativa (Os), Solanum lycopersicum (Sl), Zea mays (Zm), Fragaria x ananassa (Fa), Solanum tuberosum (St), Salvia miltiorrhiza (Sm), Sorghum bicolor (Sb), Brachypodium distachyon (Bd), Ricinus communis (Rc) and Populus trichocarpa (Pt). (DOCX) [file pone.0223581.s001.docx]

>*Smel*MET1

MGSLPALDKPDTDAGYKKNKTKQDSQSKRKTSATDKKEKKQPVSESIEELTAARKRPKRAAACSDFKEKSLPLSKTSSVIETRKDHCVEEEDVAIRLTAGLQESQRPCRRLTDFVFHNSEGIPQPFEMSEVDDLFISGLILPLEDSLDKEKAKGIRCEGFGRIEEWAISGYEDGTPVIWISTETADYDCVKPSGSYKKFYDHFLAKAMACVEVYKKLSKSSGGNPNLSLDELLAGVVRAMNGIKCFSGGVSIRDFVVTQGRFIYNQLIGLDETSKKTDQLFVELPVLASLRDESSKLETLAQPEPISFGKALHIGPKAVNGEDKIDESGLANCAASEDEDLKLAKLLHEEEYWRSLKQKKGRNTSSSSSKIYIKINEDEIASDYPLPAYYKTSNEETDEYIVFDSGVDTYHIDELPRSMLHNWALYNSDSRLISLELLPMKACADIDVTIFGSGVMTADDGSGYNFDTDANHSSSCGSRPAEIDGMPIYLSAIKEWMIEFGSSMIFISIRTDMAWYRLGKPSKQYAPWYEPVIKTARLAVSIITLLKEQSRVARLSFGDVIKRISEFKKDRPAYISSNVDVVERYVVVHGQIILQQFSEFPDVSIRNCAFAIGLSRKMEERHHTKWLIKKKKVMQRHEQNLNPRASMAPSVKRKAMQATTTRLINRIWGEYYSNYSPEVSKEVVDCEVNDDEEADEQEENEEDDVPEMNLDVPEKTHTLSSARKHIKSHSDSKEINWDGESTGKTASGELLFERARVHGHEIAVGDSVLVEHDEPDELPSIYFVEYMFEKLDGSKMLHGRMMQRGSDTVLGNAANEREVFLINECMDLQLGDIKESIAVNIRIMPWGHQHRNTNADKLDRAKAEDRKRKGLPTEFYCKSFYRPERGAFFRLPFDKMGLGNGLCYSCELQRTDQEKESFKFDMSNSSFVYLGTEYSVDDFVYVSPDHFTEERGGTGTFKAGRNVGLMAYVVCQLLEIVGPKGSKQAKVDSTNAKVRRFFRPEDISSDKAYSSDIREIYYSEEIHTVSVGTIKGKCEVRKKYDISSEDVPAIFDHNFFCEYLYDPSNGSLKKLPAQIKLKFSKVKLDDATSRKRKGKGKEGEDEAGGLNEASPQNRLATLDIFAGCGGLSEGLQHSGVTDTKWAIEYEEPAADAFRLNHPKTKVFIHNCNVILRAVMQKCGDSDDCISTPEASELAAAMDENELNSLPLPGQVDFINGGPPCQGFSGMNRFNQSTWSKVQCEMILAFLSFADYYRPKFFLLENVRNFVSFNQKQTFRLSVASLLEMGYQVRFGILEAGAFGVPQSRKRAFIWAASPEEVLPEWPEPMHVFSVPELKITLSETSHYAAVRSTASGAPFRSLTVRDTIGDLPVVGNGACKTCIEYQGDPVSWFQKKIRGSSITLSDHISKEMNELNLIRCQRIPKRPGADWRDLQDEKVKLSNGQLVDLIPWCLPNTAKRHNQWKGLFGRLDWDGNFPTSITDPQPMGKVGMCFHPEQDRIVTVRECARSQGFPDSYQFAGNILQKHRQIGNAVPPPLAYALGRKLKEAVESKKLT

>*Smel*CMT3a

MSTKRKASPAESPESKRHAVTENADQVVEEPTEDFLVECFVDEETVFDGLNDGSHAEKKERRVVQVNEEQEGVFSGETVRDSEAREKWPHRYIIKDKVKANGTPMSLNCQDGSNQLIQAKCHYTQAMVDSQIYKLGDDAYVKAADGEDDYICKIVEFFQAVDDMKYFTAQWFYRAKDTVIKAHDQFIDNKRVFLSEIKDDNPLDCLVSLQFKESLRSNSDYYYDMKYLVPFSSFVSLSSDFRDSAVFADVSSPYSESDSTISTDGDVVEANEQKQEKKLLDLYSGCGGMSTGLCLGADVCGVKLVTKWTVDLNRYACDSLKVNHPETEVRNESASDYLLLLKEWEQLCASCSLLKSNTPAHPLLKVGDEDEEDDDDGGDEDEGSGDNEEGEIFEVEEILEVCYGDPNEIKKPGLYFKVRWKGYGPDEDTWEPIEGLEYEFQTKQHVVNPCFSFLTHLSNELIFAYSGCQKKIKDFVIKGFQASLFPLPGEVDVICGGPPCQGISGFNRFRNSANPLQDPKNKQLEVFMGIVEFLKPRFVLMENVVDLLRFANGYLGRYALSRLVGMNYQARMGMMVAGAYGLPQFRMRVFMWGALPSEKLPQYPLPTHNVIVRGGIPTEFELNAVEYEEGLQVKLKRELLLEDALSDLPPVENNEPRDEMPYTGEPKSDFQCFIRSKRDGTLGTVLYDHRPLQLNDDDYQRVCQIPKRKGGNFRDLPGVRVRADNIVEWDPDVERVKLTSGKPLVPDYAMTFVRGTSQKPFGRLWWDEIVSTVVTRAEPHNQAILHPVQDRVLTIRENARLQGFPDYYKLTGPIKERYIQVGNAVAVPVARALGYSLALALKGLSGDQPLLTLPPNFPRLEELVSNEEALDKL

>*Smel*CMT2

METELNNSSKISENQTPSPEKTTPISSSSPNPDALALSFSLNEEPVPLVVCYPGRRRSSRFSTNKFSTSELMKKNRKCISPEKEVLLPSALAGKKTPAESTRRSPRLVSPSATGKVKGKGKNVNSRKQVESSRKRKYKNNDDEPENPKTDGKKRRSDSVSAKLELLALPEADGAWGTRCGTKRELLALLTPEGSKNSKRGSRGSDSGNVGHDSRGSRRKNLVLAASPGTSEKKSDVVNSVGEKSLRSRRIQGSVDNEESDTKRKMNSSDSAEESGRKQKSNVFFIGEPIDAEEAQEKWQWRYELKSQNTQRKGWKLNSGEEDEIILNVECHYAQAKVAGFVFNIGDCAFVKGEGKKKHIGRILEFFKTTEGEDYFRVQWYFRAEDTVLKGAASFHDPKRIFYSTLENDNLLDCIVSKVNVIELPTAHALNKKDVPPAHFYYDMEYCVDYSTFRTLHNVKSSISPSLVDASYKPITTYPLEVSPSCEPMKVELSLLDLYAGCGGMSTGLCLGTKLSGVNLVTKWAVDFNKSACDSLKLNHPQTHVRNEGVENFLELLKRWEKLIELYGCSDIKTSSNDAVDDRDEGEINGDSLAGSDASSGEYEVLRLVDICYGDPNNDEKSGLHFKVRWKGYGPSEDTWEPIDNLENCGESIKDFVRRGQQLKILPLPGDVDMICGGPPCQGISGYNRHRNVDDPLSDEKNRQIIVFMDVVEFLRPKYVLMENVADILRFDKASLGRYALSRLVHMKYQARLGTMAAGCYGLPQFRLRVFFWGALPSERLPPFPLPSHDVIVKYWPSPEFERNTVAYEEGQPRDLEEALVLRDAISDLPAVTWHETREEMPYEMPPETAFQKYIRLSKHEIMSCSSTGVKEAKEPVLFDHRPCQLNEDDYFRVCLVPHRKGANFRDLPGVIVGGDNVARRDTKDPKVLPNGKPMVPDCAFNFEQGKSKRPFGRLWWDETVATLVTFPNHRAQAILHPEQDRVLTVREYARLQGFPDFYRFTGTPKERYCQVGNAVAVPVGRALGYALGLAYQRLAGNEPLIKLPPNFSFLTVPMDDIVVLQK

>*Smel*CMT3b

MPSKRKASPATKPESSSGSRKSKRLAVEKPDPVVAQPSDSDFESAPPVLSAKKKSTRGTTAESPVVASQSVSNDKKLNKRVVEKAESGVASPADTDFVSEPNLVTPGKRSGRGAAVKAEPVVDSAAESDFVDEEDVDETVQGSLKKSLSISPSKRKPKRAEKVKDEECTLPGDPVPDAEARLKWPHRYNKGKANGTKNTNGQDDSDQLIQAKCHFGRAKVDGQVYYLEDDAHVKAADGDDDYICKIVEFFEAVDGTQYFRAQWFYRAKDTVIKSHDQFIDNKRVFLSEIKDDNPIDCLVTKLKIVPVPSNATLQFKENVKSNCDFYYDMKYLLPYSSFISLPPDTTSPVSSSSTISSDVDAGEVKEHNPEKKLLDLYSGCGAMSTGLCLGANSNGVKLVTKWAVDLNKHACDSLRLNHPETQVRNEYASDFLSLLKEWVQLCVSCSLIKGSVPPHPHLKVTDEVEEDEENDDEGEDSGDDKDGENFEVEELLEVCYGDPKEINKSGLYFKVHWKGYGPEEDTWEPIDGLSDCPKKIKEFVVKGFKANLLPLPGDVDVVCGGPPCQGISGFNRFRNKENPMQDPKNKQLDVYMDIVDFLKPRFVLMENVVDLVKFSNGFLGRYALSRLVGMNYQARMGMMAAGAYGLPQFRMRVFMFGALSSEKLPQYPLPTHKVIVRGVIPTEFESNTVAYDEGRDFELKKELFLGDALSDLPSVENNEPRDEMPYADEPKSDFQHFIRLGRDGVLGSVLYDHRPLQLNDDDYQRVCQIPKRKGANFRDLPGVRVRPDKKVEWDPDVERVKLPSGKPLVPDYAMSFVGGSSSKPFGRLWWDETVPTVVTRAEPHNQTIIHPLQDRVLTIRENARLQGFPDYYKLIGPIKERYMQVGNAVAVPVARALGYSLAMSMKGLSGETPLFSLPQNFPFHEEQNCNEVSQ

>*Smel*DRM2

MDKNLSEEDSDNIDWDTEDELEIQDTTFSSCRDLRTTGQYAITGDGEASSSSASGQSKFIQKFVVMGFPEESIAKAIEQNGENSDLVLDALLTLKAIEDSPEEQPSASPHLEPCINSDDSSSEYNENFLDDVYEEDSWSSDSDFCLNSAKQCYLKEESSSLSEKEQTLLFLQNMGYPVEEVSIAMERCGPEASVAELTDFICAAQMARAEDPYLPEDVKPKINGNGGFKKRKMFNQLCKSKKPRAIFDEETIRLPKPMIGFGVPTEPVSAIVHRTLPEQAVGPPFFYYENVALAPKGVWDTISRFLYDIEPEFVDSKYFCAAARKRGYIHNLPIENRFPLLPLPPRTINEALPLTKKWWPSWDPRTKLNCLQTAIGSARLTDRIRKAVEAFDGEPPMRVQKYVLDQCRKWNLVWVGRNKVAPLEPDEFEMLLGFPKNHTRGGGISRTDRYKSLGNSFQVDTVAYHLSVLKDRFPNGMNVLSLFSGIGGAEVALYRLGIQLNNVVSVEKSEVNRNIVRSWWEQTNQRGNLIDFEDVQQLNGDRLEQLIDSFGGFDLLIGGSPCNNLAGSNRVSRDGLEGKESSLFYDYVRILDLVKSIMARQR

>*Smel*DRM3

MCEYSDGENSSKPEGVSGIMPKLEDPFGEFPSLYTYTTPIGANIASSSSSNVRSSLLTMGFEASLVDKAIEENGEDNIDLLLETLIANSDPPRAESSDSLDKLFGDDEDTNSSAKYDGDVHIKEEPDPCIGVCDDKRVSLLAMSFSLNEVEFAIGKLGEAAPVNELVDVIFAARIAGNHKKDDDDDVSVVEIKESKKECTTEALFGTMEKTLKLLEMGFSDNEVSKVIEKFGSEVPLEELANLIVDPSSGRRMNKHLLNPLGRNSSIGFHPVAVKKEEYSLDTSESREIDLLEKLKGKRPQESYIDERDTFKRPKSEYDEAFNNSLGPDWKEILGINNIRPKVCRRAIQQKSRVLDCQDKQKLSMPNPCRSLDKMVAKAPYFFYGNVMNLSHDSWVRISQFLYSIEPEFVHTQLFSALSRKEGYVHNLPTENRFHIVPKPPMTIREAVPNSKKWWPSWDTRQHLNCINSETSVVSQICDRLERIVSESQGFPSVDRQRDILYQSQIFNLVWVGRYKLAAVGPEQIEHILGYPENHTRLTGTSLMERLLSLKHCFQIDTLAYCLSTLKHLYPGGLTVLSIYSGIGGAEVALHRLGIRLKAVVSIESSEENRRILKQWWSSSGQTGELVQMEDIHKLASNKVELLIKNYGGFDFIICQNPCTYSSKGNLAADIDSHASLDFMLFHEFVRVMQRVRSTMGRN

>*At*CMT1

MAARNKQKKRAEPESDLCFAGKPMSVVESTIRWPHRYQSKKTKLQAPTKKPANKGGKKEDEEIIKQAKCHFDKALVDGVLINLNDDVYVTGLPGKLKFIAKVIELFEADDGVPYCRFRWYYRPEDTLIERFSHLVQPKRVFLSNDENDNPLTCIWSKVNIAKVPLPKITSRIEQRVIPPCDYYYDMKYEVPYLNFTSADDGSDASSSLSSDSALNCFENLHKDEKFLLDLYSGCGAMSTGFCMGASISGVKLITKWSVDINKFACDSLKLNHPETEVRNEAAEDFLALLKEWKRLCEKFSLVSSTEPVESISELEDEEVEENDDIDEASTGAELEPGEFEVEKFLGIMFGDPQGTGEKTLQLMVRWKGYNSSYDTWEPYSGLGNCKEKLKEYVIDGFKSHLLPLPGTVYTVCGGPPCQGISGYNRYRNNEAPLEDQKNQQLLVFLDIIDFLKPNYVLMENVVDLLRFSKGFLARHAVASFVAMNYQTRLGMMAAGSYGLPQLRNRVFLWAAQPSEKLPPYPLPTHEVAKKFNTPKEFKDLQVGRIQMEFLKLDNALTLADAISDLPPVTNYVANDVMDYNDAAPKTEFENFISLKRSETLLPAFGGDPTRRLFDHQPLVLGDDDLERVSYIPKQKGANYRDMPGVLVHNNKAEINPRFRAKLKSGKNVVPAYAISFIKGKSKKPFGRLWGDEIVNTVVTRAEPHNQCVIHPMQNRVLSVRENARLQGFPDCYKLCGTIKEKYIQVGNAVAVPVGVALGYAFGMASQGLTDDEPVIKLPFKYPECMQAKDQI

>*At*CMT2

MLSPAKCESEEAQAPLDLHSSSRSEPECLSLVLWCPNPEEAAPSSTRELIKLPDNGEMSLRRSTTLNCNSPEENGGEGRVSQRKSSRGKSQPLLMLTNGCQLRRSPRFRALHANFDNVCSVPVTKGGVSQRKFSRGKSQPLLTLTNGCQLRRSPRFRAVDGNFDSVCSVPVTGKFGSRKRKSNSALDKKESSDSEGLTFKDIAVIAKSLEMEIISECQYKNNVAEGRSRLQDPAKRKVDSDTLLYSSINSSKQSLGSNKRMRRSQRFMKGTENEGEENLGKSKGKGMSLASCSFRRSTRLSGTVETGNTETLNRRKDCGPALCGAEQVRGTERLVQISKKDHCCEAMKKCEGDGLVSSKQELLVFPSGCIKKTVNGCRDRTLGKPRSSGLNTDDIHTSSLKISKNDTSNGLTMTTALVEQDAMESLLQGKTSACGAADKGKTREMHVNSTVIYLSDSDEPSSIEYLNGDNLTQVESGSALSSGGNEGIVSLDLNNPTKSTKRKGKRVTRTAVQEQNKRSICFFIGEPLSCEEAQERWRWRYELKERKSKSRGQQSEDDEDKIVANVECHYSQAKVDGHTFSLGDFAYIKGEEEETHVGQIVEFFKTTDGESYFRVQWFYRATDTIMERQATNHDKRRLFYSTVMNDNPVDCLISKVTVLQVSPRVGLKPNSIKSDYYFDMEYCVEYSTFQTLRNPKTSENKLECCADVVPTESTESILKKKSFSGELPVLDLYSGCGGMSTGLSLGAKISGVDVVTKWAVDQNTAACKSLKLNHPNTQVRNDAAGDFLQLLKEWDKLCKRYVFNNDQRTDTLRSVNSTKETSGSSSSSDDDSDSEEYEVEKLVDICFGDHDKTGKNGLKFKVHWKGYRSDEDTWELAEELSNCQDAIREFVTSGFKSKILPLPGRVGVICGGPPCQGISGYNRHRNVDSPLNDERNQQIIVFMDIVEYLKPSYVLMENVVDILRMDKGSLGRYALSRLVNMRYQARLGIMTAGCYGLSQFRSRVFMWGAVPNKNLPPFPLPTHDVIVRYGLPLEFERNVVAYAEGQPRKLEKALVLKDAISDLPHVSNDEDREKLPYESLPKTDFQRYIRSTKRDLTGSAIDNCNKRTMLLHDHRPFHINEDDYARVCQIPKRKGANFRDLPGLIVRNNTVCRDPSMEPVILPSGKPLVPGYVFTFQQGKSKRPFARLWWDETVPTVLTVPTCHSQALLHPEQDRVLTIRESARLQGFPDYFQFCGTIKERYCQIGNAVAVSVSRALGYSLGMAFRGLARDEHLIKLPQNFSHSTYPQLQETIPH

>*At*CMT3

MAPKRKRPATKDDTTKSIPKPKKRAPKRAKTVKEEPVTVVEEGEKHVARFLDEPIPESEAKSTWPDRYKPIEVQPPKASSRKKTKDDEKVEIIRARCHYRRAIVDERQIYELNDDAYVQSGEGKDPFICKIIEMFEGANGKLYFTARWFYRPSDTVMKEFEILIKKKRVFFSEIQDTNELGLLEKKLNILMIPLNENTKETIPATENCDFFCDMNYFLPYDTFEAIQQETMMAISESSTISSDTDIREGAAAISEIGECSQETEGHKKATLLDLYSGCGAMSTGLCMGAQLSGLNLVTKWAVDMNAHACKSLQHNHPETNVRNMTAEDFLFLLKEWEKLCIHFSLRNSPNSEEYANLHGLNNVEDNEDVSEESENEDDGEVFTVDKIVGISFGVPKKLLKRGLYLKVRWLNYDDSHDTWEPIEGLSNCRGKIEEFVKLGYKSGILPLPGGVDVVCGGPPCQGISGHNRFRNLLDPLEDQKNKQLLVYMNIVEYLKPKFVLMENVVDMLKMAKGYLARFAVGRLLQMNYQVRNGMMAAGAYGLAQFRLRFFLWGALPSEIIPQFPLPTHDLVHRGNIVKEFQGNIVAYDEGHTVKLADKLLLKDVISDLPAVANSEKRDEITYDKDPTTPFQKFIRLRKDEASGSQSKSKSKKHVLYDHHPLNLNINDYERVCQVPKRKGANFRDFPGVIVGPGNVVKLEEGKERVKLESGKTLVPDYALTYVDGKSCKPFGRLWWDEIVPTVVTRAEPHNQVIIHPEQNRVLSIRENARLQGFPDDYKLFGPPKQKYIQVGNAVAVPVAKALGYALGTAFQGLAVGKDPLLTLPEGFAFMKPTLPSELA

>*At*DRM1

MVMSHIFLISQIQEVEHGDSDDVNWNTDDDELAIDNFQFSPSPVHISATSPNSIQNRISDETVASFVEMGFSTQMIARAIEETAGANMEPMMILETLFNYSASTEASSSKSKVINHFIAMGFPEEHVIKAMQEHGDEDVGEITNALLTYAEVDKLRESEDMNININDDDDDNLYSLSSDDEEDELNNSSNEDRILQALIKMGYLREDAAIAIERCGEDASMEEVVDFICAAQMARQFDEIYAEPDKKELMNNNKKRRTYTETPRKPNTDQLISLPKEMIGFGVPNHPGLMMHRPVPIPDIARGPPFFYYENVAMTPKGVWAKISSHLYDIVPEFVDSKHFCAAARKRGYIHNLPIQNRFQIQPPQHNTIQEAFPLTKRWWPSWDGRTKLNCLLTCIASSRLTEKIREALERYDGETPLDVQKWVMYECKKWNLVWVGKNKLAPLDADEMEKLLGFPRDHTRGGGISTTDRYKSLGNSFQVDTVAYHLSVLKPLFPNGINVLSLFTGIGGGEVALHRLQIKMNVVVSVEISDANRNILRSFWEQTNQKGILREFKDVQKLDDNTIERLMDEYGGFDLVIGGSPCNNLAGGNRHHRVGLGGEHSSLFFDYCRILEAVRRKARHMRR

>*At*DRM2

MVIWNNDDDDFLEIDNFQSSPRSSPIHAMQCRVENLAGVAVTTSSLSSPTETTDLVQMGFSDEVFATLFDMGFPVEMISRAIKETGPNVETSVIIDTISKYSSDCEAGSSKSKAIDHFLAMGFDEEKVVKAIQEHGEDNMEAIANALLSCPEAKKLPAAVEEEDGIDWSSSDDDTNYTDMLNSDDEKDPNSNENGSKIRSLVKMGFSELEASLAVERCGENVDIAELTDFLCAAQMAREFSEFYTEHEEQKPRHNIKKRRFESKGEPRSSVDDEPIRLPNPMIGFGVPNEPGLITHRSLPELARGPPFFYYENVALTPKGVWETISRHLFEIPPEFVDSKYFCVAARKRGYIHNLPINNRFQIQPPPKYTIHDAFPLSKRWWPEWDKRTKLNCILTCTGSAQLTNRIRVALEPYNEEPEPPKHVQRYVIDQCKKWNLVWVGKNKAAPLEPDEMESILGFPKNHTRGGGMSRTERFKSLGNSFQVDTVAYHLSVLKPIFPHGINVLSLFTGIGGGEVALHRLQIKMKLVVSVEISKVNRNILKDFWEQTNQTGELIEFSDIQHLTNDTIEGLMEKYGGFDLVIGGSPCNNLAGGNRVSRVGLEGDQSSLFFEYCRILEVVRARMRGS

>*At*DRM3

MADMRRRNGSGGSSNHERNEQILFPKPETLDFDLPCDTSFPQQIGDNAASSSGSNVKSLLIEMGFCPTLVQKAIDENGQDDFELLLEILTKSTETEPPGPSFHGLMEPKPEPDIEYETDRIRIALLTMKFPENLVDFALDRLGKDTPIDEMVDFIVAAQLAEKYAEESEDSLDGAEINEEDEDVTPVTARGPEVPNEQLFETMDKTLRLLEMGFSNDEISMAIEKIGTKGQISVLAESIVTGEFPAECHDDLEDIEKKVSAAAPAVNRTCLSKSWRFVGVGAQKEDGGGGSSSGTANIKPDPGIESFPFPATDNVGETSRGKRPKDEDENAYPEEYTGYDDRGKRLRPEDMGDSSSFMETPWMQDEWKDNTYEFPSVMQPRLSQSLGPKVARRPYFFYGQLGELSPSWWSKISGFLFGIHPEHVDTRLCSALRRTEGYLHNLPTVNRFNTLPNPRLTIQDAMPHMRSWWPQWDIRKHFNSGTCSNMKDATLLCERIGRRIAECKGKPTQQDQTLILRHCHTSNLIWIAPNILSPLEPEHLECIMGYPMNHTNIGGGRLAERLKLFDYCFQTDTLGYHLSVLKSMFPQGLTVLSLFSGIGGAEIALDRLGIHLKGVVSVESCGLSRNILKRWWQTSGQTGELVQIEEIKSLTAKRLETLMQRFGGFDFVICQNPSTPLDLSKEISNSEACEFDYTLFNEFARVTKRVRDMM

>*At*MET1

MVENGAKAAKRKKRPLPEIQEVEDVPRTRRPRRAAACTSFKEKSIRVCEKSATIEVKKQQIVEEEFLALRLTALETDVEDRPTRRLNDFVLFDSDGVPQPLEMLEIHDIFVSGAILPSDVCTDKEKEKGVRCTSFGRVEHWSISGYEDGSPVIWISTELADYDCRKPAASYRKVYDYFYEKARASVAVYKKLSKSSGGDPDIGLEELLAAVVRSMSSGSKYFSSGAAIIDFVISQGDFIYNQLAGLDETAKKHESSYVEIPVLVALREKSSKIDKPLQRERNPSNGVRIKEVSQVAESEALTSDQLVDGTDDDRRYAILLQDEENRKSMQQPRKNSSSGSASNMFYIKINEDEIANDYPLPSYYKTSEEETDELILYDASYEVQSEHLPHRMLHNWALYNSDLRFISLELLPMKQCDDIDVNIFGSGVVTDDNGSWISLNDPDSGSQSHDPDGMCIFLSQIKEWMIEFGSDDIISISIRTDVAWYRLGKPSKLYAPWWKPVLKTARVGISILTFLRVESRVARLSFADVTKRLSGLQANDKAYISSDPLAVERYLVVHGQIILQLFAVYPDDNVKRCPFVVGLASKLEDRHHTKWIIKKKKISLKELNLNPRAGMAPVASKRKAMQATTTRLVNRIWGEFYSNYSPEDPLQATAAENGEDEVEEEGGNGEEEVEEEGENGLTEDTVPEPVEVQKPHTPKKIRGSSGKREIKWDGESLGKTSAGEPLYQQALVGGEMVAVGGAVTLEVDDPDEMPAIYFVEYMFESTDHCKMLHGRFLQRGSMTVLGNAANERELFLTNECMTTQLKDIKGVASFEIRSRPWGHQYRKKNITADKLDWARALERKVKDLPTEYYCKSLYSPERGGFFSLPLSDIGRSSGFCTSCKIREDEEKRSTIKLNVSKTGFFINGIEYSVEDFVYVNPDSIGGLKEGSKTSFKSGRNIGLRAYVVCQLLEIVPKESRKADLGSFDVKVRRFYRPEDVSAEKAYASDIQELYFSQDTVVLPPGALEGKCEVRKKSDMPLSREYPISDHIFFCDLFFDTSKGSLKQLPANMKPKFSTIKDDTLLRKKKGKGVESEIESEIVKPVEPPKEIRLATLDIFAGCGGLSHGLKKAGVSDAKWAIEYEEPAGQAFKQNHPESTVFVDNCNVILRAIMEKGGDQDDCVSTTEANELAAKLTEEQKSTLPLPGQVDFINGGPPCQGFSGMNRFNQSSWSKVQCEMILAFLSFADYFRPRYFLLENVRTFVSFNKGQTFQLTLASLLEMGYQVRFGILEAGAYGVSQSRKRAFIWAAAPEEVLPEWPEPMHVFGVPKLKISLSQGLHYAAVRSTALGAPFRPITVRDTIGDLPSVENGDSRTNKEYKEVAVSWFQKEIRGNTIALTDHICKAMNELNLIRCKLIPTRPGADWHDLPKRKVTLSDGRVEEMIPFCLPNTAERHNGWKGLYGRLDWQGNFPTSVTDPQPMGKVGMCFHPEQHRILTVRECARSQGFPDSYEFAGNINHKHRQIGNAVPPPLAFALGRKLKEALHLKKSPQHQP

>*At*MET2a

MEMETKAGKQKKRSVDSDDDVSKERRPKRAAACTNFKEKSLRISDKSETVEAKKEQILAEEIVAIQLTSSLESNDDPRPNRRLTDFVLHDSEGVPQPVEMLELGDIFIEGVVLPLGDEKKEEKGVRFQSFGRVENWNISGYEDGSPVIWISTALADYDCRKPSKKYKKLYDYFFEKACACVEVFKSLSKNPDTSLDELLAAVSRSMSGSKIFSSGGAIQEFVISQGEFIYNQLAGLDETAKNHETCFVENRVLVSLRDHESNKIHKALSNVALRIDESKVVTSDHLVDGAEDEDVKYAKLIQEEEYRKSMERSRNKRSSTTSGGSSRFYIKISEDEIADDYPLPSYYKNTKEETDELVLFEAGYEVDTRDLPCRTLHNWTLYNSDSRMISLEVLPMRPCAEIDVTVFGSGVVAEDDGSGFCLDDSESSTSTQSNDHDGMNIFLSQIKEWMIEFGAEMIFVTLRTDMAWYRLGKPSKQYAPWFGTVMKTVRVGISIFNMLMRESRVAKLSYANVIKRLCGLEENDKAYISSKLLDVERYVVVHGQIILQLFEEYPDKDIKRCPFVTSLASKMQDIHHTKWIIKKKKKILQKGKNLNPRAGIAPVVSRMKAMQATTTRLVNRIWGEFYSIYSPEVPSEAINAENVEEEELEEVEEEDENEEDDPEENELEAVEIQNSPTPKKIKGISEDMEIKWDGEILGKTSAGEPLYGRAFVGGDVVVVGSAVILEVDDQDDTQLICFVEFMFESSNHSKMLHGKLLQRGSETVLGMAANERELFLTNECLTVQLKDIKGTVSLEIRSRLWGHQYRKENIDVDKLDRARAEERKTNGLPTDYYCKSLYSPERGGFFSLPRNDMGLGSGFCSSCKIRENEEERSKTKLNDSKTGFLSNGIEYHNGDFVYVLPNYITKDGLKKGSRRTTLKCGRNVGLKAFVVCQLLDVIVLEESRKASKASFQVKLTRFYRPEDISEEKAYASDIQELYYSQDTYILPPEAIQGKCEVRKKSDMPLCREYPILDHIFFCEVFYDSSTGYLKQFPANMKLKFSTIKDETLLREKKGKGVETGTSSGMLMKPDEVPKEKPLATLDIFAGCGGLSHGLENAGVSTTKWAIEYEEPAGHAFKQNHPEATVFVDNCNVILRAIMEKCGDVDDCVSTVEAAELAAKLDENQKSTLPLPGQVDFINGGPPCQGFSGMNRFSHGSWSKVQCEMILAFLSFADYFRPKYFLLENVKKFVTYNKGRTFQLTMASLLEMGYQVRFGILEAGTYGVSQPRKRVIIWAASPEEVLPEWPEPMHVFDNPGSKISLPRGLRYDAGCNTKFGAPFRSITVRDTIGDLPPVENGESKINKEYGTTPASWFQKKIRGNMSVLTDHICKGLNELNLIRCKKIPKRPGADWRDLPDENVTLSNGLVEKLRPLALSKTAKNHNEWKGLYGRLDWQGNLPISITDPQPMGKVGMCFHPEQDRIITVRECARSQGFPDSYEFSGTTKHKHRQIGNAVPPPLAFALGRKLKEALYLKSSLQHQS

>*At*MET2b

METKVGKQKKRSVDSNDDVSKERRPKRAAACRNFKEKPLRISDKSETVEAKKEQNVVEEIVAIQLTSSLESNDDPRPNRRLTDFVLHNSDGVPQPVEMLELGDIFLEGVVLPLGDDKNEEKGVRFQSFGRVENWNISGYEDGSPGIWISTALADYDCRKPASKYKKIYDYFFEKACACVEVFKSLSKNPDTSLDELLAAVARSMSGSKIFSSGGAIQEFVISQGEFIYNQLAGLDETAKNHETCFVENSVLVSLRDHESSKIHKALSNVALRIDESQLVKSDHLVDGAEAEDVRYAKLIQEEEYRISMERSRNKRSSTTSASNKFYIKINEHEIANDYPLPSYYKNTKEETDELLLFEPGYEVDTRDLPCRTLHNWALYNSDSRMISLEVLPMRPCAEIDVTVFGSGVVAEDDGSGFCLDDSESSTSTQSNVHDGMNIFLSQIKEWMIEFGAEMIFVTLRTDMAWYRLGKPSKQYAPWFETVMKTVRVAISIFNMLMRESRVAKLSYANVIKRLCGLEENDKAYISSKLLDVERYVVVHGQIILQLFEEYPDKDIKRCPFVTGLASKMQDIHHTKWIIKRKKKILQKGKNLNPRAGLAHVVTRMKPMQATTTRLVNRIWGEFYSIYSPEVPSEAIHEVEEEEIEEDEEEDENEEDDIEEEAVEVQKSHTPKKSRGNSEDMEIKWNGEILGETSDGEPLYGRALVGGETVAVGSAVILEVDDPDETPAIYFVEFMFESSDQCKMLHGKLLQRGSETVIGTAANERELFLTNECLTVHLKDIKGTVSLDIRSRPWGHQYRKENLVVDKLDRARAEERKANGLPTEYYCKSLYSPERGGFFSLPRNDIGLGSGFCSSCKIKEEEEERSKTKLNISKTGVFSNGIEYYNGDFVYVLPNYITKDGLKKGTSRRTTLKCGRNVGLKAFVVCQLLDVIVLEESRKASNASFQVKLTRFYRPEDISEEKAYASDIQELYYSHDTYILPPEALQGKCEVRKKNDMPLCREYPILDHIFFCEVFYDSSTGYLKQFPANMKLKFSTIKDETLLREKKGKGVETGTSSGILMKPDEVPKEMRLATLDIFAGCGGLSHGLEKAGVSNTKWAIEYEEPAGHAFKQNHPEATVFVDNCNVILRAIMEKCGDVDDCVSTVEAAELVAKLDENQKSTLPLPGQADFISGGPPCQGFSGMNRFSDGSWSKVQCEMILAFLSFADYFRPKYFLLENVKKFVTYNKGRTFQLTMASLLEIGYQVRFGILEAGTYGVSQPRKRVIIWAASPEEVLPEWPEPMHVFDNPGSKISLPRGLHYDTVRNTKFGAPFRSITVRDTIGDLPLVENGESKINKEYRTTPVSWFQKKIRGNMSVLTDHICKGLNELNLIRCKKIPKRPGADWRDLPDENVTLSNGLVEKLRPLALSKTAKNHNEWKGLYGRLDWQGNLPISITDPQPMGKVGMCFHPEQDRIITVRECARSQGFPDSYEFSGTTKHKHRQIGNAVPPPLAFALGRKLKEALYLKSSLQHQS

>*At*MET3

MKTKAGKQKKRSVDSDDDVSRERRPKRATSGTNFKEKSLRFSEKYETVEAKKEQIVGDDEKEEKGVRFQSFGRVENWTISGYEDGSPVIWISTVIADYDCRKPSKKYKKLYDYFFEKACACVEVCKNLSTNPDTSLKELLAAVVRSMNGRKIFSSGGVIQEFVISQGEFIYNQLAGLDETSKNHETKFVDNRVLVSLRDESRKIHKAFSNVALRIDESKVLTSDQLMDGGEDEDLKYAKLLQEEEHMKSMDRSRNKRSSTTSAPNKFYIKINEDEIAHDYPLPSYYKNTKDETDELVLFNAGYAVDARNLPCRTLHNWALYNSDLMLISLEFLPMKPCADIDVTYLGQIKEWKIDFGEDMIFVLLRTDMAWYRLGKPSEQYAPWFEPILKTVRIGTSILALLKNETRMAKLSYTDVIKRLCGLEENDQAYISSTFFDVERYVIVHGQIILQFLTECPDEYIKRCPFVTGLASKMQDRHHTKWIIKKKRKMLQKGENLNLRRGKAPKVSKMKAMQATTTRLINRIWGEFYSIYSPEDPLEEIGAEEEFEEVEDVEEEDENEEEDTIQKAIEVQKADTLKKIRGSCKEMEIRWEGEILGETCAGEPLYGQALVGGRKMDVGGAVILEVDDQGETPLIYFVEYMFESSDNSKKLHGKLLQRGSETVLGTAANERELFLTNECLTVQLKDIKGTVSFEIRSRPWGHQYKKEHMAADKLDRARAEERKAKDLPIEYYCKSLYSPEKGGFFSLPRSDMGLGSGFCSSCKIRENEEERSKTKLNDSKTRFLSNGIKYSVGDFVYQIPNYLSKDRGKRRPVFKYGRNVGLRAFVVCQILDIVDLKEPKKGNTTSFEVKVRRFYRPDDVSAEEAYASDIQEVYYSEDTYILPPEAIKGKCEVMKKTDMPLCREYPILDHVYFCDRFYDSSNGCLKKLPYNMMLKFSTIKDDTLLREKKTETGSAMLLKPDEVPKGKRLATLDIFAGCGGLSYGLEKAGVSDTKWAIEYEEPAAQAFKQNHPKTTVFVDNCNVILRISWLRLLINDRAIMEKCGDVDDCISTTEAAELATKLDENQKSTLPLPGQVDFISGGPPCQGFSRLNRFSDGSWSKNQCQMILAFLSFADYFRPKYFLLENVKTFVSFNEGHTFHLTVASLLEMGYQVRFGLLEAGAYGISQPRKRAFIWAAAPNEVLPEWPEPMHVFNNPGFKIPLSQGLHYAAVQSTKFGAPFRSITVRDAIGDLPPIESGESKINKEEMRGSMTVLTDHICKKMNELNLIRCKKIPKTPGADWRDLPDEHVNLSNGIVKNIVPNLLNKAKDHNGYKGLYGRLDWHGNLPTCITNLQPMGLVGMCFHPDQDRIISVRECARSQGFPDSYKFSGNIKDKHRQVGNAVPPPLAFALGRKLKEALHLRNI

>*Cc*CMT3_like2

MARGKRKSSAANDDASASSSILEKKPKLVEDKNEEISSNVGVVEASAVLDRVSPAKRSGQKLDKPKKEDEEDEDEVESRFIGDPVPDDEARQRWPHRYATINNNDINFAKKKAGKSGSLETLKGMAPQKELMQARRHFSEALVDGRVNFKLGDDGYVQAGEGEDSYICRIVEMFEGMDCALYFSAQWFYRAKDTIIQACSDLIDDKRVFLSEIKDDNPLDCLLEKLKIVRVPLDADIADKQARLADGDYYYDMSYLVPYSTYQNLPPVYGKHLSYILVSERLKFLLYEQPITDNDGDGNESDSTISSESDVNGVVSEVPQVQDGKRSEMRMLDLYSGCGAMSTGLCLGANMGDVNLVTRWAIDLNRYACESLKLNHPETEVRNESAEDFLMLLKEWEKLCQSFSLVGGGDSQQRMNPASIEEDEGEADDDDDDDSDGLDEEVFEVEKVLSICYGDPKEIKKPGLYLKIRWKGYGPEEDTWEPMEGLCDCHDKIKAFVVKGFNSKILPLPGDVDVICGGPPCQGISGFNRFRNKDKPLEDEKNKQLVVYMDIVEYLKPRFALMENVVDIVKFAKGFLGRYALGRLVSMNYQARVGLMVAGSYGLPQFRRRMFMWGARPSEFNLFNTSFDVQKLPQYPLPTHNVVTRGVSPLEFESNTVVHEEGQKVELEKELFLGDAISDLPPVPNDETRDEMPYEEMPKTEFQKFIRLKKDDMPGFSASGQDSSDHLLYDHRPLKLNDDDYQRVCQIPKRKGANFRDLKGVRVRKDNHVEWDPDVERVYLPSGKPLVPDYAMTFVDGRSSKPFGRMWWDETVPTVVTRAEPHNQAILHPLQDRVLTIRENARLQGFPDYYKLVGPIKERYIQVGNAVAVPVARALGYSLAMSCKGSSGAEATFTLPSKFPNIQPVTSPSVDQQNQ

>*Cc*CMT3_like3

MPSSKRKSRASLKDADLASPTSEKRPKSEEEEKGEILPNADAVEAPAVNNGDSSVRSSGRRSENNKEEAELNDDEESRLVGDPIPDDEARQRWPHRYLGKNDAICYLYGTSLTEPQKELIQARRHFTEALVDGCIPFKLGDDGYVQAGEGEESYICRIVELFEGVDGAPYFYAQWFYRAKDTACSNLIDDKRVFLSEIKDDNPLDCLLQKLKIVRVPLDVDVASKRAMLLDGNYYYDMLYLVPYSTYQNLPLVGSHSSNVLKLKTFIGTQQLYLYTDNEADGNESESTISSESDSNAAVTETSKIQEDDKSEMRMLDLYSGCGAMSTGLCLGANMADVNLVTRWAVDLNRYACESLKLNHPETEVRNESAEDFLWLLKEWHKLCQSFSLVGGGDSQLSGDPMIVKEDETEDAVDDDDDNDGLDEEVFEVDKILSICYGDPKGLKKPGLYLKIRWKNYGPAEDTWEPIDGLGDCQEKIKQFVVNGFKSKILPLPGDVDVICGGPPCQGISGFNRYRNKDKPLEDEKNKQLVVYMDIVEYLKPRFALMENVVDIVKFAKGFLGRYALGRLVSMNYQVRIGLMTAGSYGLPQFRMRMFMWGARPSEKLPQYPLPTHNVVARGVSPVEFESNAVVYDEVSGIELEKELFLGDAISDLPPVANDEERDEIPYQEMPKTEFQKFIRLKKEDMPGFSMVGVESSDHPLYDHRPYKLNDDDYLRVCQIPKRKGANFRDLKGVQVRDDNHVEWDPDVERVYLPSGKPLVPDYAMTFVDGRSSKPFGRLWWDEIVPTVVTRAEPHNQAILHPLQDRVLTIRENARLQGFPDYYKLVGPIKERYTQVGNAVAVPVARALGYSLAMSCKGTASEGPVFRLPERFPNIEAVPVAVAVEENQ

>*Cc*CMT2_like1

MVNSAGTGEGSGSCPPSKPPMAPSSSQPSSITANYDSPALHFGQISRRRSPRLLNCSKDGKDSSVDFNKCPKQKKLRTTSPSDSFPSSPNDAPFLIGDPVSDEEARRRWPWRYECKVLSVMEVLIFSRVPDRGDDNEWALLFFLEHEGYLRSDLFRPSVTQTKKEPFKDDDDKLIANVKCHYLQAKVETHVFDLGDCACVKGEEDGHNHVGRILEFFKTNDSKDFFRIQWFFRAEDTDEAASHHKKRLFYSTLRNDNALDCIVSKVDFRSIPACDYYYDMKYNIDYSTYCTIKDDDCCLFSSHNKKEMHSNGSKTNLNGTLTSFKPHKLELSLLDMYSGCGGMSTGLCFGAKLSGVDLSTIRNESAEDFLDLIKEWDKLCKKYMVKEEKTQGNDSTFAGSADDKPSKAKKIVPEDEYEVERLVDICYSDLDGTSKRGLKFKVRWAGYGPSDDTWEPIKELSNCQEKIREFVQKGIKTNKLPRPGDVDIICGGPPCQGISGFNRHRNFESPLEDEKNYQIVVFMDIINFLRPKYILMENVVDILRFANGRLARYAISCLVRDYYQVRLGIMAAGCYGLPQFRLRQLPPFPLPTHDVVFKYGGASGFERNVVAYDEGQFRNLEKPVLLKDAISDLPLVSNSEVRDKMMYRSAPETEFQKYIRATKSDMLGIASGSSSEVEKSVLHDHRPLQLNEDDYLRGANFRELPGIVVDDDNVVSRAPEAELMPSGKHWVPDYAINLHERKSTKPFARLWWDETVSTVICTPNFRCEAVLHPEQDRVLTIRENARLQGFPDFYALCGTVNERYRQVGNAVAVPVGRALGYTLGMAVQKLCGDEPLITLPPEFAHSTTLDLLQASSLATEP

>*Cc*DRM2_like2

MGDHAPGDDSENIDWDTEDELEIQNIAPSSCAQLITRDTEAVISNGKASSSAGPSNTKLVEHFLGMGFREQWVTKAIEKNGEGDHESILDTLFAYQRFKCGDYELPAKRVVAPGLVGSHSGLDIQGVQIFGLLMRISVLQALEDPQQGHNSCHPNHLNSPQQQQCVNDDDLSSDYDESLLDDFSESDSWLGSETEDFDSLPEHEKTLKYLVAMGYTEEEASIAMERCELAYISTNPMGPGEQRNLLWPCGTRTGPGASIAVLTDFIGAAQNAKTEPVFFEDEKRKNLGENGKLKKRKLYELEAWKRNKQKGPLTEQDEVIRLPNPMTGFGVPSEPSSMVTHRTLPDDAIGPPFFYYENVALAPKGSWDTISRFLYDVEPEFVDSKYFCAAARKRGYVHNLPINNRFCILPLPPRTISDALPLTKRWWPEWDKRTKLNCLQTVIASAKLTERIRKDLEKYSDYVPLDVQKKVIEDCRKWNLVWVGKNKVAPLEPDEVEMLLGFPRNHTRGGGISRTDRYKSLGNSFQGNPCQIGFGDWLRVFMPSLPKRLDSTSNPGYRWSTHSLGYRSANNPLVDTVAYHLSVLKDIFPKGINMLSLFSGIGGAEVALHRLGILLNNIVSVEISEANRDIVRSWWEQTNQKGNLVHLPDVQQLNGDQLEQFISSFGGFDLVVGGSPCNNLTGSNRVSRDGLQALDDCLVVGYWLNDIESGYLSNAACIAPSKCFAFGTCVGIVLRPNVKVRLITGLDWTGWARVKKTGEEL

>*Cc*CMT2_like2

MFTGKKSEPCDEVSRTNSASKMVVRGSTRMNSDINVAKWGKFSTTGSDALALTLHSLSSDFYYNSDESPVPLKVYDPSLAHGKSVRRSPRFTMGESIITTKGASKNKSVLGERVEGSSSGRSSRLSSSTALIVGTLDRQRSPKSKALPRRRLRRSPRLSPVTSHAENAGSNKLKRLPQKCLRRSPRLSPIPSCPQIEWEGLHTRVKGERTTDGPKLKRIKSCVEELNSNVVTEKCTKKSSARVQHGNGNCEKLAVPALSSAETVKDDFIPNGDTARPCGLEFHVRKKEDSEVKSTSIRTHDKKAASVGNIDFGYNKAPPVRCKTSVSSTKQQSSTKKQRKLKRASFFVGEPVPEDEAQERWHWRYELKFFAVSKIRVTSEMGFEEEVNGVFQRKLGAETDLNKGKTGQRRKGQSWILNAGEEDELHLNVMCHYLQANVDGCIYRLGDCARIKLHGENNNYKDKDEHRTYTSESASKNRARAQLLIHGEGKREHVGRIVEFFKTSDDENYFRVQWFFRAEDTVMKQAAAFHEKKRLFSSTLMNDNLLDCILSKVKITEKAPALGLQSAIQPSEYYCDMEYSVKYSTFRSLATATNSLVARCDLTLPSSLDANNVTITTTPLELSSCEPYKAELALLDLYAGCGGMSTGLCLGAKISGVKLVTRWAIDYHKSACDSFKQNHPETQVRHITAEDFLELLKEWEKLCKLYVLNDTDRGLETSSNRTKKSIIRESSLLDAEVAPGEYEVSSLVDICYGDPSSTGKHGLKFKVRWKGYSPSEDTWELIQDLSDCQGHIKDFVRSGYMSKILPRPGEVDVICGGPPCQGISGYNRFRNTDDPLTDERNQQIIVFFDIVNFLKPKYALMENVADILRYDKASLGRYAISRLVHMNYQSRLGIMAAGSYGLPQFRLRVFLWGALSSETLPQFPLPTHEVIVRYFPSAEFEQNTVAYDEGQPRELKEATVLRDAISDLPAVTSHEDREEMAYDMPPETEFQKYIRLTKDEINGSTLKGVTDWRSSVLTDHRPYKLSEDDFHRVCHVPRRKGANFRDFPGLVVGADNLVRRDPTKEPVLLPSGRPLVPDYVFTFEKGKSKRPFSRIWWDENVPTVVTFPNLHSQRAIHPEQDRVLTIREYARLQGFPDHYRFCGTVKERYCQVGNAVAVFVSKTLGYALGMAFQKLSGDEALMTLPPDFAFQVPPLDQFSAQL

>*Cc*CMT3_like1

MAKRVKRDLKQSEEAIDSSVPSSDSKSSPPSKKSKTSPAAAAADDDARFIGKPVPADQARAKWPHRYESKNKVKVIASSNGELDGKEIIQAKCHYTKAVVDGIAFDLNDDAFVKAEEGKPDFIARIVEMFETVDRELYCSAQWFFRAEDTVIKSQAHLIDKRRVFYSDMKDDNPLDSIVSKVKIVQLSPNVDLAEKEKALSSFDLYYDMKYSMPVTFTTLHTEKSITESDESSVISGDASSDGVVEKSNKKAKSTEVKECVESQMTLLDLYSGCGAMSTGLCHGTNMSGVKLVTKWAVDINKHACESLKLNHTETEVRNEAAEDFLSLLKEWKNLCKEFCLLGSQHAEDTSIKSEESDSQEKEGNPDPSDGEFEVGKLLAVCYGDPNKVNNKKLHFKVFYFPIVVEVRWKGYGPSYDTWEPFDGLSNCTDAMKEFVSRGYQSRILPLPGDVDFICGGPPCQGISGHNRFRNYTDPLKDPKNHQLVVYMDIIEFLKPKFVLMENVCDIVKFADGILGYHAVGRLVSMNYQTRMGIMAAGSYGDCIVGSDNDKSYKLEKSILLGDAISDLPEVTNNNGKDEMEYAGAPRTSFQKYIRMRKQAVAKDASKRKMLYDHRPLELNEDDYARVCQIPKIKGANFRNLPGVMVGKNNKVEWDPSVERVMLPSGKPLVPNYAMTFVRGTSRKPFGRLSMDDIVTTVVGRAEPHNQVLLHPNQDRVLTIRENARLQGFPDHYKLSGPVKERYLQIGNAVSFSVSTALGYTLAKAVQGVCTSKPLTLPIKFPDCLGQSTVKQAPQESE

>*Cc*MET1_like

MKKKGKQVKATTEMSDVTVDLKTKAATKPKQKRGRSGSSEDVPVSRKMPKRAASCTNFKSKPLRLSEKSATIENKKVQVVEEEIAAISLIAGPDDPRPNRRLTDFVFHDADGMPQPVEMLEVDDIFISGIILPLEKASEKEKETGVRCDGFGRIEDWSISGYEDGSPVIWISTELADYDCVKPAASYKKLFGLFYEKAHACVEVYKRLSKSAGGNPELSLDELLAALVRSMSGSKNFPHGASIRDLIISWGGFIFEQLVGLDEAPNGTDQPFVEIPVLAALRDESKKDEECAGMNVPPGGLMNAPLKISDGEKASKSNGPKPAVDEDEDMKLARVLQENENWQALKQKKRQRTSTSSSKLYIQINEDEIANDYPLPAYYKTTAQETDEYIIFDDFDTVDPDQLPRSMLHNWSLYNCDSRLISLELLPMKPCADIDVTVFGSGIMTADDGSGFCLDNEAGSASGSSGVQNEDGIPIYLSAIKEWMIEFGSSMVFISIRTDMAWYRLGKPSKQYAPWYQTVLKTARLAIAIITLLKEQTRASKLSFSEVIKRLYVVVHGQIILQQFAEFPDDTIRRSAFVSGLEDKMEERHHTKWLVKKKAILTKAENMNPRAAMGPVISKRKAMPATTTQLINRIWGEFYSNYSPEEVKEGDSLDAKKDEEEEEQEENEQEDCEEPEEENPILPQEPEKPSPASKQKKTRCSKTDINWVGQAVRKMCDGKALYKEAVIRGEVVALGSSVLVETSGSEEDSIYYVEYLFEDSDSRKFVHGRLMLRGRQTVLGDIANEREVFLTNDCMEFELDDVIQTVVVQIRTLPWGFEHRKANANFDKMDRAKAEDRKNKGLPIEFYCKSLYWPQRGAFFCLQTDKMGLGNGVCHSCEFMEAEKKKEAFVLDESKTGFTYMATEYHVDDFLYVGPHHFDTDERGNETYKGGRNVGLKAYVVCQLQQIEAPKTSKRADPDSVMVQVRRFYRPEDLSSDKAYRSDIQEVYYSEQVHKLPLSAIEGKCEVRRKKDLSSLDSTYIFEHVFFCERLYDPAKGSLKQLPVHIKLTPPKESLVSDAAIRKRKGKSKEGENDVDMIDNQESSASKNCLATLDIFAGCGGLSEGLQKAGASVTKWAIEYEEPAGDAFKLNHPDALAFVHNCNVILRAIMTACGDTDDCISTSEADELAAKLEEDVINNLPRPGQVDFINGGPPCQGFSGMNRFNQSTWSKVQCEMILAFLSFAEYFRPKFFLLENVRNFVSFNKGQTFRLALASLLEMGYQVRFGILEAGAFGVSQSRKRAFIWAASPEEVLPEWPEPMHVFAGPELKVTLNRNTQYAAARSTATGAPFRAITVRDTIGDLPAVGNGASAATIEYKNESVSWFQKRIRGDASVLTDHISKEMNELNVIRCQRIPKRPGADWRDLPEEKVKLSTGQMVDLIPWCLPNTAKRHNQWKGLFGRLDWEGNFPTSITDPQPMGKVGMCFHPDQDRILTVRECARSQGFPDRYKFSGNIQHKHKQIGNAVPPPLAYALGRKLKEAVEAKQRQLDHHSCL

>*Cc*DRM2_like1

MDGDSSHEGSDNIDWNTDDELEIANISPSSPVSTSTNGAVISEFGEVVHNCTQIIADYPTPGMIVIYEPLISDGSKRISWSLCYKAFGFGISIGEVAPRDVIGWTHDLRRYFRGYFVISCLTLVVIAKAPFKPRSILQIPPTMSSSSKCPSKLQFMTMGFPEAMVTKVIAELGEDNTDAIVDTLLTYSIDSLGENEMDVVVDETPNQERELNDPYLKSESHPESKFSDLDSIWSDEDSDESFEKDDPLVCLIDMGYSPEEASAAISRCGKNAPLSELVDFISAANVSKEYDAEMNANVLGSCSHLLPKDKKRKFQKDNFWSKNKKFDRKNESKSKDEDHEVLHLPNPMVGFGVPREPFHVVRRTLPEAAIGPPFFYYENVALTPKGAWNRIKSFLYEIDPEFVDSKFFCAAARKRGYIHNLPLENRFPIQPLPPLTIFEALPGTKKWWPSWDKREQLNCILTCIGSAQLTDRIRLALENSNTEPSLHVKNYIIGQCRKWNMVWTGKTRVAPLEPDEIELIMGYPIYHTRGASRVERYKGLGNAFQVGTLKPPTFYFFNRYYHLKAGLHIFQAKCLIGIQHLVIYILHLIFYTFGGKTSRVFQQEFHILEVDTVAYHLSVLKNLYPNGMNILSLFSGIGGAEIALHKLGIPLNNVVSVEKSMVCRNILQGWWEQTNQKGNLVHLSDVQDVTLNKLNQWIDSFGGFDLVIGGSPCNNLAGGNRRTRDGLEGGHSSLFFDYFRILDGVKNLMKNRS

>*Cc*DRM2_like3

MDGNASGEDYENIDWDTEDELEIQNIVPSTCSNLVTRNASIVGNGEASSSAGPSNPNLLQHFLGMGFSEQLIVKAIKENGEANTESILESLLTYAALEDSPDELNPCHLNSPQQQQCVDNGQLSSDYDESFLDDISESDSWSGSEVAKPKISIGANGGLKKRKLYELELWKRKKRKGLINEEDDVLRLPNPMIGFGVPTDTMVVTHRTLPEAAIGPPFFYYENVALAPKGVWDTISRFLYDVQPEFVDSKYFCATARKRGYVHNLPIHNRFPLLPLPPRTINDALPLTKRWWPEWDKRTKLNCLQTVIGSAKLTDRIRKALEKWGDDPPLHVQKYVIEECRKWNLVWVGKNKLAPLEPDEFEMLLGFPRNHTRGGGISRTDRYKSLGNSFQDMFPNGLNLLSLFSGIGGAEVALHRLGIPLKNVVSVEISEANRDIVRSWWEQTNQKGNLIHLADVQQLNGDRLEQFMGSFGGFDLIVGGSPCNNLAGSNRVSRDGLEGEQSSLFYDYFRILDLVKCIMNKQQ

>*Cc*DRM3_like1

MDKTLRLLEIGFSEQEISAAIEKYGSEVSISELADSIVCDRMGGPCIKTEEDPFGANSWMTGNKFKSSSMGAERVLDASFYSNLALRTEESSQAAASQIRDFDIGDSCKGKQPKEETADELITIQRPKPEFDDLNSYSGPACTVPKPPVSSKVLQRQLKYKARRMAATGVPKLIQPVSCSSVDQMVAKAPLFFYGNVMNLSQDSWVKISQFLYAIEPEFVNTQFFSALSRKEGYIHNLPTKNRFHILPKPPMTIEEVIPQTKKYWPSWDTRKQLTCINSETIGISQLCDRLRNILIDSKGLLSVEQQKDLLHQCRSLNLMWVGRNRVSPIEPELVERILGYPMYHTREDGLSLGERLQSLKHSFQTDTLGYHLSVLKSMYPEGLTLLSIYSGVGGAEITLNRLGIRLKAVVSVEPSEIKRKILRQWWDKSDQTGELVQIENIQKLSSSKLESLIKKFGVFDFIICQNPYTYAPKSVTMAAAETESFAGLDFSLFYEFVRVLQRVRSAIKTR

>*Gm*CMT1
MPSKRKTRSSASPAAAPPSKRASRSSASRVADSAPVKSEAEEVVAASSVVKEEAQASFTDVTDGNVSDGEGTNARFVGEPVPDEEARRRWPKRYQEKEKKQSAGPKSNRNDEDEEIQQARRHYTQAEVDGCMLYKLYDDAHVKAEEGEDNYICKIVEIFEAIDGALYFTAQWYYRAKDTVIKKLAYLIEPKRVFFSEVQDDNPLDCLVEKLNIARITLNVDLEAKKETIPPCDYYCDTQYLLPYSTFVNLPSENGESGSETSSTISSETNGIGKYEVNSQPKEAFLPEESKDPEMKLLDLYCGCGAMSTGLCLGGNLSGVNLVTRWAVDLNQHACECLKLNHPETEVRNESAENFLSLLKEWQELCSYFSLVEKKVSHEKYVNLFSEDDDDTSSNEEVNSEDDNELNEDDEIFEVSEILAVCYGDPNKKKEQGLYFKVHWKGYESALDSWEPIEGLSNCKEKIKEFVSRGFKSQILPLPGDVDVICGGPPCQGISGFNRFRNKESPLDDEKNKQLVVFMDIVQYLKPKFTLMENVVDLVKFAEGFLGRYALGRLLQMNYQARLGIMAAGAYGLPQFRLRVFLWGAAPSQKLPQFPLPTHDVIVRGVIPLEFEINTVAYNEGQKVQLQKKLLLEDAISDLPRVQNNERRDEIKYDKAAQTEFQRFIRLSKHEMLELQSRTKSSKSLLYDHRPLELNADDYQRVCRIPKKKGGCFRDLPGVRVGADNKVEWDPDVERVYLDSGKPLVPDYAMTFVNGTSSKPFARLWWDETVPTVVTRAEPHNQAILHPEQDRVLTIRENARLQGFPDFYKLCGPVKERYIQVGNAVAVPVARALGYTLGLAFEGSTSTSDDPLYKLPDKFPMIRDRVSSVSSEDDV

>*Gm*CMT4
MNDNLIDCIMGKANVTHITPRVGLKLASISSSDFYYDMEYCVDYSTFRNIPTDASTVTESQPCSELNKTELALLDLYSGCGGMSTGLCLGAKTASVNLVTRWAVDSDRSAGESLKLNHSDTHVRNESAEDFLELLKAWEKLCKRYNVSSTERKLPFRSNSSGAKKRGNSEVHEISDGELEVSKLVDICFGDPNETGKRGLYLKVHWKGYSASEDTWEPIKSLSKCKESMQDFVRKGMKSNILPLPGEVDVICGGPPCQGISGYNRFRNCASPLDDERNRQIVIFMDMVKFLKPRYVLMENVVDILRFDKGSLGRYALSRLVHMNYQARLGIIAAGCYGLPQFRLRVFLWGAHPSEVIPQFPLPTHDVIVRYWPPPEFERNVVAYDEEQPRELEKATVIQDAISDLPAVMNTETRDEMPYQNPPETEFQRYIRSTKYEMTGSKSNGTTEKRPLLYDHRPYFLFEDDYLRVCQIPKRKGANFRDLPGVIVGADNVVRRHPTENPLLPSGKPLVPEYCFTFEHGKSKRPFARLWWDENLPTALTFPSCHNQVVLHPEQDRVLTIREFARLQGFPDYYRFYGTVKERYCQIGNAVAVPVSRALGYALGLACRKLNGNEPLVTLPSKFSHSNYLQLSKCVFGNTSNEVNSRQFRALDAEVTPGSIGQDSRVEDSTQLQTCYNNQPGNTD

>*Gm*MET1
MGSASLLNPSQPGVKKNDKSKQKSVVSKTEEEVMFKDKQKKRSLSESSEQTAAMRKMPKRAAACKNLKEKSFLIYEKSCLIETEKDHIVEEESLAVRMTAGQDNGCPNRRITEFILHDETGKSQPLEVLEVDDLFITGLVLPLEASSGKKKEKGVKCEGFGRIESWDISGYEDGSPVIWLSTEVADYDCQKPAASYKKVYDLFLEKARACVEVYKKLAKSSGGDPDISLDELLAGMVRSMSGSKCFSGAASIKDFVISQGEFIYKQLVGLDMTSKANDRMFADIPALIALRDESKKQVHAQVMPSNGSLRIDSGVGDEENKNQMDSVASVNEEDEDAKLARLLQEEEYWQSMNQKKNSRSASASNKYYIKINEDEIANDYPLPVYYKTSLQETDEFIVFDNDYDIYDTQDLPRSMLHNWSLYNSDARLVSLELLPMKPCSDIDVAIFGSGIMTSDDGSGFHLDTEAGKSSSVGSGAQVADGMPIYLSAIKEWMIEFGSSMIFISIRTDLAWYRLGKPAKQYAPWYDTVLKTARLAISIITLLKEQSRVSRLSFGDVIRKVSEFDKKDGSYISSDPLTVERYVVVHGQIILQLFAEFPDDKIRKSAFVTGLTNKMEERHHTKWLVKKKKVVPRSEPNLNPRAAVGPVVSKRKAMQATTTRLINRIWGEYYSNHLPEDAKEGIASELKDEDEVEEQEENEDDDNEETILLEGTPKAHSASKQTKKFSAETEIRWEGEPEGKTSSGYPVYKQAIIRGEVISVGRSVLVEVDETDEFPDIYYVEYMFESKIGRKMFHGRMMQRGCQTVLGNAANEREVFLTNECRDLGLHDVNQTVVVNIQNRPWGHQHRKDNIIADRVDRAQAEERKKKGLPTEYYCKSLYWPERGAFFSLPLDTLGLGSGVCPSCKIQDAEKEKDVFKVNSSKSGFLLKGTEYSLNDYIYVSPFEFEEMIEQGTHKSGRNVGLKAYVVCQVLEIVVKKEIKEAEIKSTQVKIRRFFRPEDVSNEKAYCSDIQEVYYSDETHIISVESIEGKCQVRKKNDIPECSALGRMFQNVFFCELLYDPATGSLKKLPAHVKVKYSSGQTSDAAARKRKGKCIEGDDVLESPNEGKTLNEKRLATLDIFAGCGGLSEGLQQSGVSSTKWAIEYEEPAGDAFKANHPEALVFINNCNVILRAVMEKCGDTDDCISTSEAAELAAKLDEKEISSLPMPGQVDFINGGPPCQGFSGMNRFNQSSWSKVQCEMILAFLSFADYFRPRYFLLENVRNFVSFNKGQTFRLTLASLLEMGYQVRFGILEAGAYGVSQSRKRAFIWAASPEDVLPEWPEPMHVFSAPELKITLSENVQYAAVRSTANGAPLRSITVQDTIGDLPAVGNGASKGNMEYQNDPVSWFQKKIRGDMVVLTDHISKEMNELNLIRCQKIPKRPGADWRDLPEEKIKLSTGQVVDLIPWCLPNTAKRHNQWKGLFGRLDWQGNFPTSITDPQPMGKVGMCFHPDQDRILTVRECARSQGFPDSYQFAGNIIHKHRQIGNAVPPPLASALGRKLKEAVDSKSST

>*Gm*MET2
MGSASLLNPSQPGVKKNSKSKQKSVVSKTEEKVMVKDKQKKRSLLESSEQPAATRKMPKRAAACKNLKEKSFLISEKSCLIEMEKDQIVEEESLAVRMTAGQDDGRPNRRITEFILHDATGKAQPLEVLEVNELFITGLILPLEVSTGKKKEKGVKCEGFGRVESWDISGYEDGSPVIWLSTDIADYDCQKPAASYKKVYDLFLEKARACIEVYLKLAKSSGGDPDISLDELLAGMVRSMSGSKCFSGTASIKDFVISHGEFIYKQLIGLDMTSKANDRTFADIPALIALRDESKKQANYVHAQVMPSNGSLRIDSGVGDEENKNQMDSVASVNEEDEDAKLARLLQEEEYWQSMKQKKNSRPTSVSNKYYIKINEDEIANDYPLPAYYKTSLQETDEFIVFDNDYDIYDTQDLPRSMLHNWSLYNSDARLVSLELLPMKPCSDIDVAIFGSGIMTSDDGSGFHLDTEAGQSSSVGSGAQVADGMPIYLSAIKEWMIEFGSAMIFISIRTDLAWYRLGKPAKQYAPWYDTVLKTARLAISIITLLKEQSRVSRLSFGDVIRKVSEFNQKDGSYISSDPLTVERYVVVHGQIILQLFAEFPDDNIRKSSFVTGLTNKMEERHHTKWLVKKKKVVPRSEPNLNPRAAVGPVVSKRKAMQATTTRLINRIWGEYYSNHLPEDSKEGIASELKDEDEVEEQEENEDDDNEEETILLEGTPKAHSASKQTKKISADTEIRWEGEPEGKTSSGYPVYKQAIIRGEVISVGRSVLVEVDETDEFPDIYYVEYMFESKIGRKMFHGRMMQCGCQTVLGNAANEREVFLTNECRDLGLHDVKQTVVVNIQNRPWGHQHRKDNIIADRVDRTQAEERKKKGLPTDYYCKSLYWPERGAFFTLPHDTLGLGSGVCPSCKIQDAEKEKDVFKVNSSKSGFLFNGTEYSLDDYVYVSPFEFEEKIEQGTHKSGRNVGLKAYVVCQVLEIVVKKEIKQAEIKSTQVKIRRFFRPEDLSNEKAYCSDIREVYYSDETYIISVESIEGKCQVRKKNDIPECSALGGIFQNVFFCELLYDPATGSLKKLPAHIKVKYSSGQTSDAAARKRKGKCIEGDGVSESTKEGKTLNDKRLATLDIFAGCGGLSEGLQQSGVSSTKWAIEYEEPAGDAFKANHPEALVFINNCNVILRAVMEKCGDTDDCISTSEAAELAAKLDEKEISSLPMPGQVDFINGGPPCQGFSGMNRFNQSSWSKVQCEMILAFLSFADYFRPRYFLLENVRNFVSFNKGQTFRLTLASLLEMGYQVRFGILEAGAFGVSQSRKRAFIWAASPEDVLPEWPEPVHVFSAPELKITLSENVQYAAVRSTANGAPLRAITVRDTIGDLPAVGNGASKGNMEYQNDPVSWFQKKIRGDMVVLTDHISKEMNELNLIRCQKIPKRPGADWRDLPEEKIKLSSGQVVDLIPWCLPNTAKRHNQWKGLFGRLDWQGNFPTSVTDPQPMGKVGMCFHPDQDRILTVRECARSQGFPDSYEFAGNIIHKHRQIGNAVPPPLASALGRKLKEAVDSKSST

>*Gm*DRM1
MQFHNYVGSEGDDSSPSDYDWNTDDELEVFGIPPSDPTISSQESCEDSVGESSASCSSAKHSKLIHLFVGMGFSRETVIKAIDENGRDNEEDIMEALLTLTAEKPLTVEKDEALSVLVNMGYPFEEALTAIDKCGPKAHISELADFISASQLEKGLHSPQESPNNKHDASDYTHEKPCQPSGEYYLHTSKKVKLGLGIFNEASQVISRKFPREVANKPYFYFENVALAPKGVWKTISRFLYEIEPEYVDSKYFCAATRKRGYIHNLPTHNRSPLLPIPPLTIQEAFPTTKKWWPSWDRRTKLNCLLTRVAPGPVTERIRKLLEKFGDEPPLHVQENVLVEIRKWNLVWVGKNKLAPLEPDEYEMLLGFPRDHTRGGGVTRTERYKSLGNAFQVNTVAYHLSVLKGRFPNGINVLSLFSGIGGAEVALHRLGMMLKNVVSVEIAEVNRNIIRSWWEQTNQRGNLIEVEDVQKVSSNELSQWITKFGGFDLIIGGSPCNNISGSNRVSRHGLEGEQSSLFYEYFRIVEAVMEIQRDELL

>*Gm*DRM2
MGGDDSGLESDNFDWNTEDELEIQNYNSSSSCLTLPNGDAVTGSGEASSSAVLANSKVLDHFVSMGFSREMVSKVIQEYGEENEDKLLEELLTYKALESSSRPQQRIEPDPCSSENAGSSWDDFSDTDIFSDDEEIAKTMSENDDTLRSLVKMGYKQVEALIAIERLGPNASLEELVDFIGVAQMAKAEDALLPPQEKLQYNDYAKSNKRRLYDYEVLGRKKPRGCEKKILNEDDEDAEALHLPNPMIGFGVPTESSFITHRRLPEDAIGPPYFYYENVALAPKGVWQTISRFLYDVEPEFVDSKFFCAAARKRGYIHNLPIQNRFPLLPLPPRTIHEAFPLTKKWWPSWDIRTKLNCLQTCIGSAKLTERIRKAVEIYDEDPPESVQKYVLHQCRKWNLVWVGRNKVAPLEPDEVETLLGFPRNHTRGGGISRTDRYKSLGNSFQVDTVAYHLSVLKEMYPNGINLLSLFSGIGGAEVALHRLGIPLKNVVSVEKSEVNRNIVRSWWEQTNQKGNLYDMDDVRELDGDRLEQLMSTFGGFDLIVGGSPCNNLAGSNRVSRDGLEGKESSLFFDYFRILDLVKNMSAKYR

>*Gm*DRM3

MAGNPNRREGKTVMVPKTENLDYELPPYTSFSGDVGDNVASSSGGKLRAFFIGMGFLPCLVDKVIEENGEENSDILLEALLRYSALQKSNSQSSVSLDSLFDDKDPPEISNVNQAKEEPDELSGVVDDTRGSLLMMNFSVEEVEFAIHKLGDEASIPELVDFIFALQIAKKLKKEPDDITFTYYGRGNEVTNEKLFGIMAKTLQLFEMGFSENEVSSAIDKLGSEAPISELANFIFAEQNGIDYVMEYKFPTTSTYSVGIKEEPEMDLYGTAEVKVEGFSHEPPQSSQVNLEETYNDDMVKEEEGIDAFPSNVSDQYLDVVENGRGKRPKYEHDDDPVNCLEPSWVEERVDAVVAEMSRHPKPNPSRCLSSVAAKPPFFLFGNVSNISYDSWTKMSQFLYGIEPEFANAQSFSAMDRIEGYIHNLPVENRFHILPKPPMTIEDAMPQTKKWWPPWDSRKLLSSIYCETNGIAQTCDRLGNFLADSGGVLTSEQQKDILRYCRRLNLVWIGKFKLGPVEPEQLELILGYPLNHTRATEGNVAERLKSLKYCFQTDTLGYHLSVLRPIFPHGLTMLSLFSGLGGAEIALHRLAIKIKAVVSVETSETKRKILEKWWRQSGQTGTLVQIEDIQKLTSKKLEGLISKFGGFDLVIYQNPCSYSSSRLQAGVGLSALDFSVFCECVRVLQRVRGMYQRK

>*Gm*DRM4
MAGNSNRREGKPVMVPKTEDLGYELPPYTSFSGDVGDNVASSSGGKLRAFFIGMGFLPCLVDKVIEENGEENSDTLLEALLRYSAHKSNCDSFDSLGVSHNTSRGRSAPNFYPDGHSKEALQKSNSQSSDSLDSLFDDKDPPEISNVNQAKEEPDELSGVIDDKRGSLLMMNFSVEEVELAIHKLGDEASIPELVDFIFAWQIAKKLKKEPDDITFTYYGRGNEVTNEKLFGIMAKTLQLFEMGFSENEVSSAIDKLGSEAPISELANFIFAEQNGIDYVMEYKFPTTSTYSVGIKEEPEMDLYGTAEVKVEDFSNEPPQSSQVNLEETYNEDMVKEEEGIDAFPSYVSDQYLDVVENGRGKRPKYEHDDDSITCLEPSWVEERVDVVVAEMSRRPKPNPSRCLSSVAAKPPFFLFGNVSNISYDSWTKMSKFLYGIEPEFANAQSFSALDRIEGYIHNLPVENRFHILPKPPMTIEDAMPLTKKWWPPWDSRKQLSSIYCETNGIAQTCDRLGNILADSGGVLTSELQKDILRYCRGLNLVWIGKFKLGPVEPEQLELILGYPLNHTRASEGNVAERLKSLKYCFQTDTLGYHLSVLRPIFPHGLTMLSLFSGLGGAEIALHRLGIKIKVVVSVETSETKRKILERWWRQSGQTGTLVQLEDIQKLTSKKLEGLISKFGGFDLVIYQNPCSNLSSRLLAGVGLPALEFSVFCECVRVLQRIRGMYQRK

>*Gm*DRM5
MGGDDFDWNTDDELEIENYNSSSSCLTLPNGDAGEASSSAVLANSKVLDHFVNMGFSREMVSKVIQEYGEENEDKLLEELLSYKALESSPQPQQRIEPDPCSSENAGSSWDDFSDTDIFSDDEEIAKTMSENDDTLRSLVKMGYKQEEALVAIERLGPNASLEELVDFIGVAQMAKAEDALLPPEEKLQYNDYAKSNKRRFYDYEVLGRKKPRGCEKKILNEDDDEEDEALHLPNPMIGFGVPTESSFITHRRIPEDAIGPPYFYYENVALAPKGVWQTISRFLYDVQPEFVDSKFFCAAARKRGYIHNLPIQNRFPLLPLPPRTIHEAFPLTKKWWPSWDTRTKLNCLQTCIGSAKLTERIRKAVEIYDEDPPESVQKFVLHQCRKWNLVWVGRNKVAPLEPDEVETLLGFPRNHTRGGGISRTDRYKSLGNSFQVDTVAYHLSVLKEMYPNGINLLSLFSGIGGAEVALHRLGIPLKNVVSVEKSEVNRNIVRSWWEQTNQKGNLYDIDDVRELDGDRLEQLMSTFGGFDLIVGGSPCNNLAGSNRVSRDGLEGKESSLFFDYFRILDLVKNMSAKYR

>*Os*MET1

MDKCGDSDDCISTSEAAERAAKLSEDKIKNLPVPGEVEFINGGPPCQGFSGMNRFNQSPWSKVQCEMILAFLSFAEYFRPRFFLLENVRNFVSFNKGQTFRLTLASLLEMGYQVRFGILEAGAYGVAQSRKRAFIWAAAPGETLPEWPEPMHVFASPELKITLPDGKFYAAVKSTAAGAPFRSITVRDTIGDLPAVENGAGKPTIQYGSGPVSWFQKKIRSDMASLNDHISKEMNELNLIRCKHIPKRPGCDWHDLPDEKVKLSTGQMVDLIPWCLPNTAKRHNQWKGLYGRLDWEGNFPTSVTDPQPMGKVGMCFHPEQDRIITVRECARSQGFPDSYRFAGNIQNKHRQIGNAVPPPLAYALGRKLKQAIDAKR

>*Os*MET2

MDKCGDADDCISTSEAAEQAAKFSQDNIMNLPVPGEVEFINGGPPCQGFSGMNRFNQSPWSKVQCEMILAFLSFAEYFRPRFFLLENVRNFVSFNKGQTFRLTVASLLEMGYQVRFGILEAGTFGVAQSRKRAFIWAAAPGETLPDWPEPMHVFASPELKINLPDGKYYAAAKSTAGGAPFRAITVRDTIGDLPKVENGASKLLLEYGGEPISWFQKKIRGNTIALNDHISKEMNELNLIRCQRIPKRPGCDWHDLPDEKVKLSSGQLVDLIPWCLPNTAKRHNQWKGLYGRLDWEGNFPTSVTDPQPMGKVGMCFHPDQDRIITVRECARSQGFPDNYQFAGNIQSKHRQIGNAVPPPLAFALGRKLKEAVDAKRQ

>*Os*CMT1

MVPEPAPAAATEPRRSTRRRLMTAAAMEAEAEAVADLDEIDREMSRAESRKRQRRTAKEKPGARKGATEWKPEDVEKAAAAEGVAELDEIDREMPRPELRKRQRRTAKEKPSAHEGATEWKPEDVEKAAAQEPEGTELDSGLSPAESRGKRQRGVEKVKRRTRKKTAKEKTKETTEKSAAQAPEKMKVNDAGGALAEDVCADEPDAEQMAMEEEEEAADVLEAEERMGKCVGEGSAEKAATRKRVARPSTARRVEDSDDHFVGDPVPDDEARQRWPVRYSRKGSDSLLKQEPDEDEEMKARCHYLAANVDDEIYHLDDDVYVKAGPDEENYIGRITEFFEGVDRGSYFSCQWFFRTADTVISSKLLKVHDHRHNHKRVFLSKEKNDNLIECIVSKVKIAHVDPNMTPQARAHAISDCDLYYDMSYSVAYSTFANLPADNDGALGSEATSNISCDDADNSSKGKLSADIVAPYSEQTETASLLDLYSGCGAMSTGLCLGFAFSGINLETRWAVDINKYACACLKHNHPYSQVRNEKTEDFLALIQQWDALCRKYVVHKNDTLEPSIDMPLNDADDVNEPLPEDIFDVEELLEICYGDPSNTGKNGLWFKVRWKGYDPSYDTWEPIDGLSDCPERIKEFVEKGHKENILPLPGAVDVICGGPPCQGISGFNRFRKHNDPLEDEKNKQLVVFMDIVKYLRPKYVLMENVVDILKFADGFLGRYAMSCLVAMNYQARLGMMAAGYYGLPQFRMRAFLWGALPSMVLPKFPLPTHDAVVRGIVPTTFSQSVVAYNEVDTRCLRKALLLADAISDLPKVGNDQPKDVIEYSVAPKTEFQRYIRNNRKDIQDYSFRGDDPSEEGKLFDHQPLKLNKDDYERVQRIPVKKGANFRDLKGVIVGPDNTVRLDPNISRERLSSGKPLVPDYAISFVKGKSTKPFGRLWWDETVPTVVTRAEPHNQIILHPSQDRVLTIRENARLQGFPDYYRLIGPLKEKYIQVGNAVAIPVARALGYALGLAYRGESDGDRAVLKLPESFIYADQETVVKSSAGTPGSEIADSEQLFEYFINPNFFCMMFAFLKVNVVIPLILDSKMGLSRRGAEEGEARRAVPFQNILSWEGWNRLDHRRGEIRLDVRREMDDSPLDNLFDGNGLDSSAGADSSSSTGSPPSSTSSSPPSSQSPPPGSSPPPASPPPSTPSAPPTNSSGSAPSPPSPSQSAPPANTGGGGSPPPSHGSPPAPKAVQSQPAPKRSGDGGSSSDSGSSKEGGSSSDRGKSESNGNRPGPEAAIIAGMVIGFFTFALLLAIVACVCCSKKKKRPPHMHMPYYTDENGKVYYANSMPRWQNSVDQGGGWHAQYSPGQAPPSSEMSGSHGAGPLPPPSPGMALGFSKSSFSYDELALATGGFSSANLLGQGGFGYVYRGVLAGSGKEVAVKQLKAGSGQGEREFQAEVEIISRVHHRHLVSLVGYCIAGSSQRLLVYEFVPNDTLEHHLHGLHIFPALFPAIVTRHSEGRAGDGLDDEARHRARLSQGPRVPSRRFLQGQILSVRPSNDLICCIVVVTYEGHPRIIHRDIKAANILLDENFDAKVADFGLAKLTTDTNTHVSTRVMGTFGYLAPEYASSGKLTDKSDVFSFGVMLLELITGRRPVDPTNYMEDSLVDWARPLLARALSEDGSFDELIDQRLENKFDRLEMERMAACAAAAVRHSAKRRPKMKQIVRALEGDASLDDLNEGVKPGQSMMFSTGSEYDSGNYASDINRLRKVAFESSIEDSSEYGTHSSADSGEPPRRQQHR

>*Os*CMT2

MEASVSQIRRSPPRIEKRKKKNHNSLSGKAPPTRGNCDSLPPVWNAKSASECHWTRRLTSNYLSLVVVVVGEELPPCFAPPPMETPPPDPVSPPPPAADEGSPGGDDGAEDAGGFSAGLDSLWTALFGSPEELEPMWSPPRGFGVGAEFAAAEVEPEIMDVAGGPWDGAPWRSSGVVAGEGAATALVPPTAAAGFAEFEPAAPIDSYPAGAAAASLGDVPEVSALDSGVDCSPDPPPSSSPPVDFDARGFDPVADSAPAMESPLPPSVASSEANLDGRMLDCTLNSVPSPPLASPYEVGLGAEDPIKDSSPSVAWGTTMDAKDPEVDATCANGTALRRSRRIMKIKSAASSMPLNQNGDSSRASKRRVADSRKSRSSEGSKLPAFTGPISVNTVDLINGVKVQGLQEIVAVENVSSSYDNNQKAGGLYNQVVVALPAASNSLLKDKGASVLPRRKTRLASKVLVNSDRVSAISPVVNGGPPVQKSDVCIPTKKHKLAVEECLTSLDGVDGGGIVLCNSKLKSAKSRVVSKTPQGRGRRSPQPPKTQRARTLSVKYLEKLKRAENNNNNGSMSKSPRVPMIPENNGSMSKSPRVPIIPELSTKHELVLDKHMVDSVMLETDDGSCFFVGDAVPDDEARKQWPHRYEINDQIMKKDKRTSSQTFAKLVTVSFCVVFMYVYLQMLDFRGPEGKPNYIGRLLEFFETKTGECYFRVQWFFTAEDTVIGEQAQSHDPRRLFYSDLTDDNLLDCIVSKVTIVQVPPSVDGKSKSVPSSDYYYDMKYSIDYSTFSTIEMEDTDDLMQSCYTSRINDKMKKIDVNKKHKSPVLEKMELSLLDLYCGCGGMSTGLCLGARGGGVNLSARWAIDDDEIACESFRNNHPETRVRNETTDDFLELLKEWEKLCKTYVKHSRTKACVDSTTESNNETPDCSTVPPEEFEVWKLVDICFGDPNKVSKHGLYFKVRWKGYGPHHDTWEPVEGLRNCKEAIRDFVIEGHRQRILPRPRRNIAVFLLRPSKFPLGDVDVVCGGPPCQGISGYNRNREFEAPFKCEKNKQIIVFMDVVQFLKPKYVYMENVLDILKFADATLARYALSRLVAMHYQARLGIMAAGCYGLPQFRMRVFLLGCHSKEKLPPFPLPTHEAIVKNGCPLAFERNLVGWPNDTPMQLARPIVLEDILSDLPEVANGESRDEMLYVKGPQTEFQRYIRSFNVEVHGPRAHVTKDSKSSKLYDHRPLVLDNDNYQRILQIPKRKGANFRDLSGVIVGPDNVARLDPTKERVLLPSGRPLVLDCILAYENGKSLRPFGRVWWDEVVGTVLTVPNARMQALIHPAQDRLLTIRESARLQGFPDNYRFRGTVKDRYRQIGNAVAVPVGRALGYALAMAYLKKSGDDPLMLLPPNFAFSHDLRGFA

>*Os*DRM1a

MRIASSSGILMDANGKANGSAPSALVAYFLGMGFSREMVFRAIKEIGDTDSEQILELLLTYQAIGSDPSVGNSSHSACDPQILEEEDEEEDVNWDEDDTVDNFDRATYSDGSGDEDFLQEMSEKDEKIKSLVSMGFPEDEDTEFSSFGGRKKTKLIDGSKKKRERYRSRPQWNQVPFDGSHEEPMPLPNSMVGFSLPNDGLRSVHRNLPDQALGPPFFYYENVALAPKGVWTTISRFLYDIYPEFVYSKYFCAAARKRGYIHNLPIKNRNYTRGVSRTARYRALGNSFQVDTVAYHLSVLRDIFPNGMNVLSLFSGIGGAEVALHRLGICMKTVVLVEISEVNMTLLRSWWDQTQTGTLIEIADVQNLTAERIELFIRRFGGFDLVIGGSPCNNLAGSNRYHRDGLEGKHSALFYHYYRILDSVKTIMASIFGAKGKLFRHVRKALLLKQSSSLTLKTEQDPSNNSDKDSMDK

>*Os*DRM2

MQAIGGDASVGNCSASACAPQTLEVDEEEDDTNWDEYDTAGNCDRTPHSDGSGDEDFFQEMSEKDEKMKSLVNMGFPEDEAKMAIDRCLDAPVAVLVDSIYASQEAGNGYSANLSDYEDTEFSSFGGRKKTRFVDGSKKRKRYGSGPSGNQVPFDGSHEEPMPLPNPMVGFSLPNERLRSVHRNLPDQALGPPFFYYENVALAPKGVWTTISRFLYDIQPEFVDSKYFCAAARKRGYIHNLPIENRSPVLPMPPKTISEAFPNTKRWWPSWDPRRQFNCLQTCMASAKLTERIRCALGRFSDVPTPQVQKYVLDECRKWNLVWVGKNKVAPLEPDEMEFLLGYPRNHTRGVSRTERYRALGNSFQVDTVAYHLSVLRDLFPNGMNVLSLFSGIGGAEVALHRLGIHMKTVISVEKSEVNRTILKSWWDQTQTGTLIEIADVRHLTTERIETFIRRFGGFDLVIGGSPCNNLAGSNRHHRDGLEGEHSALFYDYIRILEHVKATMSAV

>*Sl*MET1

MASPQPNSESVLELPNNDKSGHKKNKRKQDSVSKRKASATGKKEKKQAVSETIEEPTAGRKRPKRAAACSDFKEKSVHLSKKSSVIETKKDHCVDEEDVAIRLTAGLQESQRPCRRLTDFVFHNSEGIPQPFGMSEVDDLFISGLILPLEDSLDKVKAKGIRCEGFGRIEEWAISGYEDGTPVIWISTETADYDCLKPSGSYKKFYDHFLAKATACVEVYKKLSKSSGGNPDLSLDELLAGVVRAMTGIKCFSGGVSIRDFVITQGGFIYKELIGLDDTSKKTDQLFVELPVLASLRDESSKHETLAQPETISSGNGLRIGPKAGNGGDKIVESGLANGPAPEDEDLKLAKLLHEEEYWCSLKQKKDRNTSSSSSKIYIKINEDEIASDYPLPAYYKTSNEETDEYIVFDSGVETYHIDELPRSMLHNWALYNSDSRLISLELLPMKACADIDVTIFGSGVMTADDGSGYNFDTDANHSSSGGSRSAEIDGMPIYLSAIKEWMIEFGSSMIFISIRTDMAWYRLGKPLKQYAPWYEPVIKTARLAVSIITLLKEQNRVARLSFGEVIKRVSEFKKDHPAYISSNVDAVERYVVVHGQIILQQFSEFPDVSIRNCAFAVGLSRKMEERHHTKWVIKKKKVMQRLEQNLNPRASMAPSVKRKAMQATTTRLINRIWGEYYSNYSPEVSKEVADCEVKDDEEPDEQEENEEDDVPEENLDVPEKAHTPSSTRRHIKSRSDSKEINWDGESIGKTASGEQLFKKARVHGHEIAVGDSVLVEHDEPDELPSIYFVEYMFEKLDGSKMLHGKMMQRGSDTVLGNAANEREVFLINECMNLQLGDVKESIAVNIRMMPWGHQHRNTNADKLERAKAEDRKRKGLPTEFYCKSFYRPEKGAFFRLPFDKMGLGNGLCYSCELQQTDQEKESFKFDMSKSSFVYLGTEYSVDDFVYVSPDHFTAERGGNGTFKAGRNVGLMAYVVCQLLEIVGPKGSKQAKVDSTNVKVRRFFRPEDISSDKAYSSDIREIYYSEDIHTVPVEIIKGKCEVRKKYDISSEDVPAMFDHIFFCEYLYDPLNGSLKKLPAQINLRLSKIKLDDATSRKRKGKGKEGVDEVGELNETSPQNRLSTLDIFAGCGGLSEGLQHSGVTDTNWAIEYEAPAGDAFRLNHPKTKVFIHNCNVILRAVMQKCGDSDDCISTPEASELAAAMDESELNSLPLPGQVDFINGGPPCQGFSGMNRFNQSTWSKVQCEMILAFLSFADYYRPKFFLLENVRNFVSFNQKQTFRLTVASLLEMGYQVRFGILEAGAYGVPQSRKRAFIWAASPEEVLPEWPEPMHVFAVPELKIALSETSYYAAVRSTASGAPFRSLTVRDTIGDLPVVGNGASKTCIEYQGDPVSWFQKKIRGSSITLSDHISKEMNELNLIRCQRIPKRPGADWRDLEDEKVKLSNGQLVDLIPWCLPNTAKRHNQWKGLFGRLDWDGNFPTSITDPQPMGKVGMCFHPDQDRIVTVRECARSQGFPDSYQFAGNILHKHRQIGNAVPPPLAYALGRKLKEAVESKNRLT

>*Sl*DRM7

MDNNFSGEDNDSIDWDTEDELEIQEMPDATFSSCTNLRSVGYHTVSGHREARSSSEPCQSKFIQQFIVMGFPEESIAKAIEQNGENEGLVLDALLTFKALEDSPEEQPSTSTQMEPCITSDDSSSQYNENFLDDVSEDDSWSLDSDNCVNSAKQSYLNDDNCSLSENEKTLLFLANMGYPAEEASIAMERCGPEAPFPELIDFMCAAQMAREEDVHLPEDEKPKLNSGGYKRKMYNEVRVKKKQRAITDEETIHLPRPMIGFGVPTESLPAVVKRTLPEQAIGPPFFYYENVALAPKGVWDTMTRFLYDIEPEFVDSKYFCATARKRGYIHNLPIEDRFPLLPLPPRTIHEAFPLTKKWWPSWDTRTKLNCLQTSIGSARLADRIRKAMKAMENFDSEPPLMVQKYVLDECRKWNLLWVGRNKVSPLETDEFEMLMGFPRNHTRGGGISRTDRYKSLGNSFQVDTVAYHLSVLKDLYPNGINVLSLFSGIGGAEVALYRLGIPLNNVVAVEISEVNRNILRSWWEQTNQKGNLIDFHDIQQLNGDVLEQMIDSIGGFDLVIGGSPCNNLTGSNRVTRDGLEGKDSSLFYDYVRVVDLVKSIMSNRRV

>*Sl*DRM6

MDKNLSGEDNDDIDWDTEDELEIQEIQDTVFSSCTDLRTTGQHVVCCDVEASSSSVPFRSKFIQQFVVMGFPEESIAKAIEQNGENSDLVLDSLLTFKALDDSPEEQPSVSPPLEPSISSDDSASEYNKIVLDNVYEDDSWSSDSDNYINTVKQCYLNDEGSSLSEKEKMLLFLGNMGYPAEEASIAMDRCGPKASLPELVDFICAAQMSRAEDPYLLEDVKPNLKDILNDCGGYKKRKMYNELCKRKKQREISVEEPIRLPKPMIGFGIPTESVPRMVQRILPEKIIGPPYFYYENVALAPKGVWDTIKRHLYEIEPEFVDSKYFSATARKRGYLHNLPIENRFPLFPLPPRTIHEALPLSKRWWPSWDTRSKLNCLQTAIGSARLTDKIRKAVEKYDGEPPMEIQKYVLYHCKKWNLVWVGRNKVAPLEPDEVEMLLGFPKNHTRGGGISRTDRYKSLGNSFQVDTVAYHLSVLRDLFPNGINVLSLFSGIGGAEVALYRLGVPLNNVVSVEKSEVNRNIVRSWWEQTNQRGNLIHFDDVQLLSRDRLKKLIESVGGFDLVIGGSPCNNLAGSNRVSRDGLEGKESSLFFDYVRILDDVKSIMSRHR

>*Sl*DRM5

MDKHLSEEDSDNIDWDTEDELEIQDTTFSSCRDLRTNGQYAISGDGEASSSSVPGQSTFIQKFLVMGFSEESIAKAIEQNGENSDLVLDALLTLKAIEDSPEEQPSASPHLEPCINSDDSSSEYNENFLDDVYDEDSWSSDSDYCTNSVKQCYVKEESNSLSEKEQTILFLANMGYPVEEVSIAMERCGPEASVSELTDFICAAQMAREEDPYLPEDVKPKLNHGSGGYKKRKMFNQLCKSKKPRAIFDEETIRLPKPMIGFGVPTESVSAIVRRTIPEQAFGPPFFYYENVALAPKGVWDTISRFLYDIEPEFVDSKYFCATARKRGYIHNLPTENRFPLLPLPPRTINEALPLTKKWWPSWDPRTKLNCLQTAIGSARLTDRIRKAVEAFDGEPPMRVQKFVLDQCRKWNLVWVGRNKVAPLEPDEFEMLLGFPKNHTRGGGISRTDRYKSLGNSFQVDTVAYHLSVLKDMFPNGMNVLSLFSGIGGAEVALYRLGIQLNNVVSVEKSEVNRNIVRSWWEQTNQRGNLIDFDDVQQLNGDRLEQLIDSCGGFDLLIGGSPCNNLAGSNRVSRDGLEGKESSLFYDYVRILDLVKSIMSRQR

>*Sl*CMT4

MAKKQNDSSRFSTNLTPSPQKDKPISSSSELALFYPQNEEPVPLIVFYPSSVRRRSSRFTTNKFSTSATEFMTKNRDTISPEKTVFLLPSPPTLAGKKTPAEVTRRSPRLVSLSARTTTAKEKGKKVNSRKSEGGIKQVELSRKRKPASCKMDEETRKSPRFNSDSSNGVQLALPEMSACGALSAGGRTGTKRELLALMTTTPANSSASRKRAARGSDSVNVGNNSRGSRRKDPVFAESPGTSVKITAESNSAGEKNLRSRKAQGSVNYNESKGSETKRIKSSAEKSVRKQKSNACFIGEPIDTEEAQQQWQWRYELKNRKTQRQGWKLNSGEEDEIILNVECHYAQAKVAGFIFNIGDCAFVKGEGKKKHIGRILEFFKTTEGEDYFRVQWFFRAEDTVLQGAASFHDPKRVFYSTLENDNLLDCIVSKVNVVELPTRHDLNKKDVPPAHFYYDMEYCVDYSTFRTLHNVKSSVSPSLVDVSYKPITTYPLEVSPSCEPMKVELSLLDLYAGCGGMSMGLCLGTKLSGLNLVTKWAVDFNKAACDSLKLNHPQTHVRNEGVENFLELLKRWEKLIKSYGCSDIKTSSNIELDDRDEGENNDDSQSGSNASSGEYEVLRFVDICYGDPNNDGKSGLHFKVRWKGYGPSEDTWEPIENLKNCGDSIKDFVRRGQQLKILPLPGDVDMICGGPPCQGISGYNRHRNTDDPLSDEKNRQIIIFMDVVEFLRPKYVLMENVADILRFDKASLGRYALSRLVHMRYQARLGTMAAGCYGLPQFRLRVFFWGALPSERLPPFPLPSHDVIVKYWPSPEFERNTVAYEEGQPRDLEEALVLRDAISDMPAVTWHETREERPYEMPPETEFQKYIRLSKHEILSCTSTGVKETKEPVLSDHRPCQLNEDDYLRVCLVPRRKGANFRDLPGVIVGGDNVARRDTKDPKVLPNGKPMVPDCAFNFEHGKSKRPFARLWWDETVATLVTFPNHRAQAILHPEQDRVLTIREYARLQGFPDFYRFTGTLKERYCQVGNAVAVPVGRALGYALGLAYQRLAGNEPLIKLPSNFSFLTPPIDDIVVLQT

>*Sl*CMT3

MSSKRKASPADSSSDSSKRHALEVVKTVDIASDEVAEGFRDDDEFVEDRDIVCDSSIGESSGQKEVRRVAVRANEEQEGEFYGEIVLDSEARKKWPHRYILKDNVNINSASMSLNCQHDSDELIQAKCHFAQALVDNVIYKLGDDAYVKAAEDEDDYICKIVEFFQGVDDMKYFTAQWFYRAKDTVIKAHDQFIDKKRVFLSDIKDHNPLDCLVKKIKIVPISSNVSLQFKESLRLECDYYYDMKYLVPFSSFISLPSDVLSPDSESNSTISSDGDVVEVKEQKQEKKLLDLYSGCGGMSTGLCLGADVCDVKLVTKPSNTPTHPLLKVGDANVEDDDEGADDDDGGSGDEDEGEIFEVEEILEVCYGDPKEIKKPGLYFKVRWKGYGPDEDTWEPIEGLDGCQNKIKDFVTDGFKRSVLPLPGQVDVVCGGPPCQGISGFNRFRNSANPLQDPKNKQLEVFMSIVEFLKPRFVLMENVVDLLRFAHGYLGRYALSRLVGMNYQARMGMMVAGAYGLPQFRMRVFMWGALPSEKLPQYPLPTHNVIVRGGIPTEFELNAVDFEEGLKVKLKRELLLEDALSDLPPVENNEPRDEMPYIDEPKSVFQRFIRSRRDGTLGTVLYDHRPLQLNEDDYQRVTQIPKQKGANFRDLPGVRVRADNVVEWDPDMERVKLPSGKPLVPDYAMTFVRGTSQKPFGRLWWDEIVSTVVTRAEPHNQAILHPVQDRVLTIRENARLQGFPDYYKLTGPIKERYIQVGNAVAVPVARALGYSLALALKGLSRDQPLLTLPPNFPCLEELVSNDESLDKV

>*Sl*CMT2

MPSKRKSSPATKPESSSGSRKSKRLVVERPDPVVAQPSDSDFEPEPVLSSKKKSTRRTTAESSVVACQSESNNKKLKKPTVEKAESGVASPADRDFVSESDSETPSKKSTRRAAVKVEPLVDSVAGSDFVEEEEVDGMELGSLKKSLSISPSKRKPKRAEKVKDEECVLAGDPVPDAEARLKWPHRYNKGKENGTKSLNGQDDPDQLIQAKCHFSRADVDGQIYYLEDDAHVKAADGEDDYICKIVEFFEAVDGVQYFTAQWFYRAKDTVIKSHDQFIDKKRVFLSEIKDDNPLDCLVTKLKIVPVPSNATSQFKENVKSNCDFYYDMKYLLPYSSFISLPPDTTSPVSSSSTISSDIDAGEVKEHNLEKKLLDLYSGCGAMSTGLCLGANSKGVKLVTKWAVDLNKHACDSLRLNHPETQVRNEYASDFLSLLKEWVQLCVSCSLIKGSVPPHPHLKVTDEVDEDEENDDEGEDSGDDKEGEIFEVEELLEVCYGDPKENNKPGLYFKVRWRGYGPEEDTWEPIDGLSDCPKKISEFVVKGFKANLLPLPGDVDVVCGGPPCQGISGFNRFRNKENPMQDPKNKQLDVYMDIVDFLKPRFVLMENVVDLVKFSNGFLGRYALSRLVGMNYQARMGMMAAGAYGLPQFRMRVFMFGALSSEKLPQYPLPTHKVIVRGVIPVEFESNTVAYDSVRDLELKKELFLGDALSDLPLVENNEPRDEMPYTDEPKSDFQHFIRMGRDGLLGSVLYDHRPLQLNEDDHQRVCQIPKRKGANFRDLPGVRVRPDNKVEWDPDVERVKLPSGKPLVPDYAMSFVGGSSSKPFGRLWWDETVPTVVTRAEPHNQTIVHPLQDRVLTIRENARLQGFPDYYKLIGPIKERYMQVGNAVAVPVARALGYSLAMSIKGLSGETPLFTLPKNFPSHEDQNCNEVSQ

>*Fa*MET1a

MGSIAAADDAAAAAAATAPLNDAESAPSALSSGTTKKKGRKATQKEAPAGGRGRKRNVPPTGEEQSQSRKMPKRAAACKDFKERSVRVSEKSAFIESKEEQVVPDESLAVVMTCGKDPDDEKPNRRLTQFILHNEDGVAQPLEMVQHGDLFITGTLLPLDASSDNGKGKGKEKGIICEGFGRIEAWDISGYDEGHPIIWLTTDVADYCCVKPASSYKKHYDNFCEKARACIEVYKVLSKCNSDCSLDELLAGIARSMSGNKFFSGSASIKEFIISQGVFIYNQLEGLEESSKKNDRVLAQLPVLAALRQECIKQGYYVTSNPAASSGTLKIASDGGNSSSKVETEEDEDAKLARLIQEEEYFKSMKQKKRQGVTSMSKKFYIKINEDEIANDYPEPAYYKTAIEETDEFIVVDSEDGDLPTHMLHNWCLYNSDSRLISLELLPMKACADIDVTIFGSGVMSEDDGSCGFDLDSIQSSSSGSVAPVAEDAYGMPVYLSSIKEWVIEWGASMIPVSIRTELAWYRLGKPSKQYAKWYAPILKTARVGRSIITMLKAESRVARLSFADVIKRLSEFPNSNGGYISSDPASVERYVVVHGPILLQLFTEFPDGKIKTCPFVHGLRDKMEKRHHTKWEVNKKILQKSESNLNPRASMGPVVSKRKAMQATTTKLINRIWGEYYSNYSPENSSLVETCEKIEEDEADEEVQEDLEEDDAEENSSVVKEAQRPSPILRKIKSSSSSKKGFVDEEPVGKTSSGEALYKHAVLRGEEISVGGAVMVEVDGSDELPVIYFVEYMYESSDGEKMLHGRMMQRGSDTVLGNTANEREVFLTNECINLALKDVKQTVVVGIKSMPWGHKHQKENADAERIDRANAEERKKKGLPTEYYCKSLYCPEKGAFLSLSCDTIGLGSGFCFSCKANEEEKAKEVFEVNSSKTGFIYSGVEYSVHDYVYVSPYHFSVQTIETELFKAGRNLGLKPFVVCQVLGIITKGSKQSEMKSTQVKVRRFFRPEDISVEKAYCSDVREVYYSEELHILPVDSIEGKCEVRRRSDLPECNAPALFQHIFFCEHLYDPSNGSLKQLPANIRVKYSTVGGDTESRKRKGKCKEGEDVPEVEQQRVDSEHMRLATLDIFAGCGGLSEGLHQAGVSITKWAIEYEEPAGQAFQLNHPESKVFINNCNVILKAVMDKCGDTDDCISTTDANDLASALDEKEKSDLPLPGQVDFINGGPPCQGFSGMNRFNTSTWSKVQCEMILAFLSFADYFRPKYFLLENVRNFVAFNKGQTFRLTLASLLEMGYQVRFGILEAGAYGVSQSRKRAFIWAAAPDEILPEWPEPMHVFGVPELKINLSSNSYYAAVRSTASGAPFRPITVRDTIGDLPAVGNGASKVNMEYESDPVSWFQKKIRGNMAVLTDHISKEMNELNLIRCKRIPKRPGADWKDLPEEKVKLSTGQLVDLIPWCLPNTAKRHNQWKGLFGRLDWEGNFPTSITDPQPMGKVGMCFHPDQDRIITVRECARSQGFPDSYQFYGNTLHKHRQIGNAVPPTLAYALGRKLKEAIDSKKRSSSQE

>*Fa*MET1b

MGSIAAADEAATAPLNDAESAPSAPSSGTTKKKGTQKEAPAGGRGRKRNAPPTGEGQSQSRKMPKRAAACKDFKERSVRVSEKSAFIESKEEQVVPDESLAVVMTCGKDPDDEKPNRRLTQFILHNEDGVVHPLEMVQHGDLFITGTLLPLDASSDNGKGKGKEKGVICEGFGRIEAWDISGYDEGNPIIWLTTDVADYCCVKPASSYKKHYDNFCEKARACIEVYKVLSKCNSDCSLDELLAGIARSMSGNKFFSGSASIKEFIISQGVFIYNQLEGLEESSKKNDRVLAQLPVLAALRQECIKQGYYVTSNPAASSGTLKIASDGGNSSSKVETEEDEDAKLARLIQEEEYFKSMKQKKRQGVTSMSKKFYIKINEDEIANDYPEPAYYKTAIEETDEFIVVDSEDGDLPTHMLHNWCLYNSDSRLISLELLPMKACADIDVTIFGSGVMSEDDGSCGFDLDSIQSSSSGSVAPVAEDAYGMPVYLSSIKEWVIEWGASMISVSIRTELAWYRLGKPSKQYAKWYAPILKTARVGRSIITMLKAESRVARLSFADAIKRLSEFPNSNGGYISSDPASVERYVVVHGPILLQLFTEFPDGKIKTCPFVHGLRDKMEKRHHTKWEVNKKILQKSESNLNPRASMGPVVSKRKAMQATTTKLINRIWGEYYSNYSPENSSLVETCEKIEEDEADEEVQEDLEEDDAEENSSVVKEAQRPSPILRKIKSSSSSKKGFVDEEPVGKTSSGEALYKHAVLRGEEISVGGAVMVEVDGSDELPVIYFVEYMYESSDGEKMLHGRMMQRGSDTVLGNTANEREVFLTNECINLALKDVKQTVVVGIKSMPWGHKHQKENADAERIDRANAEERKKKGLPTEYYCKSLYCPEKGAFLSLSCDTIGLGSGFCFSCKANEEEKAKEVFEVNSSKTGFIYSGVEYSVHDYVYVSPYHFSVQTIETELFKAGRNLGLKPFVVCQVLGIITKGSKQSEMKSTQVKVRRFFRPEDISVEKAYCSDVREVYYSEELHILPVDSIEGKCEVRRRSDLPECNAPALFQHIFFCEHLYDPSNGSLKQLPANIRVKYSTVGGDTESRKRKGKCKEGEDVPEVEQQRVDSEHMRLATLDIFAGCGGLSEGLHQAGVSITKWAIEYEEPAGQAFQLNHPESKVFINNCNVILKAVMDKCGDTDDCISTTDANDLASALDEKEKSDLPLPGQVDFINGGPPCQGFSGMNRFNTSTWSKVQCEMILAFLSFADYFRPKYFLLENVRNFVAFNKGQTFRLTLASLLEMGYQVRFGILEAGAYGVSQSRKRAFIWAAAPDEILPEWPEPMHVFGVPELKINLSSNSYYAAVRSTASGAPFRPITVRDTIGDLPAVGNGASKVNIEYESDPVSWFQKKIRGNMAVLTDHISKEMNELNLIRCKRIPKRPGADWKDLPEEKVKLSTGQLVDLIPWCLPNTAKRHNQWKGLFGRLDWEGNFPTSITDPQPMGKVGMCFHPDQDRIITVRECARSQGFPDSYQFYGNTLHKHRQIGNAVPPTLAYALGRKLKEAVDSKKRSSSQE

>*St*MET 1

MAIRLTAGLQDSQRPCRRLTDFVFHNSKGIPQPFGMSEVDDLFISGLILPLEDSLDKVKAQRIRCEGFGRIEEWAISGYEDGTPVIWISTEIADYDCIKPSGSYKKFYDHFLAKATACVEVYKKLSKSSGGNPDLSLDELLAGVVRAMTGIKCFSGGVSIRDFVITQGGFIYKQLIGLDDTSKKTDQLFVELPVLASLKDESSKQETLAQPEHISSGKALHIGPKAGNGEDKIDESGLANGPAPEDENLKLAKLLHEEEYWCSLKQKKGRNTSSSSSKIYIKINEDEIASDYPLPAYYKTSNEETDEYIVFDSGVDTYHIDELPRSMLHNWALYNSDSRLISLELLPMKACADIDVTIFGSGVMTADDGSGYNFDTDANHSSSGGSRSAEIDGMPIYLSAIKEWMIEFGSSMIFISIRTDMAWYRLGKPLKQYAPWYEPVIKTARLAVSIITLLKEQNRVARLSFGEVIKRVSEFKKDHPAYISSNVDVVERYVVVHGQIILQQFSEFPDASIRNCAFAIGLSMKMEERHHTKWVIKKKKVMQRLEQNLNPRASMAPSVKRKAMQATTTRLINRIWGEYYSNYSPEVSKEVVDCEVKDDEEADEQEENEEDDVPEENLDVPEKAHTPSTRRHIKSCSDSKEIKWDGESIGKTASGEHLFKRARVHGHEIAVGDSVLVEHDEPDELPSIYFVEYMFEKLDGSKMLHGRMMQRGSDTVLGNAANEREVFLINECMNLQLGDVKESIAVNIRMMPWGYQHRNTNADKLDRAKAEDRKRKGLLTEFYCKSFYSPEKGAFFRLPFDKMGLGNGLCYSCELQRTDQEKESFKFDMSNSSFVYLGTEYSVDDFVYVSPDHFTAEREGSGTFKAGRNVGLMAYVVCQLLEIVGPKGSKQAKVDSTNVKVRRFFRPEDISSVKAYTSDIREIYYSEDIHTVPVETIEGKCEVRKKYDISSEDVPAIFDHIFFCEYLYDPLNGSLKKLPAQIKLRFSKIKLDDATSRKRKGKGKEGEDEVGELNETSPQNRLATLDIFAGCGGLSEGLQHSGVTDTNWAIEYEEPAGEAFRLNHPKTKVFIHNCNVILRAVMQKCGDSDDCISTPEASELAAAMDENELNSLPLPGQVDFINGGPPCQGFSGMNRFNQSTWSKVQCEMILAFLSFADYYRPKFFLLENVRNFVSFSQKQTFRLTVASLLEMGYQVRFGILEAGAFGVPQSRKRAFIWAASPEEVLPEWPEPMHVFAVPELKIALSETSHYAAVRSTASGAPFRSLTVRDTIGDLPVVVNGACKTCIKYQGDPVSWFQKKIRGSSITLSDHISKEMNELNLIRCQRIPKRPGADWRDLEDEKVKLSNGQLVDLIPWCLPNTAKRHNQWKGLFGRLDWDGNFPTSITDPQPMGKVGMCFHPEQDRIVTVRECARSQGFPDSYQFSGNILHKHRQIGNAVPPPLAYALGRKLKEAVESKKRLT

>*St*CMT2

MPSKRKSSPATKPESSSGSRKSKRLVVERPDPVVAQPADSDFEQEPVLSSKRKSTRRTTAESSVVACQSESNNKKLKKPAVEKVESGVASPADSDFVSESNSATPSEKSTRQTAVKVEPLVDSVAESDFLEEEDVDGMELGSLKKSLSISPSKRKPKRAEKVKDEECTLAGDPVPDAEARLKWPHRYNKGKSNGTKSLNGQDDPDQLIQAKCHFSRAEVDGQIYYLEDDAHVKAADGEDDYICKIVEFFEAVDGMQYFTAQWFYRAKDTVIKSHDQFIDNKRVFLSEIKDDNPLDCLVTKLKIVPVPSNATSQFEENVKSNCDFYYDMKYLLPYSSFISLPPDTTSPVSSSSTISSDIDAGEVKEHNLEKKLLDLYSGCGAMSTGLCLGANSKGVKLVTKWAVDLNKHACDSLRLNHPETQVRNEYASDFLSLLKEWIQLCVSCSLVKSSVPPHPHLKVIDEVEEDEENDDEGDDSDDDKEGEVFEVEELLEVCYGDPKENNKPGLYFKVRWRGYGPEEDTWEPIDGLSDCPKKIKEFVAKGFKANLLPLPGDVDVVCGGPPCQGISGFNRFRNKENPMQDPKNKQLDVYMDIVDFLKPRFVLMENVVDLVKFSNGFLGRYALSRLVGMNYQARMGMMAAGAYGLPQFRMRVFMFGALSSEKLPQYPLPTHKVIVRGVIPVEFESNTVAYDSGRDLELKKELFLGDALSDLPLVENNEPRDEMPYTDEPKSDFQHFIRMGRDGLPGSILYDHRPLQLNEDDHQRVCQIPKRKGANFRDLPGVRVRPDNKVEWDPDVERVKLPSGKPLVPDYAMSFVGGSSSKPFGRLWWDETVPTVVTRAEPHNQTIVHPLQDRVLTIRENARLQGFPDYYKLIGPIKERYMQVGNAVAVPVARALGYSLAMSIKGLSGEAPLFTLPKNFPSHEEQSCNEVSQ

>*St*CMT4

MEYCVDYSTFRTLHNVKSSVSPSLVDASYKPITTYPLEVSPSCEPMKVELSLLDLYAGCGGMSMGLCLGTKLSGVNLVTKWAVDFNSAACDSLKLNHPQTHVRNEGVENFLELLKRWEKLIKSYGCSDIKTSSNGELDDRDEGENNDDSQSGSNASSGEYEVLRLVDICYGDPNDEGKSGLHFKVRWKGYSPSEDTWEPIANLENCGDSIKDFVRRGQQLKILPLPGDVDMICGGPPCQGISGYNRHRNTDDPLSDEKNRQIIIFMDVVEFLRPKYVLMENVADILRFDKASLGRYALSRLVHMRYQARLGTMAAGCYGLPQFRLRVFFWGALPSERLPPFPLPSHDVIVKYWPSPEFERNTVAYEEGQPRDLEEALVLRDAISDMPAVTWHETREERPYEIPPETEFQKYMRLSKHEIMSCSSTGVKETKEPVLSDHRPCQLNEDDYLRVCLVPRRKGANFRDLPGVIVGGDNVARRDTKDPKVLPNGKPMVPDCAFNFENGKSKRPFARLWWDETVATLVTFPNHRAQAILHPEQDRVLTIREYARLQGFPDFYRFTGTLKERYCQVGNAVAVPVGRALGYALGLAYQRLAGNEPLIKLPSNFSFLTPPIDDIVVLQT

>*St*DRM5

MRVQKFVLDQCRKWNLVWVGRNKVAPLEPDEFEMLLGFPKNHTRGGGISRTDRYKSLGNSFQVDTVAYHLSVLKDMFPNGMNVLSLFSGIGGAEVALYRLGIQLNNVVSVEKSEVNRNIVRSWWEQTNQRGNLIDFDDVQQLNGDRLEQLIDSFGGFDLLIGGSPCNNLAGSNRVSRDGLEGKESSLFYDYVRILDLVKSIMSRQR

>*St*DRM6

MDKNLSGEDNDDIDWDTEDELEIQEIQDTVFPSCTDLRTTGQHVVCCDGEVSSSSVPFRSKFIQQFVVMGFPEESIAKAIEQNGENSDLVLDSLLTFKALDDSPEEQPSVSPPLEPSISSDESASEYNENVLDNVYEEDSWSSDSDNYINTVKQCYLNDEGSSLSEKEKMLLFLANMGYPAEEASIAMDKCGPKASLPELVDFICAAQMSRAEDPYLLEDVKPNLKDILNDCGGYKKRKMYNELCNRKKQREISGEEPIRLPKPMIGFGIPTESVPRMVQRILPKKTIGPPYFYYENVALAPKGVWDTIKRHLYEIEPEFVDSKYFSATARKRGYLHNLPIENRFPLFPLPPRTIHEALPLSKRWWPSWDSRSKLNCLQTAIGSARLTDKIRKAVEKYDGEPPMEIQKYVLYHCKKWNLVWVGRNKVAPLEPDEVEMLLGFPKNHTRGGGISRTDRYKSLGNSFQVDTVAYHLSVLRDLFPNGINVLSLFSGIGGAEVALYRLGVPLNNVVSVEKSEVNRNIVRSWWEQTNQRGNLIHFDDVQLLSRDRLKKLIESLGGFDLVIGGSPCNNLAGSNRVSRDGLEGKESSLFFDYVRILDDVKSIMSRHR

>*St*DRM7

MDNNFSGEDNDNIDWDTEDELEIQEIPDATFSSCTNLRNVGHHTVSGHGEASSSSVPCQSKFIQQFIVMGFPEESIAKAIEQNGENEGLVLDALLTFKALEDSPEEQPSTSAQMEPSISSDDSSSQCNENVLDDVYEDDSWSSDSDNCMNSAKQTYLNDDNSSLSENENSLLFLANMGYPAEEASIAMERCGPEASFSELIDFMCAAQMAREEDVYLPEDEKPKLNSGGYKRKMYNELPVRKKQRAITDEEPIHLPRPMIGFGVPTESLPAIVQRTLPEQAIGPPFFYYENVALAPKGVWDTMSRFLYDVEPEFVDSKYFCATARKRGYIHNLPIEDRFPLLPLPPRTIHEAFPLTKKWWPSWDTRTKLNCLQTSIGSARLADRIRKAMKAMENFDGEPPLMVQKYVLDECRKWNLLWVGRNKASPLETDEFEMLMGFPRNHTRGGGISRTDRYKSLGNAFQVDTVAYHLSVLKDLYPNGINVLSLFSGIGGAEVALYRLGIPLNNVVSVEISEVNRNILRSWWEQTNQKGNLIDFNDIQQLNGDVLEQMIDSFGGFDLVIGGSPCNNLTGSNRVTRDGLEGKDSSLFYDYVRIVDLVKSIMSNRRV

>*Sm*MET1

MFAPMGHGLSFIEVKLPPAFTLPHLPTHTQTPENAHSSATSTQPPPLLHRRDRSSSFAFASFRKRKQISSASTKDMVTNQKEKKKRASQTVEEPTVSRKMPKRAAACSDFKEKSVRISDKDSVIETKKDGVVEEEVLAVRMTAGKEDGRPCRRLIDFTFHNSDGISQPFEMLEVDDIFISGLILPLEESADKEKCKGIRCEAFGRIEEWAISGYEDGSPVIWVSTEVADYDCIKPLGSYKKFYDRFYAKASACVEVYKILSKSSGGNVSLDELLAGVVRSMSGMKCFSRGVSIRDFILSQVLTALKDESSKLVDFAEAQPVSLPGNLRIGPKIEDENKNDLLAKPCLTEEDEDSKMARLLQEEEDWRSMKQKKGRGSSSSSKYYIMINEDEIANDYPLPAYYKMSNEETDEFIIFDSGIDVLHIEDLPRSMLHDWALYNSDARLVPLELLPLRPCDEIDVTIFGSGIMTADDGSGYICDGDSTQSSNGSGASAVEGIPVFLSAIKEWVIEFGSSMISISIRTDMAWYRLGKPSKQYAPWFERVLKTARLAISIITMLKEQIRVSRLSFADVIKRISELAKDHPAFISSTLQVVERYVVVHGQIILQQFSEYPDDIIKKCAFVIGLAKKMEERHHTKWLVKKKKLLHRNEQNLNPRAAIEPVVSKRNAMQATTTRLINRIWAGYYSNYSPEARDGVNGEVKEVHEIEEQEENEEDDALEEKLVVFEQTQTTKSAPRQTKLSSCVEEVKWGGESVGKLLNGEDLYKRAIVHGNEIVVRGAVLLKDDEMDDFPPIYFVEYMFEKSNGTKMFHGRLMQRGCQTVLGNTANEREVFLTNECMDFQLQEVKESIFVDLRSIPWGHQHRKENANAEKIDRARAEERKKQGLPTEYYCKSLYWPERGAFFTLPYSSLGLGSGSCEACNIKESERQKEKFILDASLTSFIYQGTKYSINDYIYVSPSYFSSEEMEAEIYKAGRNVGLKAYVICQLLEICDLKKSKRSEANSVQVKVRRFFRPEDISSEKAYSSDIREVYYSEETHTIPLDVIEGKCEIRRKKDLGPQDIPFTSHHVFFCEHLYDPSRGSIKQLPSHIKLQYSTGQLNDDATFRKKKGKCKEGENDDKEPEKVVSDEHRLTTLDIFAGCGTSLTKWAIEYDAAAGDAFKLNHPGSLVFVNNCNVILRAIMQKCGDSDDCISTPEAAELAASMDQVEVENLPLPGQVDFINGGPPCQGFSGMNRFNQSTWSKVQCEMILSFLSFADYYRPKYFLLENVRTFVSFNQGQTFRLTLASLLEMGYQVRFGILEAGAYGVPQSRKRAFIWAAAPEELLPDWPEPIHVFSAPELKISLSKNLQYSAVRSTSKGAPFRPLTVKDTIGDLPPVVNGASNTSLEYQSDPISWFQKKIRGNLNILTDHISKEMNELNLIRCQKIPKRPGADWRDLPEEKVKLSTGQMADLIPWCLPNTAKRHNQWKGLFGRLDWEGNFPTSITDPQPMGKVGMCFHPDQDRIVTVRECARSQSANSVSLTTYSLVRSGLFFYTRRSSETELEHNTEDVASLPSTPNKLGFPDSYKFFGTIHHKHRQIGNAVPPPLAYALGSKLKEAVEKKRTRNASPTSTATGATPFIRFTFAGEIWQIPYDLGKFTVSFIRFIRHFHPSRSGAAPCFFDEKRLFDINRDRGNVVQS

>*Sm*CMT1

MGRGKKRAEATSSPPPPISASPKKPKRSTPVDTELGFISSPVPSHEAKSRWPHRYQTKNVKTTVTVTLSDGSTEEKELFQARSHYTRAVVDAVVFELFDDAYIQASEGELDFIAKIVEMFETTNGEMYICTQWFYRAKDTVIKNQDYLIDAKRVFYSDIRDDNPLSCISRKAKIKQLPPNMAAAAREKAMDSYDLYVDAMYTFPHAFSNLSKDINNSPLTNGVMETKTEDVKKSPEDSQRSEITVLDLYCGCGAMSTGLGIGASLLGEKIVTKWAVDFNSYACESLKYNHPNTEVRNEDAENFFDLLKEWENLCTKFNVVGSKDIELEPQDSDSEASEVDDGVVIPSEGEYEVQKLLAICYGDPNKSKKTGVYFKVRWKGYGRADDTWEPIDGLSNCEERIKEFVLRGYRARVLPLPGDVDFICGGPPCQGISGFNRFRDALAPLKDKKNHQLVVYMNIVEFLKPRYILMENVTDILKFAKAQLTSYAVGRLVSMNYQARLGIMAAGAYGVPQCRMRFFLWGAGPTEILPQFPLPTHEVLKKGVVINEYKELIIGHGEEHCQLHKAALLGDAISDMPEVTNNTDQDEMPYGNAPRTEFQKLIRLSRQKIFGYSDAKKKNSHKSILHDHRPYQLNQDDYERVCHVPKFKGANFRNLPGVLVGPDRVVKLDESIDRPRCKSGKPLVPDYALSFEDGKSCKPFGRLGMDDIVPTVVTRPEPHNQVLLHPNQDRVLSVRENARLQGFPDCYKLFGPVRERYMQIGNAVSFAVSIPLGYCLAKALQGAQLTTPLSLPFKFPDCLGQLQSLRQEPEELVSE

>*Sm*CMT2A

MKEQQKLPDQLPLRKSSSISSSPSARLCNGAKLEPEARPVDALALSLYVAEAEQQQQPLSICYPSHEEEEEDNSVRRSPRFTSNKYKYLSPERSRSQKHTALVVSKLSMSPRIFFSSSAPALTLTDGGNVEAGPKKRRRLLALPGPAGSGVCLSERCLRSRTVLMRVAESPEASGAKRLRSVGGSPQIEMLALPQATPISSKNRTRPSASLNKECSVTPEKESSLAGVRRNGESAVKSKKKEKSILCCFIGEPIPEEEARERWQWRYDLKSRIKGKNWKINAEEDEIILNVASHYAQAKVGNFVLNIGDCAFVKGEGNKKHIGRILEFFKTTEGEDYFRVQWFFRAEDTVLKDTASSHDKRRIFYSILMNDNVLDCIISKVKIVNVPPMPCLKRNRVQSAAFYYDMEYSVQYSTFRTMVTEKSMGNESFLSNSKHPDDGPITLTPLELLPGYDSPKQELTLLDLYSGCGGMSTGLCLGAKLSSVNLMTKWAVDYNSSACESLRLNHPETNVRNESAEDFLDLLKQWESLCKRYVSDIERTLKCELEELPEAEAEDCLIPSDTEESSGEYERIEDFVRRGFNAKILPLPGDVGVICGGPPCQGISGYNRHRNFASPLEDERNHQIVVFMDTVEFLKPKYCLMENVVDIIRFDKGSLGRYALSRLVLMRYQARLGTVASGCYGLPQFRLRVFIWGAHPGETLPQFPLPTHDVVARYWPPIEFERNIVAYDEGQPRALHGAIVLGDAISDLPPVTNDEDREEMAYGKPPETEFQRRIRTGAGDVLYDHRPLKLSEHDYMRVCRVPHRKGANFRDLEGVVIGEDNVARRDPTREAVMLPTGRPLIPECVFKLELGKSRRPYGRVWWDETVSTVVTFPHIRAMAILHPEQDRRLSIRECARLQGFPDYYRFCGTIKERYSQVGNAVSIPVARALGYALGIAYRKLAGEDPLMTLPPDFSSST

>*Sm*CMT2B

MADSAQLDSADPPLAPPSPPPSLPLQLNGNSDPATPLPSSTDGGSDILDNYPQSNGVAMKKSANRRVETLRTSEDHFRRRSPRLTGNVAGERSVREVSSLKLDGPDEIYPPLKKPKGGKQVSFFIGGPVPDDEARRRWTWRYEGAEGKQNNELSPKHKVVVDDDDDGDKLVSNVKCHYSQAEISKIVFDLGDCAYVKGPKGRPNYVGKILEFFETMDGENYFSVQWFFRAEDTVIKNDGRSHDKKRLFYSTLVNDNLLDCIVSKVKVVQIKPNVCLKFVTLKDNTIPPCDYYFDMKYDVDYSTFSSILTDGDSELFPHPLMMSSKGKYATSKISTKCSLDENCKSELALLDMYSGCGGMSTGLCIGAKACGLDLVARWAVDIDEAACESLKLNHPETQIRNEAAEDFLDLLKEWDCMCRKYGKYDQKELRLKTVREADEVKLSKKNQESSCEYEVARLIDICYGDPSNSGKRGLKFKVRWVGYGPSDDTWEPIENLGKCPEHIRDFVQKGIKAQILPRPGDVDVICGGPPCQGISGYNRFRNFDSPLDDERNRQILIFMDIIEFLKPKFVLMENVIDILRFANGCLARYAISRLVSMHYQARVGIMAAGCYGLPQFRLRVFLWGAQPLEASILNPYLGTSDIPLRRNLVLPQFPLPSHDVVIQYGFPSDFERNVVAYDEGKFRDLEKKVLLSDALSDLPPVANNEKRDSIPLKTVPEAEFQKYIRAAKCDMTASPSSVTTTHKKPVLYDHRPYPMNEDDYLRVCKIPKRKGANFRDLPGIVIGAGNVIRRAKEQDLMPSGKPWVPDYCLSYRDGRSHKPFGRLWWDETVPTVFCFPDHHSRAILHPEQDRILTLRECARLQGFPDYYMFSGSLKQRQVFMSYSQVGNAVAVSVGRALGYSLGMAVQRLSGDEHLLTLPPKFSHSTTVELLSLLNQ

>*Sm*CMT3

MANKRKSESATKDPAAATAEGSRRSKKPASAESTGAVDEETRIPTVIDDGSEDAGEALFLGEPVAGEEARKRWPHRYQKDNQSTEIVQAKCHFLQAKVDGLVYCLEDDAHVKAEEGKESYICKIVELFQAVDGTQNFSAQWYYRADDTVIKTCSNLIDNRQVFFSEVKDDNPLDCLVKKLRIVLLPLKVEDQVKEKLRSTHDYYYDKMYLLPYTSFVNLSSESTTNSNESGSTISSDADGNSVTHVFSELEGPSGEKSLLDMYSGCGAMSTGLCLGANSNGVKLVTKWAVDLNPYACESLKLNHPETQVRNEKADDFFHLLVEWEKLCTSYLSSENKGTSSEHVKEEVEDDSEEDENGDEDDSEVFEVEEILEVCYGDPNEKESPELHFKMGSNDKEPCIHCSSSCPQKLKEFVLRGYKSRILPLPGAVDVVCGGPPCQGISGFNRFRNKEAPLDDPKNKQLVVFMDIVSHLRPKFVLMENVVDLVKFAGGFLGRYAMGSLVGMGYQARMGMMAAGAYGLPQFRMRVFVWGALSTEKLPSYPLPTHNVVVRGVTPLEFEANTVAYEEGCKLDLKKALVLGDAISDLPHVENDEGRDEIPYGSEPQTEFQRFIRQRRDEMPGYLNVKSEVFEHLLCDHRPLQLNQDDYERVCQIPKKKGANFRDLPGVRVRPDNKVEWDPDVPRQKVSSGKPLVPDYAMTFVNGSSSKPFGRLWWDETVPTVVTRAEPHNQAILHPSQDRVLSIRENARLQGFPDYYKLVGPIKERYIQVGNAVAVPVARALGFSLARSLRGLSHNEPVFTLPEDYPMMRDVASPTIDMI

>*Sm*DRM1

MILMFLVPIQDENVSGGDVSDVDWTTDDESEINLNPVTTIVGNEEACSSRSASRSKMFDHFVGMGFSDKLVSKAIEENGEEDSDSILNWLLTFSSLEDSPPQQLSTNFDPCSSDYNEIFPNDLSDMDSWSDDEIEDCLKPNGAPNGAANGVYRSEKHKMLLSLAASGSLTEREEKLLSLADMGYPLEEAEIALERCGPKALIGELSDFLCAAQMAREEDCYLPDDDLKPKHLLSGSSKSKKRKFLEMKKNVIEESIRLPNPMIGYGVPSMQLERVHRSLRDDARAPPYFYYENVALTPKGVWDTISRFLYDIEPEFVDSKFFSACARKRGYVHNLPIENRFPLIPLPPLTIQQAFPLSKRWWPRWDPRDKLNCIQTAIASAKLTERIRSALERVESEADPPESVQRYVMEQCRKWNLVWVGRNKVAPLEPDEVEMLLGFPKNHTRGGGISRTDRYKSLGNSFQVDTVAYHLSVLKDIYPNGVNVLSLFSGIGGAEVALHRLGIKLKNVVSIEKSKVNRDIVRSWWEQTNQTGMLIDFDDVQQFDDTRIEQIIGSIGGFDLVVGGSPCNNLAGSNRVSRDGLEGKESSLFYDYFRILGSVKSVMRNNRFA

>*Sm*DRM2

MCDVVDISDDEDSFLLENDAGVIPKDENLDYDLPPPKTFSMPREEDGASSSGTNLRSSFIGMGFAPALVDKAIKEKGEENSELILEALFSYDALQKPKNEVLDNSFSGECSDSLAGNLRAEEDFHKSESSDSLGRLFAEGTKKNAYVEADIPLKVELDVGDSVIDEKTASLLKMNFSLDEVGFAMQRLVANLQDCNNEVLFGIMEKSLQLLEMGFTENEISAAFERCGSEAPVTLLAESIVTGGTCPLPDKYSSTLLSTSGVRRSFQSSKRKMEDYNSICIKTEEYSSDVASQVRTSDLLEKLKGKMPEVDIDEPNNFKKPKEEFMEDYPSVGNSAAVLQRRGGCFASIDDDRKPTISLPNACRSLSDGVAKPPYFLYGNVTSLSPGSWAKICKFLYSVQPEFVDSHLFSALNRKEGYVHNLPSEDRFHIFPKGPMTIAEAIPYAKKCWPSWDNRKQLSSITCDTTGLPYLCEKLGRMLAECNGPPSAELQTRILQQCVAKNLVWVGKQKLGPLEPEHLEHIMGYPQHHSRIAGYSLTERLKSLKLSFQTDTLGYHLSVLKRLFPSGVTVLSFFSGIGGAEVALHRLGIRLKGVVSVEPCETKRRIMKKWWEKSAQSGELIQLESINKLT

>*Sb*CMT3a
MAPSSPSPAAPTRVSGRKRAAKAEEIQEEEVVVASSAKRSRKAPSSGKKPKPTPKQAKPAKAARKKKGEAERKEPVEDDVCAEEPDEEELAMGEEEAEAAADEQAMQEEVAAVVAGSPGKKRVGRRSAAAGGDHEPEFVGNAVPAAEARSNWPKRYERSTAAKKPEEDEELNAKCHYRSAKVDNVVYCLGDDVYVKAGENEADYIGRITEFFEGTDRCHYFTCRWFFRAEDTVINSLVSINVDGHKHDPRRVFLSEEKNDNVLDCIISKVKIVHVDPNMDPKAKAQLIEHCDLYYDMSYSVAYSTFANISSENGQSGSETASGISSDDVDLEAPSSMPERTATLLDLYSGCGGMSTGLCLGAALSGLKLETRWAVDLNSFACQSLKYNHPQTEVRNEKADEFLALLKEWAVLCEKYVHQDVDSNLAGSEDQEDEGSPLDKDEFVVEKLIGICYGGSGRKDGIYFKVQWEGYGPEEDTWEPIDNLSDCPLKIREFVQEGHRRRILPLPGDVDVICGGPPCQGISGFNRFRNRDEPLKDEKNKQMVTFMDIVAYLKPKYVLMENVVDILKFADGYLGKYALSCLVAMKYQARLGMMVAGCYGLPQFRMRVFLWGALSSMVLPKYPLPTYDVVVRGNTPNAFSQCMVAYDEKQKPHLKKALLLGDAISDLPKVENHQPKEVMEYGGSPKTEFQRYIRLSRKDMLDWSFGEEAGPDKGKLLDHQPLRLNNDDYERVQQIPVKKGANFRDLKGVKVGANNIVEWDPEIERVKLSSGKPLVPDYAMSFIKGKSLKPFGRLWWDETVPTVVTRAEPHNQIILHPTQARVLTIRENARLQGFPDYYRLFGPIKEKYIQVGNAVAVPVARALGYCLGQAYLGESEGSHPLYQLPPSFTALGRTAVQARASPVGTPVGEVVEQ

>*Sb*CMT3b
MAPSSPSSAEPTRVSGRKRAAKAVEIHQSQEEEEEQKVVAASSAKRSRKAASSGKKPKPTPKQAKAGRKKKGEAERKEPVEDDVCAEEPDEEELAMGEAEAEAEAEADEQAMQEEVAAVAAGSPGKKRVGRRSAAAGGDHEPEFVGNAVPAVEARSNWPKRYERSTAATKPEEDEELKARCHYRSAKVDNVVYCLGDDVYVKAGENEADYIGRITEFFEGTDRCHYFTCHWFFRAEDTVINSLVSINVDGHMHDPRRVFLSEEKNDNVLDCIISKVKIVRVDPNIDPKAKAQLIEHCDLYYDMSYSVAYSTFANISSENGQSGSETASGISDGDVDLETSSSMPERTATLLDLYSGCGGMSTGLCLGAALSGLKLETRWAVDLNSFACQSLKYNHPQTEVRNEKADEFLALLKEWAVLCEKYVQDIDSNLAGSEDQEDEGSPLDKDEFVVEKLIGICYGGSGRKNGIYFKVQWEGYGPEEDTWEPIDNLSDCPLKIREFVQEGHRRKILPLPGDVDVICGGPPCQGISGFNRFRNRDEPLKDEKNKQMVTFMDIVAYLKPKYVLMENVVDILKFADGYLGKYALSCLVAMKYQARLGMMVAGCYGLPQFRMRVFLWGALSSMVLPKYPLPTYDVVVRGGAPNAFSQCIVAYDETEKPSLKKALLLGDAISDLPKVENHQPNDVMEYGGSPKTEFQRYIRLGRKDMLDWSFGEEAGPDEGKLLDHQPLRLNNDDYERVQQIPVKKGANFRDLKGVKVGANNIVEWDPEIERVKLSSGKPLVPDYAMSFIKGKSPKPFGRLWWDETVPTVVTRAEPHNQAILHPTQARVLTVRENARLQGFPDYYRLFGPIKEKYIQVGNAVAVPVARALGYCLGQAYLGESEGSHPLYQLPPSFTALGQTAVQARASSVGTPAGEVVEQ

>*Sb*CMT1
MPIHSGPDSSSPVPSPSCSPIPVALEERAPVVIPGSEPPLPLPVASSEAILEEGTLECTFNSVPSPPALPSSDRPDLGAEDGPNDWSSLLKVLSGSMPVIDMAATERPGDNAANCSMCMSDIATGDGTPLRSSPRIMAVKTKANAASLEHEIESSRASEHRLAVSRQISTSEGSQQPVSARLFEETTVASGKPVHLPDGVKPQGSPDIIDIYTSSTDVVVALPVVSKRSLKNKVVSIKTKANAASLEQEIESSRASKHRLAVSQQSTVSEGSQQPVSARLFEETTVSSGKPVHLPDGVKPQGSPEIVAIHCSSTDVVVALPVVSKRSLKNKVVSSSPRKTRSSSKVVVNSNRVSAVSQVMNCVPAVHKASSSIPPRKHKLASEKCLPNLERVDAMLHNSGLISANKAVHGTPLEVEAALSQPPKAKQPRICLGKCSLNLKSAENSSSSICELPMVTVTSAPEDKPISAPENKPLDTHCTDSEMVDSMDGSYFFVGDAVPDEEAQKRWPHRYQNNHCLLKKDKRSNTQTVSNVGKAVLDVKCHYLEASICGSTLCIGDCAFIKGLEGKPHYIGRLLEFFETTADEYYFTVQWFFRAEDTVMEDQAQSHDPRRLFYSDLKDDNLLDCIVSKVTIMPVSPSLNEKPRLIPSFHYYYDMKYSLDYSTFSTMEMGDPNDTLHSHYTSSNNVKRIDFTEKQKSPTSEMRELSLLDLYCGCGGMSTGLCLGARGGGVNLVARWAVDGDEVACETFRLNHPETRVRNETTDDFLELLKEWKKLCKTYVKPCSKVKSQSNVPTQSSNGTPDCSTFSTEEFEVWKLVDICFGDPNGVRKRALYFKVRWKGYGPNDDTWEPMEGLKNCKDAIRDFVIEGHNKKILPLPGDVDVICGGPPCQGISGYNRNREFDAPFNCERNKQIIVFMDVMQFLKPKYIYMENVLDILKFADATLARYALSRLVSMHYQAKLGVMAAGCYGLPQFRMRVFLVGCHPKEKLPPFPLPTHEAIVKNGCPLAFERNLVGWPDSMPMQLEKPIVLEDALSDLPEVENGEKREEMLYMKGPQTEFQRYIRSFNSEVLGSRAHVIKVSKSKLFDHRPRALDVDNYLRVLQIPKKKGANFRDLPGVIVGPDNIARLDPTKERILLPSGNPLVVDCVLAYKNGKSLRPYGRLWWDEVVGTVLTCPNAHMQALIHPAQDRLLTIRESARLQGFPDSFRFRGTVKDRYRQIGNAVAVPVGRALGYALAMAYLNKTGDDPLMVLPPKFAFSHDVQGLSSVNGLHGRPIG

>*Sb*MET1
MTHELSGDMVKTPRSPVTTGTRRCRAKPQKKEKESTENSKLENGSQDATEEVHHGVEKGDGHVTRKRPRRAAACSDFKEKSIRLSEKKSVVMVKKNRMEEEEIDAVSLTKLGPEDPPPCRKLIDFILHDAEGNPQPFEMSEIDDFFITALIMPMDDDLEKERERGVRCEGFGRIEDWNISGYDEGTPVVWVSTDVADYECVKPATNYKSYFDHFYEKAQVCVEVFKKLAKSVGGNPNQGLDELLASVVRSINAMKGYSGNMSKDLVISIGEFVYNQLVGLDETSSNDDEKFATLPVLLSLRDQCRSRVELTKLPSNISNTSLKIKDTVCEGTAEEEDDDAKLARLLQQEEEWKMMKKQRGRRGTPSQKNVYIKISEAEIANDYPLPAYYKPFSQEMDEYIFDSDDSIFSDDVPVRILNNWTLYNADSRLISLELIPMKSGAENDVVVFGSGFMRDDDGSCCSTAESVKSSSSSSKADQQDAGVPIYLSPIKEWLIEFGGSMICITIRTDVAWYKLRQPTKQYAPWCEPVLKTARLAVSIITLLKEQSRASKLSFADVIKKVAEFDKGNPAFISSNIALVERYIVVHGQIILQQFADFPDETIRRSAFVSGLLLKMEQRRHTKLVMKKKTQVMRGENLNPSAAMGPASRRKAMRATTTRLINRIWSDYYAHHFPEDSKEGDGNEAKEVDDEQEENEDEDAEDEGQIEEDKISKTPPSTRSRKLLSQTCKEIRWEGETSGKTSSGETLYKCAYVRELRIPVGGTVVLEDDSGDTVMCFVEYMFQKSGGAKMVHGRILQKGSHTILDNAANEREVFLTNDCLEFKLDDIKELVSVDIQSRPWGHKYRKENSETDKVEQAKAEERKKKGLPMVYFCKSLYWPEKGAFFALPRDKMGLGSGVCSSCEHIEPDSDELKVLSKTSFVYRKVTYNVNDFLYIRPDFFSEDEDRATFKAGRNVGLKPYAVCQILAIPEGAGSKKLNPASANISARRFYRPDDISTAKAYASDIREVYYSEDVIDVPVDMIEGKCEVRKKNDLASSDLPVMFEHVFFCELIYDRASGALKQLPPNVRFMSMVQKATIASKKNKGKQICETDQIDSGKWLDVPKENRLATLDIFAGCGGLSEGLQQAGVSFTKWAIEYEEPAGEAFSKNHPEAVVFVDNCNVILKAIMDKCGDTDDCISTSEAAEQAAKLPELNINNLPVPGEVEFINGGPPCQGFSGMNRFNQSPWSKVQCEMILAFLSFAEYFRPRFFLLENVRNFVSFNKGQTFRLAVASLLEMGYQVRFGILEAGAFGVAQSRKRAFIWAAAPGEMLPDWPEPMHVFASPELKITLPDGQYYAAARSTAGGAPFRAITVRDTIGDLPKVENGASKLTLEYGGEPVSWFQKKIRGNMMVLNDHISKEMNELNLIRCQHIPKRPGCDWHDLPDEKVKLSNGQMADLIPWCLPNTAKRHNQWKGLYGRLDWEGNFPTSVTDPQPMGKVGMCFHPDQDRIITVRECARSQGFPDSYEFAGNIQNKHRQIGNAVPPPLAYALGRKLKEAVDNKRQEAGVGVPAP

>*Sb*MET2
MTHELSGDMVKTPRSPVTTGTRRCRAKPQKKEKESTENSKLENGSQDATEEVHHGVEKGDGHVTRKRPRRAAACSDFKEKSIRLSEKKSVVMVKKNRMEEEEIDAVSLTKLGPEDPPPCRKLIDFILHDAEGNPQPFEMSEIDDFFITALIMPMDDDLEKERERGVRCEGFGRIEDWNISGYDEGTPVVWVSTDVADYECVKPATNYKSYFDHFYEKAQVCVEVFKKLAKSVGGNPNQGLDELLASVVRSINAMKGYSGNMSKDLVISIGEFVYNQLVGLDETSSNDDEKFATLPVLLSLRDQCRSRVELTKLPSNISNTSLKIKDTVCEGTAEEEDDDAKLARLLQQEEEWKMMKKQRGRRGTPSQKNVYIKISEAEIANDYPLPAYYKPFSQEMDEYIFDSDDSIFSDDVPVRILNNWTLYNADSRLISLELIPMKSGAENDVVVFGSGFMRDDDGSCCSTAESVKSSSSSSKADQQDAGVPIYLSPIKEWLIEFGGSMICITIRTDVAWYKLRQPTKQYAPWCEPVLKTARLAVSIITLLKEQSRASKLSFADVIKKVAEFDKGNPAFISSNIALVERYIVVHGQIILQQFADFPDETIRRSAFVSGLLLKMEQRRHTKLVMKKKTQVMRGENLNPSAAMGPASRRKAMRATTTRLINRIWSDYYAHHFPEDSKEGDGNEAKEVDDEQEENEDEDAEDEGQIEEDKISKTPPSTRSRKLLSQTCKEIRWEGETSGKTSSGETLYKCAYVRELRIPVGGTVVLEDDSGDTVMCFVEYMFQKSGGAKMVHGRILQKGSHTILDNAANEREVFLTNDCLEFKLDDIKELVSVDIQSRPWGHKYRKENSETDKVEQAKAEERKKKGLPMVYFCKSLYWPEKGAFFALPRDKMGLGSGVCSSCEHIEPDSDELKVLSKTSFVYRKVTYNVNDFLYIRPDFFSEDEDRATFKAGRNVGLKPYAVCQILAIPEGAGSKKLNPASANISARRFYRPDDISTAKAYASDIREVYYSEDVIDVPVDMIEGKCEVRKKNDLASSDLPVMFEHVFFCELIYDRASGALKQLPPNVRFMSMVQKATIASKKNKGKQICETDQIDSGKWLDVPKENRLATLDIFAGCGGLSEGLQQAGVSFTKWAIEYEEPAGEAFSKNHPEAVVFVDNCNVILKAIMDKCGDTDDCISTSEAAEQAAKLPELNINNLPVPGEVEFINGGPPCQGFSGMNRFNQSPWSKVQCEMILAFLSFAEYFRPRFFLLENVRNFVSFNKGQTFRLAVASLLEMGYQVRFGILEAGAFGVAQSRKRAFIWAAAPGEMLPDWPEPMHVFASPELKITLPDGQYYAAARSTAGGAPFRAITVRDTIGDLPKVENGASKLTLEYGGEPVSWFQKKIRGNMMVLNDHISKEMNELNLIRCQHIPKRPGCDWHDLPDEKVKLSNGQMADLIPWCLPNTAKRHNQWKGLYGRLDWEGNFPTSVTDPQPMGKVGMCFHPDQDRIITVRECARSQGFPDSYEFAGNIQNKHRQIGNAVPPPLAYALGRKLKEAVDNKRQEAGVGVPAP

>*Sb*DRM3
MQVKIEDHAEDGGIDADVRGLGEAAVDPLPGSVHATFKEEEGEPSSSSGKLRSEFIGMGFSSKLVDKVLQRHGDDDSNTILESLLSYSDLKQSGSESSGSLGSLFDSDNEENNSSLESRKVINQDIKPELDSFSEKWSFLLRTMNFSQQEVDLAFKQLGDEAPLEQLVDFIVNAQSGVSSGGPENGDATNEGKTELLYGVMEKTLSLLQMGFTEEEVSSVLENSDQRATIQELADSILARRIANSIEQKEVKIESDFLGEAEPDYSSYQPSYSAASYYDDDNNNIRVKRAKHVFIDDTGASSSHLGNPWSMGTSDMPVKVELEAMTPGRRANVQGDLAKPPFFLYGNMVDIPKDTWYQLTQFLYNVEPEFVNSQSFSALTRKEGYIHNLPVEKRRVVVPKSPMTIEEALPFTRQWWPSWDTRKHISVVTIEVGEIEQTCERLGSMVRESRGVLSEARQMQIIHQCRVSNLIWVGQNKLGPLEPRQVERILGYPHNHTNLFELNQSDRFGAMKFAFQTDTLSYFLSVLKGMYPDGIRVLSIYSGIGGAEVTLHRLGIPLKCVISVEESEVNRKILRRWWSKTEQTGVLRQHPGIWKLKTHVIEDLVKEFGGFDLIIGGNYTSCKGGNTVNTTMGLDSNRFYEYARVVKRVRTAVGLS

>*Sb*DRM1

MADRVGGSDDGDKSEWNSDGEVARSLDGAGSPIAPSRNKLKDAPGPSLLVNGAGPSASLVEDFVGMGLIKGNGDGGAESLLELLLAYKAIGNDPSLDKSSASVCGPRTICDDKDILANWDAHDASTSSDRDHASDDSGDEDFLQGLSQKDEKIESLVRMGFPKDEAEMAIVRCGQDAPMSVLIDLIYSSEASEDGYYGNFSGHEDDSFGGRKEKRKRSEDAEQGSSHDEPMPLPNPMVGFNLPNVSLRSVDRSLPSKAIGPPFFYYENVAIAPKGVWTTISQFLYDIQPEFVDSRFLCAAARKRGYVHNLPIENRSPLLPLPPKTIFEAFPHTKKWWPSWDPRKQFNCLLTSMAKPKLTEQIHHALAKCKDPPPRRVQKYVLETCRTANLVWVGLNKVAHLEPDEMEFLLGFPKDHTRGIGRPERFKSLGNSFHVDTVAYHLSALRDMFPHGMNVLSLFSGIGGAEVALHRLDIRMKTVVSVEISETNRFVLRTWWNQTQTGTLIEITDVQSLTTERLESCIRRIGGFDLVIGGSPCNNLAGRNRFHRDGLEGEQSSLFYHYYRILDTVKSIMGRM

>*Sb*DRM2A

MAHWVSDGDGSDSFEWDSDGNGEEAGSFNTAGASSSAMASTNTDAPGPSRRVSQVANGNGKAGPSASLVQRYIDMGFAEEIVVKAIKDNGDNGADALVELLLTYQELGNDLNVDNGFASGCVPQTVDDSGDDDFLENWDDLDAGGRSTRVANSVDDSGDEDFLHEMSQKDNKIDSLVKMGFPEDEAALAITRCGQDASISVLADSIYASQTAGDGYCGNLSDYEDGGRNKGRFMDGNNKKRKRYGSQALGSRGPLDGSADEPMLLPNPMVGFSLPDQWPRPVNRDLPALAMGPPYFYYENVALAPKGVWTIISRFLYDIQPEFVDSKYFCAAARKRGYIHNLPLENRSPLLPKPPRTITVAFPHTKRWWPSWDPRQQFNCLQTCVSSAKLLEKIRVTLTNSSDPPPPRVQKLVLEECRKWNLAWVGLNKVAPLEPDEMEFLLGFPKDHTRGICRTERFRSLGNSFQVDTVAYHLSVLKDMYPEGMNVLSLFSGIGGAEVALHRLGIRMKTVISVEKSEVNRTILRSWWDQTQTGTLIEINDVQTLTSERIEAYIRRFGGFDLVIGGSPCNNLAGSNRHHRNGLEGEHSSLFFQYVRILESVKSIQRL

>*Sb*DRM2b
MAHCVGDSDGSDNFEWDSDGDGEEAASFNAAGASSSAMASTNADAPGPSTRVANGNGKAGPSASLVHKYMDMGFPEEIVLKAMKDNGDNGADSLVELLLTYQELGNDLKVDNGFASGCVPQNVDDSDDDDILENWDDEDAGGRSTGVANSVDDSGDEDFLHEMSQTDEKVYSLVKMGFPEDEAALAVTRCGQDASISVLVDSIYASQTAGDVYCGNLSDYEDNSYGGRNKGRFMDANKKRRKRYEGQAHGSRGPLDGSSDEPMPLPHPMVGFSLPHQCTRPVDRSLPSQAMGPPYFYYENVALAPKGVWTTISRFLYDIQPEFVDSKYFCAAARKRGYIHNLPLENRSPLLPIPPKKISEAFPHTKRWWPSWDPRRQFNCLQTCVSSAKLLERIRVALTNSSDPPPPRVQKFVLEECRKWNLAWVGLNKVAPLEPDEMEFLLGFPKDHTRGISRRERYRSLGNSFQVDTVAYHLSVLKDMYPQGMNVLSLFSGIGGAEVALHRLGIRMKTVISVEKSEVNRTILKSWWDQTQTGLLIEICDVQTLTSERIEAYVRRIGGFDLVIGGSPCNNLTGSNRYHRDGLEGEHSSLFYHYVRILDSVKSIMERL

>*Bd*CMT3
MAPSSPSSAAAAAPARASSRKRTASAKAELAQEATKRPRKAAASSGKKKVTKPKAKAEKAPRKKKEVEVLGEDEVCAEEPDEEELALGEEDESAASGEQQPEEPAAARKRVAQPIRARNQAGGDKDHGFVGAPFPAGEARSKWPQRYQPTKPRRPEEEEEDPKARCHYRSAKVDEAIYNLGDDVYVMAGENEPHYIGRITEFFEGIDKKCYFTCRWFFRPEDTVISTAKFVNDHTHDPKRVFLSEEKNDNVLECIVLKVNIVHVDPNMDSEAKAQLVAESDLYYDMSYSVAYSTFANITSDTNDNSGISSDVDSEAASPVRTAVLLDLYSGCGGMSTGLCLGSALAGLKLETRWAVDLNSFACKSLKYNHPGTEVRNEKAEDFLALLKEWAILCDTYVHGNNSDPASPSEDEEEDDEPLGKDEFVVEKLLEICYGGSGREKAIYFKVQWKGYGPEEDTWEPIGNLSDCPLKIKEFVQEGHKRNILPLPGDVEVICGGPPCQGISGFNRFRNRKEPLKDEKNQQMVTFMDIVSYLKPKFVLMENVVDILKFADGYLGRYALSRLVSLNYQARLGIMVAGCYGLPQFRMRVFLWGALPAMVLPKYPLPTHDVVVRGGAPNAFSQSIVAYDETQKPTLKKALLLGDAISDLPKVDNFQPHEVMEYGAQPKTEFQSYIRLSRKDMLDYSFGDNTCPEEGKLLDHQPLRLNQDDYDRVQQIPVKKGANFRDLPGVKVGANNIVEWDPEVQRVYLKSGKPLVPDYAMSFIKGRSLKPFGRLWWDETVPTVVTRAEPHNQIILHPNQARVLTVRENARLQGFPDYYRMNGPIKEKYIQVGNAVAVPVARALGYSLGRAYQGEMEGSNPLFTLPDSFTNVGQTVVLARASSVGTPAGEVVEQ

>*Bd*CMT3b

MAPSSPPSAALRASSRKRTASAKAAPDQEPSAATKRPRKGAASSSAKKKTADPKAKALKAPRKKKEVAAAEEKLSEDEVCAEEPDEEELELGEEDESAASGEQGQATARRRVAQPTRERNLAAGDKEPEFVGEPFPADEARSKWSQRYQRAAPRRPDEEPELKARCHYRSANVDGTVYALGDDVYVKAAENEADYIGRITEFFEGTDRHCYFACRWFFRPEDTVISTAKFVDDHTHDPKRVFLSEETNDNVLDCIIKKVKIIHVDPNMDPEGKAQLVADSEAELYYDMSYAVAYSTFANIPSDTNENSGISSDADLEAGTPPVRTAALLDLYSGCGGMSTGLCLGAALAGLKLETRWAVDLNSFACKSLKYNHPGTEVRNEKAEEFLALLKEWAILCDTYVHVNNSESDSPIEDEEEDDEPLAKDEFVVEKLLEICYGGSGRGKGIYFKVQWKGYGPEEDTWEPIGNLSDCQLKIKEFVQEGHKRKILPLPGDVDVICGGPPCQGISGFNRFRNRKEPLKDEKNQQMVTFMDIVSYLKPKFVLMENVVDILKFADGYLGRYALSRLVALNYQARLGMMVAGCYGLPQFRMRVFLWGALPDMVLPKYPLPTYDVVVRGIVPNAFSQSVVAYDETQKPTLKKALLLGDAISDLPEVDNYQLHEVMEYGTKPKTEFQRYIRLGRKDMLDHSFGDNTCPEEGKLLDHQPLRLNQDDYDRVQQIPVKKGANFRDLPGIKVGANNIVEWDPEVPRVYLKSGKPLVPDYAMSFIKGRSLKPFGRLWWDETVSTVVTRAEPHNQIILHPNQARVLSVRENARLQGFPDYYRMNGPIKEKYIQVGNAVAVPVARALGYSLGQAYQGEMESTGPLFTLPASFTNVGQTEELARASSVGIPVGEVVEQ

>*Bd*CMT1
METPPHNPISPPSPPPVTDGSPAPYAGADGDEAAGGFDGSLESMLERIFGPPELEPWSPPREFDFAAEFAAVAATDPDPLEDLGGGPWDAAPWMSTGLVACEGAAATTRAPSPPTAAPGFAPAPTPAFDSSEGAPEVRSLDHLDNYSPMPESPSTPVAVDMREKLVFVSDSAPVLESSPSPPVACPDLSTTEPMKETTNAAPERSDLPLPNRVEQSTSSLSNQVVLALQTPQVVREMPQEVEGKSSRPLKAKRVRGSTDKCLPNLKRAKKNSGSIGRLPNVTTMPDPGNTHEVILDKHLTDSEMVESDDGSCFFVGEAVLEEEAKQRWPHRYKKNHHFVEKFLSLKDKRSGNQPFANAGNDVLDVKCHYLQASVSGSTLCVGDCAFVKGPEGRPNYISRILEFFETVAGECYCRVQWFFRAEDTVMEDQAQSNDPRRLFYSDLQDDYSLDCIVSKLTIVQVPPCVDKESKSIPSSQYYYDMKYSLDYSTFSTLEMEDTNAILQSSHASSIEMKKVDFSKKQKSPVPNKKDLSLLDLYCGCGGMSTGLCLGAHGAGVNLVKKWAVDDDLVACESYRLNHPETRVRNETTNDFLLLLKEWQKLCKKYVEQSEVKDHADGLTETINEIPDDSVDPTEELEVWKLVDICFGDPNGIENHCLHFKVRWKGYGRNDDTWEPIDGLGNCNEAIRDFVIEGHKRKILPLPGDVDVVCGGPPCQGISGYNRNREFDAQFKCEENKEIIFFMDVMQFLKPKYVYMENVLDILKFADATLARYALSRLVAMRYQAKLGIMAAGCYGVPQFRMRVFLLGCHPEEKLPPFPLPTHEAIRKNGCPLAFERNLVGWSDGTTVQLAKPIVLEDILSDLPEVGNEESRDEMPYVKDPQTEFQRYIRAFNSEVRGPKSRAAKSKSRKAKPKLYDHRPFVLDNDNYLRVLQIPKKKGANFRDLPGVVVGPDNVAKLDPTKERILLPSGNPLVLDCILKYEDGKSLRPFGRLWWDEVVGTVVTCPNAHTQAFIHPAQDRLLTIRESARLQGFPDSYRFHGEVKERYCQIENAVAVPVGRALGYALAMAYLNKTGSDPLMVLPPKFAFSQNIEGTL

>*Bd*MET1
MVKTKSPRSPVTTGTKRCRAKPQKKKEESTGEGQLETEPKDSAEVMHNEVENGAGSAARKRPRRAAACSDFKEKSVRLSEKSNVVMIKKNRMEEEEIDAINLTKLGPEDSPPCRKLIDFILHDADGNLQPFEMSEIDDFFITALIMPVDDDLEKERERGVRCEGFGRIEDWAISGYDEGTAVVWLSTELADYECVKPASNYRSYYSHFYEKAQVCVEVYRKLMRSVGGNPNLSLEELLATVVRSINAIQGYSGTMSKDFVIATGEFVYNQLIGLDQTAGNDDEKLATLPVLLALRDECKSRVEFNKMPPKISNGSLKINDAECNEVAEDDDEKLARLLQEEEEWKMMKKQRGKRGVPAQKNVYIKISEAEIANDYPLPAYYKPSTQEMDEYIFDGDDGMFSDDVPVRILNNWVLYNADSRLISLELIPMKSGTENDIVIFGSGFMRDDDGSCCSTAESANSSSSSSKAEHQDAGVPIYLSPIKEWLIEFGGSMICITIRTDVAWYKLRQPIKQYAPWCEPVLKTARLAVSIITLLKEQSRASKLSFVDVIKKVAEFDKGDPAFISSNISLVERYIVVHGQIILQQFADFPDETIRRSAFATGLLMKMEQRRHTKLFMKKKAQVTRGENLNPIATMGTSSKRKAMRATTTRLINRIWSDYYAHHFPEDSKDGDENEAKEIDDEQEENEDEDAEEEVQIEEEKVSETPPSTRSRKLVSQTSKEIRWEGKSTGKTASGEALYKCGYARELRIAVGGTVTLEDDSGEIVICFVEYMFQKPDGEKMVHGRMLQKGSETVLGNAANERELFLTNDCLEFELKDIKELVSVNLQSMPWGHKYRKENSEADKIERARVEERKKKGLPMEYLCRSLYWPEKGAFFSLPHDKLGLGNGVCSSCEHREPERDELRILSKTSFIYRKVTYSVHDFLYIRPEFFSQEEDRGTYKAGRNIGLKPYAVCHLLDVCEPVGSKKINPASAKVSVRRFYRPDDI

SSDKAYTSDIREVYYSEDIINVPVDMIEGKCDVRKKIDISNSDLPVMVEHVFFCEHIYDPMTGALKQLPPNVKLTSMVQKAAGALKKNKGKQICENDQVDSDKRKEVPKENRISTLDIFAGCGGLSEGLQQAGASFTKWAIEYEEPAGEAFRQNHPEAAVFVDNCNVILKAIMDKCGDASDCVSTSEAAEQAAKLAEENIKNLPVPGEVEFINGGPPCQGFSGMNRFNQSPWSKVQCEMILAFLSFAEYFRPRFFLLENVRNFVSFNKGQTFRLAVASLLEMGYQVRFGILEAGTFGVAQSRKRAFIWAAAPGEILPDWPEPMHVFASPELKITLPDGKYYAAAKSTAGGAPFRAITVRDTIGDLPKVENGANKLILEYGGEPTSWFQKKIRGSTIALNDHISKEMNELNLIRCKHIPKRPGCDWHDLPDEKVKLSSGQMVDLIPWCLPNTAKRHNQWKGLYGRLDWEGNFPTSVTDPQPMGKVGMCFHPDQDRIITVRECARSQGFPDSYQFAGTIQSKHRQIGNAVPPPLAFALGRKLKEAVDAKRQQA

>*Bd*MET2
MEKVPHTAVTTGTKRRRAKPREMADESIEDDRLETEFANDKKESNGATENGDRPIAHKRRKRAAACSNFKEKAFDLSEDDSLVAIKESRVEEEIGAVRLTKTGPEDKKSCRKLIDFILHDENGNAQPFEMSHSDGISITALVMPLDDNMEKDREKGIRCLRFGPIKNWAISGYKQGTAAIWLSTELADYKCVKPASGYRSYFDHFSRKADVCVEVYKKLARSVGGNLQLGLEELLASVVRSINSNRSFNGTVNKDFVISIGEFIYNQLSGLDNTSDKDDETLSELPVLVALRNECTSRIEFNKLPGMTSSETLKIKDGQCKEEVTRNEDEDEKLARLLHEEEEWKLMKQRSKRQASKKNVYIKISETEIANDYPLPAYYKPYSLEMDEYMFDSDMGMFSDDVPVRILNNWALYNSDSRLISLELIPMKSGAENDIVIFGSGFMREDDGSFFSTAEPTQLSSSSSKSDQEDQGVPIYLSPIKEWVIEFGGSMICISIRTDIAWYKLHQPLKQYAPWCDTVLKTARLAVSVITLLKGQSRASKLGFADVIKKVAEFESGHPAFISSNATLVERYVVVHGQIILQQFANYPDPSIRRSAFVTGLTAKMEDRRHIKLVMKKKSQPTRGENLNPSANIGPIIRRKLMRATTTRLISKIWGDYYATHFPEDSKEGAEDEPKEIEEEQEENEEDAEEEVKVEEEHVLSTPPSARSRKSSSNNCKEIEWEGQTVGKTRSGDDLYKCARVRELNISVGGAVKLEDDSEDALMCFVEYMYEKQDGTHMIHGRILQKGSQTVLGNAANEQEVFVTNDCLEFEIGDIKELVSVNVQLIPRGHKYRKENLQANRIERAKAEERKKKGLPMEYICKSLYCPEKGAFFSLPYDKLGTGTGICSSCQQREAVGDEFKILSETTFVFKNSTYAVHDFLYVRPEFFSRVDGHETYKAGRNVGLKPYVVCHLHSINAPAGSKKVNPESTKVSVRRLYRPDDISSARAYSSDIREVYYSEDMLSVPVAMIEGKCEVTTKNNLPDSNLPVVVDHVFYCEYLYDPDTGALKQLQSNVKLMTIARKAPTSKKNKGKQICDSDQAGSDKQKAAASENSLATLDIFAGCGGLSEGLQLSGASHTKWAIEYEEPAGQAFGENHPEAAVFVENCNVILKAIMDKCDDTDDCISTSEASEQAAKLSDEKIKNLPVPGEVEFINGGPPCQGFSGMNRFNQSPWSKVQCEMILAFLSFAEYFRPRFFLLENVRNFVSFNKGQTFRLTLASLEMGYQVRFGILEAGAYGVAQSRKRAFIWAAAPGETLPDWPEPMHVFASPELKITLPDGKYYAAAKSTAGGAPFRSITVRDTIGDLPPVENGASKPTIQYGSEPISWFQKKIRGDVLSLNDQISKEMNELNLIRCKHIPKRPGCDWHDLPDEKVKLSSGQMVELIPWCLPNTAKRHNQWKGLYGRLDWEGNFPTSVTDPQPMGKVGMCFHPDQDRIITVRECARSQGFLDGYRFAGNIQSKHRQIGNAVPPPLAYALGRKLKQAINARN

>*Bd*DRM1
MVDLISDSDDSVAFEWESDGDAEPSSAPVLRDFDAPGPSTLVRQDTNGRPNGEALPASLVEEYVGMGFPKDMVLKAIKQIGNLSSNNFHFICLVLFAPCVSTDSFAFRSRDANSLVELLLTYKVLAESDASVGNPSSSGCAPHSVENDDDDNLDSEDWDDDDDSDGSSDEDFLQEMSKEDDKAKSLVDMGFPVEEAKMAITRCGADAASIVLVDSIYASQAADSGNVFDHEVSDRSFRSFGERKRAISIEGSKKKAKRYGGGAQGNRTPLDGSDDEMPLPNPMNRRLPVLATAPPFFYYENVARAPKGEWREMSRNLYDIHPEFVDSVHLCAAARKRGYIHNLPIVNRSPILPLPPKTILEAFPHYSKWWPSWDSRKKLNCLQTSGASAKLTERIQLTLANSTNPPPPHVQKYVLNQCKRWNLVWVGKNKVAPLEPHEVEYLLGFPKDHTRGICKKEREKSLGNSFQVDTVAYHLSVLRDKFPNGMNVLSLFIGIGGAEVALHRLGIRLKTVVSVEISPANRRILKGWWDQTQTGTLIEIEDVKTLKSDTIASFVSKYGGFDLVIGGSPCNNLAGSNRHHRVGLEGEHSSLFYHYPRILGDVKRAMDGR

>*Bd*DRM2

MDCISDSDDSAKFEWESDNEAEPSSAPVLRNFDAPGPSTDANGWANGDAPSTSLVEEYVGMGFPKEMVLKAIKEIGHNDANALLELLLTYKVLGEDPTVGNCSTLGCAPQSVEDDDDGDLDSEDWDDEDDADGGEPNFDSSGDEDFLQEMSEHDKKIKSLVDMGFPEDESNMAIVRCGVDAALTVLVDSIYASQAAGDCNSRNSSHHEVCDSFGGRRNKKKRKQYGGGAQGNRPSECHEELMPLPNPMVGFSLPTARLPSVSRRLPKQATGPPFFYYENVALAPKGVWTIISRNLYDIAPEFVDSKYMCATARKRGYVHNLPIENRSPLLPLPPKTIFEAFPHYKKWWPSWDPRRQLNCVQTCVSSAKLTERIQCALARSGDPPPLHVQKYVMHECRKWNLVWVGKNKVAPLEPDEMEYLLGYPRDHTRGIGKTARYKCLGNSFQVDTVAYHLSVLRDIFPNGLNVLSLFTGIGGGEVALHRLGIHMKTVVSVEISEVNRRILRGWWDQTQTGTLIEIPDVQSFTSDKIRSFIRRFGGFDLIIGGSPCNNLAGSNRHHRDGLEGEHSSLFYHYPRILDTVKDVMAGM

>*Bd*DRM3

MARTKRPAKRKPKGAANLKLPVNSRHCGSSSRFMEYAALEADLIKMVKTEDQDEDEGVSASVGALRDGTVDPQPDTAHAGVKAEEDGQPSSSSSYSKFIGMGFPPTLVDKMLQKHGDKDFNAILESLLFHSSLTKSGSESSSSLGSLFDSDSEENISRLGSMKEPHEEIKPEPDSFSERRSYLLSAMNFSQQEVDWAFNQLGEEAPLDQLVDTIVTAQVAGFTGGNENVDATTEGKAESLFGVMEKTLHLLQMGFTEEEVSGAIDSSGQEATVQELADSIFARRIDNSIKQTEVKIESDFLGGTENQHSTCHQRLRYYDDEDDKIGVKRAKSIFTDNSSGASSSRPGNQPSLTPWLSGCTGSVSNGYVKEEFDAMASGPRPDVRPEIAKPPYFLYGNVVDIPKGTWHQLSDFLFNVEPEFLNSQYFSAVMRREGYLHNLPMETRHIVVPKSPMTIEDALPFTRQWWPSWDTRKHIGVVTLDVAEIEQMCEKLGKIMTDSRGVLSHEKQAHIMQQCKMSNLIWIGKDRLSPLEPHQLEHILGYPRNHTEQFELNTPDRLAAMKYTFQTDVLAYLLSVLKSKYPNGLRLLSIYSGLGGAEVALDRLGIPIKCIVSVEESDVNRKILRKWWRRTNQAGELRQFVGIWKLKTIVLEDLVKEFGGFDLIIGGNYSSCRGGRTINATMGMDSNKFYEYARVVKIVRALHNS

>*Rc*CMT1

MGRSAKRPNREPEVEEEDGATLSDQKGPITLLSARPKETKKAKLDADLSFIGNPISATEARKKWPQRYKSQISKVKNGSEPQNGVLSKDDDVTQAKCHYKQAMVDGILYDLGDDAYVKAEDGKPDYIARIVEMFESIDGEPLFTAQWFYRAEDTVIKDYVKTAESRRVFLSEIRDDNPLDCIVSKVKIALVEPNLDLAEKERNLPPCDLYYDMKYTLPFLTYETIKTDDSGRDSGSSSTISSENDSNNSIDDVKVTTAKPLKVLSKVHSSEKSELYLLDLYSGCGAMSTGLCMGASLSGVKLVTKWAVDINAFACKSLKTNHPETEVRNEAAEDFLSLLKEWEKLCRKFSLFGSEKHPEQSSNSASDEEEEDEEEEEEEKGKDKDDDDDEIFEVEKLLAVCYGDPNKVNKRGLYFKVRWKGYGPSEDTWEPIEGLSDCKDKLKEFVTKGFRSKILPLPGDADFICGGPPCQGISGFNRFRNTKAPLDDPKNHQMVVYMDIVEYLKPKYVLMENVVDILRFAGGFLVRYALGRLISMNYQARLGMMAAGSYGLPQFRMRVFMWGSQPSESLPQYPLPTHEVVVKGGIPNEFEEITVAYNKLDPCQLEKALYLGDAILDLPPVNNDESQDERKYGTTPQSDFQKYIRLKKSDVVGFATDKNASHPQMLYDHRPLKLNDDDYQRVCRVPKKKGANFRDFPGVLVGTDNKVEWDPAVERVLLPSGKPLVPDYAMSFVRGTSCKPFGRLWWDETVATVVTRAEPHNQIVIHPMQDRVLSIRENARLQGFPDCYQLHGPVKERYTQVGNAVAVPVATALGYSFGIASQGFSDNKPLTTLPFKYPNCLQKSS

>*Rc*MET1

MVSMREVSESKEAPFSDNNKKTGAAEDTSNIQESKPENGDGAASKVKLKRGLSKNVENVYSSRKMTKRAAACSDFKEKPLDFSEEYSFTEIKKEVIVEHEAVALGLTAGRDDLRPNRRLKDFIFHDAYGNIQAVEMLEFFDLFISGLIVPLEESSDKDKEKGIRCNGFGRVESWAISGYEEGSPVIWVSTDMADYDCIKPANRYKKLYNLFFEKAHACVEVYRKLSRMHGGDPNISFDELIAGVVRAMSGSKNFPQGMSIKDFIISQGEFIYNQLIGLDATSKKNDQMLTGLPVLLAMRDEKKKREGLFMHEATTTGRTVNRNLTIDGKLTNQLLSSSLAEEDEDMKLASLRQEVENWLSMKQNKRRFSVPSSHFYIKINEDEIVNDYPWPTYYKTSAEEIDEFLVICDDEIFNPPENVLHNWTLYNSDARLISLELLPMRYCADADMTIFASGIVTKDGDLSGRYLDVNSDLPSSASSDVQDVGLPIYTSAIKEWKIEFGSSMIFISLRTDLSWYRLGKPSKQYAPWYKPVVKTARLAISIITLLQEQRRVSRLSFADVIQRVSKFNEGHHAFISSLAAEVERYVVVHGQIILQLFSEFPVKAIQNCPFVTDLHSKMQQINHLKLRVKEKRVLKKGLNLNPREAVATISERKAMAATTTKLINRIWGDYYSNYSPEESNGADGLEIKEDDEVVEEQEESEEEDSEEIEEQKMEIEKKSEKTRSTEKLNTWQCSNQEIRWVGGLLGKSCTGKAFYKKAFVHGGIVAVGDFVMLGTTDADQLSPIYFVEYMFENSDMRKMAHGKLMLYGSQTVLGNTANKRELFLANDCLEFELSDVKQNVVVEFKLTPWGYRHRKASIIKDRTDRERTEERRCKGLPMEYFCKSQYLPERGAFICLSKDTMGLGNGVCHSCKTKEIQMGKESFRLNLSSDSFVYKGTEYCIHDYVYRAPHHFTVDVMDLQTFKGGRNVRLRPYVVCQLLEIQVGKGLLQVTPESILVKVRRFFRSEDISAENAYCSDIREVYYSEHIETFPILDIQGKCEVRKKHDLPSFDKLAIFEHIFFCEHIYDPEKGTIKQLPAHIKLSSSKERMNDDILYRKKKGKCKEGENDFDDNVKRSEALSRNQLATLDIFAGCGGLSEGLERAGISVTKWAIEYEEPAGEAFKQNHPEALMLINNCNVILRAIMSACGDADDCICTSEASELAEKLDEKEISNFPRPGEVEFINGGPPCQGFSGMNRFNQRSWSKVQCEMILAFLSFADYFRPKFFLLENVRNFVSFNKGNTFRLTLASLLEMGYQVRFGVLEAGAFGISQSRKRAFIWAASPEEILPEWPEPMHVFAGPELKIKLSGNSQYAAVRSTANGAPFRAITVRDTIGDLPAVGNGASLTTMQYKNEPVSWFQKTIRGDALVLTDHISKEMNELNLIRCKRIPKHPGADWRDLPDKKVKLSNGQLVDLVPWCLPNTAKRHNQWKGLFGRLDWEGNFPTSVTDPQPMGKVGMCFHPEQDRILTVRECARSQGFADSYQFLGNIQQKHRQIGNAVPPPLAFALGRKLKEAVEMKRSLSVRCLCNYCTAIVVQNSLPSTYP

>*Rc*CMT2

MNKFYGNDLHYDQLLQGEGGQKHVGRIVEFFKTTDGEDYFRVQWFYRAEDTVIKEEAGFHDKKRLFYSTIMNDNPIDCIISKVRIVQLPSMIGSKSSIRSTDFYFDMEYNVDYSSFRTLLIGKHFCLRDNSSSPNVVEAVSTTGGNTSLENMRSSESNKAELALLDLFSGCGGMSTGLCLGAKVSCVDLVTRWALDSNKSACESLKLNHPETNVRNEAAEDFLELLKVWEKLCKRYAVKDIKSMQQSRSIASRVAEENDNSLSDNDITPGEYEVSKLVDLCYGDPDNIGKRGLKFKVHWKGYSTSEDTWEPVEGLRNCQECIRDFVRKGFKSKILPLPGDVDVICGGPPCQGISGYNRFRNVNAPLSDERNRQIVVFMDIVQFLKPKFVLMENVVDILKFDKASFARYALSRLVHMKYQARLGTIAAGCYGLPQFRLRVFLWGAHPSEKLPQFPLPTHDVIVRYWPPNEFERNTVAYDEGQPRELEKAAVLQDAISDLPPVTTHETREEMAYEKPPETEFQRFIRSTEHEMTGSSQRGTTQMNNLLSDHRPYALTEEDCARVCLIPKRKGANFRDLPGILVGNDNVVRRDPTKEQVLLPSGKPLVPDFAFTFEQGKSKRPYARLWWDETVPTVVTYPSIHSQAMLHPEQDRALTIRECARLQGFPDYYRFSGTVKERYRQIGNAVAVPVGRALGYALGMAFLKLSGDGPLMTLPPKFSHSTNLQLAKSLFQKTD

>*Rc*MET2

MKKNKGKPKSSVSKANKEQINNIKEKKRSSSQKNEEPAGSKKRPKRAAACTDFKEKAVRLSDKSSVLESRRDQFADDEILAVHLTHGQDDGRPNRRLTDFVVHDENGTPQPLEMIEVDDMFISGLILPLDENPDKEKEKRVRCEGFGRIEGWDISGYEDGFPVIWLTTDIADYNCLKPANNYKKFYDHFFEKARACIEVYKKLSRSSGGNPDLTLDELLAGVVRSMSGSKCFSGAASIKDFVISQGNFIYKQLLGLDETSKNNDQKFADLSALLALRDKSEEHGNFVLAKAVNTSGNLTIYQKFGDSVSNVNQSISSTAAGEDEDAKLARLLQEEEYWQTTKKQKKIHGSASSSNTIYIKINEDEIANDYPLPVFYKHSDEETDEYIAIDTEEHIMVDPDELPKRMLHNWSLYNSDSRLISLELLPMKPCEDIDVTIFGSGRMTEDDGSGFSLDDDPDQSSSAGSGAQDDVGLPIFLSAIKEWMIEFGSSMVFISIRTDMAWYRLGKPSKQYTSWYKPVLKTAKLARSIITLLKEQSRVSRLSFGDVIRRVSEFKKDDHGYISSDPATVERYVVVHGQIILQLFAEFPDEKIKKCAFVVGLTSKMEERHHTKWVVNKKQILQKNQPNLNPRAAMSSMAPVVSKRKAMQATTTRLINRIWGEYYSNYSPEDLKEATNCEAKEEDEVEEQEENEDDAEEEKLLLSDKTQKACSMSSRTKSYSKDEVLWDGNPVSKTHSGEAIYNSAIVRGEVIKVGAAVYLEVDESDELPAIYFVEYMFETSGGSKMFHGRVMQHGSGTILGNAANEREVFLTNECLNYELQDVKQAIAVEVRKMPWGYQHRNDNATADRIDRAKAEERKKKGLPLEYYCKSMYWPERGAFFSLPFDSMGLGSGICHSCKVKEVEMEKYIFRVNSSRTGFVHMGTEYSIHDFVYVSPCHFTIEREAETYKGGRNVGLKAYAVCQLLEIVVPKEPKQAEATSTQVKIRRFSRPEDISSEKAYCSDIREVYYTEETHLLSVETIEGKCEVRKKNDIPPCGSAAIFDHIFFCEHLYDPSKGSLKQLPAHIKLRYSTGTQESDAASRKRKGKCKEGEDEVENKREATQGRRLATLDIFSGCGGLSEGLQQAGVSSTKWAIEYEEPAGEAFKLNHPESLVFINNCNVILRAVMEKCGDTDDCISTSEAIELAASLDEKIINDLPLPGQVDFINGGPPCQGFSGMNRFSQSTWSKVQCEMILAFLSFADYFRPKYFLLENVRNFVSFNKGQTFRLALASLLEMGYQVRFGILEAGAYGVSHSRKRAFIWAASPEEVLPEWPEPMHVFSAPELKISLSGNSHYAAVRSTANGAPFRAITVRDTIGDLPVVGNGASATNMEYKNDPVSWFQKRIRGNMVTLTDHISKEMNELNLIRCQKIPKRPGADWRDLPDEKVKLSTGQLVDLIPWCLPNTAKRHNQWKGLFGRLDWEGNFPTSITDPQPMGKVGMCFHPEQDRILTVRECARSQGFRDSYKFAGNIQHKHRQIGNAVPPPLAYALGIKLKEALDGRLK

>*Rc*DRM3

MQLISFSNSNAVLSWLTAEASGHDKMRKVHNGETSSNQKTTVPKPEMLDFDFPSDFIYSSQLGETAASSSASDVRSFLTGMGFLPSLVDKVIQENGEDNVDLLLEILMECSDPQKSNSQSSDSLDTLFNDKDASNPPEYSKFGQPKEEPDVFGFDDSKRASLLHMNFSAEEVDFAMEKLGTDAPVDEIVDFITAAQVAANFDEERDDELEHDVEQNKYCSRHSRMNPSHVARGGESFNMEIRDEQRRDSYGRKKPENLSQEAVSRSSNLNLEEIHRGKRPKQEYTEGYSNTQDEHFGFEENYEEEGPKPEYTADSSFSFESAWVEEKVDTEITGFRIPREPKFKSCKSVDRMVAKPPYFLYGNVATVSLDTWGKISQFLYAVEPEFVNTQFFSALSRKEGYVHNLPTENRFHILPKPPMSILDAMPHIKKWWPSWDTRKQLSFINYETNGLSQLCDRLGRMLTDSRGLISSQQKRDILHHCHKLNLVWVGLHKVRPIEPEHLELILGYPLNHTQTTEGSLTERLHALRYSFQIDTLGYHLSVLKSMFPEGITVLSLFSGIGGAEVALHRLGIHMKGVVSVETSETKRKILRMWWRNSGQTGELEQIEDIQKLTTKKIDRLIERFGGFDFVICQSPCTCASGNPKMEQRADARSALDFSLFCEFVRVLQRVRTQVQRKR

>*Rc*DRM2

MGVIVYCFRITTCKPVYMEHKTLYLEILGIAVASKEMDGDSFCGEGDNFDWDSEDEREIENFGLTNSSCLAVPAVEATARSVEKCLMGFSCILRHASSSVRSSGTKVMDHFVGMGFPEKMVAEAIQENGEENADLILETLLKFSTISSPSSSGSKLIDHFVGMGFDVEMVDRAIQENGEEKTDSILETLLTYSVIEKSHQEQPHVDSDHWSSEYEGSFLNDFSDIDSSENEENGKSQPVEGNTLRFLARMGYTTDEASIALERCGPDANIAELTDFICAAQMAKAADASFPEEKPKIRHFDDDYPKNKKRNYAEYDMWKKKKQMKLDKKLLNGDDELIRLPNPMLGFGVPTDPAIVTHRKLPEAALGPPFFYYENVALAPKGVWQTISRFLYDVEPEFVDSKYFCAAARKRGYVHNLPIQNRYPLLPLPPNNIHEALPLTKRWWPSWDTRTKLNCLQTCVASAKLTDRIRKALEDYEGEPPLSIRKYVLDECRKWNLLWVGRNKLAPLEPDEVEMLLGFPRNHTRGGGISRTDRYKSLGNSFQVDTVAYHLSVLKDMFPEGISLLSLFSGIGGAEVALHRLGIRLKRVVSVEISEVNRNIMRCWWEQTNQTGTLIDIADVHDLNADRLEQLMSSFGGFDLVVGGSPCNNLAGSNRHHRDGLEGKESSLFFDYCRILDLVKCIMTRN

>*Rc*DRM1

MDRFLSLVNVNVLLKHVTLKRQNSILRSQANSLTGCSSSRMVHQFVNMGFSEKLVMQAIDENGEDEASILDSLLTYSLRKEDFPCKKRKLHQNRSSKSQESKRKAKNFLERNGRTLHLPNPMIGFGIPHEPWKITERKLHPIAVGPPYFYYENAALTPKGVWSKISSFLYDIQPEFVDSIYFCAAARKRGYVHNLPIQNRFPLLPRPPLTIHEVLPSTEKWWPSWDKRTKLNCLLTSYGNAKLTECIRKALEDCDGEPTLRAKKYVLEQCKRWNLVWVGKNKVAPLEPDEMEMLLGFPKDHTRGGGINRNDRYKALGNSFQVDTVAYHLSVLKNLFPYGITVLSLFSGIGGAEVALHRLGIPLNSVVSVEISEVNRNIPRSWWEQTNQKGYLIEIGDVREVDGDSIKRWIKLFGGFDLVIGGSPCNNLAGANRRNRDGLEGEQSSLFYDYYRILKLVRDTFGEIMDSEILNRILSYPDL

>*Pt*CMT3

MPRKRQRSTASKPETSSSSPPKTRKMMSTTTAPKQSRLNEKKAEEEQSTASTRSRLKEKKAEKEQSKAAARSCLKEKKAAEEQLTTATRSRLMEKEAEEEQSTAPNRSRLREKKVKKEETEEVFLDAEEDDTNSIDEVGAKMGTDNGDSRSETKEKEKEKRGSGKTKKISTPEKAKEEDGTPARFVGNQVPDAEARKKWPHRYANKIKNKTPISKPSNSLDDSEEIIKARCHYKRAEVDGIIYNLYDDAHVQASDGEADYICRIIEMFESEDRTPHFTAQWYYRSTDTIIKDKYISDPKCVFFSEIRNDNPLDCLTRKLKIVRLALGVDSETRRAKTLNCDFYCDMLYLLPYSTFVKLPSENNTTAPESSSTISSDIDAAGVKFECDEVCESSGRRKSEVALLDLYSGCGAMSTGLCLGANLSGLNLVTKWAVDLNKHACESLRLNHPETQVRNETAEDFLMLLKEWEKLCIRFSLVKNDDPEKQQTYSFDMDDEDDDDDEEEEDDDNNDVSDNNDDSEVFEVEKILEVCHGDPKEIGGQRDLYFKVSWKNYGPDYDTWEPISGLSNCREAIKKFVMHGYKSNILPLPGDVEVICGGPPCQGISGFNRFRNVKNPLEDPKNKQLVVFMDIVDFLKPKFVLMENVVDLLKFADGFLGRYAMGCLVSMKYQARLGMLAAGAYGLPQFRMRVFLWGACATEKLPQYPLPTHDVLVRGVVPLEFEGNTVAYEEGVKPQLERKLFLEDAISDLPAVANDEKRDEMPYGESPKTEFQRMIRLKKMGLELNDLLFDHRPLELNDDDYQRVCQIPKRKGGNFRDLPGVRVRPDKKVEWDPEVPRQYLSSGKPLVPDYAMTFVNGSSSKPFARLWWDETVPTVVTRAEPHNQAIMHPEQDRVLTIRENARLQGFPDYYQLCGPIKERYIQVGNAVAVPVARALGYALGRAFQGFAGDDPVFSLPKKFPRITEDPSSSSEAQC

>*Pt*CMT2

MKSSSSPTDSRKLTPLQVYESTSTSRRSPRLAPPNNNVPSKTPKSNKPKTTHISLRRSPRLEPTPASPQSVSRKKHSRNVSKTKCSARNVSVDRKIPSRSSRVGNSRMQNVAANRTVLLDSSALRRSPRLSSADMEIVVSERNASVVVADTARLRRSPRLANGYAEVAVSKVRTFLKRKRVSDEMSRSPRNQNGRLDVQFALVQSEKCSRFIVNEENERNCLKVECSSKDLSKGSPVACSSANAKLGCCEKRGCQSVLLTISDSEVGDSGEFEAKPLLLPWHGDNESQTGMVKCDDGSSVFEEEPLRMSPSFDFANENAVINESPIKSCKWSSALDGDGNFEVISCSSGIFDSNDACPSKRVKICEENSVSGMSDEKLLRRSPRTNSIVVTENGENKSGKKQSPKTNSGKELSLEKASSGKKQKEHRGNCSLIGDPVAHDEAQERWHWRYEMKSKRTKHPRLALDDDDEDKVVWNVECHYTQANIEGRIINLGDCVYVKGEGAKNHIGSILEFFKTTDREDYFRVQWFYRAEDTVMKEAADFHDNKRLFYSTVMNDNPIDCIISKVTVVQISPRVHLKFHSTPASDFYFDMEYCVDYSTFRTLLTDCSLRGHELSPLPFCDSRSATPSDISMENMSTCGSYKAKLTLLDLFSGCGGMSTGLCLGAKVSCVDLVTRWALDSDESACQSLKLNHPETHVRNEAAEDFLELLKEWQKLCKRYAVNDVGRTHKSRSMASSMSKQNKNSSNDDDIASGEYEVARLVDICYGKTDKRGKRGLKFKVHWKGYSTSEDSWEPIEGLSNCEHSIRDFVREGFKSKILPLPGDADVICGGPPCQGISGYNRYRNVDSPLADERNIQIVVFMDIVQFLKPKYVLMENVVDILRFDKASFARYALSRLVHMKYQARLGTVAAGCYGLPQFRLRVFLWGAHPKEKLPQFPLPSHDVIVRYWPPPEFERNTVAYDEDQPRDDLEKATVLRDAISDLPDVTSHETREEMAYDKPPETDFQQFIRSTRNEMTGSELSGTRMINLLYDHRPYSLTEEDFARVCQIPKKKGANFRDLPGVVVGADNVARRDPTEEQMLLPSGKPLVPDFALNFEGGKSRRPYARLWWDETVSTVVTFPDLHSQAVMHPEQDRVLTIRECARLQGFPDYYRFCGTVKQRYRQIGNAVAVPVGRALGFTLGMAFQKLSGDDPLMTLPPKFSHSTNLQLAKSLFQKAE

>*Pt*MET1

MRKNKKGKQKSSVSNAKKEVPEKTAKGKKRNFSDTNKEDPAGGSLTRPRRAAACKDFKEKSLRLHEEKSSVVESKKEQVVNEEILALRLTQGQEEGRPNRRLIDFVVHDANGNPQPLEMIEVDDMFISGVIMPLEESLDKEKEVPVRCEGFGRIEAWNISGYEDGSPVIWLTTEVADYDCIKPSGGYKKFFDRFFQKALACIEVYKKLSRFSGGNPEFTLDELLAGVVRAMSGNKCFSGAPSVKNFLVSQGEFIYQQITGLDQTSKKNDKFFSDLPALVALRDESRNHGSVLLAKAANPGGNLVIDPKSVDGAIVNQSNQSSTIAEEDEDAKLARLLQEEEYWHSNMRQKKSRGSASASNTIYIKINEDEIANDYPLPVFYKHSDEETDEYVVVASDDVIDHPDDLPRKMLHNWSLYNSDSRLISLELLPMKPCEDIDVTIFGSGRMTEDDGSGFCLDDDPDQSSSRGSEAQDDMGLPIFLSAIKEWMIEFGSSMIFISIRTDMAWYRLGKPSKQYGSWYKPVLKTVKLARSIITLLKEQSRVSRLSFADVIRKVSEFKKDHHAYISSDPAAIERYVVVHGQIILQLFAEFPDQKIKKCAFVVGLTRKMEERHHTKWVVNKKAIVQKFQSNLNPRAAMDTVAPGSKRKLMQATTTRLINRIWGEYYSNYSPEDLEEGAECEVKEEDEAEEQYENEDDDKEEVVEKTLKPRSVSERTKSHTSQKEVRWDGNPVSKTSSGEAIYKRAIVCGEVIVVGDAVLVEVDESDELPAIYFVEYMFETRNGSRMFHGRMMKRGSETVLGNTANDREVFLTTECMNYKLQDAKQAIILEVLKRPWGHDHRKDNINADRIDREKAEERKKKGLQVEYYCKSLYWPERGAFFTLPLDTMGLGSGVCHSCNLKIAEEDKDIFRVNSSQTGFSYKGTEYSVHDFVYVSPHQFASERGENETFKGGRNVGLKPYVVCQLLEVVLKEPKQAETRSTQVNVQRFFRPDDISPEKAYCSDIREIYYSEETHLLSVETIEGKCEVRKKNDIPTCSAPAIFDNIFFCEHMYDPSKGSLKQLPAQVKSKFSAVSRDGDVASRKRKGKSKEGENDIEADKQREASPENRLATLDIFAGCGGLSEGLQQAGVSSTKWAIEYEEPAGEAFKLNHAGSLMFINNCNVILRAVMEKCGDADDCISTSEAGELASSLDAKVIDGLPLPGQVDFINGGPPCQGFSGMNRFNQSTWSKVQCEMILAFLSFADYFRPKYFLLENVRNFVSFNKGQTFRLTIASLLQMGYQVRFGILEAGAYGVSQSRKRAFIWAASPEEILPEWPEPMHVFAAPELKITLSEKSQYSAVRSTAYGAPFRAITVRDTIGDLPDVGNGASKTNLEYGNDPVSWFQKKIRGDMVVLTDHISKEMNELNLIRCKKIPKRPGADWRDLPDEKVKLSTGQMVDLIPWCLPNTAKRHNQWKGLFGRLDWEGNFPTSITDPQPMGKVGMCFHPEQDRILTVRECARSQGFPDSYQFSGNIHHKHRQIGNAVPPPLSYALGRKLKEALDSKRRK

>*Pt*MET2

MGSSSILDTTTNDPVDNTISSSSSVGMRKNKKGKQKSSVSNVKKEVPEKNTKGKKRNSPDTNKEEPTGGDGSLKRPKRAAACKDFKEKSVRLHEEKSYVVESKKEQVVDEEILAVRLTQGQEEGRPNRRLIDFVVHDANGNPQPLEMVEVDDMFISGIIMPHEESLDKEKEVHVRCDGFGRIEAWDISGYEDGSPVIWLSTEVADYDCIKPAGGYKKFFDHFFQKALACVEVYKKLSRFSGGNPEFTLDELLAGVVRAMSGNKCFSGAVSIKNFLISQGEFIYHQIIGLDETSTKNDKKFADLPVLVALRDESRNHGNVLIAKAANSGGNLVIGPESVDGAVVNQSNQSSTTVEEDEDAKLARLLQDEEYWQSNMRQKKSRGSVSASNTIYIKINEDEIANDYPLPAFYKHSNEETDEYIAVASDDVIDHPDDLPRRMLHNWSLYNSDSRLISLELLPMKPCEDIDVTIFGSGSMTEDDGSGFCLDDGPDQSSSRGLEAQDDMGLPIFLSAIKEWMIEFGSSMIFISLRTDMAWYRLGKPSKQYASWYKPVLKTVKLARTIITLLKEQSRVSRLSFADVIRKVSEFKKDHHAYISSDLAAVERYVVVHGQIILQLFAEFPDQKIKKCAFVVGLTRMMEERHHTKWVVNKKAIVQKCHSNLNPRAAMDTVASGASKRKLMQATTTRLINRIWGEYYSNYSPEDLKEGNDCDVKEEDELEEQDENEDDDKEVVVEKTLKPYSVFEHCKSHTSQKEVRWDGNPVRKISSGEDIYKQAIVCGQVIVVGAAVLVEVDEPDELPAIYFVEYMFETRNGSKMFHGRMMKWGSETVLGNTANDREVFLTNECMNYKLQDVKQTIILEVRKRPWGHHHRKDNANADRIDREKAEERKKKGLPLEYYCKSLYWPERGAFFTLPFDTMGLGSGVCHSCNLKISEEDKNISKVNSSQTGFSYKGTEYSVHDFVYVSPHQFAVESGETETFKGGRNVGLKPYAVCQLLEVVPMETKQSETRSTEVKVQRFFRPDDISPEKAYCSDIREIYYSEETHLLSVEVIEGKCEVRKKIDIPTCSAPAIFDHTFFCEHMYDPSNGSLKQLPAHIKSKFSAVSKDGDVASRKRKGKSKEGENDTEDDKQLEASPEYRLATLDIFAGCGGLSEGLQQAGVSTTKWAIEYEEPAGEAFKLNHAESLMFINNCNVILRAVMERCGDADDCISTSEAAKMASSLDAKVIDGLPLPGQVDFINGGPPCQGFSGMNRFNQSTWSKVQCEMILAFLSFADYFRPKYFLLENVRNFVSFNKGQTFRLTIASLLQMGYQVRFGILEAGAYGVSQSRKRAFIWAASPEEILPEWPEPMHVFAAPELKITLSEKSQYAAVRSTAYGAPFRAITVRDTIGDLPDVANGASKTNLEYGNDPISWFQKKIRGDMVVLTDHISKEMNELNLIRCKNIPKRPGADWRDLPDEKVKLSTGQMVDLIPWCLPNTAKRHNQWKGLFGRLDWEGNFPTSITDPQPMGKVGMCFHPEQDRILTVRECARSQGFPDNYQFFGNIQHKHRQIGNAVPPPLAYALGRKLKEALDSKRQK

>*Pt*MET3

MNVGMRKNKKGKQKSSVSNVKKEVPEKNTKGKKRNSPDTNKEEPTGGDGSLKRPKRAAACKDFKEKSVRLHEEKSYVVESKKEQVVDEEILAVRLTQGQEEGRPNRRLIDFVVHDANGNPQPLEMVEVDDMFISGIIMPHEESIDKEKEVHVRCDGFGRIEAWDISGYEDGSPVIWLSTEVADYDCIKPAGGYKKFFDHFFQKALACVEVYKKLSRFSGGNPEFTLDELLAGVVRAMSGNKCFSGAVSIKNFLISQGEFIYHQIIGLDETSTKNDKKFADLPVLVALRDESRNHGNVLIAKAANSGGNLVIGPESVDGAVVNQSNQSSTTVEEDEDAKLARLLQEEEYWQSNMRQKKSRGSVSASNTIYIKINEDEIANDYPLPVFYKHSNEETDEYIAVASDDVIDHPDDLPRRMLHNWSLYNSDSRLISLELLPMKPCEDIDVTIFGSGSMTEDDGSGFCLDDGPDQSSSRGLEAQDDMGLPIFLSAIKEWMIEFGSSMIFISLRTDMAWYRLGKPSKQYASWYKPVLKTVKLARSIITLLKEQSRVSRLSFADVIRKVSEFKKDHHAYISSDLAAVERYVVVHGQIILQLFAEFPDQKIKKCAFVVGLTRMMEERHHTKWVVNKKAIVQKCHSNLNPRAAMDTVASGTSKRKLMQATTTRLINRIWGEYYSNYSPEDLKEGNDCDVKEEDELEEQDENEDDDKEVVVEKTLKPYSVFEHCKSHTSQKEVRWDGNPVRKTSSGEDIYKQAIVCGQVIVVGAAVLVEVDEPDELPAIYFVEYMFETRNGSKMFHGRMMKWGSETVLGNTANDREVFLTNECMNYKLQDVKQAIILEVRKRPWGHHHRKDNANADRIDREKAEERKKKGLPLEYYCKSLYWPERGAFFTLPFDTMGLGSGVCHSCNLKISEEDKNISKVNSSQTGFSYKGTEYSVHDFVYVNPHQFAVESGETETFKGGRNVGLKPYAVCQLLEVVPMETKQSETRSTEVKVQRFFRPDDISPEKAYCSDIREIYYSEETHLLSVEVIEGKCEVRKKIDIPTCSAPAIFDHTFFCEHMYDPSNGSLKQLPAHIKSKFSAVSKDGDVASRKRKGKSKEGENDTEDDKQLEASPEYRLATLDIFAGCGGLSEGLQQAGVSTTKWAIEYEEPAGEAFKLNHAESLMFINNCNVILRAVMERCGDADDCISTSEAAKLASSLDAKVINGLPLPGQVDFINGGPPCQGFSGMNRFNQSTWSKVQCEMILAFLSFADYFRPKYFLLENVRNFVSFNKGQTFRLTIASLLQMGYQVRFGILEAGAYGVSQSRKRAFIWAASPEEILPEWPEPMHVFAAPELKITLSEKSQYAAVRSTAYGAPFRAITVRDTIGDLPDVANGASKTNLEYGNDPISWFQKKIRGDMVVLTDHISKEMNELNLIRCKNIPKRPGADWRDLPDEKVKLSTGQMVDLIPWCLPNTAKRHNQWKGLFGRLDWEGNFPTSITDPQPMGKVGMCFHPEQDRILTVRECARSQGFPDNYQFFGNIQHKHRQIGNAVPPPLAYALGRKLKEALDSKRQK

>*Pt*DRM2

MEDPNRSRSSSDWNNNNKNLIKPKHEKLDFDLAFESLRSREVGDNVASSSRSYLRSFFTEMGYSPSLVNRVIEENGEDNVDLLLEILMECSGLQKPNSQSSDSLDCLFDDRGESSPPKYSTVTGVKEEPDVFDEVYDDKRVSLLKMNFPAKEVELAMDKLGENAPINEIIDFIIAAQIANNLDRETEDMPDIDAENKEDVNDETLYGTMDKTLCLLNMGFSENEVSLAIDKFGSEVPVTELANAICAHQLGETYVIKKKYSENSTASCSSAAEDSRSFGVETENNTRHHSFSWVKSETEDCRRDAILQSRDMNTKETRKGKRPRQEHIEGYQEAQPRHDSLEENCAGEQPKQEYDYGSSSYFEHEWVEEKVNSDTTTFGMPKPFKCNPCKILDQIAAKPPYFFYGNVATASSDTWGKISQFLYGIEPEFVDTQFFSALSRREGYIHNLPTENRSHILPKPPITLEDLMPSTKKWWPSWDARKKMSCRNFDSSGSSQLCDMLGRMLDDSRGLLSAEQQRDLLRHCQALNLMWVGPNKLSPLESAHLEKILGYPLNHTLIADYPLTERMYSLRYSFQTDTLGYHLSVLKSIFPQGITVLSLFSGIGGAEITLHRLGIHLKGVVSVETSETNRRVLKRWWYSSGQTGRLEQIEDIRKLTSSTVERLVENFVCFDFVICQNSFTRPSKIPGVGSGLESQHFFDFTLFNEFVRVLQRVRSAIERKR

>*Pt*DRM1

MDGDSLCGDNDDFDWDSEDEKEIENFASSSSSSLRLPQVETRSSSAEASSSVGSSSGSKMIDHFVKMGFPEKMVAEAIQENCKGEEDEDSILETLLKYSTSSSASSSGSKLFDRFVGMGFAEKMVAKAIKENGEGDADSVLETLLTYAAIGKSPQEQPNNDSDHCSSGHEGSFLDDFSDVDSADDEVITKTVSDEDNKLAFLRRMGYKEADASIAITRCGTEATISELADFICAAQIAKAEDAFFAEDEKKPKHLDKQKKRSFLESDVLEKKRQKGLENGDDEGVRLPNPMVGFGVPTEPGIVTRRTLSEAAIGPPFFYYENVALAPKGVWQTISRFLYDVEPEFVDSKHFCAAARKRGYVHNLPIHNRFPLLPLPPNTIHEALPLTRKWWPAWDERTKLNCLQTCIASAKLTERIRKALEAYEGEPPLHVQKFIMDECRKWNLVWVGRNKVAPLEADEVEMLLGFPRNHTRGGGISRTDRYKSLGNSFQVDTVAYHLSSLKDLFPGGINVLSLFSGIGGAEVALHRLGIRLKNVVSVEISNVNRSIMSCWWEQTNQTGNLIHIEDVQHLTADRLEQLMNMYGSFDLVVGGSPCNNLAGSNRHHRDGLEGKESSLFFDYCRILDVVKNLTSRYS

>ZmMET1a

MLTNDFSGTRRCRAKPQKKEEESTENNKLENGSLDATEEVHHGVEKGDGHVTRKRPRRSAACSDFKEKSIRLSEKKSVVMVKKNRMEEEEVDAVNLTKLGPEDPPPCRKLIDFILHDAEGNPQPFEMSEIDDFFITALIMPMDDDLEKERERGVRCEGFGRIEDWNISGYDEGTPVIWVSTDVADYECVKPSTNYKSYFDHFYEKAQVCVEVFKKLAKSVGGNPNQGLDELLASVVRSTNAMKGYSGTMSKDLVISIGEFVYNQLVGLDETSNNDDEKFATLPVLLSLRDQCRSRVELTKLPSNFSNTSLKIKDSECDETAEDDDDAKLARLLQQEEEWKMMKKQRGRRGTPSQKNVYIKISEAEIANDYPLPAYYKPFSQEMDEYIFDSDDSIFSDDVPVRILNNWTLYNADSRLISLELIPMKSGAENDVVIFGSGFMRDDDGSCCSTAESVKSSSSSSKADQLDAGIPIYLSPIKEWIIEFGGSMICITIRTDVAWYKLRQPTKQYAPWCEPVLKTARLAVSIITLLKEQSRASKLSFADVIRKVAEFDKGNPAFISSNITLVERYIVVHGQIILQQFADFPDETIRRSAFVSGLLLKMEQRRHTKLVMKKKTQVMRGENLNPSAAMGPASRKKAMRATTTRLINRIWSDYYAHFPEDSKEGDGNETKEIDDEQEENEDEDAEDEGQIEENISKTPPSTRSRKLLSQTCKEIRWEGETSGKTLSGETLYKCAYVRELRIPVGGTVALEDDSGDTVICFVEYMFQKVDGSKMVHGRILQKGSQTILGNAANEREVFLTNDCLEFKLDDIKELVMVDIQSRPWGHKYRKENSEADKVEQVKAEERKKKGQPMVYFCKSLYWPEKGAFFALSRDKMGLGSGLCSSCDNIEPDSDELKIFSKTSFVYRKVTYNVNEFLYIRPDFFAEDEDRATFKAGRNVGLKPYAVCQILSIPEGAGSKKLNPASANISARRFYRPDDISSAKAYASDIREVYYSEDVIDVPVDMIEGKCEVRKKNDLASSDLPVMFEHVFFCELIYDRASGALKQLPPNVRFMSMVQRTSALKKNKGKQICEPDQIDSGKWLDVPKENRLATLDIFAGCGGLSEGLQQAGVSFTKWAIEYEEPAGEAFNKNHPEAVVFVDNCNVILKAIMDKCGDTDDCVSTSEAAEQAAKLPEVNINNLPVPGEVEFINGGPPCQGFSGMNRFNQSPWSKVQCEMILAFLSFAEYFRPRFFLLENVRNFVSFNKGQTFRLAVASLLEMGYQVRFGILEAGAFGVAQSRKRAFIWAAAPGEMLPDWPEPMHVFASPELKITLPDGQYYAAARSTAGGAPFRAITVRDTIGDLPKVGNGASKLTLEYGGEPVSWFQKKIRGNMMVLNDHISKEMNELNLIRCQHIPKRPGCDWHDLPDEKVKLSNGQMADLIPWCLPNTAKRHNQWKGLYGRLDWEGNFPTSVTDPQPMGKVGMCFHPDQDRIITVRECARSQGFPDSYEFAGNIQNKHRQIGNAVPPPLAYALGRKLKEAVDKRQEASAGVPAP*

>ZmMET1b

MLTNDFSGTRRCRAKPQKKEEESTENNKLENGPLDATEEVHHGVEKGDGHVTRKRPRRAAACSDFKEKSIRLSEKKSVVMVKKNRMEEEEVDAVNLTKLGPEDPPPCRKLIDFILHDAEGNPQPFEMSEIDDFFITALIMPMDDDLEKEREKGVRCEGFGRIEDWNISGYDEGTPVIWVSTDVADYECVKPSTNYKSYFDHFYEKAQVCVEVFKKLAKSVGGNPNQGLDELLASVVRSTNAMKGYSGTMSKDLVISIGEFVYNQLVGLDETSNNDDEKFATLPVLLSLRDQCRSRVELTKLPSNFSNTSLKIKDSECDETAEDDDDAKLARLLQQEEEWKMMKKQRGRRGTPSQKNVYIKISEAEIANDYPLPAYYKPFSQEMDEYIFDSDDSIFSDDVPVRILNNWTLYNADSRLISLELIPMKSGAENDVVVFGSGFMRDDDGSCCSTAESVKSSSSSSKADQLDAGIPIYLSPIKEWIIEFGGSMICITIRTDVAWYKLRQPTKQYAPWCEPVLKTARLAVSIITLLKEQSRASKLSFADVIRKVAEFDKGNPAFISSNITLVERYIVVHGQIILQQFADFPDETIRRSAFVSGLLLKMEQRRHTKLVMKKKTQVMRGENLNPSAAMGPASRKKAMRATTTRLINRIWSDYYAHHFPEDSKEGDGNEIKEIDDEQEENEDEDAEDEGQIEENISKTPPSTRSRKLLSQTCKEIRWEGETSGKTLSGETLYKCAYVRELRIPVGGTVALEDDSGDTVMCFVEYMFQKVDGSKMVHGRILQKGSQTILDNAANEREVFLTNDCLEFKLDDIKELVMVDIQSRPWGHKYRKENSEADKVEQVKAEERKKKGQPMVYFCKSLYWPEKGAFFALSRDKMGLGSGLCSSCDNIEPDSDELKIFSKTSFVYRKVTYNVNEFLYISPDFFAEDEDRATFKAGRNVGLKPYAVCQILSIPEGAGSKKLNPASANISARRFYRPDDISSAKAYASDIREVYYSEDVIDVPVDMIEGKCEVRKKNDLASSDLPVMFEHVFFCELIYDRASGALKQLPPNVRFMSMVQRTSALKKNKGKQICEPDQIDSGKWLDVPKENRLATLDIFAGCGGLSEGLQQAGVSFTKWAIEYEEPAGEAFNKNHPEAVVFVDNCNVILKAIMDKCGDTDDCVSTSEAAEQAAKLPEVNINNLPVPGEVEFINGGPPCQGFSGMNRFNQSPWSKVQCEMILAFLSFAEYFRPRFFLLENVRNFVSFNKGQTFRLAVASLLEMGYQVRFGILEAGAFGVAQSRKRAFIWAAAPGEMLPDWPEPMHVFASPELKITLPDGQYYAAARSTAGGAPFRAITVRDTVGDLPKVGNGASKLTLEYGGEPVSWFQKKIRGNMMVLNDHISKEMNELNLIRCQHIPKRPGCDWHDLPDEKVKLSNGQMADLIPWCLPNTAKRHNQWKGLYGRLDWEGNFPTSVTDPQPMGKVGMCFHPDQDRIITVRECARSQGFPDSYEFAGNIQNKHRQIGNAVPPPLAYALGRKLKEAVDKRQEASAGVPAP*

>ZmMET2a

MAPSSPSPAAPTRVSGRKRAAKAEEIHQNKEEEEEEEEVVAASSAKRSRKAASSGKKPKSPPKQAKPGRKKKGDAEMKEPVEDDVCAEEPDEEELAMGEEEAEEQAMREEVVAVAAGSPGKKRVGRRNAAAAAGDHEPEFIGSPVAADEARSNWPKRYGRSTAAKKPDEEEELKARCHYRSAKVDNVVYCLGDDVYVKAGENEADYIGRITEFFEGTDQCHYFTCRWFFRAEDTVINSLVSISVDGHKHDPRRVFLSEEKNDNVLDCIISKVKIVHVDPNMDPKAKAQLIESCDLYYDMSYSVAYSTFANISSENGQSDSDTASGISSDDVDLETSSSMPTRTATLLDLYSGCGGMSTGLCLGAALSGLKLETRWAVDFNSFA
CQSLKYNHPQTEVRNEKADEFLALLKEWAVLCKKYVQDVDSNLASSEDQADEDSPLDKDEFVVEKLVGICYGGSDRENGIYFKVQWEGYGPEEDTWEPIDNLSDCPQKIRDFVQEGHKRKILPLPGDVDVICGGPPCQGISGFNRYRNRDEPLKDEKNKQMVTFMDIVAYLKPKYVLMENVVDILKFADGYLGKYALSCLVAMKYQARLGMMVAGCYGLPQFRMRVFLWGALSSMVLPKYPLPTYDVVVRGGAPNAFSQCMVAYDETQKPSLKKALLLGDAISDLPKVQNHQPNDVMEYGGSPKTEFQRYIRLSRKDMLDWSFGEGAGPDEGKLLDHQPLRLNNDDYERVQQIPVKKGANFRDLKGVRVGANNIVEWDPEIERVKLSSGKPLVPDYAMSFIKGKSLKPFGRLWWDETVPTVVTRAEPHNQVIIHPTQARVLTIRENARLQGFPDYYRLFGPIKEKYIQVGNAVAVPVARALGYCLGQAYLGESEGSDPLYQLPPSFTSVGGRTAGQARASPVGTPAGEVVEQ*

>ZmMET2b

MAPSSPSSARPTRASGRKRSAMAEEIHQNQEEEEEVVAASTAKRRRKAASSGKKPKPTPKQAKPAVAGMKKKGETEKTEPVVDDVCAEEPDEEELAMGEEEAEAEEQAMQEVVAAVAAGSPGKKRVGRRSAAASGDHVPEFIGSPVAAAEAHSNWPKRYERSTAANKPEEDDELKARCHYRSAKVDNIVYCLGDDVYVKAGENEADYIGRITEFFEGTDRCHYFTCRWFFRAEDTVINSLVSINVDGHKHDPRRVFLSEEKNDNVLDCIISKVKIVHVDPNMDPKAKAQLIEHCDLYYDMSYSVAYSTFANISSENGQSGSETASGISSDDAGLETSSNMPERTATLLDLYSGCGGMSTGLCLGAALSGLKLETRWAVDLNSFACQSLKYNHPQTEVRNEKADEFLALLKEWAVLCEKYVHQDVDSNLAGSEDQEDADTLDKDEFVVQKLIGIRYDGTGRKKGVYFKVQWEGYGPEEDTWEPI
DNLSDCPLKIREFVQEGRKRKILPLPGDVDVICGGPPCQGISGFNRFRNRDEPLKDEKNKQMVTFMDIVAYLKPKYVLMENVVDILKFADGYLGKYALSCLVAMKYQARLGMMVAGCYGLPQFRMRVFLWGALSSMVLPKYPLPTYDVVVRGGAPNAFSQCMVAYDETQRPSLKKALLLGDAFSDLPKVENHQPNDVMEYGGSPKTEFQRYIRLGRKDMLDWSFGEEAGPDEGKLLDHQPLRLNNDDYERVKQIPVKKGANFRDLKGVKVGANNVVEWDPEVERVYLSSGKPLVPDYAMSFIKGKSLKPFGRLWWDETVPTVVTRAEPHNQVILHPTQARVLTIRENARLQGFPDYYRLFGPIKEKYIQVGNAVAVPVARALGYCLGQAYLGESDGSQPLYQLPASFTSVGRTAVQANAVSVGTPAGEVVEQ*

>ZmMET3a

MAHWVSDSDGSDNFEWDSDGNGEEPANFNAAGAGSSALRSTNTDAPGPSTRVANGNGKAGPSASLVQKYMDMGFTEEIVRKAMKDNGDNGADSLVELLLTYQELGNDLKVDNGFASGCAPQTVDDSDDDDILENWDDEDADGGRSTRVANSIDDSEDEDFLNEMSQKDKKVDSLVKMGFPEDEAALAITRCGQDASISVLVDSIYASQTAGDGYCGNLSDYEGNSNGGRNKGRFMDGNKKKRKRYGGQAQGNRGPLDGSCEEPMPLPNPMVGFNLPDHWTRPVDRSLPTQAIGPPYFYYENVALAPKGVWTTISRFLYDIQPEFVDSKYFCAAARKRGYIHNLPLERRSPLLPLPPKTIFEAFPRTKRWWPSWDPRRQFNCLQTCTSSAKLLERIRVTLANSSDPPPPLVQKFVLEECRKWNLAWVGLNKVAPLEPDEMEFLLGFPKDHTRGISRTERYRSLGNSFQVDTVAYHLSVLKDRYPQGMNVLSLFTGIGGAEVALHRLGIRMNTVVSVEKSEVNRTILKSWWDQTQTGTLIEINDVQTLTADRIEAYIRRIGGFDLVIGGSPCNNLAGSNRHHRDGLEGEHSSLFYHYFRILDSVKSIMERL*

>ZmMET3b

MVHWVSDSDGSDNFEWDSDGNGEQTVSFNAAGAGSSALAATNTDAPGPSTRVANGNGKAGRSASLVQKYVDMGFSEEIVLKAMKDNGDNGADSLVELLLTYQELGNDLKVDNDFASSCAPKTADDSDDDDTLEIWDDEDAGGRSTRVANSVDDSDDEDFLHEMSRKDEKVDSLVKMGFPEDEAALAITRCGPDASISVLVDSIYASQTAGDGYCGNLSDYEDNSYGGRSTGNKKKRKRYGGQAQGSRGPLDGSCDEPMPLPHPMVGFNLPDQWSRRVDRSLPAQAIGPPYFYYENVALAPKGVWTTISRFLYDIQPEFVDSKYFCAAARKRGYIHNLPLENRSPLLPIPPKTISEAFPRTKRWWPSWDPRRQFNCLQTCVSSAKLLERIRVALTNSSDPPPPRVQKYVLEECRKWNLAWVGLNKVAPLEPDEMEFLLGFPKDHTRGISRTERYRSLGNSFQVDTVAYHLSVLKDLFPQGMNVLSLFSGIGGAEVALHRLGIQMNTVISVEKSEVNRTILKSWWDQTQTGTLIEITDVQTLSSERIEAYIRRIGGFDLVIGGSPCNNLTGSNRHHRDGLEGEHSALFHHYFRILHAVKSIMERL*

>ZmMET3c

MEKTLSLLQMGFTEEEVSSAIDNFDQRATVQELADSILARRIANSIEQKEIKVESDFLDEAETDYSSYQPSYSDVSYYDDDNN
NTRVKRAKHIFIDDIGASSSHLGDPWSMGQRAGTNDMSVKVELEAMTPGRRANVQGDLAKPPFFLYGNVVEVPKDTWHQLKQFLYNVEPEFVNSQSFSALTRREGYIHNLPVEKRSVVVPKSPMTIEEALPFTRQWWPSWDTRKHISVITTEAAGIEQTCERLGGMVRESRGVLSQARQMQIIHQCRVSNLIWVGRDRLGPLEPRQVEKILGYPYNHTNLFELNQPDRLAAMKYAFQTDTLSYLLSVLKGKYQDGIRVLSIYSGIGGAEVALHRLGIPLKCVISVEESEVNRKILRRWWLKTEQTGVLRQHAGIWKLKTHVIEDLVKEFGGFDLIIGGNYTSCKGGTTVNTTMGMDSNRFYEYNRVVKRVRTAVGLS*
