# Supplementary material for: Identification of DNA methyltransferases and demethylases in Solanum melongena L., and their transcription dynamics during fruit development and after salt and drought stresses
Source: PLoS One. 2019 Oct 9;14(10):e0223581. doi: 10.1371/journal.pone.0223581 (PMC6785084; doi:10.1371/journal.pone.0223581)
Supplement: S2 File — Solanum melongena (Smel), Solanum lycopersicum (Sl), Arabidopsis thaliana (At), Cynara cardunculus (Cc), Ricinus communis (Rc), Brassica rapa (Br), Capsella rubella (Cr), Cucumis sativus (Cs), Cucumis melo (Cm), Fragaria vesca (Fv), Malus domestica (Md), Zea mays (Zm) and Oryza sativa (Os). (DOCX) [file pone.0223581.s002.docx]

> SmelDemethylase_1

MAGREGSMIPQADDCQIGTPWFPMTPAKPSLPPIYGNRQQNQLEQINGVQSQRISQGQDLSQVNKIELKDFLQEPQAQCAAACCGLTNSVTVTEYFDAWEAEAGTESQMYTNNNVNNISTDNVDEWSNVSFGHLLALAHVAGSTTAIENTNAETNFTTNGSFGSLVSSRDADGSSICSRFPFNLNSPTRDEDLSCNNAFQFEPITPYQINKKGSASDAPSLDFNETPVARHVQSSKDTLKRAEANDLNTEQSGQVLNISELPENRMIDKAVNQDAEQNNTPQQKRRKKHRPKVVIEGEHKRTPKPKTPQQHGSMETKKEKRKYVRRNKFEEPPSTPSDEVNDMTRHEGHLPSPGKIQRAKRTNIRRNQVNRFTINPAEEGTLDPPYVSRPRRFPRRSLNFDSETRLNDENSSHWSSSTVEDLHENQSNSSVHPGNGIEVTTAKTALSSVYDVTYSNQELKTCQTHHDHSTQKKIGLNHGKFIMNKQNEVSRGKCKIIFSDETHDKQASILEMTPKRLNSSNCSSSTCLIQETPERVFKRRRSFRTGEAKLYSANVRGAYFNSMQAYQAILPANEPYAQSTQGMHFPTIYKKKRTEKGHPSATSYIKPFTCEINSLFMSQSNIGLSQASTSTNDKANNLMPNQELVPAFAEVEGLRRKRSKSISKVRDLASLLEICKHFPTLPVKTVSEFGERCEISDQPNTCMEALVADTRAIMKTKKRSKRSTLVSSTASHMYALTEFTTNARGSIPAITWRSPVDEIVKRLEQLDLNRESTRPYQYAENALVIYQRDGSIVPFARSFVRKRRPRPKVDLDDETTRVWKLLLQDINSEGIDGTDEDKAKWWESEREVFHGRVDSFVARMRLVQGDRRFSPWKGSVVDSVVGVFLTQNVSDHLSSSAFMTLAARFPLKSDISVEKNEESTGIIIEEPEVSNLEPDDTIGWHDDQSTQQTPGQEFSSAESDDEKTAVHSSESSENSTNCTSSTENYILQQPGSSRESSCVHHEATTYGSTIAIAATKFLGDQVDPDNLLSSQNSAISSQDANFSAVPTSEGTESRNFLGSASFLKLLQIARTSKSHRVQDQKTENILLEKDIDGQLKHMACCSHFQMHGENHRGSIENDCTCSYLGSYTVSNSGARQNECKSNLEEAAKFSDLSTELDVPEQSKSSAKPANRALYGEMSETYISHNNNQNKVYTATIDDPVVNIVQQIQIEESNYNMQRVTEAPKFSEASIDVREEVSIVDSSKSEHTALGSNTNKGKYHAGSTLDRANHNAKAKKERLGKEKPNVDWDSLRLQAQSSSKKRERTANTMDSLDWDAVRCADVNEIAHTIRERGMNNMLAERIKDFLNRVFREHGSIDLEWLRDVPPDKAKEYLLSIRGLGLKSVECVRLLTLHNLAFPISEPIFEDIINMKIACVPTNQVFNKVDTNVGRIAVRLGWVPLQPLPESLQLHLLELYPVLESIQKYLWPRLCKLDQRTLYELHYHMITFGKVFCSKSKPNCNACPMRGECRHFASAFASARLALPAPEEKRIVSATENKAAGQNPFQNFNQQLLSLPQANQTPLEHPKLIDSAPVIKVPATPVPIVEEPATPEPEQEAPEIDIEDVCFEDPNEIPTIELNMAQFTQNVKSFVQNNTELQQVEMSKALVALTPAAASIPTPKLKHISRLRTEHQVYELPDSHPLLEGFEKRELDDPSSYLLAIWTPGETSNSIQPPGRQCNSQETGILCDNETCFACNSIREAHAQTVRGTILIPCRTAMRGSFPLNGTYFQVNEVFADHDSSLKPIDVPRNWLWDLPRRTVYFGTSIPSIFKGFVCVRGFDKKTRAPRPLIARLHFPASKLTRTKEKPDEN

> SmelDemethylase_2

MDTGQGSSWIPATPGKPDFAKSPPTSSNREENQQTQVDWLDLQGKQALGHATGSTVKAQNAAAYSSSTSSVTVDQCFISSEAAAGTKYGGGINMYNNFPSDNVDMWNNMSFGDLLAMAHAGGSGTTPADEIAFSVKSSFQTPINTPNADERSNFSSFPFKMNSPSRMTGATSSCNISFQFEPVTPDLIKIKVQGSNESNLDINVTSAARITQSNEDIIKRAEANELQQNKVQSELTLNQSELQEYQPDKEGKQVTQLNNTPEQKPRRRKHRPKVVAEDKPKRTPKPRTKKQPGSEETKTEKRKYVRRNKAGEPAATFAEEVNSTICHEGKAPGSEKTPTAKRKYVRRNQATKSTGKPSEEGSSGTIGRPAATSAEEINNTIFQNGKPPCSEETPIRKRNVRRNQANKSMEKLSEEGSSGTIDPTEVPRSRKTCRKSLSLEFESPPSDENSSYKPSTMDLQANNSGSTSQSAESVQLGPGKEATSEETEVGITCNITSLNQEVRNYLPQPAMQYPGPPTPDKAGWNHGKAMVGNHNESTRGNNRVIFSDLTLDKHASILRMTPQNLNCSNSSSSACLPNGKGLKRQHSCRTDEAQFYSINARGAYFNSMQAYQAILPAHKPDVYSNVGMHFSAIYKKMRAEKGHSSTSSYIRHFTGETNCVPSSQCNISGSPSNNSATSIGNNRMWNSNAMSAFVEAERLRRRSNSVTQVHDLASLHEIYKQFQTSTSKEATTYGFGERYKATHISSACMGAPIADTRAAMKTKRQSKKSILVSSAASNMYAHQHFTKNARGSLPALTWRGMSLIDEIAERLQHLDLNRESSQNEGQHGIITDHTKFQRESALVLYQRDGSMVPFGSSLARKRKPRPKVDVDDETDRVWKLLLQDINSEGIDGTDEDKAKWWEDERRVFNSRADSFIARMRLVQGDRRFSPWKGSVVDSVVGVYLTQNVSDHLSSSAFMSLAAHFPLKTDSTQKHEGNTGIIIEEPEECATEPNVSIRWYEDQPNQSTHCQDSSGVYSTDSNEEKAVVNDSESSENSTQCIKSAECSVILQSDSSREGSDLYHGSTVTGSQDQKELNDLPSSSSSVVSSENSAVIQASEGTDSSNFCSSTSFLKLLQMAGTSGARGTSCPEHLQEGENLPFLGKELSGPEKSELSAESAHSALSAVNPQNKLDIETVNDAVVNVEVQFQTEDSNCNVQQVAEAPTFSETIADVTERANIIFDSCKSEQRGLESNLKNDTNHVCSKVDKVNDSPSKAKNGRPGKEKEDIDWDSLRLQAQANGKKRERTRNTMDSLDYEAVRCANVDEIAHTIRERGMNNKLAERIKAFLNRIVSDHGSIDLEWLRDVPPDKAKEYLLSIRGLGLKSVECVRLLTLHHLAFPVDVNVGRIAVRLGWVPLQPLPESLQLHLLELYPILESIQKYLWPRLCKLDQKTLYELHYHMITFGKVFCTKSKPNCNACPLRGECRHFASAFARYNVFHKLDLIKMPQFSARLALPAPEEKSIVSATEDKAANNNPRENFSHLPLQLPPGNEQPVEHQKLITSAPIIEVPATPQPIVEVPSTPEQEQIQAPEIDIEDTCLEDPSEIPMIELNMTEFTQNVKKYVENNMELRQVEMSNALVALTSEAASIPTPKLKNVSRLRTEHQVYELPDSHPLLEGMDKREPDDPSSYLLAIWTPGETANSTQPPETQCNSQESGTLCEDETCSSCNSIREAHSQTVRGTLLIPCRTAMRGSFPLNGTYFQVNEVFADHDSSLNPINVPRDWLWNLPRRTVYFGTSIPTIFKGLNTESIQHCFWRGFVCVRGFDHKTRAPRPLLARFHFPASKLNRTNGKTNEDKGVAS

> SmelDemethylase_3

MNLGRKFSTPQENGVVQNGDHRIPFTPQKPLLQRPNLVPAEMQGNQMERTDWSKLLGMYGDLLKMPACDAEAAQNPATPVCLNKGYRGHWTDVAVGHRCSHTDINSCENVPDHSKPACTKVNSLEELIGMKNQSNVLSIRERSSNSIQLSNIPILHNSYAQVDSRYEQPQLKSAVQNNSIHLGDIPSFHNSYSRYEQQQLKSAGQKLLNESQAFTGPNHTSDCYNRPQPLDGTQGPNQANRSLISPLAPDAGTSSSTNSFFPFAPVTPEHNHFKDTQHFERENFQIQERSSFEKVKRDNVLGSMKSKDDHSDKLLQRVADSLVAVSSLSEKVDNGNIGNVDIDLNKTPASKTPKRRKHRPKVVIEGQTKRTPKRAAPGDGTPNENPSGKRKYVRRDGLKVSTTNQTEVNESAAPRNSTPNENPSGKRKYVRRKGPNATTQQTEVVDKDKVPDAEETQKTCRKMLNFDLEDITKDESLASTNIRHTEEHQQRKTTIDLNLNSQDMEASLAIVEASAISAGHNQRKGESAEELLKEKPKDLAVPPPSANQATRNNQALNALARSLSMRTVTQYHNSIQLYEPRQLALGRTPLLLRDTASTHDNKGRSNRDQFPSFPFQPRIFSQIGSVRPEMLGNDNRRRNCSTSSGFPFSTAAAIHDSTKFLPSSFSINGYNGVSEEGTRHYAGPMVVQHNSQLNPSQFHSYTETIPQHITQQMAGIHGSQVQATSSNLNHQYQFQSLSTVAQEVERINSHNNLRGQMQNMGKTSPTEPFNEVLARENRNSQADQHHLTKATGLQGTRRHAASVGTGLQGTHGYAVSIDIITQQLERLIISDRKKNAAKVEQKALVPYKGSGTIIPYDGSDPIKRRKARPRVDLDPETNRLWNVLMGKEGGTETMDKDNEKWWEDERKVVRGRVDSFVARMRLVQGDRRFSPWKGSVVDSVIGVFLTQNVSDHLSSSAFMCLAAKFPLPTSTKNTLSQDGCNIVMEEPEVEIIDPDGTIIYHKARLQHRMESHFHTTRAYLVSEHDKRSDEEVISLQNSPDSLIVQANEELRSSSGSDSEYEDQPSSPNLNKDSTQANRSPPTKWTAAFPEYQSHFMRNTLSEKLSIFGHQKTDTASDMRHNQNLDAETYLHGYPVNPHVQVEEISARTSSNSWLKIIPEFGKHQTACPEKENALGKSMKHIAGSSSTLIAQQTALPIIHAPLMGEIGNVKRQPHKENNQHSVSSHQKEMAIASQPESACIRQSANHSEAIAKGEEGQAHPSSTQPSVAGTNISKTRKRKVEEGDKKAFDWDSLRKEVLSKNGKKERSKDAMDSLNYEEVRRASVEEISDSIKERGMNNMLAERIKDFLNRLVRDHGSIDLEWLRDVAPDKVKEYLLSIRGLGLKSVECVRLLTLHNLAFPVDTNVGRIAVRLGWVPLQPLPESLQLHLLELYPILESIQKYLWPRLCKLDQRTLYELHYHMITFGKVFCTKNRPNCNACPLRGECRHFASAYASARLALPGPEEKSIVSSAVPIPGKGNAAAASMLLPPAAEVRMVYPNAPVEAANIPSFLERPMSIPQEMTNLLNREATLITSNCEPIIEEPKTPEPLPELLESDIEDGFFEDPDEIPLIELNMKEFTTNLETILQEHNKEGDVSKALVALKPDAASIPTPKLKNVGRLRTEHQVYELPDSHELLEKLDKREPDDPSPYLLAIWTPGETVDSIQPPETKCDHSGPGSLCNETTCYSCNGIREANSQTVRGTLLIPCRTAMRGSFPLNGTYFQVNEVFADHESSLNPINVPRKLLWSLSRRAVYFGTSVSTIFKGLSTDQIQYCFWRGFVCVRGFDREMRAPRPLIARLHFPASKMVKNRSDDKKKDGAGAEKVAGSNSPKSVHTK

> SmelDemethylase_4

MEVGNADKKKFEEVPFFTPTTPLKCMPAATGFNSINAGFNNFLSTSSSGEEVEMEVGNADKKKFEEVPFFTPTTPLKCMPAATGFNSINAGFNNFLSTSSSDNSQKKDEEVSAVIGGRTEFLSQYLDGFSKSASVAPSTPMKGNPRKKQCRSLDLNERPLKKPRIKKHTPKIFDESKPKKTPKPKIKPSIPKSRNFKASAKIKKKLEDCDKVSDDLSEDMDMQTPNSKIPETIPESLVLQVSTPQPQILEHAIPAISPPDLQHNILTDLGHVSKSSKRSLHFNLENEMDVFSVGEAEVIRNHVTHGHNKFLSNPLDSVNEQAVDINSSIGLEDNLEAESTSQPNQGNNPSIYQDDQRAYQHHFLKVYERRKTKIDSMPLAGNEAHIYQSNHGSIVPSNCWDHFPKVYKRRTRVNTDVVPLAGNDAISQNDHKARDKGGSQLDFLENYRTQDNIGTADYADKGWNALDINTKCKLTSSAKRTGRRTGKKRATNKRVKIARRDKKSCFTISKRISRRNFEIVNGAKNTCFSTINTGQKGKFSSQFVGKVLPNENQIAAAMADPESFECVFSLFPMVKSRRKRSIHPKRTKSVCREENTELEPLSLALTLSPLITSKRKRSKKCNRSTAISAINGIESFTNKWSQISSWNEVQECSQHEDSWPQTNKSACNDIQECPRHEGLLPRTNKSTCSEIQKCPQHENLRPRTKCSTNFESLVAKLIRRFEKIKICGKRTYKKKTTSNNAGMLVLKNPLGAITTYKKKDKLRETLPKVDLDMESLRMWNLLIENGGIDEYPDEEKKWEEQRNIFKGRVASFIARMRLVLGNRTFSQWKGSVVDSVVGVFLTQNVSDHLSSNAFMLLASAFPLQNKHEMTTQQEQALAITQGMQDSFEKDFDAYDSSSRNASECLQEENIGNESTCGSQSSVNSCNPEVITFAKSCDLGNETGKLPNMTSGEKNKSPKREIDWDELRKTYSTGKYSGSTESNGDSVNWEAVRNADVKQIFETIKCRGQANVLAAKIKNFLNRLVEDHGSFDLEWLRDVPTDDVKEFLLSIYGLGPKSVDCVRLLSLRHHSFPVDINVARVAVRLGWVPLQPLPDGIQMHLLESFPLQSSIQKYLWPRLCKLDMLTLYELHYHMITFGKVFCTKKKPNCDACPMRAECRHFASAFASTRLRLPGLKKEGEATSEQPDIVHDVPNMHVSLPNLSHSSESFLESSSFQTQGCEPIIEMPESPEHRHLESLEQDIEDFPYEAEHKQEIPTIKLNTKAFGENILNFIDKSNDEFKESMLSFVDEISRLHRDEEVSKVLVLWNPKSASDPARKLKTEGRLRTEHSVYELPDDHPLLSGKKKGKVSSSKELELYDSEMCLDSTSYSPNDQIVYGTILLPCRTVNRGSFPLNGTYFQINEVFADHETSINPIPVARASIWNLVRRTLYCGTSVTSIFRGLSTEEIQSCFWKGFMAIRGYDRKQRAPRPLHYRFHSKDGEI

> SmelDemethylase_5

MEKSSKGSCNIKNEGINRRTNPNRGKNKKETRNQRAEPILRRKSDNADLKSEAIEWKNIRKSSSFVNPKERNDNTLDAVDWHAVHAATAEEISKAIMQRGMDKKLAQRIKNFINRLILEQGNVDLEWLREVQPEKAKEYLLSIYGLGMKSVECIRLLTLQHQAFPVDTNVGRVLVRLGWIPIQPLPRGREMHLLNMYPSVKDVHKYLWPRLCTLNYLTLYEFHHQMITFGKVFCTKTSPNCNACPMKAECRHFASAFTSARLLLTGPGETSSASTDTALVPVDSDSCQANLVSSFGDASNSQGDHQNELITYPALECIGQTDIEDYCANDIPTITFDCQAFMENLLSYIDENDVSTKGTDLSKALVIATQEAPLPKLKHIGRLRTEHQVYELPDSHPLLEGIEKREPEDPCPYLLAIRTPASEEPTKEKRESDETQNKVDASNIGAGNDIQTVSGTLLIPCRTANKGKFPLNGTYFQVNEVFADEESSQQPIEVPSAWIWNLPRKTLYCGNSIRAIFRGMSTKEVQNCFWRGYVCLRGFNRKTGIPGSLPLDLHRPISKLGKAKRS

>CcDemethylase_like3

MDLKSEFGYFESFYNGFENQKHHQVDLDSAGYVAAADAAHRLMESLTSSNFSSNDCISISIDFHERLEPIVGLIREEVSPMKLSNDDEDKLVGEKQAMTKMQQVKKFRPKVMTPKPITPSPSSSSSRGHTSVKSSCRRALDFDAQTKTMLIYTKGGHCFTRSVNKYMEGRLECNDKEEDMNEQEEELFRQRALSFITSMRHVQGNRGFMGWKGSVVDSVVGVFLTQNAPDNLSSSAFMCLAAKYLIEDPKEGISKHALDWNAVRCAQPYEISHVIQERGMNNRIAARIQVTFLDSIYNHKSGLLDDLEWLRKAKPEKTMEFFSKIYGLGIKSMDCLRLLTLRQHAFPVDRHVARIVVRLGWVPVEKLPDGVLIHELEEYPMMEAVQDYLSQRLSNLDVDTLYELHYQMITFGKVFCTKKKPNCNSCPLKKECKHFASAFGRFPPQGEFKTFVPRTPTPGQSSSRVQVILEEDIEDLCKVHPVIKVMKNAAGKGKEEEEVVVGRFLVPCRTATRGSFPLDGTFFQINEVFADDESCKKPVVVSRNLLSDLTIKTLFCGTSISAIFQVAAVSIDLVPLVTASHELRLCRLPRVAASHELRLCRLPRVAASQSAVSISPPSYYSFLLLLVPPSASFRLLPVGRSSLDNS

>CcDemethylase_like4

MGEEGESSSSRQVYPPEVAYAPATPAKPDRSDWGPIGIDWLKNQFDEVIFEETSAKKSISCWEGNSVSTSHIYDLSGFSMDDVETWNSISCRDLLALADATIRRGSDDGDHDRSDGLDFDNRSNCIDTQQYGWLNLGNYSPDLNLPPEMVMKPLVSTGLSTQITPGTPDQARRAEHKQMGSDIVAKVVADNNKERYNLDEQPQVQVLVEQLQGDVSTIVEANQDFEKGLTAETNLNETPQPKQRRRKHRPKVVREGQQKKAKQSATPQKPDGSSTGKRKYVRKKGVEKSPATPAVEEGSGTIDPSSDQQNKKSCRKKINFDETEKGNEVTVETAEVNITVDKTCSMNQVVETILESQSASPITPSKTELPIKDAKHTYRKVKCRINFLQETHDKRPSSVSSPNESNCSTSASFNKGEAQGSKMELSSKIVGMELWDENAIGVGCNLSKFTNDCSGGKQGMHLPANKKKRIEKCRSSITSGAISSVCSAQSSDCSFLAEQNASKAPQMSKDYVLKDDQQPYKQAFGHLENSKKKRRSKALSLIPDLALFPGIVEGRHWQTPKEGSRYEVAYQQQTYTEAHAADFHVSIATKKRMKKNAKLPSLYQDHLRFTKGCIDSLINQFERLDINSQMAEEGRDALIPYLSRYNEKNALVLYQERGLVPFEGLFNPVKRRKPRPKVDLDEETSRVWTLLLENINSQGIDGTDEDKAKWWEEERRVFRGRADSFIARMHLVQGDRRFSRWKGSVLDSVIGVFLTQNVSDHLSSSAFMALAARYPLKSKSSSEPLHDDESILSVKEPCQVDQDETITWHEKLNQPSGDHGPMMLQDIDLCEEKEVVNSNKFPKNSGCVDLNVSSEGEVPELAEKDLAMYKESVVDQIENDDIASSQNSANMSPSSVQSSVAHTTERLGSCSKEEQKDMSKATIFGGYTSFVELLHMQGTTTVHETYSQQQAEESSNKKIGQDELESVAYLEEQNDGISPHSNSSRALEVETFELREERNITQKKSQEEFASEESGLSAESASQAMVQLVKTTSSQEASKSCNTCHIRLQERSRSRCKMIAVNPNINTEQHTEDNNCEVQEVIAIANVSADNSKATESNNILKASGETAHKVVEINSVDHGTHQIVNGMDEGSSKVKRQKSGKVKQKIEWDNLRLHAEVTEKRERTPNTSDSLDYEAVRTADVNEVADTIKERGMNNVLAARIKDLLDRLVEDHGSIDLEWLRDVPPDKAKEYLLSFRGLGLKSVECVRLLTLHHLAFPVDTNVGRIAVRLGWVPLQPLPESLQLHLLELYPVLESIQKYLWPRLCKLDQRTLYELHYQMITFGKVFCTKSKPNCNACPMRGECRHFASAFASARLGLPAPEERMASLTENRTGQSSIGLIEQCHITLPSASEQWQQLSDIQNCNSGIEEPATPGSTVEVPATSGPIVEVPATSGTIVEVPATSGPIVEVPATPGPIVEVPETPGPIIEVPATPEPEPIQEEFDIEDFCEDSEEIPMIKLNIEEFTQNLQTYMEKHMVLGEGDMSKALVALTSEAASIPTPKLKNVSQLRTEHQVYELPDSHPLLEGLDTREPDDPCSYLLAIWTPGETADSIQPPEGQCCSQESGTLCSEETCFFCNSTREANSQTVRGTLLIPCRTAMRGSFPLNGTYFQVNEVFADHESSLNPIDVPRSWLWNLPRRTVYFGTSIPTIFKGLTTEDIQYCFWRDVDAVSVVNPRYKWQDNHVQTAAADIQILQKTLSSWPEWHDS

>CcDemethylase_like2

MNFERGFPIPRHGNEDSRSSVTHGIPVPPESGIEHHHGSWTATSGGSGTVGGGDARATPRRPIPMDNGTVHGSESWNTINLEQPTPMGNGVTEDRNPTLERTILQRSGIARNANDWKSVIPGNLFPHRNGIGQSRNSWSPATPEKIVNQRSNTENGLESENWQDLIGMYTGLLKEDTVDKNGVLEDINPTPSKVRDYGNQNWVASNNKNATHRSSPSPYWNHTSNQASTTSDPYLKTNDPANWNSNLLATLVGSQNSSTHTSSANKAQTNGIHHISNRSAVPNSANQVESNSMRSTSWTSMLGSQRTMRFTSNNLINDAHNTEDGFPVAYQPGYKPNSPPRSAASSIIDSFPFAPITPDNQGKHMHSQRVPENGNFRVEGTSTPAKDSRENQTVSREDAENLYNELLQTIGDSPPSAISTTQKELGVPENTDEQGIDLNKTPQQKTPTRRKKHRPKVIREGKPKRTPKPKDPSDGTSNETRVKRKYVRKKGINILETQGDDVTKNIPVASVGKRKYVRKKGLEKFGDEQQSRMDDVATSVVGIPAKSCKKQLNFDLDPVAQDESYGIRSSQQGIDVNVETPARSCKKQLKFDLEPVAQDKGYGIRSSQEGININVNPQDIGQERRINSILERPAIEIAQQNISMQSGNQYELNVPITPLANTKHHALNMLARNMTIKNSIPEHDRRGNLYNKVNQRFHGEGIENLVLQADMVSTNLERVREPNLMSTPQSLASKGMLNLDERRGIKRQSPEQMCLNANTMDSLLLYQKLLLGVAHRAYDRNNLSSILLENSKKTKMQSEFQTLVSSEPSCIIPHKPRQETRQINGIYGNGSAMHLLTSSTEGVNPYKTMHVGGNVINGQFRPPMAATHYLQKHQVFSGMQHHPLRSVPERSQRYIQGHDIGSKTAIMSWNLPPPTPSKETSRYAVTAYPATSLEKRQTAKPNSYNQRLNGLNQMFQHHRNDPLKGYQQPTTVARGRPRKQKPELSVDDITYRLEGLHIYDGNKKEQHELVLYRGSNALIPFEPIKKRIPRPKVDLDPETDRLWRLLMGKEGSEGAETLENGKEKWWEEERRVFRGRADSFIARMHLVQGDRRFSRWKGSVVDSVIGVFLTQNVSDHLSSSAFMSLAAKFPPKLSTTKETCCQDGACEEPIEVAEPNGITKCHEKIKQPVPDQSFFVSSKPSEDMTHQISSTRGAANKQSGISEEEVILSQDSFDSSTTQTVDEIRSSSGSNSEADDVTTGFETSKQSDPPVNLIQEKDHSCHDNWSTLIDEPKASIHHLPKEPECSMQLPRMNGVDLNSSSSFIPANSLQQESFVSSGQYQMSATPGPQKAGLLHFGVLGKESTSSLPSSNSEITEACHTSNVTCSENETPKFAGSSQGQYNLPSSHPVHQENFQPEPPVCSSQILNTNHPQVGEFFKETTRHGETLAKGKNGAQKQDTPMFEGIPSLVDKQICFENTVPEAKAKEQNHSSHEPPSGAGTNMSKAQKRKAEDERNRAFDWDSLRKEALSNGEKGERSKDATDSLDYEALRRAHVSEISDAIRERGMNNLLADRIKDFLNRLVRDHGKIDLEWLRDAPPDKAKDYLLSVRGLGLKSVECVRLLTLHHLAFPVDTNVGRIAVRLGWVPLQPLPESLQLHLLEMYPVLESIQKYLWPRLCKLDQLTLYELHYQMITFGKVFCTKSKPNCNACPMRAECRHFASAFASARLALPGPEEKRIVTSDAPVATDPIPPMVIRPMPLPQAENGFDKSERSFGRNCEPIIEEPTTPEPEAAELSISDIEDQYYEDSDEIPSIKLNMEEFTTNLQNIMQDSMELQDDMSKALVALNPNAASIPTPKLKDVSRLRTEHQVYELPDSHRLLEGLDKREPDDPSPYLLAIWTPGETANSVQPPERECSAQQSGKLCDRTTCFSCNNIKEANSQVVRGTILIPCRTAMRGSFPLNGTYFQVNEMFADHASSMNPIDVPRTWIWNLPRRTVYFGTSVSTIFKGLTTGGIQYCFWKGFVCVRGFDRKTRAPRPLMARLHFPASKI

>CcDemethylase_like5

MNFENGPDNRFNKATFGIGTRSFGLEDAVFERNTGGMYDGRFLMNNMWNTIPCSDLLALADAAITTKSEVNMHFQNRNQNQNHEDRQHERTRYLFDLNSPPIATDPQLITGISSQLSPVTPKITRRVDHRQVVSDIINLDEDGSTDEAADKQDNERSDPEQPQLILEQSQDTISMQLEEVNGDFEEELSDDFNPKKTPQPKQRRRKHRPKVVIESKPKSAKKSTPPQPDGSSTGKRKYVRKIMSERSTETPTSEASLPKTRNSCRRKINFEEEQNLHTELQGIKKKTEIELMCDKEFLTDQMTMLSPITPNKSELRHERLAKDVNPMSMRAKRNLSFSRKAHDNDSNCSTSGCFNEDGQETRGSKRECSGDENGLGNRCNNSLEAYLSMITNFPAIYKKKRTEKCQSSVTFRAVSSIWSPEYNNRSFPETSTVYGLNFQQSYKHMLAFGHVESFRKKRSKGVKRMRDLASFAGMIEGKKSASKALCQHHTCMEALSADFSASIATKKRTKRKYIVPSSSNPYDIDALINQFESMSLNNRMVEQDQHALVPYSTRYREKNALVLYKQDKSVVPFEGSYQLRRRKPRPKVDLDDETSRVWRLLLEDINSKGIDGTDEDKEKWWEEERNVFSGRAASFIARMHLVQDSVIGVFLTQNVSDHLSSSAFMNLAARYPLKSKSSNEPLQDDKSSISVKEPCQLDLEETIRWHEGNSNQQPAQDHGSIMLQDADSYEEKEVVNSNERSVRLKDILSGEVLEISRNDSGILESFTQENRGVDDLVSPQNSVDTCPNSVQSSIVDTAERLGSWLVRNSQSEPLDASKPIISEMFTSFASTKLHEVYSQEQAHKPSNKRIGQDKLGSINNSPEQNEGISHDHITSGLRIPPKDHELQTAHGSAALEAEYIELREERIIIDNKPEEKCASEQSEISAESASQATVEMVRTRSFQETPISCNTLQSRTISENSIIVTNQHAHENNCNAQKALDIENCSAEISEVTESNNMTDNSRETAHKIVESNSNEHGNLQTTASGINEGSAEVKKGKTRKAKKIKPNWDSLRIGAQVNHKRETTPNTKDSLDYEAVRCADVNEIADTIKDRGMNNMLAERIKDFLNRLVRDHGSIDLEWLRDVPPDKAKEYLLSFRGLGLKSVECVRLLTLHHLAFPVDTNVGRIAVRLGWVPLQPLPESLQLHLLELYELHYQMITFGKVFCTKNKPNCNACPMRGECRHFASAFASARLALPAPEERSIVSATENKIPDQSPTRITSQLHLTLPLDNHCLHQQSQVQNHDPVIEEPATPEPIVEVPATPEPEKIQQESDIEDFCEDPEEIPMIKLNMEAFTQNLQTYMENNMELAEGDMSRALVALASEAASIPAPKLKNVSQLRTEHQVYELPDSHPLLEGLDKREPDDPCSYLLAIWTPGETAESIQPPGVRCSSEESGTLCHKETCFSCNSIREANSQTIRGTLLVFADHDSSLNPIDVPRSWLWNLPRRTVYFGTSIPTIFKGLTTEGIQYCFWRGFVCVRGFDQKTRAPRPLMARLHFPASKLRRSKGMADET

>CcDemethylase_like1

MVKMVKGRGITLFIVASSDPPAASSSSSFSSPFSRSRSFSLLSISYLCSIVLTGNAIVTSTFLSSSHLDLHQFYQTSPKTLANCSKAKGEMTEPEFGSWRMTPVRSTLIGNGIVGQRDSCTTTHQENGIVVNGEIWNSAMPTKSFPQGNGNGNGTSSWTPVTPGKPVPQRSIPQNQVETENWEDLVEIYQDLLKEETLTLNKVVAQSLYPTPPPSTVGNIGNQHQVASTPNRNLNSTPIPNLNHGSYQPSTSFTYFPSEDPANWDSSSLLAAIVRPKKSSASLNIAQNTSLHASNRTSLPNTSTQVGSNSISVEPDAASSEITGPLAFAPITPDTRQKHTDSQWVLAKDRHESQRNEDGDNHYNEQLHTIGDSTSSAVSTTQKEHLVSEEGDELGIDLNKTPQQKTPARRKKHRPKVIREGKPKKTATPKDPKNTPPNETRVKRKYVRKKDVNVSETPQRNGVEISQNGVPRSSGKRKYVRKKGVENSDVQQKTRVEEATAPVVETPAKSCKKQLNFDLEIVARDGRQDINLNASPRDIEQERRINGILERSAMNVVQNNRYAGVGTHQKISTNHMQAGTQNMALPELLNVPSTPMAKARDVALNVLAQHLTMKNPITVRDVWRNGYNQVGQQQVSPNLEPSGRMVNFDERRGIKRQSFEQMDPHSLNAMDSLIMYQKLLLDRTDASNNLASIILESHKKTKTQSDHLQALVSSTPPLEDNLRGESRQINGVYGNAPASLQLLNSCTGRVEPSYKVMNAGGGNINGRQFQPPRAATQNLQKHMVTSGMQPIAERLQRPTPGHGVNPVTAMISWNRPPATPPKDYSRSALVTYPSPLVDKKRTATSNSSNRRSNGADQVFLQLRKDALEFQQQSFRKPNGGPRKHKVEVLVEDITFKLEGLSIYDGNDKKQNALVPYKGNNAIIPFEPIKKRKPRPKVDLDPETDRLWRLLMGKEGSEATETLDKDKEKWWEDERRVFRGRADSFIARMHLVQGDRRFSRWKGSVVDSVIGVFLTQNVSDHLSSSAFMSLAAKFSPKSTSTNETCCQDGACILVEEPIETVLPNDSTKCHDKIERQPVFNQSSFVSCESSEHMRHHHISSTKAAAIKDNRTSEEVILSQDSLDSSTIQTVDEIRSSSGSNSEAEDQITGFETSKEPGPANPMQAEKVSMFTELFSHDNRSPSLNDRSQYMHHLPKTPPYNMQIPIIGGVNNLNNASRFTPPNSSLHLVQEQLASSSRFQMNMAMGLQNVGSPGFGLLRGGSISSLPSSKSGITEAYHTSNVTYQENEMPRFQAPPLAQYDFLSNHPTHLKSFQPRSHIGSVLNSSHQQGRELYRETTVHAETLAKAQNGAPKQDSCSEDRVSAVDKQNCIENAAAEANSKEQNYASHEPLSGAGTNIPKVRKGTAEDEKKKAFDWDSLRKKVLSNGEKRERSKDAKDSLDYEALRRAHVNEISDAIRERGMNNLLADRIKDFLDRLVRDHESIDLEWLRDVPPDQAKDYLLSIRGLGLKSVECVRLLTLHHLAFPVDTNVGRIAVRLGWVPLQPLPESLQLHLLEMYPVLESIQKYLWPRLCKLDQLTLYELHYQMITFGKVFCTKSKPNCNACPMRAECRHFASAFASARLALPGPEEKRIVTSDAPNGTHTIPPVIMRPMSLPPAENNYSKDAQFSGRECEPIIEEPTTPEPESAELTLSDIEDQYYEDDDEIPTIKLDMNEFTMNLQKMQDSMEGDMSKALVALNPQAASIPTPKLKNVSRLRTEHQVYELPDSHPILKGLDKREPDDPSPYLLAIWTPGETATSTQPPERGCQSQESGMLCDRTTCFSCNCIKEANSQVVRGTILMPCRTAMHGSFPLNGTYFQVNEMFADHASSLNPIAVPRAWIWNLPRRTVYFGTSVSTIFKGINSLDALFVASLSAINTEILVLVGLTTQEIQQCFWRGFVCVRGFDQKTRAPRPLMARLHFPASKLVKTKNEAK

>At_DME

MNSRADPGDRYFRVPLENQTQQEFMGSWIPFTPKKPRSSLMVDERVINQDLNGFPGGEFVDRGFCNTGVDHNGVFDHGAHQGVTNLSMMINSLAGSHAQAWSNSERDLLGRSEVTSPLAPVIRNTTGNVEPVNGNFTSDVGMVNGPFTQSGTSQAGYNEFELDDLLNPDQMPFSFTSLLSGGDSLFKVRQYGPPACNKPLYNLNSPIRREAVGSVCESSFQYVPSTPSLFRTGEKTGFLEQIVTTTGHEIPEPKSDKSMQSIMDSSAVNATEATEQNDGSRQDVLEFDLNKTPQQKPSKRKRKFMPKVVVEGKPKRKPRKPAELPKVVVEGKPKRKPRKAATQEKVKSKETGSAKKKNLKESATKKPANVGDMSNKSPEVTLKSCRKALNFDLENPGDARQGDSESEIVQNSSGANSFSEIRDAIGGTNGSFLDSVSQIDKTNGLGAMNQPLEVSMGNQPDKLSTGAKLARDQQPDLLTRNQQCQFPVATQNTQFPMENQQAWLQMKNQLIGFPFGNQQPRMTIRNQQPCLAMGNQQPMYLIGTPRPALVSGNQQLGGPQGNKRPIFLNHQTCLPAGNQLYGSPTDMHQLVMSTGGQQHGLLIKNQQPGSLIRGQQPCVPLIDQQPATPKGFTHLNQMVATSMSSPGLRPHSQSQVPTTYLHVESVSRILNGTTGTCQRSRAPAYDSLQQDIHQGNKYILSHEISNGNGCKKALPQNSSLPTPIMAKLEEARGSKRQYHRAMGQTEKHDLNLAQQIAQSQDVERHNSSTCVEYLDAAKKTKIQKVVQENLHGMPPEVIEIEDDPTDGARKGKNTASISKGASKGNSSPVKKTAEKEKCIVPKTPAKKGRAGRKKSVPPPAHASEIQLWQPTPPKTPLSRSKPKGKGRKSIQDSGKARGPSGELLCQDSIAEIIYRMQNLYLGDKEREQEQNAMVLYKGDGALVPYESKKRKPRPKVDIDDETTRIWNLLMGKGDEKEGDEEKDKKKEKWWEEERRVFRGRADSFIARMHLVQGDRRFSPWKGSVVDSVIGVFLTQNVSDHLSSSAFMSLAARFPPKLSSSREDERNVRSVVVEDPEGCILNLNEIPSWQEKVQHPSDMEVSGVDSGSKEQLRDCSNSGIERFNFLEKSIQNLEEEVLSSQDSFDPAIFQSCGRVGSCSCSKSDAEFPTTRCETKTVSGTSQSVQTGSPNLSDEICLQGNERPHLYEGSGDVQKQETTNVAQKKPDLEKTMNWKDSVCFGQPRNDTNWQTTPSSSYEQCATRQPHVLDIEDFGMQGEGLGYSWMSISPRVDRVKNKNVPRRFFRQGGSVPREFTGQIIPSTPHELPGMGLSGSSSAVQEHQDDTQHNQQDEMNKASHLQKTFLDLLNSSEECLTRQSSTKQNITDGCLPRDRTAEDVVDPLSNNSSLQNILVESNSSNKEQTAVEYKETNATILREMKGTLADGKKPTSQWDSLRKDVEGNEGRQERNKNNMDSIDYEAIRRASISEISEAIKERGMNNMLAVRIKDFLERIVKDHGGIDLEWLRESPPDKAKDYLLSIRGLGLKSVECVRLLTLHNLAFPVDTNVGRIAVRMGWVPLQPLPESLQLHLLELYPVLESIQKFLWPRLCKLDQRTLYELHYQLITFGKVFCTKSRPNCNACPMRGECRHFASAYASARLALPAPEERSLTSATIPVPPESYPPVAIPMIELPLPLEKSLASGAPSNRENCEPIIEEPASPGQECTEITESDIEDAYYNEDPDEIPTIKLNIEQFGMTLREHMERNMELQEGDMSKALVALHPTTTSIPTPKLKNISRLRTEHQVYELPDSHRLLDGMDKREPDDPSPYLLAIWTPGETANSAQPPEQKCGGKASGKMCFDETCSECNSLREANSQTVRGTLLIPCRTAMRGSFPLNGTYFQVNELFADHESSLKPIDVPRDWIWDLPRRTVYFGTSVTSIFRGLSTEQIQFCFWKGFVCVRGFEQKTRAPRPLMARLHFPASKLKNNKT

>At_DML2

MEVEGEVREKEARVKGRQPETEVLHGLPQEQSIFNNMQHNHQPDSDRRRLSLENLPGLYNMSCTQLLALANATVATGSSIGASSSSLSSQHPTDSWINSWKMDSNPWTLSKMQKQQYDVSTPQKFLCDLNLTPEELVSTSTQRTEPESPQITLKTPGKSLSETDHEPHDRIKKSVLGTGSPAAVKKRKIARNDEKSQLETPTLKRKKIRPKVVREGKTKKASSKAGIKKSSIAATATKTSEESNYVRPKRLTRRSIRFDFDLQEEDEEFCGIDFTSAGHVEGSSGEENLTDTTLGMFGHVPKGRRGQRRSNGFKKTDNDCLSSMLSLVNTGPGSFMESEEDRPSDSQISLGRQRSIMATRPRNFRSLKKLLQRIIPSKRDRKGCKLPRGLPKLTVASKLQLKVFRKKRSQRNRVASQFNARILDLQWRRQNPTGTSLADIWERSLTIDAITKLFEELDINKEGLCLPHNRETALILYKKSYEEQKAIVKYSKKQKPKVQLDPETSRVWKLLMSSIDCDGVDGSDEEKRKWWEEERNMFHGRANSFIARMRVVQGNRTFSPWKGSVVDSVVGVFLTQNVADHSSSSAYMDLAAEFPVEWNFNKGSCHEEWGSSVTQETILNLDPRTGVSTPRIRNPTRVIIEEIDDDENDIDAVCSQESSKTSDSSITSADQSKTMLLDPFNTVLMNEQVDSQMVKGKGHIPYTDDLNDLSQGISMVSSASTHCELNLNEVPPEVELCSHQQDPESTIQTQDQQESTRTEDVKKNRKKPTTSKPKKKSKESAKSTQKKSVDWDSLRKEAESGGRKRERTERTMDTVDWDALRCTDVHKIANIIIKRGMNNMLAERIKAFLNRLVKKHGSIDLEWLRDVPPDKAKEYLLSINGLGLKSVECVRLLSLHQIAFPVDTNVGRIAVRLGWVPLQPLPDELQMHLLELYPVLESVQKYLWPRLCKLDQKTLYELHYHMITFGKVFCTKVKPNCNACPMKAECRHYSSARASARLALPEPEESDRTSVMIHERRSKRKPVVVNFRPSLFLYQEKEQEAQRSQNCEPIIEEPASPEPEYIEHDIEDYPRDKNNVGTSEDPWENKDVIPTIILNKEAGTSHDLVVNKEAGTSHDLVVLSTYAAAIPRRKLKIKEKLRTEHHVFELPDHHSILEGFERREAEDIVPYLLAIWTPGETVNSIQPPKQRCALFESNNTLCNENKCFQCNKTREEESQTVRGTILIPCRTAMRGGFPLNGTYFQTNEVFADHDSSINPIDVPTELIWDLKRRVAYLGSSVSSICKGLSVEAIKYNFQEGYVCVRGFDRENRKPKSLVKRLHCSHVAIRTKEKTEE

>At_DML3

MLTDGSQHTYQNGETKNSKEHERKCDESAHLQDNSQTTHKKKEKKNSKEKHGIKHSESEHLQDDISQRVTGKGRRRNSKGTPKKLRFNRPRILEDGKKPRNPATTRLRTISNKRRKKDIDSEDEVIPELATPTKESFPKRRKNEKIKRSVARTLNFKQEIVLSCLEFDKICGPIFPRGKKRTTTRRRYDFLCFLLPMPVWKKQSRRSKRRKNMVRWARIASSSKLLEETLPLIVSHPTINGQADASLHIDDTLVRHVVSKQTKKSANNVIEHLNRQITYQKDHGLSSLADVPLHIEDTLIKSASSVLSERPIKKTKDIAKLIKDMGRLKINKKVTTMIKADKKLVTAKVNLDPETIKEWDVLMVNDSPSRSYDDKETEAKWKKEREIFQTRIDLFINRMHRLQGNRKFKQWKGSVVDSVVGVFLTQNTTDYLSSNAFMSVAAKFPVDAREGLSYYIEEPQDAKSSECIILSDESISKVEDHENTAKRKNEKTGIIEDEIVDWNNLRRMYTKEGSRPEMHMDSVNWSDVRLSGQNVLETTIKKRGQFRILSERILKFLNDEVNQNGNIDLEWLRNAPSHLVKRYLLEIEGIGLKSAECVRLLGLKHHAFPVDTNVGRIAVRLGLVPLEPLPNGVQMHQLFEYPSMDSIQKYLWPRLCKLPQETLYELHYQMITFGKVFCTKTIPNCNACPMKSECKYFASAYVSSKVLLESPEEKMHEPNTFMNAHSQDVAVDMTSNINLVEECVSSGCSDQAICYKPLVEFPSSPRAEIPESTDIEDVPFMNLYQSYASVPKIDFDLDALKKSVEDALVISGRMSSSDEEISKALVIPTPENACIPIKPPRKMKYYNRLRTEHVVYVLPDNHELLHDFERRKLDDPSPYLLAIWQPGETSSSFVPPKKKCSSDGSKLCKIKNCSYCWTIREQNSNIFRGTILIPCRTAMRGAFPLNGTYFQTNEVFADHETSLNPIVFRRELCKGLEKRALYCGSTVTSIFKLLDTRRIELCFWTGFLCLRAFDRKQRDPKELVRRLHTPPDERGPKFMSDDDI

>At_ROS1

MEKQRREESSFQQPPWIPQTPMKPFSPICPYTVEDQYHSSQLEERRFVGNKDMSGLDHLSFGDLLALANTASLIFSGQTPIPTRNTEVMQKGTEEVESLSSVSNNVAEQILKTPEKPKRKKHRPKVRREAKPKREPKPRAPRKSVVTDGQESKTPKRKYVRKKVEVSKDQDATPVESSAAVETSTRPKRLCRRVLDFEAENGENQTNGDIREAGEMESALQEKQLDSGNQELKDCLLSAPSTPKRKRSQGKRKGVQPKKNGSNLEEVDISMAQAAKRRQGPTCCDMNLSGIQYDEQCDYQKMHWLYSPNLQQGGMRYDAICSKVFSGQQHNYVSAFHATCYSSTSQLSANRVLTVEERREGIFQGRQESELNVLSDKIDTPIKKKTTGHARFRNLSSMNKLVEVPEHLTSGYCSKPQQNNKILVDTRVTVSKKKPTKSEKSQTKQKNLLPNLCRFPPSFTGLSPDELWKRRNSIETISELLRLLDINREHSETALVPYTMNSQIVLFGGGAGAIVPVTPVKKPRPRPKVDLDDETDRVWKLLLENINSEGVDGSDEQKAKWWEEERNVFRGRADSFIARMHLVQGDRRFTPWKGSVVDSVVGVFLTQNVSDHLSSSAFMSLASQFPVPFVPSSNFDAGTSSMPSIQITYLDSEETMSSPPDHNHSSVTLKNTQPDEEKDYVPSNETSRSSSEIAISAHESVDKTTDSKEYVDSDRKGSSVEVDKTDEKCRVLNLFPSEDSALTCQHSMVSDAPQNTERAGSSSEIDLEGEYRTSFMKLLQGVQVSLEDSNQVSPNMSPGDCSSEIKGFQSMKEPTKSSVDSSEPGCCSQQDGDVLSCQKPTLKEKGKKVLKEEKKAFDWDCLRREAQARAGIREKTRSTMDTVDWKAIRAADVKEVAETIKSRGMNHKLAERIQGFLDRLVNDHGSIDLEWLRDVPPDKAKEYLLSFNGLGLKSVECVRLLTLHHLAFPVDTNVGRIAVRLGWVPLQPLPESLQLHLLEMYPMLESIQKYLWPRLCKLDQKTLYELHYQMITFGKVFCTKSKPNCNACPMKGECRHFASAFASARLALPSTEKGMGTPDKNPLPLHLPEPFQREQGSEVVQHSEPAKKVTCCEPIIEEPASPEPETAEVSIADIEEAFFEDPEEIPTIRLNMDAFTSNLKKIMEHNKELQDGNMSSALVALTAETASLPMPKLKNISQLRTEHRVYELPDEHPLLAQLEKREPDDPCSYLLAIWTPGETADSIQPSVSTCIFQANGMLCDEETCFSCNSIKETRSQIVRGTILIPCRTAMRGSFPLNGTYFQVNEVFADHASSLNPINVPRELIWELPRRTVYFGTSVPTIFKGLSTEKIQACFWKGYVCVRGFDRKTRGPKPLIARLHFPASKLKGQQANLA

> Rc_DME
MDSRIHLGEGFSVSQQETELQYVDSWMPVTPNKPIAIRSNPVLIDRHESQLGSTNWQELVGFPSGYVQEMSNYSSLAQHCTQIDQFTRNGSFAEKNRMINHIAGSYTRALLNENVSWSSNSLADLLVMNNTAPTAYPSRTLHRNTSIAERPLIPNLNTPVNSLREFNSGELFYTNQAHCSSSNVPSGHNSLFQMPQYGFPIPYNPNYDLNSPPSIEADAASTVTNSFQFAPIIEQAKKLENQLSALVNFPQGKGSSEERDKQDNYVVSLGNVPNQHNPDKLFQNIVDSASAVISTPFEEPKESCQGSDQVIDLNKTPQQKTPKRRKHRPKVIVEGKPKKTPKSVTPKTVDPNEKAIEKRKYVRKKGQKESTTEHPDSIGETTNSTEKPKQKRKYVRKKSLKEPQIRNADYAGETTYPSAGTAASCRKALNFEMENTYSEREKNLVAQQEIMNKGKETYNLNTGFHVSESLETHRTKSDLQMRRHNGSLLEFQQSRDVNNLTPFMNQISNNHQSNSHRREGAVRPTARKDGQMDNSNGSGRDIDVGMLQHIHAEGTGRTVLPEKTNCKSLEKNEEIVYHSTESVTKIPLLTEGRGYKRDYHQAELTMQNTGNPRGKLIFQEGVLIDDCHLNSHNSNAACPETCKKQKNDGIQKNKNGMPPPVAAVNQSGGGNSKTDSSASTVERNRELLKSYLKSKRDVVEHYKHSVASGQDLSLQHKWAGQNSCIERTGENCNIVPPTPPKMAPQSRDQLQPQICHIDASTKQTMASTQSLSVPSRKGNMLQTQKNILKDQKSTAKRKAGQPAKQKPITIEEIIYRMEHLNLNEVKGEQTAIVPYKGDGALIPYDGFEIIKKRKPRPKVDLDPETERVWKLLMWKEGGEGLEGTDQEKKQWWEEERRVFGGRADSFIARMHLVQGDRRFSKWKGSVVDSVIGVFLTQNVSDHLSSSAFMNLAAKFPLKSMRNRTCERDEPRRLIQEPDIYMLNPNPTIKWHEKLLTPFYNQSSMTPHESIEHRRDQETSCTERTSIVEAHSYSPEEEVLSSQDSFDSSIVQSNGVIRSYSGSNLEAEDPAKGCKHNENHNTSNAQKLEFEEFFSHVSGRSLFHEGSRHRHRELEDLEDGQQWTRLDRLDNSLKGSSTFNQHDNSNNSQLQTRVESSQLYREDSISSWPSSTSKVGKEKDASCTSIRVLQGAENVAKPTTQQYGSEKYPETSTAESHAFLCKQLMHEQSNPQLYHGSQSHEMNKTFQLGSKSIAEPVNLSDAQDYRQSSYGQHVSNIPQLAAKVFDVEERITLMDNKQTDSENNFIGSNSKENTHFTNKANLNRNASKARKAKAESGQKDAVDWDSLRKQVLVNGRKKERSESAMDSLDYEAMRSAHVNEISDTIKERGMNNMLAERIKDFLNRLVREHGSIDLEWLRDVPPDKAKEYLLSIRGLGLKSVECVRLLTLHHLAFPVDTNVGRIAVRLGWVPLQPLPESLQLHLLELYPILESIQKYLWPRLCKLDQRTLYELHYQMITFGKVFCTKSRPNCNACPMRAECRHFASAFASARLALPGPEDKSIVTATVPLTTERSPGIVIDPLPLPPAEDNLLTRRGSDIVSCVPIIEEPATPEQEHTEVIESDIEDIFDEDPDEIPTIKLNMEELTVNLQNYMQANMELQECDMSKALVALNPEAASIPTPKLKNVSRLRTEHQVYELPDSHPLLNRMDKRQPDDPSPYLLAIWTPGETANSIQPPERHCQFQGPDKLCNEQTCFSCNSIRETNSQTVRGTLLIPCRTAMRGSFPLNGTYFQVNEVFADHESSLNPIDVPRAWIWNLPRRMVYFGTSVSTIFKGLSTEGIQYCFWKGAYAVEMRTSILEFLSTYRLSFYSITQNLHYY

>Rc_ROS1
MDIKNKEELKEDSWIPQTPFKSILTKPNDEFSPDFFSRNGSWEVASNPLNAKRDTNIKTCNRTFPPLLILQDQASTASTSGNSHNNGGFLAAQNHHTGKHVQGFQYSLPILQGSAYDLNLPPGGTAVTFTSKTSCQIAPMTPEKGTRVECRQVAQLQFFSADERANQEGNEQDDQVAMVSFDINRPQTDNELQKTEKEGSHVINLSETPEQKPRRKKHRPKVITEGKPRPRKPATPKHALNTERPTGKRKYVRKKPLSKAPTTNLAVSTEIPAGKRKYVRRKPLNKISTPPVEATGKSVSTISVEPAKKSCRRSLNFYTEGQPRDNNSKCKLNSDEDSQLQAHDVGEGNQSKSIVTVGNGIEVIVETTQTGIAYDLNHSVNQKLQDYLALPEKQAPSTPVYANIDPARRIRNNNSQESPAEREGQVTTHSVQQNAAQSMLQTDNQLPPINPNDSICGKYTTLTMEGQSNGSKRKVFSCIQQPGTCNTNLTGIFCNALPQYQATSEFQVPSNFKKRRTEKAHIATTCSISCSTTLSGTLRLDIPGPQKNNRGDPPTSIVDCLLPASHSNADRIPGAFGETNGCQLKNETSGCVLAQTERSTKKRSRGHTRTRDLASLTKIAQGAMHLSSTSRRAYDVQQVRDINGPDACVDALVAEMRGTLTRKKRTKKRLLVSSASSSTNVEQAHGKIILYNQNLFSANSLVALPEVIREQMSTIDAIAEHLQHLDINREGSNFTYIEQNALVPYSAGNEQQNALVLYRSNGTVVPYTDSLIKKRRSRPQVDLDEETNKVWRLLMANINSEGINGTTEDKAKWWEEERNVFRGRANSFIARMHLVQGDRRFSQWKGSVVDSVVGVFLTQNVSDHLSSSAFMSLAAHFPLKSQSHNEQCYEERTGSVIDKPIVCMPDLEDTLKWNEEMSNQSICDQTSMTLHDSELDEEREAVYSNESSTSSTGIVNSSGLETCYESKENRSTTEIIKIIDTCDVREKTRVTNDALSLQHSVFSSHNFVNSPIDQTAERKEAFSGSEAGFVTEGHRPNMLNKETLDTMESSSALDKPEIFLQKTTESDMIEHGFSEIKELDEMNAATRKAKSRRVGKEIREDVDWDALRKQVEANGRKRERTPNTMDSLDWEAVRCAEVNDIANTIKERGMNNVLAERIKNFLNRLVREHGSIDLEWLRDVPPDKAKEYLLSIRGLGLKSVECVRLLTLHHLAFPVDTNVGRIAVRLGWVPLQPLPESLQLHLLELYPVLESIQKYLWPRLCKLDQRTLYELHYQMITFGKVFCTKSKPNCNACPMRGECRHFASAFARSKNLNVIKETSYNWSFRLSTMNSMCKQSTGLFPVSKDHHVKCSARLALPGPEEKGMVSATENRTNEPNPAAMVGQLPLPLPQATEQSEENQLSKSIQQSEAQYGVNSCEPIVEEPSSPEPERIQVTENDMEDTFCEDPDEIPTIKLNIEEFTQNLQNYMQNNMELQECDMSKALTGSSIQYGHPLYTLTLFQSHISPSYWTCACETANSIQPPENNCNLQEDGKLCDEKTCFSCNSIREANSQIVRGTLLIPCRTAMRGSFPLNGTYFQVNEVFADHDSSLNPIDVPRSWIWNLPRRTVYFGTSIPTIFKGLTTEGIQHCFWRGTR

>Rc_DML3
MADIRIYQLKSEDDMEFGSTMENKKETEQPEVQNNWVPETPAKPAPTRMLKFYSRRCQKGNSATGSLAVISKLEAQADDILFVGAMTFADKSHEQKDQKPLSMEPLCSSDGVIGLAKSTGNLEDVDIGFTRSEDVEGSEEGIPESASEDYKGEPASFVVGSPEDSDIEDFNLEKTNNSLEDRQACTGNVARRLDFSALGLGNLNVPSDSIPSTEPLSYAGLAPPCIGATTQSEDQSSSQEFSRASAEGAVYQKIDTTFSLSLDQENNTNIQDYHATRCDGQKDVQGNFISSVENEKLCSGERLEPATPSLKQNFNKRSRNGIDVDQRPLKRTKIKKHRHRPKVAGAGRKRRVSSSLEEKEKRKNVTKTQPKEKEKSKYVRRSLKTSTTEHHTESVAARSELLSVVNQPSKENYIEVMQPNASGELDVVIGDQERDDKMMLPTMSELAASHGSDDNKATFWDLSLEMKSKEQCTEKIRGQNLLEIKHSRFHKNLRSAGCHSRFSWNLRNASHNKNVEPSFPKEYKRKRMRRKPKSKISILLRFLDLYSHPKKKRCKGLVQRIHPIYNHKLNSLLNRCTFELAYMSIKGQPAEEESHAKAVSATSVAGLSIGQTTGYGMPTAPLVQDQPGLAPPIRGIGGFALGMVQSQIWCPRVSQLSAPSYPLPVIRPPGQGCPLMAWDPPPPHFVARPAYGLGFPMPPLLQQFGRRPVGMLLQSQVGLLRGLPAPPRPGMLLPRLGMPPPGCGPFQPGMPPPPPNPQLKNQQQCLLGPKKRFNNRKHKNLLIDDIGEGRLDVCDADAIVSYQDPPTKIEGSSAIVPYQDPSSTKIGGSSAIVLHKHKKKKLTAQVLLDKETIRQWNLLVKIDNVLGEKEEDKEREKKWEEEVEIFHGRIKSFTSRMHVILGDRRFKPWKGSVVDSVVGVFLTQNVTDFLSSAAYMSLASKYPIQSKSNQEASDDELDYKNSQEPIGDDIVCTGTTENLHANGYFITEFEEGTSVDIVEEISLVDVKDIGNAILQKQSYRQEIKALTHENPENASRGSDSRNPISGSERSNSLKATFEMPCSSSSACHNSYNSHLMEDHRAENVVEEKASPDMDFLPQDCSLQAEVGITSILDAQSVLHDEKLNMPEEVVWQDSALNPIAPATSMKNKRSGLVPGETRKTTKGTKNVVKEKSYWNDLGRKYSRPRSSAATDSIDWEAVRQAPETEIADAIKSRGQHNIMARKIKKSLNRILDYHGSIDLEWLRHAPDDDVKVYLLEIEGLGLKSVECLRLLTLYHDAFPVDTNVARIAVRLGWVPLEPLPGVLQLHLLEEYPVMDTIQKYLWPRLCKLDQKTLYELHYQMITFGKVFCTKLKPNCGVCPMRAECRHLASAIASENLCLPGIPKRGEERSKVPNMSLESSAVDANDALIVNPTAVSLSGYVKASESKFETQSCEPLIEEPKSPEPVADIEDFEIANGIDINDGEEIPIIQLSNEPFRANVQYFMDEYRNNLQTDSSSRALVPLSVNVDSVPVRKLKNISRLRTEHQVYEIPDDHELLIGLPRPDRNDQSPYLLAIWTPGESPASCQPPEKRCNSQGAELCKDETCFYCQSIWEERTETVRGTILVPCRTAMRGRFPLNGTYFQVNEVFADHESSYNAIIVPRSSIWYLRRRIVYCGTSPNAIFKACSLKEIQENFWKGFICVRGWDAKTGAPKPLAKRFHCPPSKMVKNSSKS

>Sl_DML1

MYGENNIKTCNDVSTDDIDEWSNVSFGHLLALAHAAGSTAVTENANEEINLALNGSFNSLISSQDADGSSTCSRFPFNLNSPTRMTDEDSSSNNAFPFEPITPYQIKKKGPASDAPGLDINATPIPRHVQSSKDTLKRAEANDLQQNTEKSGLVLNISELSDNMIDKVVDQDAEQNNTPQQKRRKKHRPKVVIEGEHKRTPKPKIPQQHSSMGTKKEKGKYVQRNKIEDPPGTPSDEVNDMTKHEGHLPSSAKIQRARRTYIRRNQVKKFAPKPAEEGSIDPPNVSRPRRYPRRSLNFDSENILSDENSLRWPSSTVEDLHENQSNSSVHPGKGIEASTAKTRLGSVYDLKCSNQELKNCQTHHEMSHTDPFTLKKIGLNHSKFTMNKENGISRGKCKIVFSDETHDKQASILEMTPKSPNSSNCSSSACLIPETPERALKRRRSLRTDQAKLYSTNVRGAYFNSMQAYQAILPATEPYAQSTQGMHFPIIFKKKRTEKGHPSATSYSKPFTCEINYLSLSQSNIGLSQASTSATDNANNLMPNRELVPAFVEAEGLRRKRSKSISKVRDLASLLEICKHFPTSSVKETMVSGFGERYENSDQPNTCMEALVADTRTIMKTKKRSKRSIPVSSTASHMYARSQFPTNARGSIPAITWRSPVDEIAERLQHLNLNRESIHPYQYEENALVIYQRDGSIVPFAGPFVRKRRPRPKVDLDDETTRVWKLLLQDINSEGIDGTDEDKAKWWESEREVFHGRVDSFVARMRLVQGDRRFSPWKGSVVDSVVGVFLTQNVSDHLSSSAFMTLAARFPLKSDISVKKNEERTGIIIEEPEVSTLEPDDTNGWHDYQSTQTTLGQKFFTISSTESDDEKTAVHSSESSENSTNCTSSTENSILQQPGSSRESSCVHHESTTYGSATANAATSFLGDQVEPDDLLSSQNSILSSQDSANFSVVQTSEGTESSNFSGSASFLKLLQIAGTSKSHGVQDQRSENILLEKNINVQLKHVACCSHIQKDGENHRGSIGNDCPCSYLGPCTMSNSGAQQAKFKSDLEEAAKFSDPSGELGDPEQSKSSAEPANQRVAEAPKAPTFSEAIDVREEVSVVVDSSKSEHTVLRSNSNNGKIHAGSTLDGANHNTKAKKEGPGKEKQNVDWDSLRLQAESNGKKREKTANTMDSLDWDAVRCADVNEISHTIRERGMNNMLAERIKDFLNRIFREHGSIDLEWLRDVPPDKAKEYLLSIRGLGLKSVECVRLLTLHNLAFPVDTNVGRIAVRLGWVPLQPLPESLQLHLLELYPVLESIQKYLWPRLCKLDQRTLYELHYHMITFGKVFCSKSKPNCNACPMRGECRHFASAFASARLALPAPEEKSIVSATENNAADQNPFQNFNQQPLTLPQANQTPLEHPKLINSAPIIEVPATPQPIVEEPASPEPEQDAPEIDIEDVCFEDPDEIPTIELNMAQFTQNVKNFVQNNMELQQVEMSKALVALTPAAASIPTPKLKHISRLRTEHQVYELPDSHPLLEGFEKREPDDPSSYLLAIWTPGETSDSIQPPGRQCNSQETGRLCDDETCFACNSIREAHAQTVRGTILIPCRTAMRGSFPLNGTYFQVNEVFADHESSLKPIDVPRNWLWNLPRRTVYFGTSIPSIFKGLTTESIQHCFWRGFVCVRGFDKKLRAPRPLMARLHFPASKLTRTKGKPDEN

> Sl_DML2

Fine modulo

METGQGSSWIPATPGKPSFAESPPICSTGQENQQAQVDLSDLQRKQAVEHANGSTAEAQNAVEHANGSTAEAQNAAANRGSTSSVEDQCFTTSEAVVGTKSEMCGGGINMYNNFPSDNVELWSSMSFGDLLAMAHAGGSGTTPADETAYSVKSSFQPLINTQNADESSILSSFPFNLNSPPKMTGATLSSNIQFQFEPVTPDMMKIKGQASNASNLDINVTTAARVIQSNEDIIKGAEANELQQNKEQSVLILEGKLDTELNNTPEQKTRRRKHRPKVVVEDKPKRTPKPKIQKQPGAEETKTEKRKYVRRNKVGEPTATFADEVSNTICHEGKPPSSEKTPTAKRKYVRRNQVNKSTEKPSEEGSSVTIGTPAATSTEEVKNTTFHVGKAPSSEETPTAKRNVRTNQVNMSMEKLSEEGSSGTNDPSEVPHSRKSCRKSLSFELESQASDEYSSYRPSTLDLHANNSGSTAQSVQLGQGKETTSEETEMGITHNITRSLNQEVRNYLSQPRMQYPSPPTPDKVGWNHDKTMVGNHNESTRGNSRIIFSDVTHDKQASILQMTPQSLNSNCGSSSCLPHGKGLKRQHSCRTDEAQFYSINAKGTYFNSMQAYQAILPANKPDVYSNVGMHFPAIYKKMRAEKGHISTSSYIKLFTGETNYVSSSQCYISGSPSNNSATNIGNYGMWNSNVMPAFVEAERLRNKISNGPTQVHDIASLHEIYKQFPTSTSKELTKYGFGERYKTSHLSSACMGTPIADTQAATKKKRQSKKSILVSSAASNLYTHQHVAKNARGSLPALTWRGMSPIDEIAERLRLLDLNRESSQNQGPHGITYHTKFQRESALVLYQRDGSIVPFGSSLVRKRKPRPKVDVDDETDRVWKLLLQDINSEGIDGTDEDKAKWWEEERRVFNSRADSFIARMRLVQGDRRFSPWKGSVVDSVVGVYLTQNVSDHLSSSAFMSLAAHFPLKTDSTQKHEGNTGIIIEEPEECATDPNVSIRWYEDQPNQSTHCQDSSGVYNTDSNEEKPAVNDSESSENSTECIKSAECSVILQSDSSREGSDLYHGSTVTSSQDRKELNDLPSSPSSVVSSEISAVIQASEGTDSSNFCSSTSFLKLLQMAGTSGAQGTRCTEHLQEGENVPFLEKELISPKKSVLSAESAHSALYTTPQNKLDIETMTDAEDNVELQFPTEDSNSNVQQVPEAPACSETIVNVTERASIVFDSCKPEQRGLESSLKNDSNHVRSKVDKVNDNPSKAKNGQLGKEKENIDWDSLRLQAQANGKKRERTANTMDSLDYEAVRCANVNEIAHTIRERGMNNKLAERIQAFLNRIVSDHGSIDLEWLRDVPPDKAKEYLLSIRGLGLKSVECVRLLTLHHLAFPVDVNVGRIAVRLGWVPLQPLPESLQLHLLELYPILESIQQYLWPRLCKLDQRTLYELHYHMITFGKVFCTKSKPNCNACPLRGECRHFASAFASARLALPAPEEKSIVSATEQKATNNNPRENFTHLPLPLPPGNQQPVENQKLINSAPIIEVPATPEPIVEVPSTPEQEQIKAPEIDIEDAYFEDTNEIPMIELNMAEFTQNVKKYVENNMELHQVEMSNALVALTSEAASIPTPKLKNVSRLRTEHQVYELPDSHPLLEGLDKREPDDPSSYLLAIWTPGETANSMQPPETQCNSQESGELCEDETCSSCNSIREAQSQTVRGTLLIPCRTATRGSFPLNGTYFQVNEVFADHDSSLNPINVPRDWLWNLPRRTVYFGTSIPTIFKGLNTESIQHCFWRGFVCVRGFDHKTRAPRPLLARFHFPASKLNRTNGKTNEDKGVAS

> Sl_DML3

Fine modulo

MNPGRVFSTPQENGGVQNGDPRIPFSQQKPVLPLPDLVPAEMQRNQIEMTGWPDLLGMYGDFLLMPASETGVVQNSVTSVGWDKGSTGHWSDVVVRNRSSEIDTYSCGNIPDQSKPACTRVNSLEELIGMKNQSNRISTHGRSSNSTRSDIPILRNSYAQVDRRHEQTQLKAAGQTVLNQSQLFKSPNQMVDCYNRHLPLDGMRSPYQVNRSLISPVAQDAGTSPSTNSFFTFAPVTPDHNHFNDNQHFERQNVPIQERSSLEKDGQENVLGSMKSKDNHSDKLLQRVTDSVVVNSPLTEKVDNGNVGNVDIDLNKTPASKTPKRRKHRPKVVIEGETKRTPKRAAPVDGTPNENPSGKRKYVRRDGLKASTTEQTEVNESAAQRNSTPNENPSGKRKYVRRKDPNASTTQQTEVVGKDKVPDAGESEKTCRKMLDFDLEDITKDESLPSTNIHHPEKHQQKKETFDLNLNSQDMELSLAIMEATAISAGQKQRKEEIAEKLLMEKPQELASPLPSANQVTRNNQALNALARSLSMRTVTRYPNSIQLYEPRQLALGRMPLLLRDTAYTDNDGRGSKRDQCPSSPFQPRTFSQMGSVCSEMLGNDNCRRNCSTSSGIPSYTAAAIHDSTKFPSSSLSINRYNRASEEGSRHCASPMVVKHNLQKQTNPSQMHSYAQPIPQHIPQQTAEIHGSQVQATIRNWNHQYQLQSLAMVVQNIERRNSHKKMPAQQNMGKTSPNELSNYVELLPRENKNSRADQHHLTKARGLQETHRHAVSVDTGLLQGLQRHVVSVDTGLQGTHRHAVSVDVITQQLERLFISNSKKNAAQVEQKALVPYKGSGTIIPYEGFDPIKRRKARPRVDLDPETNRLWNVLMGKEESAETMDKDNEKWWEDERKVVRGRVDSFVARMRLVQGDRRFSPWKGSVVDSVIGVFLTQNVSDHLSSSAFMCLAAKFPLPTSTKNTLSQDGCNIVVEEPEVEIIDPDGTTIYHKARLQRRMENHTHTSRAYLVSEHDKRVDEEVISLQNSPDSLILQANEELRSSSGSDLESEDRPSSPNLNKDRTQASHSPPTKWTAAFQEYQSHFMRNGISEKLPVFGNQKIETVADMGRHNENLDAETYLHGYPINPHIQVQEIPIRSASNSWLNMTPEFGKHETACHEKEIDMSKSMKQIAGSSSPLIAQRTTHPFIHAPRMGEIGGVEMQPGKVDNQHSVSSHQNEMAMASQLESSCIRQSVNHSEAVAKGQEEGQAYPSSKQPSITGTSISKTRKRKVEEGDKKAFDWDSLRKEVQSKSGKKERSKDAMDSLNYEAVRSAAVKEISDAIKERGMNNMLAERIKDFLDRLVRDHGSIDLEWLRDVAPDKAKEYLLSIRGLGLKSVECVRLLTLHNLAFPVDTNVGRIAVRLGWVPLQPLPESLQLHLLELYPILESIQKYLWPRLCKLDQRTLYELHYHMITFGKVFCTKSKPNCNACPLRAECRHFASAYASARLALPGPEEKSIVSSAVPIPSEGNAAAAFKPMLL
PPAAEVRMAYPYAPIEAGDLPSFLEKSMPIPQEMTDSLNREATVVTNNCQPIIEEPKTPEPLPELLESDIEDGFFEDPDEIPLIELNMKEFTTNLETILQEHNKEGDVSKALVALNPEAASIPTAKLKNVGRLRTEHQVYELPDSHQLLEKWDKREPDDPSPYLLAIWTPGETVNSIQPPETKCDHSGLGNLCNETTCYSCNGIREANTQTVRGTLLIPCRTAMRGSFPLNGTYFQVNEVFADHKSSLDPIHVPRKLLWSLSKRTVYFGTSVSTIFKGLSTEQIQYCFWRGFVCVRGFDREMRAPRPLIARLHFPASKMVKNRSDDKKKEGTAAEKVAGFNSPISVHTK

> Sl_DML4

Fine modulo

MKNWGKLPITSTKLNCFNPTYFTAPFSHQPPFFAVEKSGMELGNADQEELEKTGFFTPITPFKCISAATEFNSINAGLNNFWSTNSSYNSQKKDEEVSDVIGGRSEFLSRHLDGGSESATVAPSTPMTKANPRKKQCGSVDMNERPLKKPRMRKHTPKIFDESKPKKTPKPKIKPSIPKSGNSKVSAKIKKKIEKDHDKVSVDLSGDAYMQTPNSKIPETIPGSLVLQVSTPQPETLEHALSAISPPDFEHDNLTDFGHVSKSCKRSLPFNLENENDFLSIEAAEVIRYHVTQSYNKFLTNPLDGFNEQAVDTNSSIGLEDNLEAESQPNQGNDPSIYQDDQRACQHHFLKVYERRKTQIDLMPLAGNEAHVYPNNHGTISACRDHSVKVYKRRTIGNTGAVPLAGNETTSQNDHKSQEKGGSRLDFLESYRTSNNIGTADYADKGWNPLDTNTKSKVTSKSSFTICKRVTRRDFEIVNGAKNTCFSNMKSGQKGKFRSHFVVMVLPNKNQIAAAMADIESFECVFSLSPMVKSRRRRLIHPKRTKSAHREEYTKLEPLPLSARREENTELGPILPMNALTLSPLVTSKRKRSKNCKRSTAILDPLCLKSGLSAINGFESFVDKWSQISACNEIQECSQHEGLWPQTDKSGCNDIEECPQHEDLLPRTNKSTCSEIQEYTQHENLLPKTKRSTKIITVAAIIRIFKKIKICDTRTYKKKTTTSNNNDQLVLWNPDGSMIVQNPKAKELPRVNDDQLFLRNPDGSMIVQKRKAKKLPKVNDDQLVLRNLDGSMIVQKRKAKELPKVDLDSESERVWEQLIENGGIDEDPDEKKNIWWKEQRELFKGRADSFIARMCLVLGKRTFSPWKGSVVDSVVGVFLTQNVSDYLSSNAFMLLASAFPLQNSREMTTQQEQALAITHVLPDSDEIFVSNTSESMQEENIGNQNTCGSQSSANCGNPEVISFAKSCDLVNETGDLPDEMSREKNKLPKREFDWDRLRKAYSTGTYTGSTESNRDSVNWEAVRHADVKMIFEQIKCRGQGNVLAAKIKNFLNRSFEHHGSVDLEWLRDVPKEDVKEYLLSIYGLGPKSTDCIRLLSLRHHSFPVDINVARIVVRLGWVPLQPLPDGLQMHLLEKFPLESSIQKYLWPRLCELDVSTLYELHYHMITFGKVFCTKKKPNCDACPLRTECRHFASAFASTRLRLPGPQQKGEADSKQPDTVDDVLNMCVSLPNLPHSSESFSHSSFQTQHYEPIVEMPESPEHRPLELLEQDIEDFSYEAEHEQEIPTIKLNTKAFGENILSFIDKSNKDFKEIVLSFIDEISKLQSDEEVSKALVLLNPKSAACPARKLKTETRLRTEHLVYELPDDHPLLSGFEKREPDDDCPYLLAISQTEQVVHKQEKNGKDSSSNELEKCSDNTNYFPKDEIIYGTILIPCRTANRGSFPLNGTYFQINEVFADHESSLNPIPVARASIWNLRVTTLYCGTSVSSIVKGLSTMDIQKCFWKGFMVVRGYDRKQRAPRPLHARFHRKGNIEKFDDE

>Br_demethylase1

MDKQQAREETTWVPQTPIKPITPIYPDQIQTEERRFAGNKDKSGLDHLSFGDLLALANNASSVFLSRQNGIDKEHVIKTPEKPKRKKHRPKVVREAKPKRDIKPKTPKKPAAAVEGEESKTPKRKYVRKKKEAGEDQEEYTPVEESSGAAAEDGEASDHDGKKPCRRALEFDDQSLKPQNGEAQHRDETKQDQDLQESHMAAVPSTPKRKRSSQSRRMGKEMKNNEAQATKRRQGKEPTRSNIYFSGQQYEQVFADNEAQWLFSTEWLQKGMRSRSTTGQQLVTQENVSAFESSCRVLTFQGRQSFESNAHLDKIETPTKKRTTGHARFRGMSSTNKASEQSQAGWYSRQTQVASSRKKRTTKSQTKQLTLLPNHCQFPPSFAAGLAPEAIWQQRHSIEAISELMRLLDINREYSETALVPYGMNSSYSVGNQIVIFNGGAGAIVPSTRVKKPRRERAKVHIDNETDRVWKLLMESIDSEGVDGSDEKKAKWWEEERNVFRGRADSFIARMHLVQGDRRFTPWKGSVVDSVVGVFLTQNVSDHLSSSAFMSLAAEYPVPFVPSSDFEVGESSMPSIRITYLDSDEPISNPPVPSETSAALDNTQPDEEKEYVNSKDTSRSSSEIASSGNESTVKTTDSKAQVDSDRTGSSVEVSKTVLIVQELFPSEDSVLTCQNSLVSETPKMTERAGSSSEINSEAENCTHFVKLLESQGSAQLQEKESDVITADTVLVEEASQKTQCSSSPGSLQISPNTSPGDCSSEVKDFKSLKGKGKYSDDEPCCFFGDFLSVQKPEIPESSSSVPSTKLVIETPIPDINESTNCLDVQEGTEKQQPGPDSSSKKISPMDKATFNADGKKILKEVDEEFDWDSLRREAEGREGKREKTARSLDSVDWEAIRTADVNEVAETIKSRGMNHKLAERIQGFLNRLLTDHGSLDLEWLRDVPPDKAKEYLLSFNGLGLKSVECVRLLTLHHLAFPVDTNVGRIAVRLGWVPLQPLPESLQLHLLEMYPVLESIQKYLWPRLCKFDQKTLYELHYQMITFGKVFCTKSKPNCNACPMRGECRHFASAFASARFALPGPEKGMERPDVPLQSLPEPLRRQQGLEVVNHSEAANRVTSCEPIIEVPASPEPECAEVSMADIEDAFFEDPEEIPTIRLNMDAFTNNLKKIFEHSKELQDGNMSGALVALTAEAASLPMPKLKNISQLRTEHQVYELKDDHPLLAQFEKRETDDPCSYLLAIWTPGETVDSIQPTRSKCIWQEAGKMCNEKTCFSCNNIRETQSQTVRGTILVPCRTAMRGSFPLNGTYFQVNEVFADHESSLTPIDVPRDWLWDLARRTVYFGTSIPSIFKGLSTETIQQCFWRGYVCVRGFDRQTRAPRPLIARLHFPKSKMKSQVNPDDA

>Br_demethylase2
MEKSKDLDNFSGLVVQGTPVKPERPLKFYVRRKPPKLLNKLQNIDDSASSVSSDFNTNNTTKEESVKKSEEPETFKVDLQSLHGTQGKEKSKEETEEKNAKSTILQDDSQHVNGERKKNYSQKISKKRFHRPMIMEDGKKPRNRANLQMRTISNKRSKKEKCDEEEYIVELQTPEKQSSPKRRKNKAKKSVARTLPFDEEVISGCLEFSRSFWPSFPKGKRRMTTLRRIDFHVLISPISFPMPIWKKQSKRSSRKKNMTRWTMIALSYECIEETLSLVEIHPDDIDIKKMPPKLSLMHSNQKITDGSNEHLKKAKKIRRQDASVQTKDLHTEEKKVMNNLTLQLNYQREHNLPSLADVPLHKEDILMRSLTNITPEQSMKKTTKGVAKLIKEMEKLNINRRVTTLGKAKKKLVIAKVNLDPETIKEWELLMENDLPHQSYSNEENTESKWKGEREIFQSRIELFINRMHLLQGNRKFKQWKGSVVDSVVGVFLTQNVSDYLSSNAFMSVAAKFPVDAKESLESLAYFIEEPQEVNNLVANGQIPIQNEKDDAKSSTGSVSLQENLEQHEKDVKRKNKKTGIMEDESVDWESLRKIYTKEGFRDTIHMDTVDWNAVRLSDQQVLADTIMKRGQHNGLARKILKFLNDEAKQNGTVDLEWLRDAPSDLVKRYLLEIEGIGLKSAECVRLLGLKHSAFPVDTNVGRIAVRLGWVPLEPLPDGVQLHQLFQYPSMDSIQKYLWPRLCKLPQETLYELHYQMITFGKVFCTKVIPNCNACPMKSECKYFASAYVSSKVLLEGPEEKTQESQESQEFQEFQESQESQEFQESQESQTSYCHDNDAKMTSKINSIEECVSTECSNQTNCCEPIVEFPTSPAREIPELPDIEDTPCRSSCRSNAAIPGMDIDIDAFKQNVVDVFKKIGTMFNGSDDEISKALAVMTQENACIPMKLPRKTKYYDRLRTEHVVYVLPDKHELLNDFERRERDDPSPYLLALWQPGETSNSFMPPKKKCDSDGTNLCVIKTCSYCWNLREESSNTYRGTILIPCRTAMQGGFPLNGTYFQTNEVFADHETSLEPIVFSRELCNGLEKRALYCGSSVSSIFRLLEETRTRLCFWTGFVCMRGFDRKQRTPEGLVRRLHTPPDERGEKHMRD

>Br_demethylase3
MNSRGDLGDGYVPVPAENQFMGSWTPITPRKPMQGGSSGVVVDGGGQDRNYYTGEREDPLGRSNVASNSQGGCSNVFELDDLLDTDQMPMSFTSLLSGGDHLFQAPQCGTPVSSRPLYNLNSPPTSEAVEYICGGSVQPVPSTPSLSRTGRDNGFLETMITRTTGQTSDNGMQSVVASSVVNSTEVAEQKDGSRQNDLGFDLNQTPQQKPSKKKKKFMPKVYVEGKPIRKPRKPATQEAVKPKATGGGKRKKAQKTNLKESAANKPAIGGVMSNTSLEVTGKSCRKALSFDLEKTGDVGLDDSGSEIFQNTSGSNSFTETRDAAGGTSGSWLDSVTQVDQTNGLVAANQPLEASTVVTLPRGSEVNHSRMLARDQQPELFTGNQQRQFPMENQQRQWPMENQQRQSPMENQQAWLHMKNQLCGFPVGNQQPRLAMGNQQPMYLMGTQRSALASGIQQPGGLQGNNQPMFLNQQSQQTYLPAENHQYGSPSGMQQHVMSTRGQQHGMLLDNQRSHQQPGSSVRGQQTCSPAGNQQYGSASGMQQHVMSTRGQQHELLLDNQRSHQQPGSSMRGQQTFLPAGNQQHGSPSGMQQYMSTRGQQHALLLDNQRSHQQPGSSMRGQQTCLPAGNQQYGSPSGMQQHVMSTREQQHGMLLDNQRSQLLMRNQQPGSSMRGQQTCLLAGNQQYGSLSAMQQPVMSPRQQQHGMLLENQESQFLMSNQQPGSSMRGQQPCVPLMNRQLGTPKGFTHLNQMVAANMSSSGHRPHPHSQTPATNLYMESVSRTLNGSAGTYQRSSIAGYGSSQQDIYQGNERIPSHERSNAEYFDLRKKAPSQNSALPTPDNAKDVEARGLKRQHDRAMGHMQNTVAHWPLLQQIAQSQDVERQNISTSAKHVDAAKKMRIQKPVQEKVHGVAPEVIDIEDYPTDGARKDKSGVPKTPAKKGPRGRKKAVPPPAHASGSCLAQNSADTEKGIVPETPARKGPRGRKKIVPPPPNASEIQVYQPTPAKKPSSRSKAKEKGMKSKQDSGKARGQSGELLREDCIAEIIYRLQNLYIGDESRKQEQNALVPYKGDGAVVPYEAKTKKPRPKVDLDDETTRIWNLLMGKEGKEGEEEMNKKKEKWWEEERNVFRGRADSFIARMHLVQGDRRFSPWKGSVVDSVIGVFLTQNVTDHLSSSAFMSLAARFPPKPSSKPEDERNIRSVVVEDPEGCILNLNDIPPWQEKVQTSSDTQVSGVDSGSKEQQRSCSNSGIERFSFLENSSQNLEEEVLSSQDSFDPASWTSQSSGRVGSSSGSKSDAEFSTTRSETKAASGSAQSVQIGSPNLSVERSLLHQESGDVQIQETSNVAQKKPDMTADLVDIEDCGMNFVPINFTMAREKKGTQAAGKKPTSQWDSLRREVLERKGKKERSKESMDSIDYEAIRRASVYEISDAIKERGMNYMLAVRIKDFLERIVKDHGSVDLEWLRDVHPDKAKDYLLSIRGLGLKSVECIRLLTLHNMAFPVDTNVGRIAVRLGWVPLQPLPESLQLHLLELYPVLESIQKYLWPRLCKLDQPTLYELHYQLITFGKVFCTKSRPNCNACPMRGECRHFASAYASARLALPATEERGLTTATIPVPPQPFPPASIPMMELPPPLETFLTREVPSNGGSSEPIIEEPATPEQEVTEITESDIEYAYYNEDPDEIPTIELNISQFGETLKEHMKNNMELQEGDMSKALVALDPSRTSIPTPKLKNISRLRTEHQVYELWDSHPLLAGMDKREPDDPSPYLLAIWSPGETADSPGQKCGGKASGKLCFEEACSECNGVREANSQTVRGTLLIPCRTAMRGSFPLNGTYFQVNEVFADHESSLKPIDVPRDWIWDLPRRTVYFGTSVTSIFKGMSTEQIQYSFWRGFVCVRGFEPKTRAPRPLMARLHFQKNKVKKNKT

>Cr_demethylase1

MEGEVRKYRERQARVQRGLVIQEQPIFQNMQHNQVPESDRRRLSLENLPGLYNLSCSQLLALANNTLGTSSSVGASPSSQYLMDFWIDSSSMESNPLISNPGSSLGINTGIPGERTMQTPQHDVPTPQKFLCDLNLTPEEMVSTSFQPTEPEIPPVTLDTPGTRLSETDQKPHDPIKKSILETGSPSGVKRRKRPRIDENAQLKTPASKRKKIRPKVVREAKTNEASSKSGIKKPSVSAATATKPSEESSYIRPKRSRKRSVRLDFDLQDEDQEYCGLDFPSEYRTGGEGPTLGLFGSIPKGRRKKRIHANKRQQKINPSSERGNDCLSSTLSLVNTGPAAFSESEEDIASNSHIYQSLGRKRSRLVTIPRNFGSLAKLLESIVPSKCSLPLLNVSEIQPKVPRKKRRQRDPLASQLNARILHREWQSPKPKVTSFAEMWIRSMELDSVTKKLQELDINKKFQESALVLYQTLYEEQRVLIKYSKKQLPKVDIDPETNRVFKLLMSSINNDGVDGLDEDKRKWWKDERNVFHERANSFIARMSIVQGDRTFSRWKGSVVDSVVGVFLTQNVADHSSSSAYMDLAAEFPVNWNFNKRSSLEEWGSSATSNPRIRKSTCVIIEEIDDDDDEDGTDAVCAHESSKTSDSSMSSTNQSTMTLLDPSAGTSTSSHCELNLNEVPHEVENCDVDALTNKFYEEIQVQHMSSHQQELDSTLQAQDQEKNTRKEVVKNKEKKKPTTSRPVGRPPKNKVKESKKKSKKPAKSKLEDSFDWDSLRKQVESGGKKRERTERTLDTVDWDALRGSSVNKIAAIIIKRGMNNMLAKRIKDFLNRLVEEHGSIDLEWLRDVPPDKAKEYLLSINGLGLKSVECVRLLSLHQIAFPVDTNVGRIAVRLGWVPLQPLPDQLQMHLLELYPVLNSVQKYLWPRLCKLDQKTLYELHYHMITFGKVFCTKLKPNCKACPMKAECRHYASAQRARLALPEPEVSDRTTVMLYERRYKRNPFVVNFRPSLLFSQENEQEAQRCEPIIEEPASPEPEYTMPDIEDYPWDNNNVAMSTILENDPWEDKDIIPTIMLNKEAVTSTDLVVLSTQAAAIPTRKLKIMEKLRTEHLVYELPDYHSILQGFERREDEDIVPYLLAIWTPGETENSIQLPKQRCEFQGDNSTLCHEKKCFECNKIREEQSQIVRGTILIPCRTAMRGGFPLNGTYFQTNEVFADHGSSINPIHVPRELIWKLPQRIAYFGSSVSSICKGLTVENIRENFKSGYVCVRGFDRVNRKSKTLAKRLHCTKSKHN

>Cr_demethylase2

MNSRADDPGDGYFRVPFENQTQKEFQVRGSWIPFTPNKPPQGRSSLILDERVIHQDLNGVPSCEFEDMGFCSSGAVHDLNHALQGMDQNGVYDHGAHQGINNLQGSYAQARCNTERDLLGRSDATSPLPPVIRNITGNVELVNGKFTSDVGMVNGSFTQSGTSQAGYTEFELDDLLDPGQMPFSFTSLLSGGDSLFQVRQYGTPACSKPLYNLNSPIRREVVGSVFENSFQSVPSTPSLCRTGENNGFLEKIVTTTGHEITEPKSDKSLKSIVDSSVVNSTEVAKQNDGSRQDVLEFDLNKTPQQKPSKRKKKFMPKVVVEGKPKRKPRKPATQENVKSKEPGSSKRKKAQTKNLKESTTKKTARDMSKGSPEVTLKSCRKALNFDLENSGDARQGDSESEFIQKNSGSNSFSEIRDAIGGTSASFLDSITQIDQSNGLVATNQPLEASMGNQPDELPRMLQENHTQILSRDQQPDLLIGNQRCQFPVATHNTQFPVGNQQAWLQMKNQLIGFPFGNQQPPMTIRNQQPCLAMGNQQPMYLTGTPQPALVSRNQQLGNLQGNKLPIFLNQQTCLPAGNRQYGSPADMNQLVMSTGGQQHGLLMKDQHPGFLLRNQQPGSTVRGQQRCVPLMNQQPGTPKGFTHLNQMVAACMSSPELQRHSQLQIPATNLHSKSLNRNAGTCQRSGNTEYGTLQQTHQGNEYIPSHERSNGEFFDVCKKNLSQNSYLSTPVMAKFEEARGSKRQYHHAMGQMHNHDLNLAGPWPLLQQIAQSQDVERQNSTTSAEYFDAAKKNKIQKVVLENLHGMPPEVIAIEDNPTDGARQDKNTASIIKTPSKPISCRVQKSGADEKFIVPKKTARKGRGRRKQSVDSPPHASAIQIWQPTPPKAPSSRSKAKEKGRKSIPDTGKAPSSRSKAKEKGRKSIPDTGELLCEDSITEIIYRMQNLTLGENSRAKEQNALVLYRGDGAVVPYESKKRKPRPKVDLDDETTRIWNLLMGNGEKEGDEEMDKKKEKWWEEERRVFRGRADSFIARMHLVQGDRRFSPWKGSVVDSVIGVFLTQNVTDHLSSSAFMSLAARFPPKLSSSRKDEKSIRSVVVEDPEGCILNLNDIPSLQESIQNRSETQVSEVDSGSKEQQIDCSNSGIERFNFLNSSQNLEEEVLSSQDSFDPAIFQLCGRVRSSSCSKSDANFSTTRCETKSASGSSQAVQTESPNLSVEICLQENERLLPYERSGDIQIQETTNVAQKKPDLDKPMNWKDYLPFDQPSNDVNWQKGGPTNPSSSYEQSMIQQPHVLDIEDFGMQGEGLGYSWLSISPRVDRGKNRNVPRRFFRQGGSVPREFTDQVIPSTPHVIPGMGFSVSASTHQVHQGDAQQQHEMNKASHLQKTFMDLLNSSEECLTRQSSTTQNITDGCLPRVRTAKDVAESNSRNKEQTTVEYKETNATIVREMKGTLADGKKPTSQWDNLRKDVGGNEGRKERNKESMDSIDYEAIRRASISEISEAIKERGMNNMLAVRIKDFLERIVKDHGGIDLEWLRDVPPDKAKDYLLSIRGLGLKSVECVRLLTLHNLAFPVDTNVGRIAVRLGWVPLQPLPESLQLHLLELYPVLESIQKFLWPRLCKLDQPTLYELHYQLITFGKVFCTKSKPNCNACPMRGECRHFASAYASARLALPAPDERSLTSSTFSVPPESFTPTAIPMMELPSPLEKVLTRGAPSNRGNCEPIIEEPSSPEQECTEITESDIEDGYYNEDPDEIPTIKLNIEQFGMTLREHMERNMELQDGDMSKALVALNPTNTYIPTPKLKNISRLRTEHQVYELPDSHPLLSGMDKREPDDPSPYLLAIWTPGEMANSAQPPEQKCKGKASDKMCFDETCLECNSVREANSQTVRGTLLIPCRTAMRGSFPLNGTYFQVNELFADHDSSLQPIDVPRDWIWNLPRRTVYFGTSVTSIFRGLSTEQIQYCFWKGFVCVRGFEQKTRAPRPLMARLHFPASKLKNNKT

>Cr_demethylase3
MERQGREESSIQQPPWMPQTPMKASSPICSNIVEERAHRNQEEERRFDGNKGVGGGLAHLSFGDLLALANSAAFCFSGNTEMMQKDNEAVESLSSVSNNVAEEIINNVAEEIIKTPEKPKRKKHRPKVLKDAKPKRAPKPPTPRKSVVADAQESKTPRRKYVRKKVQVDKNQESTPLDPSVAVETSTRAKKLCRRALDFEPEKGENHSNCNTKQADEMESVLVEKLLASGNHESNDFLLSVPTTPKRKRSQGKRKGKEPKNNGTDLEEVDISMAQAAKRRQVKEPTCRDLNLSGIQYNELCDYQKMHWLYFPNLQQEGMRSDVICSKSFTGQQLMDVSAFDSNCYSFTSQHSANGVLTTEKIREVTFQGRQPSEFNVLPEKIDTPIKKRTGHARFRNLSSMNKLMEVSEQLPSGYHCKPHQKILVDTRVTVSKNKRITKPKKSQTNQKTLLPNLCQLSASFSGLSPDELWKQRYSVEAINEQLRLLDINRENSDNALVPYPMKTKGNQIVLFRGGAGAIVPVTPVKKRRPRPKVDLDDETDRVWKLLLENINSEGIDGSDEQKAKWWEEERNVFRGRADSFIARMHLVQGDRRFTPWKGSVVDSVVGVFLTQNVSDHLSSSAFMSLAAEFPVPSVPTTNFEAGTSSAPSIQITYLDSEESMSNPSDNNQSSVILKNTQPDEEKDYVNSNETSRNSTETSSSAHESVGKTTEVKTYVESDRQGLSVEVDKKYQECFVLNLFPSEDSVLTCQHSMVSEAPQNTERARLSSEVNLEEEYRTSYMKLLHGVQVLQEESNQKNQYDTSRQEVGVSSNPGSLQVSPNISPSDCSSEVKDLQSLKGPTKSSDSNELCCCYRQDGDVLSSQKPVMPESSSSVCSTKHKEKLVTETLIPDINESTSCLDVQEGTEKPPGPDSRQHLDSSCKEVSPTDGATSKAKGKKKLKAKKEAFDWDSLRREAQGRVGIREKSTRTMDTVDWEAIRTVDVSEVAETIKSRGMNHKLAERIQGFLDRLVDDHGSIDLEWLRDVPPDKAKEYLLSFNGLGLKSVECVRLLTLHHLAFPVDTNVGRIAVRLGWVPLQPLPESLQLHLLEMYPILESIQKYLWPRLCKLDQKTLYELHYQMITFGKVFCTKSKPNCNACPMRGECRHFASAFASARLALPGTEKGMGTPDKKPLPLHLPEPLQKDQGSEVVKHAEQAKKFTCCEPIIEEPASPEPESAQVSIADIEDAFFEDPEEIPTIRLNMDAFTSNLKKIMEHNKELHEGNMSSALVALTAETASLPMPKLKNISQLRTEHQVYELPDDHPLIAQLEKREPDDPCFYLLAIWTPGETADSVQPAVSKCIFQENGKLCDEETCFSCNSIKEARSQTVRGTILIPCRTAMRGSFPLNGTYFQVNEVFADHASSLNPIDVPRDWIWYLPKRTVYFGTSIPSIFKGLPTDTIQQCFWRGYVCVRGFDRTTRGPKPLIARLHFPASKLKAQQANLA

>Cr_demethylase4

MVSPMDSIYPCMVEERSPRKQVEERPNYFSGHTSWASNASETTRAYDSGLMISDEISPIYPNTVEEQAHWNQLEQRSLDMSGLEQLSFGDFLALSNNAPVYCSGQTIPWASNALETTRNSEIMQQRNEAVNCLSTVSNNVTSVSFSGQTHLVSEPTTTRYTEMMKKGNEAVEILSSVSNNVAEEIINNVAEEIIKTPEKPKRKKHRPKVVKEAKPKRAPKLPTPRKSVVAHGQESKTPRRKYVRKKVEVNMDQESTPVEPPPVVETLTHAKKLCRRVLDFELENGENQSNSDTKQEDETESAPREKLLGSINQELKDFLRSSPSTPKRKRSQCKRKGTEPNKNGGSQEGVDRSVAQAAKRRQAKEPTFGDLNPPRFQYDEVCDYQNMHWLYIRNLQQEGIRSEPICSTSFAGQKHKDVSAFDSNCYSFTSQPIADRVLTIEEKREGIFQGRQQFEFNVLSDKIDTPMKTRTTGHVRLRNLSSMDKNMEVAEQLPSGYHSNSQQNNNNILVDKRVTVSKKKRITKSKKSQANQKTHLPSHCHFPASFSGLSQDELWKQPNLVEAITEQLRILDINRESCETAIVPYSSKNQGNQLVLFRGGGGAIVPVTPVKRRRPRPKVDLDDETEKVWKLLLENINSEGIDGSDEQKAKWWEEERNVFRGRADSFIARMHLVQGDRRFTPWKGSVVDSVVGVFLTQNVSDHLSSSAFMSLAAEFPVPAATSTNFDFGTRSMPSIQITYLDSEESMSSPPDHNQSSVTLKNIDPDEENGYVHSNETSRSSSEFASSSHESDGKTTYSKMYVESDRKGLSVEVAKIDQECLTLNLFPSEDSALTCQRSMVSDAPQNTEKAGSSEINLEGEYRTSYLKLLLGVQVSLEESNQKSQYENSRQEVGVSSNPSSLQVSPNMSPGDCFSEVTDFQSLKRPTKSSDDSYEPSCSYQQDGDVLSSRKPVMPESCSKKHKGSFQIPDLNESTSCLDVIEDTEKPPDPYSRPLRDSSCKELNPIDDVTLNANGKKVQKQKKEAFDWDSLRREAEGREGKRKKSTRTMDSVDWEAIRTADVSEVAETIKKRGMNHMLAERIQLTMNMKVMQGFLDRLVNKHGSIDLEWLRDIPPDKAKEYLLSFRGLGLKSVECVRLLTLHHLAFPVDTNVARIAVRLGWVPLQPLPESLQLHLLEMYPILESIQKYLWPRLCKLDQKTLYELHYQMITFGKVFCTKSKPNCNACPMRGECRHFASAFASARLALPGTEKGMGTPDKNPLPLHLPEPLHREQGSEVVKHSEPVKKVKFCEPIIEEPASPEPESAQVSIADIEDAFFEDPEEIPTIRLNTDALTSNLKKIMEHNKELQVGNISTALVALTAEAASLPMPKLKNISRLRTEHQVYEIPDSHPLLVELEKREPDDPCSYLLAIWTPGETADSIQPAVSKCISQANGKLCDEETCFSCNNIKEARSQTVRGTILIPCRTAMRGSFPLNGTYFQVNEVFADHASSLNPIDVPREWLWDLPRRTVYFGTSIPTIFKGYVCVRGFERKTRGPKPLIARLHFPVSKMKSRANPPNQSAV

> Cr_demethylase5
MKFSNHLDKSSFTIIPSTPIKPKQILNFYVRRKSIKSLHKPQESDDCISPKDSSKEEEIKEKYIFSKGDSKHVIGREKINNSKGTQKRKRFHRPRIMEEGKKPRNPAATRLRTISKTRKKKEDYSEDEVIAEPLTPQKQSFPKRSKDAKSKKKVVRSLNFSKKAPISCLEFNRICGPNFPKGRKRMSTVRRSDFHCLISPISFPLPVWKKQSKRFKRKKNVVRWARIALSFDQQEVLPSGADVSLHFEETLGHVSCVAPKRKNKSTKTIAEHLVQQMCYQKDHGLSSLADVPLHIEDTLMKSSSNVPQEGPIMKTKDIAKLIKEMKRLKINKRVTTLVGSSKKLVLAKVNLDPETVKEWELLMMNDHPNISYDDNETEAKWKQERESFKSRIDLFINRMHILQGNRKFKQWKGSVVDSVVGVFLTQNVSDYLSSNAFMSVAAKFPTDSMDDLEGLTYYIEEPQDANDSVMDDDQRPNHVGNDDGKSTDFVTLSADSIADVEVHAKTAKRKNEKTGIIEEETVDWKNIRKLYTKQGSRHKMHMDSVNWDGVRLSGQQAFETIIKKRGQFRVLSKRILKFLSDEAEQNGMIDLEWLRDAPSDLVKRYLLEIEGIGLKSAECVRLLGLKHSAFPVDTNVGRIAVRLGWVPLEPLPNGVQLHQLFQYPSMDSIQKYLWPRLCKLPQETLYELHYQMITFGKVFCTKVVPNCNACPMKSECKYFASAYISSKVLLEGPEEKTHNPDESFAHANSQDVVDDMTSNINVIEECGSPGCSDQDIYCEPLVEFPSSPRADIFEITDIEDTPCVNSYQPHARIPEIEFDLDALKKNVEDELRYSGGKTLSSVDDEISKALVVLTPENACIPIKLPRKIKYYDRLRTEHVVYVLPDNHNLLQDFERREVDDPSPYLLAIWQPGETSYSFTPPKKKCNSEGSNLCNIKTCSHCWAIREKIANTFRGTILIPCRTAMRAGFPLNGTYFQTNEANYESSIEPIVFPREWCEGLEKRALYCGSSVTSIFRFLDTGRIQLCFWTGFLCMRAFDRKQRNPKELVRRLHTPKFMQDDDDI*

>Cs_demethylase1

MGRTSSTNHLLSSSEDLFCSSTTISSEGKEAMGLKRKWYQNIKQDDASSFDLLEEFYSIYGSQMPQAEYFPKENSDKVQHFGPSSTYFNVTGETCKVSSLKENSCTSKARYQLPRPQNHSLFPRVHEGSVTPNKLQPFESSLATGQMKMTHTRFDAQDYVWTLGSWSHHCNRQSKYSHKQSLAVTDLQRVESSHRLPSSGAQVDKIKIQTTSSIQSLLYIVAAKLPNFLQLSTNAMIEEMKMLDINREGKISLYEKQNEIVTYKMQNQEHSALVVYRGDGSIVPFEGALDPIKKRRRFAKVDLDEETVRVWKLLMDNSNKELVEGPDEAKDKWWEEERSVFSGRTDSFIAKMHLIQGDRGFSQWKGSVLDSVIGVFLTQNVSDHLSSSAFMSLAARYPLKSKSLHESSVDEQTSLILNESQVTLCQAEDSVIWAKQISDQSICKQSCTTVCEIDQAEENFLTSSDSSGSKTAGVTSMRGYQCSVTSYSSKKIVELEDRRLTTEINTTVEACSLGNEKTADAAISSQMSVVSEHSINSLCPLSSENRMPCLKSNYGKDLSSKDICGNGCASSVEVKQITETNKLKSDFKIASATDSSDEKSEGTCSTSEEKYVCQREHNENPDSPKNHLKESPSQSSNQLQKISNSGVTEVECCKLCREATPFPYVYKQRDVYHTNERSHTLNLVSQTSVVNTNNVEAKRCCRELCSLDQLSDHNVMIQSEGRLIEVPHGVESQTSMSHWNIHQTLPTSLIDNSFGPTSWETGEPAQNKHDHSLSSKFNDPKSDIIKPNRERVKKEKRVGVDWDSLRKQVEATGRRDRTTNTMDSLDWEAVRCADIDDIAYTIRERGMNNRLAERIKDFLDRLVKDHGSTDLEWLRDVPPDQAKEYLLSIRGLGLKSVECVRLLTLQQVAFPVDTNVGRIAVRLGWVPLQPLPESLQLHLLELYPVLESIQKYLWPRLCKLDQRTLYELHYQMITFGKVFCTKSKPNCNACPMRGECRHFASAFASARLSLPAPEEKSLINATERKADINQAVVVHQQPLALTQESEPIESIQQLISVKSGGSNKDPIIEEPQTPEPECPQISEIDIEDTLYEDPDEIPTIKLNIEAFTKHVQNYMQENMELQEGSMSKALVLLSPEAASIPMPKLKNISRLRTEHQVYELPDSHPLLEKLQLERREPDDPCFYLLAIWTPGETANSVEQLHAHCSSQESGGLCGEKECFSCNSVREPDSEVVRGTLLIPCRTAMRGSFPLNGTYFQVNEVFADHDSSLNPIDVPRSWLWKLSRRTVYFGTSIPTIFKGLSTEEIQGCFWKGYVCVRGFDQTTRAPRPLIARLHFPASKMTRIKGKTDNPDGK

>Cs_demethylase2
MNSQVNSSGDFYAGNLLLRNQNIYSGSRPSTNNSFAQHVLTYGLPMFQPNYNLNPVSMTQTNQIFTNSVHTTPPVSSNVESVAYNQVSTPSFLVRDESSCFRKNADDFIRMFQDEAPRQHCDELLQSIVESSCVGNSTPFKGVESSCVGNSTPFKGTKDFVKQKDLEIDLNRTPEQRPPKRRQHTPTVFSGERFTDLLNLPLDGNLSLYEETQENFVTVPLDEATQKRHDELLKDLTDTLSAAISEPTKEVEKGSDQAIDLNKTPEQKTPKRRKHRPKVIKEGKPKKSPKPVTPKISKETPSGKRKYVRKKNIKEATTPPANVVEIKDSNTATKTKSCRRVIHFEMEKTGDEEQEKKQNEKDVSEENMGNFCFMTRPNVPDFCSQSTSVCGTSQDVHDSTQLGPMVAENVRPTIPSNPTHMNHMTTSHILQSEREAAEVPLNKSGYNKAENWLNVLRILHQGRANQYQTGFSNGYAPVQQNICAEDMQQFANQAKRNTYYKEVMGINSGYCQTVPNHQSNINEARGSKRGRPLTTYPTQPCSITTLDSSMTCQEVRQIGEFQRQGSNINIGPLENPGKKFESGLYATLHKRYSTIQSNEGCSSHLNTIGCNPTNSVGFTAEMKQAMLNGHHIRSNQITAKEIIGDRHIHSVVHENHFQRQQVSHNLHPAVDRTSVASGLNKVASYRSLMTGDKCNMIQPFPHPKAPEQGYACRQSDNSILTVRQAYQPMISGSLATNEVHKQGYSFGFQKFPAKTTSLLENEILHKMKRLSLNDHEVSIRSEQNAIVPYKGNGAVVPYVESEYLRKRKARPRVDIDPETERIWNLLMGKEGSEGIESHEKDKEKWWEEERKVFRGRADSFIARMHLVQGDRRFSRWKGSVVDSVIGVFLTQNVSDHLSSSAFMSLAARFPVKSASNLRTQGEVETSIVANESAACVLYPAESIRWHVQELSVPRFEMPQTSINHQNQIANSGTEKIFTELGGQIVEEEVISSQDSFDSTITQGTAGARSCSGSNSEAEEPIVSYNSSSTHYSNFTDIKQMETTATIQKSFSDLNRSSVSDEVSEHKHWQLPDGKQGSLTSEWNEIDNLSGHSLINFLVNIENQPKQVPDAPSNNQLHITPDCGVLEVEGREAFSEESTSSGPSIVSGCSTEKNMTFHRLNIGALEQRLDKTSAEDNVQARSHETTRMEHSESVSEHSVHLQGNGIQFRSHCEYNLHGKYEPCERNNTSPVESVSVTNPPPELDTPAEKSAVSNVVHVHAHTEKLLPGKGNLINFSNNEAHSLSQAHNEGNISPSKAKRRKVNSEKKGGMDWDSLRKQVEANGQIKEKGKDAMDSIDYEAIRLADVREISNAIKERGMNNMLAERIKEFLNRLVTDHGSIDLEWLRDVPPDKAKDYLLSVRGLGLKSVECVRLLTLHHLAFPVDTNVGRIAVRLGWVPLQPLPESLQLHLLELYPVLESIQKYLWPRLCKLDQRTLYELHYQLITFGKVFCTKSKPNCNACPMRGECKHFASAFASARLALPAPDEKGIVASTNPMSTEKQPPIVTNPLPILPPEGSTYAENTSGPSKCEPIVEVPATPEPEPNEITESDIEDAFYEDPDEIPTIKLSMEEFKTTLQHYIPEGDMSKALVALNPEAAFIPTPKLKNVSRLRTEHQVYELPDSHPLLREMDRREPDDPSPYLLAIWTPGETANSIQPPEQSCGSQDPNRLCNEITCFTCNSRREANSQTVRGTLLVPCRTAMRGSFPLNGTYFQVNEMFADHESSMKPIDVPRKWLWNLPRRTVYFGTSVSTIFKGLVTEEIQQCFWRGFVCVRGFDQKTRAPRPLIARLHFPASKLAKVKNGQTE

>Cs_demethylase3

VETNQMDSGQPEGNKADVQGSSWIPATPMKPILPKPPLQPLIYARMDRNQPRPYWLGPERLFSNSDKEAETSSGVACYGGANSMTANGSNDWEAAQARQFQVACNDNGTVTIHSMDALGGIPFLQLMALADAASIVGADAALGGNASDLFDSGSSYQIELESSSMKDRLSGSCIPEAKEYETSDHGSQHAHDLNFPSRTESDAAGIRVTSQFAPLTPDMGKIKYTERGMELQQIPTENSQDERELNHNCNTSITVDGENLRQNQELLEPAMHSTINCTPDGKEGKNDGDLNKTPASRQRRRKHRPKVIVEGKTNRTKQNLKTPSSNPSVRKRVRKSGLAKPSATPSIEVTGETSEQEIVKHRRKSCRRAITFDSQAQTRDESLDLGPLEQGSLTQNIQSTTGLEEVRIEEVGSSTDPNWSMNQMLKKYESLSEKEAPPTELSAENDSSEQTQPSKSQKENDTEQNGKVISSSDKENTVETILNDENHSLPGNSHGLIFCKNPPLTSIEQATCCLRKRPRAIKQAHTGSINLTGAHYNTLSAYQSMSWMHFPHIYKKKELRRGRTLFPQVHLLRLQISQGQKVHALSMTPKEITCKTVAGHEGNNLQDKLQTCGGIVGLGQTGRTKKKPRTAKRLSSSARPERISHWEKQPIYPTNHPPPAGSAKNINTSGTCINGLFEIMHATVAKKKRTKKKPSNSALLNINKDLQDRRFEHNAIVVYGRDGTIVPFNPIKKRRPRPKVELDEETGRVWKLLMGNINSKGIDGTDEENIKWWEEERKVFQGRADSFIARMHLVQGDRRFSQWKGSVVDSVVGVFLTQNVSDHLSSSAFMSLAARFPPKSKCRQASCSQEPIIELDEPEEACMFNLEDSMKLNKQIIHQQISEEDLLMKDEMEKGEGRIIVENNESSGSNVEDGSSNKEPEKKSFSSSHNILETCSNSVGEISLTETSSMQACLSGEKETYDSFSSQDCLDSSIPQTNESVEPSSEGNSEDLPSWSTEAHIDSSSEELTQMTGLNTLNANFTIDTCVEQSENTITNKLVENKCDNRIDDTSQPVDPEISLKNSVYHLSGYQTQQNQTSKSLEVDCCQTSNGVQTSNDCQNKDEQFHTEQSTLTVESDNHAIVEMELIVDIVEAPSSSSELSINAKEPCLTLQSQSSVIEDPQNVESPAECTNTVHEIPPNATEIATKPNPKECNLLSNEFKELKPASSRSQSKQVAKEKDNINWDNLRKRTETNGKTRQRTEDTMDSLDWEAIRCADVNEIAHAIRERGMNNMLAERIKDFLNRLVKDHGSIDLEWLRDVEPDQAKEYLLSIRGLGLKSVECVRLLTLHHLAFPVDTNVGRIAVRLGWVPLQPLPESLQLHLLELYPVLESIQKYLWPRLCKLDQRTLYELHYQMITFGKVFCTKSKPNCNACPMRGECRHFASAFASARLGLPAPEDKRIVSTTECREPDNNQPRTIDQPMLSLPPSTISSVEIKPSESHQSDGKTTAGACVPIIEEPATPEQETATQDAIIDIEDAFYEDPDEIPTIKLNIEEFSQNLQNYVQKNMELQEGDMSKALIALTPEAASIPTPKLKNVSRLRTEHQVYELPDNHPLLEKLDRREPDDPSSYLLAIWTPGETANSIQLPEKRCSSQEHHQLCCEEECLSCNSVREANSFMVRGTLLIPCRTAMRGSFPLNGTYFQVNEVFADHESSLNPIDVPRDWIWNLPRRTVYFGTSIPTIFKGLSTQGIQHCFWRGFVCVRGFDQKTRAPRPLMARLHFPASKLNRGRGKTEDQ

>Cs_demethylase4

MDSSCKRFLVPYDADGCYNEALFKNVSPYVGTNNNNGLSHHLQKEGTASVHGKQIIPYAADGCYNEARFKNASPYVGTNNNNGLSHHLQKEGTASVHEKQIIPYARKGGKKNSKHEHNPNSLDGMQGAIVPHPKSLNSTKKKEFGRVYLEPRDITVWKVLIENDSNSEKEKIDEEWWENERKVFRGRINAFNAIMHLILGDRRFSPWKGSVVDSVVGVFLTQNVSDHLSSSAYMSLAATFPLLETENYHGEEVFCIQQSTQRNERLFLCESKWNNDSMMETNKATGDPEEAKELMSADDAISSQGCQGSSIKENHDLTLLSSTCLEDDCGTCLCKNLDDTDNLALHSDKSTFEKEPYSSNQDSTLSCGSNKKNRTSEYKEVGWRDQNPISGNLNPSDTVHTPRSLGKCYSSAECISKSKSGLENNAEDSNSCEEMAVDLQFTPNEKSQGFIGSIEKFQNQEIQLIGDVNAKCSLCSESNEGKMEAGSQFSSDIDNSSLLVDFDVERVQSDESVVPASENTNKAKEKEKKEVKGYLEDRNPNHLNDEKETDKGKAKKSKMKPEVDWNSLREKWDSLRRKHPPCEPRSRDHMDSVDWEAVRFAEPTKIADAIKERGQHNIIAGRIKQFLDRTARLHGCIDLEWLRHAPPKDVKEYLLEIDGLGLKSVECIRLLALQQVAFPVDINVGRIAVRLGWVPLEPLPEEVQMHLLETFPMMDSIQKYLWPRLSMLDQRTLYELHYQLITFGKVFCTKRKPNCNACPLRADCRHYASAYASARLALPGLQEKGIVSTMSSGKSFEGNTRASLLHIDANPFSAECSTDNCEPIVEAPPSPEPAHDESQLTDIEDLYEYDSDDVPIIRLSSGQFTTTSLNCVDGSIGALVPLHPRVASIPMRKLKHIERLRTEHQVYELPDIHPLLSKLERRDPNDPCPYLLCILSPGETVDSCEPPNTICMYREIGEICSEGSCSSCNIVRQQNSGAVHGTILIPCRTAMRGKFPLNGTYFQVNEVFADDETSKNPIQIPREWIWDLPRRIAYFGTSTTTIFRGFICVRGFNRRTRTPKRLTERLHRATNASIKARANKTDDQKQKTYASNSNSPT

>Fv_demethylase1

MDMNEQRKDALHDWASCIAPTTPYRPLLPKLSQQHRHHEFFMPPNKFPFALPPIEGNNGAGLGGAGNTLEMSREVPCIDLLALAHAASNAADKNVVMHGQPQYDFPCHLPYDLNSLPATTYGQFAPITPEKASSNADYRKNQQIEEQMNAGATTCEIFEQRNNKDVANPATDSSHVTPSTQLQENNIIKEGDNSIDLNQTPQLKQRRRKHRPKVIREGKPKPPPKPPATKETPARRKYVRKNALDKNATPPPPKELGECTDSTKRKSTKRSCRRVLNYDMEDPGDDISSCRSLNSGSDSQVHNSCTNGASESTVQLRNGINSTVDNTPMGLAHELLLSTNQWLKDHLSLPEQQSPATPYPARKSSMEAREYADCQNNTAEGKATVRDQIGYKNVLDDEIRSSLQRPNDSNCSSSMILTQDNELNGSKRKYSSAVEQTESRPRNFLGVHYNNMPAYENMMSYMHFPYIYKKKRTDKAYTSIISSTSCRVTMAENVWRQSELQDVETILPSYQTQSSKRRRSKAPTRVRDLASLIRTPEHMLLQSTCLTKPPADGNGQRAMNCNSTQTCMDALVTEVGATLAKKKRTKRSTVISTHRSLVLYKNQPFLSGSSGVPPEVACTQILSVDAITDQLKCLNINRESSKFAYHGYNVVYNTQKQENNALVLYRRDGTVVPFEGAFDPIKKRRPRPKVDLDEETDKVWKLLMDNINSEGVDGTDEQKAKWWEEERRVFQGRADSFIARMHLVQGDRRFSPWKGSVVDSVVGVFLTQNVSDHLSSSAFMSLAARFPLKSVNNQTASDEKVASLAVDEPEVCISEISNQPLCDCSSVTFHDTEHSEEKVVNSNENTEITSEGVISTSEPDCKITHSLVNRTASECYIEEDLRTGYDTVSSQNSVDSSTSHTVEKTGSCESNSETEDAPNSCQNGSLDHSTLFLQKVEVHSVRSSHLSSHENLNCELHEPICMQHDNERKYIESGGASQDPSNNCCVHNTSNPEVVQVECSELIEEVIHSSNIFKNNYEDSLGEQSVLTAESVSQDTTSIKLTVNDQDAQRCFSESCTCIQGKSNVVLSQFRVGGNPNKVYVPTEKHTNKIQQSCNISGETADIMHKGPESDLSFNEVSKKDAATSKTKNRRPGKDKKAQQDWDKLRERAEPNGRKREKTANTMDSVDWEAVRTANVNDIAQTIKERGMNNMLAERIKEFLNRLLREHGSVDLEWLRDVPPDQAKEYLLSFRGLGLKSVECVRLLTLHHLAFPEGYEADVRTRLKVRTSVRSPPSGAGDGVPPPRRLQQRPRPLPLPGESILFRFSSEITQNFKQSRSRRKLEKNGLAGEGKWSGTRLESSRVDTNVGRIAVRLGWVPLQPLPESLQLHLLELYELHYQMITFGKVFCTKSKPNCNACPMRGECRHFASAFASARLALPGPEEKSIVSATQNRNKYRNPGEINNRMPLPIPLPLPHPHPTEQLGGNQQLEASQQSRPKSALGYTEPIIEEPASPEPECTEIVEDIEDFYEDPNEIPTIKLNMEQFTQNLQNYMQQNMELQQGEMSKALVALTPDAASLPTPKLKNVSRLRTEHQVYELPDSHPLLDRLGMDKREPDDPCNYLLAIWTPGETANSIQPPENRCSSQEFGKLCDDKECFQCNSAREAYSQTVRGTLLIPCRTAMRGSFPLNGTYFQVNEVFADHDSSLEPLDVPRGWLWNLNRRTVYFGTSIPTIFKGLTTPEIQQCFWRGFVCVRGFDQKSRGPRPLMARLHFPASRLAKPKDKKEE

>Fv_demethylase2

MDMNEQRKDALHDWASCIAPTTLDSPNLPKVSQQHRHHEFYKFPFALQPIEGNNGTGPGGAGNTLEMSREVPCIHLLALVHAASSAAQKTAVMHGEHQYDFPCHLPCDLNSAPETTFGQFAPITPEKASSNVDHRKNQQIEEQMNAGATSCEIFEQRNNKDVANPATDFSHATPSTQLQENSINKEGDNSIDLNQTPQLKQRRRKHRPKVIREGKPKPTPKPPTTKETPVRRKYVRKNALDKNATPPPPKELGECTDLTKPKSTKRSCRRVLNYDMKDPGDDISSLQKDTATLYPARKSSMEGREYVDCQKDTAEGKATVRAQIGHKNAVETELDGDTSSSLQRPNDSNCSSSMILTQENEQLNGSKRKYSSAVEQTEPRPQNFLGVHYNNMPAYENMMSYMHFPYIYKKRRTDKGCASIISSTSCHVTMAENVWRQSELQDVETILPSYRTQSSKRRRSKAPTRFRDLASLIRTPEHILLQSTCLTKPPADVNWQRAMNCNSTQTCMDALVPEVGDTLAKKKRTKRSTLTSSHRSLVLYKNQPLVSGSSGVPPEVACTQILSVDAIADQLKCLNINRESSKFAYQGYNVVYNTQDQENNALVLYRRDGTVVPIEGAFDPIKKRRPRPKVDLDEETDKVWKLLMDNINSEGADGTDEQKAKWWEEERRVFKGRADFFIARMHLVQGDRRFSPWKGSVVDSVVGVFLTQNVSDHLSSSAFMSLAAHFPLKSVNNQNASDEKVASLAVDEPEVCTSEISNQPLCDFSSVTFHDTEHSEEQVVNSSENTETTSEGVISTNEPDCKLTPSLVNGSATKNPRTASECYIEEDLRKRCDIVSSQNSVDSSTSQTVEKTGLCESNSETEDAPDTCQNGSLDHSTLFLQKAEVHSVRNSHLSPHDNLNCELHEPICMQHDDERIFIESGGASQDASNNCCIHNIPNPEVVQVECSELFEEVIHSSNISKNKYEDSPGEQSVLTAESVSQDTTSNKLTVNDQDAQRCFSESCTCIQEKSNMIQSQFRVGGNPNKVYVPAEKHTSKIQQSCNISEETTDIMHKEPESDLSFNEVSNVDAATSKTKNRRPGKDKKAQQDWDKLRERAEPNGRKREKTANTMDSVDWEAVRTANVNDIAQTIKERGMNNKLAERIKEFLNRLLREHGNVDLEWLRDVPPDQAKEYLLSFRGLGLKSVECVRLLTLHHLAFPVDTNVGRIAVRLGWVPLQPLPESLQLHLLELYPVLESIQKYLWPRLCKLDQRTLYELHYQMITFGKVFCTKSKPNCNACPMRGECRHFASAFASARLALPGPEEKSIVSATEDRNTYRNPGEINNKIPLPIPLPLPLPHPHPTEQLGGNQQLEASQQSRPKSAPGYTEPIIEEPVSPEPECTQIVEDIEDFYEDPDEIPTIKLNMEQFTQNLQNYMQQNMELQQGEMSKALVALTPDAASLPTPKLKNVSRLRTEHQVYELPDSHPLLDRLGLDKREPDDPCNYLLAIWTPGETANSIQPPENRCSSQEFGKLCDDKQCFQCNSAREAHSQTVRGTLLVPCRTAMRGSFPLNGTYFQVNEVFADHDSSLEPLDVPRGWLWNLNRRTVYFGTSIPTIFKGFVCVRGFDQKSRGPRPLMARLHFPVSRLAKPKGKKEELPSQNFQKKYA

>Fv_demethylase3

MKFGGDFSVPGDEDFRSMPPWKPVTPQKPLPLKPHNIMLNPQGNHLPAADYQHEFGNVQLGDNYSGMSQAFSPIVQFPRNVGFYSVGGGLAPQNGMTNHIAGSYPQFLGSDSSSWNDGLFPQLLPQDNTAAVYVASANVNYSRYVDMAANAPPTPNLYPQVNKRNNSSSFMLTNRNCNNGSYHSSPVMSRPVVVDFPSHFDVSQGHSNVTNWLLNNENHSSSANLLSNDDRSPHISQNVFPMPLRPSYDLNLNFIPGTEADTTSCAPSQLQFTPYRANILENNQHSSIQTWLTGESCSKENEKQVDFFTSTGDVAIENHGAELLQNIVESASAAFPTPYKENKDSDHESERGIDLNQTPPHKTPKRRKHRPKVVIEGKPKRTPKPAAEKNAEPKEPRTGKRKYTRKIIQKESSSQVADGTSQATYQKEPRTGKRKYTRKNIQKESSSQVADDTSEATYRNAGIGAKTCRRVLDFDLDVTTVEKQGKAARINQSKMKSSNLIDLENEVPVEKQTPETTSYPSPSLSHLRSNYIILPEGGESTVLLATRGDTQINNLHDARIHADSQYESAPMLQPSYAEGIAKHAMQAKTNGENHEKTKESANQDAFQSAIKSLSPANEGRKSKREYCHNIQQTRICSNYLPSSLLCEETFEFGCHQESSSSISKSSHKKQRVDTGYLSIHDMPLKVIPVEDDLGRSERKRANDANSNGFAAALNHTILHSYIGSNIVKERENTGVNKCTSDRFIEPIASGHNLPKQQMPIKSNSFKDITQVLSFSTNSTTDTCNQLVSSPPKKSSRRGKRQELQTQEHKSATKQTVRYTVLESALSSTEKRLQEQNFLHDHQQSFTKTSGLLLKTIYPSFMDDIIYRFSVLSINGSCNESMDQERNALVPYKGDGAIVPYEGAEYIKKRKPRPKVELDSETNRIWNLLMGKEGIAGTEGPDKQKQKYWEDERKVFQGRVDSFIARMHLVQGDRRFSRWKGSVVDSVIGVFLTQNVSDHLSSSAFMSLAARFTSKHQTQDKVGTDILVKEPELCIPIPDDATKSPENIIRQPIYNPVFMAPYASAEHLRDSVNSERNIMEAHSQCLEEEFVSSQDSFGSSVTQGTAENRSYSASNSEAEDPTARRQTNKVCCNSAYPPMDKDIIFQDFYHEVQGIPLLDDGSRQKHSKWEQLKTRSGKIDDFRSTYSVTNPINLDSRKMRPSIPPSTSNHIHMYQSSGELEPFGLESFSEESISSYWPSTAPKVNMEKDDSNKSFRNIELSGTVVSSSVEQNTLWRFQEPAIKDPYDALRLPSTNQPNYSQPRSYEYYQPSCNSHQYEGNQTFQTQVKKNSFIEPVKHSQRLPEKKYDNMQHVPNVNQLNTKSSNARDSTCTSIVNNGSVCPSAKEKYSHERSQNETSKNISRGRKKKSESDKKNTVDWDNLRKQVFADGRKEDRNKDVMDSLDYEALINADVKEISDAIRERGMNNMLAERIQEFLKRVVKDHGSINLEWLRDVPPDQAKDYLLSIRGLGLKSVECVRLLTLQHLAFPVDTNVGRIAVRLGWVPLQTLPESLQLHLLELYELHYQMITFGKVFCTKSKPNCNACPMRGECRHFASAFASARLALPGPGEKSIVSSTSSPVAEKNPAVAGNIMSLPPPENYPLQKVGAHRSEPIIEEPASPEQEFTNLSHSEIEDLLNEEEYDDDPGDVTHINISMEQLTDTLQDHMQNNMDLKDGDLSKALVALNPAAASLPTPKLKNVSRLRTEHQVYELPDSHPLLEGMDRREPDDPSPYLLAIWTPGETANSFQPPESSCGSKEGNTLCSEKTCFSCNSIREEKSQIVRGTILIPCRTAMRGSFPLNGTYFQVNEMFADHASSANPIDVPRGWIWNLPRRPVYFGTSVTSIFRGLTTEGIQYCFWRGYVCVRGFDRSTRAPKPLIARLHFPQSKMTKTRDEE*

>Fv_demethylase4

MTKAPRAKDVMDSSVHTPVRPQTMKFYQRKNTAKDDIVMDSSVHTPVRPQTMKFYQRKNRAKDNIVMDSSVHTPVRPQTMKVYQRKNRAKDDIVSSVHNPVKPQTMKVYQRKRKNKAKVGGDIPVKTRAIRIPESGSIEGSFMPVPSQPESMSQYNLKNGELVDGKFCIGDTSNISILKRKLHDATLQTPQKRLRRPNKKVYRPKVVPNLGMSVPRMELKTPTKETPKRMNNVSLKKSFITIDLQKIQKKKRLGRRRMEVERWQKIAIKFQANYLEQKYNKRRAAPGISQKELQKLFDNEQQLSPCFITQSGDLNIEESLSGIVSLLESDDLELLVKKIDTFEVNISHKSNHGIRVCYQENEKHGPHVELGIVAHANGERGMVAMTKKKGRWQEKLEKLEHGELFHPWNLQLQNIVKEGDIHDEEHSQFWEKERELFRGRIDLFLTRMRDIQGNRTFSEWDGSVVDSIVGVFLTQNVSDHLSSSAFISLAAAFPCQPTCDKQSKIVKIEEYIEGGTSKSSCEPSNDQTYGEDINIASSTSNLVLYDESECLVLYDESECWDDIISNNSQTHFNAIKQKGKKLKLKAKQKGKKLKADAEEINNIEGALAVRQADVDDIAEVIKGRGQQHNIADRIKQFLNEVNEDHKEINLEWLRWAPAEQARTYLTTIKGIGLKSVECVRLLALKQIAFPVDVNVARIAVRLGWVPLQPLPEDVQIHVLENMPVVDDIQKYLWPRLKKLRGFKELYELHYHLITFGKVYCTKRKPNCHSCPMKNECKYYLSAYARCEPPNPTSHTFIEDTTKLEYKLRECEPIIEEPASPGRSSPEPEYVERDIEDLFTDESVTRSPHMNAFGGSDISSLVPFMTREEAFSIPARRLKEIERLRTKHSVLSTQDDTDTIRGTILIPCRTATRGSFPLNGTYFQINEVFADHESSINPIKVHRDLLWNQTTRDVYCGTSASHILKGLSRDELSECFKDGFICVRAFNRRTRTPEPLHERLHISTVKTAKKAGIQGGRPQMSNTVDGHMPAREINCSGHGFSTWKKKCLDLLGFQQESDTLLLYYPKAEINYKPSREALDHSLPLILSCSSSSPESYSVNQPTQIGVFIKKS

>Md_demethylase1

MAELKVYQRREPDSRDNAMEEEEKEEVVQNRKEGLWVPQTPAKPATARTQKSYFRRRFKSNSGQNNSIVMSDSRVELERSDGVSVFSAAATSSEEKTCLIDPIEECPREEEKVQNLGGIGSTNLSCVGLFDLNKIVLECDEGCVSFSYGGSEKGCLEGLVAAEEVAANDDEILTVGEELVLQGIIVEPPISQSSTNATKGALFENINCCFIPVAQTGINEEKLQQGSAIPDCMSQCKSGDQQNQESVKQEDVIHRAENKNRTSNAANFMGGSKFPSTSSPSQKKKNLKRSPDGGIDLNEGPHKKPKKKVYRPKVVCIGRPRPIEPKTPKKATPKRPASKPKTPKPTAKMHVSSEQNMSAEKSSSSPKHSVPIEVTLDKVAFSVSDRATLDGDLKSKSNTENVIILANPPQKVRASHGTLMNLSCKKNLGLNIPATCRKQRLARRRREEDRWSAIARYFLKSERNLHKGLGDKNQGRVSFQKTETTDLIGALQPLITTRKKRSKGHTRGRNGGLVTEKWTNMGHSLNLELHNGECEGHPSKHEGPCSPNIRDSQEDNIGPFVQKCPDISHGVNHDKDAHVPSNDHGILVPYKEKHSKRRPELSPTDLERVKSWKLQLQAKVNEGQEKESEEEWWTNEREVLHGRINLLISRMNTIQGDRKFSPWKGSVVDSIVGVFLTQNVSDHLSSNAFMSLAARFPCQSHSNETDCEYINMVGTQESVGSNILAIMPKSEEELHTGEVALSMDYRHKESSSDKPNDLEGASTPPCDFHNPPEVSQIGFSEKVNHLRVLEDNVVSDNCGVQVISEKVEFHTSVEMSSLSPPCSQTKDAIVEPNDESHLCSQNMLVDKPESAVDTIIQNLVEPPNEANNFNQVNQKGQKPDLNEQQSGMKFDKTMKAPRRRKNEKAIVKTKLSWGWCKSIFSTSRERNRNHMDSVDWEAVRIAEVGQIAAAIKVRGQHNMIAGRVKKFLNQVHEDHKQIDLEWLRNAPPKLVTKYLREIDGIGLKSVECVRLLALQHVAFPVDVNVGRIAIRLGWAPLEPLPEQVQLHVLKQMPLLDTIQKYLWPRLKTLDPKTLYELHYQMITFGKVFCTKKKPNCNSCPMKGECRHYASAFASARLALPGPEKKPGSTKNGSSTDPLMMALQALNPPTVSSLEVSLDSKYQGKSCEPIIEEPSSPQPEYTEASLRDIEDLFLDDPNDMPTIKMRDERFSTTLEPYTGVFQENDMATGLVPSRYANFRAPKAKDAFRLCTKHLVYVLPDYHPLLQTLENRDPDDPSQYLLAIWPSDEKRCDPRESRELSNIVEPCFSLNNHSQAASQMVRGTLLIPCRTATRGSFPLNGTYFQVNEIFADHETSIRPIEVPRSSLWSLARLTVYCGTSTSSILKGLPLNEIKDCFVKGFICVRAFNRKTRTPEELSERFHVSTTKKGGKGGKGGKKNKKKG

>Md_demethylase2

MGEQGGDQSSCCWMPPTPYRPILTRPETKLRPEISTSSRPLGVEEIQENRLVIDLDPDPAEESGFVYHGAGVGAGSGVETSTDAYFRRVAQWRDVPCKELMILAVASANDGSGSAVLGDTQHPCVSGPTDPRGSVRLDPRASXPLIIRERLDAHEFSFPDNHPYDLNIPPPTTNGQFAPITPDKSMRVDSEQMYRIPSSNADDGQGQEIEEQWDANSATINIIDLENNKDIEKSAVDSSQATFSTQLQEHCNPDKEVNISIDLNKTPQPKQRRRKHRPKVIIEGKPKRNTQPSVSMENPKPKRKYVRKSTLNKSTTPPPQESTEHIDSNNLQPTKRSCRKALNFDAEEPRDGSSSSKSLHVGSQSQEMNVGTNGVQTNSTAPHRNEVELVADNTQAGIAQDLIRSTSRMLKHYLSLPDQQPPSTPQQTRGSTTYVDSQKEAAEEVGQMSTHSGYTAQTMLDXDTRSSQRSANDSTHSTTTNVMSWVHFPYIYKKKRTDKVQNSTIPSTSYXVNMAENIWRPSAAGCLTPGPQVNAGNVSTALEEVGNNPQDRPQSVHSFXPLYQTERSTKRRSSCPTRVRNLASLTRTPEHILHRTCLANQPPTDGNGQRVNQFDRSQTCIDALVTDVGATLAKRKRTKRNPLSSSQXGLLIYKNQPYFATASGVPPQVPFEQLLSAITEHFKCLDINRENSSRFAYHXYNVXSSYKAQDQEHNALVLYRRDGTVVPFDGSFDPIKKRKARPKVDLDQETDXVWKLLLENINSEGINGTDEEKEKWWEQERKVFRGXADNFISRMHLVQGDRRFSPWKGSVVDSVVGVFLTQNVSDHLSSSAFMSMAAHFPLKSRSNEISCDEEVASLVVDEPEVCISENSNQPGCDWSSLTFHDAEHSEEKVVNGHNNSGSTTEGVISTNEAQCKLSHPSEPGPGMYPNSLMNRSTTKITRTELYLQEDMRTYNGVSSQNSVDSSTSQTVEKTGSCESNSETENPPNRCENSSLDHSTSFVELLQRAESSMLHYSLGSTHMSSHDISNCGGYQPACVQHNDQRCEINREASLEPSSNCCLNLTPNPGVQQVEYFDLYGEVTQSSYASKNKCEDSPSERSALTSESPSQDTTHNKLTXNVQEAPRCSGNSCNNIQVGNNMAQSQLGLAGSSNNVDIHSQEQNSKIQQSCLNISGGTTDVMQKATELGSNEQSNSVNKEFSSTNAATSNTKNRKAGKEKKDQQDWDKLRKQAELNGKKREKTENTMDSLDWEAVRCADVNEIAQTIKERGMNNMLAERIKDFLNRLVREHGSVDLEWLRDVPPDQAKEYLLSFRGLGLKSVECVRLLTLHHLAFPVDTNVGRIAVRLGWVPXQPLPESLQLHLLELYPVLESIQKYLWPRLCKLDQRTLYELHYQMITFGKVFCTKSKPNCNACPLRTDCRHFASAFASARLALPGPEEQSIVSATEDRTTHPNPAGXNNRMPLPFPQATYQQLEASQKSEVKSTFGHCEPTTFQQLEASQISDAKSAVGXCEPIIEEPASPEPVCTQISEDIEDFCEGPDEIPTIKLNIEEFTQTLQNYMEKNMELQEGEMSKALVSLTSEAASLPTPKLKNVSRLRTEHQVCIWISENLTIRAITFWLFGLQAIPLNALMFIPGETPNSIQPPEKRCSSQELGKLCDDMECFSCNSAREANSQTVRGTLLIPCRTAMRGSFPLNGTYFQVNEVFADHDSSINPIDVPRAWLWKLYRRTVYFGTSIPTIFKGFVCVRGFDQKTRAPRPLMARLHFPASKLVKTKDKREE

>Md_demethylase3

MGEEGGDRSSCCWMPPTPYRPIVPRPEPKLQPEIATASRPLAFGVEETQENRVVIDLDPDPEEESGFVYHGAGAGAGAGVESSTDAYFCRVAQWRDVPCKELMILAVASANDGSGGAVLGDTQHEYSFPDNHPYDLNVPPPTTYGQFAPITPDKSMRVDSVQMSQIPSSNADDGRGQEIEEQWDANSATINIIDLENNKDIEKSAVDSSQATFSTQLQEHCNPDKEVNIIIDLNNTPQPKQRRRKHRPKVIIEGKPKRNPQPSGSMENPKPKRKYVRKSTLEKNKTPPPQESTEHIDSNNLKPPKRSCRRTLNYADTEEXIDGISSSKSFNVGSQSQEMNVGTXGVQSNSTGPCRNEVELVADNTQAGIAQDLVRSTSRMLKHYLSLPDQQPPXTPQQIRGSTTYVNSQKEAAEEKGQMXTCSGQANTAQTMLDCDTRSSQRSANDSTCSTTTVLATEEEQAKRSERNYLNAVEQAXPRTGNLFGANYNNXPAYYNVMSWVHFPYIYKKKRTDKAQNSTIPSTSYRVTMAENVWRPSTAGYLTSGPQVNADNFSTALGEAGKTPQERPQGVHRFLPLYQTERSTKRRSSCPTRVRDLASLTRTPEHILHRTCLVKQPPTDGNGQRVNHFDGSQTCIDALVTDVGATLAKKKRTKRNPLSSSQRGLVIYQNQQSFATASGCFQICXAAFVTVPDHQSQVFLQKENSSRFAYQGYNVISSYKAQYQEHNALVLYRRDGTVVPFDGSFDPIKKRKARPKVDLDQETDRVWKLLLENINSEGINGTDQEKEKWWEEERRVFHGRADNFIARMHLVQGDRRFSPWKGSVLDSVVGVFLTQNVSDHLSSSAFMSMAAHFPLKSRSNEKSCDEXVASFVVDEPEVCXSENSNQPGCDWSSLTFHDAEHSEEKVVNGNENSGSTTEGISTNEAECKLSHPSESGPGLYPNSLMNKSTTKITRTGSYLQEDMRTYDGVSSQNSVDSSTSQTVEKTGSCESNSETEYPPNRCENSSLDHSTSFVELLQRAESSMLHYSLKGTYMSSHDTSNCGGYQPECMQHNDQRCEINREEASLDPSSNCCLNLTPXPGVRQVECFDLFAEVTESSYTSKNKWEDSLSERSVLTAESPSQDTIXNKLTANVQETPRCSRNSSNNIQAGNNMAQSQLGWSNSVSKEFTSTNAATSKTKNRRAGKENKDQQDWDKLRKQAESNGKKREKTENTMDSLDWEAVRCADVNEIAQTIKERGMNNMLAERIKDFLNRLHREHGSVNLEWLRDVPPDQAKEYLLSFRGLGLKSVECVRLLTLHHLAFPVDTNVGRIAVRLGWVPLQPLPESLQLHLLELYELHYQMITFGKVFCTKSKPNCNACPLRAECRHFASAFASARLALPGPEEQSIVSATEDRTTHPNPAGINNRMPLPFPQATYQQLEASQKSEVKSTAGHCEPTTYHQLEVSEISEVKAAVGNCEPIVEEPASPEPVCTQISEDIEDFCDDPDEIPTIKLNIEEFTQTLQNYMEKNMELQEGDMSKALVSLTPEAASLPTPKLKNVSRLRTEHQVYELPDTHPLLEMLHMDKREPDDPCNYLLAIWTPGETPNSIQPPEKRCGSQELGKLCDDKECFSCNSSREANSQTVRGTLLIPCRTAMRGSFPLNGTYFQVNEVFADHDSSINPIDVPRAWLWKLNRRTVYFGTSIPTIFKGIDSEILCFDLFLNVSNKNTQYSSSSCQGYRHQKFSNASGEDFTKRLEHRAHYWPDCTSQPASWPEQKIRERSSHPEVSKQVYRAADSPH

>Md_demethylase4

MKFGRGFSVPQDDDLXFMDPWVPMTPEKLILAKGHPIPVDHRGNLLQGANXQQQLTGISREHVPVSVNYNGMNQDVSPNXQLLXNGGQYSXDGSLAKENLMINHXAGSFTQLLXGDISCWNNNPMTQLLHDKAAYVASANMNLGRSVDIAANTPLIPKLHPQSGNQGHNLGSYLLTKQNCSISYPSSNMMSRSLVMDFPFPVDNSRRDSNSFHWLFGNQNHCSSLSSNLLSNGDSSSQISQYGFLPSYDLNSLPSTEGDTASGVAGQLQFTTNEAKNLENDQXSAMLKSLTDESARKEKGKQVLSIEDEATQKNGDGRLQNIVESSSAAISTPYDKNKESGRGGDRGIDLNKTPQQKPPKRRKHRPKVIVEGKPKRTPKPATPKNTESKETRLEKRKYARKNVQKESPSQLAEVTRETAGPTAGKSAKSCRRVLHFDLEKTVDENLCREIGQQENKRTFDLNFDSQGTHAGTETNQVLTEKAAERSVLLNELMVDKHIPSTXRNPIPSMSLXPNNYTFRPESQASASLLATAKDMHLKNSHVMRRRTXTANSDLCQRKCRDEDTPVQQHTHAEGIXQDVIRAEIRCENLQKTKENVNQGDSLLKILSLPSEGRGSKREFFQTAEHPCRSTNNPPSSLSFQEILQLDRIQKNSGTPSKDCSGSRKKKKVENGHLSISXMPSKVTAVEKCLGKVDRTGENNLNSNGFASKNHTILSSYFENNKMIDRRNKGISKFMTDRYAYSITSGNGFLHRPISSKSNSCXGSTQVLSFSTHSXSQTCNQLASSPPKKSFQLGTKLVSHDNMSAKKQAGGTTVLKISSGTDKVRQEKDASYDYQQPSAIAIGFPIRTXSTIPIDDIIHQFNGLNLNGSCSXILEQENALVPYKGDGAIAPFKGIIKKRKSRPRVELDPETNRIWNLLMGKEGSEGVERTDNEKEKYWEDERKVFQGRVDSFIARMHLVQGDRRFSKWKGSVVDSVIGVFLTQNVSDHLSSSAFMSLAARFPLQSSNHQAPHKVGTNILVKEPEVRMTSPDDATKWHKDISSQPIYCQISRTLHESXENQRDXXNSLTERNLDEAHSQCFEEEFVSSQDSIESSVTQGAVGIRSYQASNLETEDPITGCQPNKIPISISTYQQMEKATMFQDFYRQVNGSSLLDDGSKNAQMEYSQIKTRLDKINHLTGSSFTNPINXDDENIQVPVXPSSNNQLHMYPNSGEPEPWRFGNFSEDSISSWPSTASRFNIEHDKYKNLRNEELLGSVVNSSMQQNGLRRSQEMPPVDPYALFRQHSIDLKNSSETRPSTGHNPSNYSHQRKGNLTFQLESTSVREPEKHAESLQRNKSASMQHVENVGDLSKKSFNVVDGRQIHMKNQSIDSNVQEQLYSYSQSQKETSKKSSKRRKGKADSEKKNDVNWDILRKQVQANGRKKERNKDATDSLDYEALKNXNVKEISEAIKERGMNIMLAERIQEFLNRLVREHGSIDLEWLRDVPPDKAKDYLLSIQGLGLKSVECVRLLTLHHLAFPVDTNVGRIAVRLGWVPLQPLPESLQLHLLEMYPMLESIQKYLWPRLCKLDQXTLYELHYQMITFGKVFCTKSKPNCNACPMRGECRHFASAFASARLALPGPEEKSIVSSSVPIAAEINPTLAVTPMSLPPPEINSFQIAGAEINNCEPIIEEPASPEQEFTELSQSDIEDLFYEDPDEIPTIKLNMEQFTSTLQNYMQEKMELQEGDMSKALVALTSEAAFIPTPKLKNVSRLRTEHQVYELPDSHPLLKGMDRREPDDPSPYLLSIWTPGETANSIQPPESRCGSEDQHKLCNEKTCFSCNSIREERSQTVRGTILVRKIDCGNXSNKKRTYMQFYTEISFNLXQIPCRTAMRGSFPLNGTYFQVNEASDIPRSILSNVQNLDKMFADHESSHNPIDVPRGWIWNLPRRTVYQQREFNTAFGEDKSLIVQSDKQAYILTD

>Md_demethylase5

MGPAADANNIFSSYVDDCSATPCRVTDVSTAQDKSTSKKRGYDAIYLRHKQPKSVRIQMEDKSKGEILIGGKRTKSPKPVKLKETRSSKCATAQKLPNVGRYNRVSVRKVQNDTQAQSMKQDQKAHGDILGKRNKSVHQVGPSDSTLRWHAQPQIDWSKDVSDNIKDKEEWWEEETKLFCKRANSFIACMNQVQGNRHFSAWRGSVVDSVVGAFXTQNAKDQSSSSAFMSLAAKYPTIQMEQACDYARNEKNVRSIVGWDFLEYIYYTRRTTKERSNDTMDSLDWEAVREAPLTEISKSILGRGMNNKLAERIKGFLDILKRDHGSIDLEWLRDXSPEGVKKYLLSIDGIGLKCVECVRLLALKQHAFPVDTNVXRIVMRLGWIPIQPLHWXLQHHHLKSARSEGDIEDLCIKVHDKNPTIKVNPKGCKIVKADHAQSKQKCFNRTSESLQEVVMSKSSAPVPTSXAPRSKRMSQFRTKHQLERREPDDPCPYLLVVWSSGKLXNPVGSTEGQTACFDENGQFLGXNGEDTAQTVLGTILIPCRTAMRGTFPLNGTYFQANELTFSVPGLLTKEIRDCFKKGAMYLEKYVKSKXCKLTCIDVKSTLNLAERELLLACLNDPCTGGHITVRLFTPKGVEELLQNTXLDLMKNYYGLLARNYERSILLIFKMQVVEPEDPYLLHNVDV

>Cm_demethylase1

MQNQELNAIVVYGRDGTIVPFNPIKKRRPRPKVELDEETGRVWKLLMGNINSTGIDGTDEENIKWWEEERKVFQGRADSFIARMHLVQGDRRFSQWKGSVVDSVVGVFLTQNVSDHLSSSAFMSLAARFPPKPKCHQASSSQEPIIELNEPEEVCMFNLEDSMKLNKQIIHQQISEEGSLMKDEMEKSEGRIIVDNNESSGSNVEDGSSNKEPEKKSFGSSHNILETFSNSVGEISLTETSSMQACFSGEKETYDSFSSQDFLDSSIPQTNESMEPSSEGNSEDLPSWSAEAHIDSSSEELIQMTGLNTLNANFTIDISVEPSENTITNNLVENKCDNRIDDTSQPDDPEISIKNSVYHLSGYQTQQNQTSKSLDVDCCQTSNGVQTSNDCQNKDEQFHTEQSTLTVESDNHANVEMELIVDIVEAPSSSSELSINAKDPGLTLQSQSSVIEDPQNVESPAECTNTVHGSPPNATEIATKPNPKEYNLLSNEFKELKPASSRSQSKQVAKEKDKINWDNLRKQTETNGKTRQRTENTMDSLDWEALRCADVNEIAHAIRERGMNNMLAERIKDFLNRLVKDHGSIDLEWLRDVEPDQAKEYLLSIRGLGLKSVECVRLLTLHHLAFPVDTNVGRIAVRLGWVPLQPLPESLQLHLLELYPVLESIQKYLWPRLCKLDQRTLYELHYQMITFGKVFCTKSKPNCNACPMRGECRHFASAFASARLGLPAPEDKRIVSTTECREPDNNQPRTIDQPMLSLPPSTISSEEIKPSESHECDGKTTAGACVPIIEEPATPEQETATQDPRIIDIEDAFYEDPDEIPTIKLNIEEFSQNLQNYVQKNMELQEGDMSKALIALTPEAASIPTPKLKNVSRLRTEHQVYELPDNHPLLEKLKLDRREPDDPSSYLLAIWTPGETANSIQLPEKRCSNQEHHQLCCEEECLSCNSVREANSFMVRGTLLIPCRTAMRGSFPLNGTYFQVNEVFADHESSLNPIDVPRDWIWNLPRRTVYFGTSIPTIFKGLSTQGIQHCFWRGFVCVRGFDQKTRAPRPLMARLHFPASKLNRGRGKTEDQ

>Cm_demethylase2

MDSIDYEAIRLADVHEISNAIKERGMNNMLAERIKEFLNRLVTDHGSIDLEWLRDVPPDKAKDYLLSVRGLGLKSVECVRLLTLHHLAFPVDTNVGRIAVRLGWVPLQPLPESLQLHLLELYPVLESIQKYLWPRLCKLDQRTLYELHYQLITFGKVFCTKSKPNCNACPMRGECKHFASAFASARLALPAPDEKGIVASTNPMATEKQPPVVTNPLPILPPEGSTYTENTLAPGNCEPIVEVPATPEPEPNEITESDIEDAFYEDPDEIPTIKLSMEEFKTTLQNYIPEGDMSKALVALNPEAAFIPTPKLKNVSRLRTEHQVYELPDSHPLLREMDRREPDDPSPYLLAIWTPGETANSIQPPEQSCGSQDPNRLCNEITCFTCNSRREANSQTVRGTLLVPCRTAMRGSFPLNGTYFQVNEMFADHESSMKPIDVPRKWLWNLPRRTVYFGTSVSTIFKGLVTEEIQQCFWRGFVCVRGFDQKTRAPRPLIARLHFPASKLAKVKNGQTE

>Cm_demethylase3

| MAETPLASKHVKEVEMEPSISTPLQQEAKRKRPQNNGIEATKRKKPRKKMYRPKVIGEGRKRKSKGSNTTPAKQQPVTPNPKTPSRVVPKLTTRKPRPLPKPRTRKLVPCQKNSILLEDGCRDLAEFAEINAIESCRDLVLVENEREIEKFIEEVAAIEAKETEPDNRVDAIDSCRNLVLVENELEIEKVFEEVDAIETKEADKENSNITVVRTPVDLSESFCLTKECKRKRSSRRISRKIIERKPYGLRAAREKGRGSRKKLLPFLFSKRKRTPMVRRCNLASLFELPVCNQLPRNIHKHAVNSGKTEILNGNNIVPIVGWQLKRPRNQRKSQARIALQILNCSSGDDDGVTKIGELACQSAFDLNANGRETHVGTAITDVNKEEILTKGSAQTSISHAIGRETSIETALTNVNKEETLTKGSAQISLAQVNGLTQGNRRETSIGTALIDVNKEETLTKGSAQTSILQANGRDTSTGTTITDVNKEERLTRGVSQTSLPQASSKFSDMRSEGGLRKMGEHNEQVTMKWLDISHFLTNSRLRMGRSGNNPPENGLSIPRITTGSGGNLTRHQDFTFSAKPSGNANKVRPTLSMVLWNNREGIRNNHEHNRLKGETRGVLGEEMDSSCKRFLVPYAADGRYNEALSRNVSPYVATNNNNGLSHHLQKERTASVHEKQIIPYARKGGKKNSKHEHNPNSLDGMQGAIVPHSKSLNSTKKKEIGRVNLEPRDIIVWKVLFENDSNSEKEKIDEEWWENERKVFRGRINAFNAIMHVILGDRRFSPWKGSVVDSVVGVFLTQNVSDHLSSSAYMSLAATFPLPETENYHGEEIFCIQQSTQRNEKLFLCESKWDNGRMETNKATGDPEEAKELMSVDDAISSQDCQGLSIKENHDSTLLSSICLEDDCGTCLSKNLDDTDNLALHSNKSTFEKEPYSSSQNSTSSCGSNQKNRTSESKEVGWRDQNPISGNFNSSDTMHTPRSLGKCYSSAECISKSKSGLENNAEDSNRCEEIAVDLQFAPNEKSQGFFASTEKFQNQEIQLIGDVNAQCPLCSESNEGKMEAGSQLSSDIDDSSQKVDFDVEKVQSQESVTQASNNTNEAKEKEKKEAKGYLEDGNPNHLNDEKETNNPKGKAKKSKMKPEVDWNSLREKWDSMRREHPPCEPRSHDHMDSVDWEAVRCAEPTKIADAIKERGQHNIIAGRIKEFLDRTARLHGCIDLEWLRHAPPKDVKEYLLEIDGLGLKSVECIRLLALQQVAFPVDINVGRIAVRLGWVPLEPLPEEVQMHLLETFPMMDSIQKYLWPRLSMLDQRTLYELHYQLITFGKVFCTKRKPNCNACPLRADCRHYASAYASARLALPGPQEKGIVSTMAPEKSFEGNTQAMNSASVLHIDANPFSEATNNCEPIIEAPPSPEPAHDESQLTDIEDLYEYDSDDVPIIRLSSGQFTTTSLNCVDDTITKALVPLHTRVASAPLRKLKHIERLRTEHQAYELPDTHPLLSQLERREPNDPCPYLLCILSPGETVDSCEPPNTRCVYRETGEICTEGSCSSCNIIREQNSGAVRGTILIPCRTAMRGKFPLNGTYFQVNEVFADDETSKNPIQIPREWIWNLPRRIAYFGTSTTTIFRGLAKEDIQYCFQKGFICVRGFDRRTRTPKRLAERLHRATNASIKARANKNDDQKQKTYASNSNSPQQL |
| --- |

>Os_demethylase1

MQDFGQWLPQSQTTADLYFSSIPIPSQFDTSIETQTRTSAVVSSEKESANSFVPHNGTGLVERISNDAGLTEVVGSSAGPTECIDLNKTPARKPKKKKHRPKVLKDDKPSKTPKSATPIPSTEKVEKPSGKRKYVRKKTSPGQPPAEQAASSHCRSELKSVKRSLDFGGEVLQESTQSGSQVPVAEICTGPKRQSIPSTIQRDSQSQLACHVVSSTSSIHTSASQMVNAHLFPPDNMPNGVLLDLNNSTSQLQNEHAKFVDSPARLFGSRIRQTSGKNSLLEIYAGMSDRNVPDLNSSISQTHSMSTDFAQYLLSSSQASVRETQMANQMLNGHRMPENPITPSHCIERAALKEHLNHVPHAKAAVMNGQMPHSYRLAQNPILPPNHIEGYQVMENLSELVTTNDYLTASPFSQTGAANRQHNIGDSMHIHALDPRRESNASSGSWISLGVNFNQQNNGWASAGAADAASSHAPYFSEPHKRMRTAYLNNYPNGVVGHFSTSSTDLSNNENENVASAINSNVFTLADAQRLIAREKSRASQRMISFRSSKNDMVNRSEMVHQHGRPAPHGSACRESIEVPDKQFGLMTEELTQLPSMPNNPQREKYIPQTGSCQLQSLEHDMVKGHNLAGELHKQVTSPQVVIQSNFCVTPPDVLGRRTSGEHLRTLIAPTHASTCKDTLKALSCQLESSRDIIRPPVNPIGPSSADVPRTDNHQVKVSEETVTAKLPEKRKVGRPRKELKPGEKPKPRGRPRKGKVVGGELASKDSHTNPLQNESTSCSYGPYAGEASVGRAVKANRVGENISGAMVSLLDSLDIVIQKIKVLDINKSEDPVTAEPHGALVPYNGEFGPIVPFEGKVKRKRSRAKVDLDPVTALMWKLLMGPDMSDCAEGMDKDKEKWLNEERKIFQGRVDSFIARMHLVQGDRRFSPWKGSVVDSVVGVFLTQNVSDHLSSSAFMALAAKFPVKPEASEKPANVMFHTISENGDCSGLFGNSVKLQGEILVQEASNTAASFITTEDKEGSNSVELLGSSFGDGVDGAAGVYSNIYENLPARLHATRRPVVQTGNAVEAEDGSLEGVVSSENSTISSQNSSDYLFHMSDHMFSSMLLNFTAEDIGSRNMPKATRTTYTELLRMQELKNKSNETIESSEYHGVPVSCSNNIQVLNGIQNIGSKHQPLHSSISYHQTGQVHLPDIVHASDLEQSVYTGLNRVLDSNVTQTSYYPSPHPGIACNNETQKADSLSNMLYGIDRSDKTTSLSEPTPRIDNCFQPLSSEKMSFAREQSSSENYLSRNEAEAAFVKQHGTSNVQGDNTVRTEQNGGENSQSGYSQQDDNVGFQTATTSNLYSSNLCQNQKANSEVLHGVSSNLIENSKDDKKTSPKVPVDGSKAKRPRVGAGKKKTYDWDMLRKEVLYSHGNKERSQNAKDSIDWETIRQAEVKEISDTIRERGMNNMLAERIKDFLNRLVRDHGSIDLEWLRYVDSDKAKDYLLSIRGLGLKSVECVRLLTLHHMAFPVDTNVGRICVRLGWVPLQPLPESLQLHLLEMYPMLENIQKYLWPRLCKLDQRTLYELHYQMITFGKVFCTKSKPNCNACPMRAECKHFASAFASARLALPGPEEKSLVTSGTPIAAETFHQTYISSRPVVSQLEWNSNTCHHGMNNRQPIIEEPASPEPEHETEEMKECAIEDSFVDDPEEIPTIKLNFEEFTQNLKSYMQANNIEIEDADMSKALVAITPEVASIPTPKLKNVSRLRTEHQVYELPDSHPLLEGFNQREPDDPCPYLLSIWTPGETAQSTDAPKSVCNSQENGELCASNTCFSCNSIREAQAQKVRGTLLIPCRTAMRGSFPLNGTYFQVNEVFADHDSSRNPIDVPRSWIWNLPRRTVYFGTSIPTIFKGLTTEEIQHCFWRGFVCVRGFDRTSRAPRPLYARLHFPASKITRNKKSAGSAPGRDDE

>Os_demethylase2

MGAEAEESLDHPGSLSGMSPATPDVAWKPAERRRRRSEADAEGSSCCSLSAATAAWVGAGNVESDDPSVRSVAAGEQSRSVSRPEEEEEECASCTQDSTVSPPVSECGDRTAQQEPSTQEYTVSPPPVSECGDKVVQQESSNQESTVSPPPVSECGDKIAQQGSSTRESTVLLPVSECCNKVAQQESSTQESTVSLPVIECGDKVAQQESSTQESTVSLPSSECGDKVAQQESSTQESTVSLPLSECGDKVAQQESSTQESTVSPPVSECGDKIARQEGAASAIPTPEKVEATPRRPRKRSTKGLTRFKIMKDHKAAQRTATPVEVKIKRKAKDNGRRPLGDKSVRRKLNFEGDAVDFEGNREFSRAKLMEDLRCLAKVHGLRDDLGAGKRSKKGKKRKKMTGEHQDNGESALVPYQKAPAATSSSALVPIQNSTQLAIVHHRNHLKNLRTKVLGLDEKTLQVYNVLRKWDETDSESFEGVDIGSGPEWNETRRHFEHYVDVFIATVHGLLGPRRFSEWGGSVTDSVVGTFLTQNVADNLSSNAFLNLVAKFPPTKRHINAEACSNLSLLIDDMRRKLNLNEQSNGTDSGSSDFTKPVDFEKENGYNEEVKGNYGRDYSTIIENFISIIEKHHKDMSTWDNARLENMVKDKSGTPVCSHRTLRKFMDTFEEKDTSHWDKLREEAYSKGYKIKGTGISDSADWEAVLHAPAVEIANSIAVRGQHYVIALRIQAFLKRVKKDHGNFDLDWLRYVPRESAKNYLISILGLGDKSVDCIRLLSLKHKGFPVDVNVARIVTRLGWVKLQPLPFSAEFHLVGLYPIMRDVQKYLWPRLCTISKEKLYELHCLMITFGKAICTKVSPNCRACPFSAKCKYYNSSLARLSLPPAEGHGHEYGEEQASTATPGRLLLSNDSHIAGFQQVCQPQIKINMPAGRESIYKCEPIIEIPPSPEHEYEESPYEQELYEDDLCDIEDTIPELQYDFEIDLCSLKHTVNNGSWTPNSGKDLALINSQHASVQNKRLKNIGRLRTEHNAYVLPDDHAILEEFEDRVPEDPCPYLLVVISCSDEHTVKGTILIPCRTATRGNFPLNGTYFQDHEVFADHSSSRSPITIPRECIWNLDRCIVYFGSSIQSIMKGQTRQDIEDCYKKGYICVRGFDRNTRYPKPICAKLHATNERNGTGENSRKKKKTSQEGKKIDDKSSFGKLEIN*

>Os_demethylase3

MRRAAQRAIAAAEKAARRKTQRAVAAAARAARRKKKAAESVARREQKAAMREQKAAAAAGQKKRKAAAAAAAAVRATARRKLDFDGEQQQIMPPERIYRQTSSSSRADLMDNMRLLLVAFDLSPPEQPCTPPARSERQILRLPPPATVTTAIVALKNKPKKATTVNKLALVPYKPTRAAASAVDEVLPGALVLYGDGEPTTQAARMFVPRWTSVRLVFDKLPPRFGLVVGLDAATRAVYNELVRREATSYGDDELHDVPGGPEWNERRREFERKVDHFMYNMRSIIGDRNFSPWGGSVVTSVVGTFLTQNVSDNLSSNAFMTIAARFPLKNRRNAGHHSDNVPLLAQNSGNVPLLLADGHDEQEQCHCQLQSIAQCSSGSKSGVAEPGDVSQRAEQTECPDKDLEAIMSAIRSGDISNFDDDHIQKVLKVRFKDSTPPPSESSSSRKKSISTAETIFKDIKSIKKNDTSHWHSLYDEARNRGYIRDDDIPDMVDWEALMNAPFADVVDCIKDRGQHSQMAFRILAFLIRMKRDHGNIDLEWLRFIPRAKANYKARMGATPSFAIISGVSSGRQQNSKFSTLIQAMPGYLSFDRICLTNQRTNRYELHCQMITFGKAICRKSKPNCGACPFTSECKYYKSQFGRAALALPEYSQQDATKDANMDDPAKTYDLIFKAHQYQIEYGKNTEMNYCEPVIEIPPTPLHENRGETSDEDDENGYYFDDDMEDIGRHDYDMEDIEHDYDMEVDLRSAKPTTNTSQAGATPGKEMIPINPRAKSTPMVKKFSLRTEYTAIFYSWALIILVFFTIAVFRCIIPDGHIILKKFDPRVPGDRNPYLLVFRSFDEHTVKATILVFADHSSSRSPIEINRDLVWELRRQTCIVHFGTRVHSVTKGQTREGLYHFYNEGYICTREFDRRTKFPKQLCVEIHATNVNKDIGKKRARPSTTRFYSEEDSGDEWSDW

> Os_demethylase4

MAKDENPSYLHLIFLSSRIRVFILDPPLSLPLSHGQCELQVVEEAAMDPSGLNLQGNPAENQESWTSGVSVGRGTPNLGVGTAVAGRSCPSSTLFPGSSLSSTALLNTMHEGSFPQTALVAGSVSSADEQHGAPPVRPSYNLPAGCTQVPISILVFHRRLTGRGSRCRSPQSRSFMPAPALSGVSEDGAYGPIPQSDFLSLRGPSEVFPGDMAMNHSEPATSYGYNSEYAPMHLQPNGLYTEASNTESEREASQLQQSAEAVICDSLSKLESAMEKIQGQNPQESSGLVAEGSADDNIHKYHQKAKRARTQITHSDKIDLPTQAVSACKEKTITQIEMQIADAERTEALKGEDAPAQKLKTRRRKHRPKVIREDRPAKKQMATTSEEKPLNQKPKRKYVRKNRNPSSLEKCAEPFSDHSISRESRTTVRSSIASVRRRLQFEFGEHGVQRDQSSMTNSWYQNQEKPVNAESSLCSVTKSSVQVEHGQELHMENSPEGLFFGINSKLNKILDEYIHLPEAAPKPSEQIPLAASGHVSEELARKQYDVRHTHDPDSTSYNIERSGLITTKGHKKDLDLNYSNTNGFQMYCSASLLPEMDSTKGSMTKVSKMDKNKKRHYGGESSLAGTQSSIIMRTAAEMLAVYQACGIKKKRSARVRRNSFLSVMDLEKNTSQESTRLPRSCMEALYESSYIKFMTKKRSQKARLNSPNSIQPNIDQKNRFSSETVFSGGFNGLKRSEETFQKTLPQIPDDKRINLDIHCKVPVESSPNTSTPPYMDYLQGVTSKFRYFDLNTEQVHKTEMHLSQTMPSLSSLGATNYLPNALVPYVGGAVVPYQTQFHLVKKQRPRAKVDLDFETTRVWNLLMGKAADPVDGTDVDKERWWKQEREVFQGRANSFIARMRLVQGDRRFSPWKGSVVDSVVGVFLTQNVADHLSSSAYMALAASFPTGSHGNCNDGIAGQDNEEIISTSAVGDRGTFEFFYNGSRPDIGLNFEFSMACEKIHMEPKDNTTVNELTKGENYSLHCKESAGSLCDHETEIDHKAKSISDFSAVELTACMKNLHATQFQKEISLSQSVVTSESILQPGLPLSSGMDHARRNFVGSISDTASQQVGSNFDDGKSLTGNDVTANETEYHGIKAAATNNYVVDEPGIPSGSSLYPFFSAIDCHQLDGRNDTHVSSTSPNCSICSASSNFKIGTIEENSSLFMPFDAHLAQRNGNMIVDTNLSSALESTELPVKLLHCGKRSCYEASEFQDHESLYATGGVIPETATKADDSTLKSGFASFNGLPDTAAQASKPKKSRTTSKKNSENFDWDKLRRQACGNYQMKERIFDRRDSVDWEAVRCADVQRISHAIRERGMNNVLAERIQKFLNRLVTDHGSIDLEWLRDVPPDSAKDYLLSIRGLGLKSVECVRLLTLHHLAFPVDTNVGRICVRLGWVPIQPLPESLQLHLLELYPVLETIQKYLWPRLCKLDQQTLYELHYQMITFGKVFCTKSKPNCNACPMRSECRHFASAFASARLALPSPQDKRLVNLSNQFAFHNGTMPTPNSTPLPQLEGSIHARDVHANNTNPIIEEPASPREEECRELLENDIEDFDEDTDEIPIIKLNMEAFSQNLENCIKESNKDFQSDDITKALVAISNEAASIPVPKLKNVHRLRTEHYVYELPDSHPLMQQLALDQREPDDPNELKDTREAPKPCCNPQTEGGLCSNEMCHNCVSERENQYRYVRGTVLVPCRTAMRGSFPLNGTYFQVNEVFADHSSSHNPINIPREQLWNLHRRMVYFGTSVPTIFKGLTTEEIQHCFWRGFVCVRGFNMETRAPRPLCPHFHLAASKLRRSSKKAATEQTH

>Os_demethylase5

MAKDENPSYLHLIFLSSRIRVFILDPPHSLSLSNGQCELQVVEEAAMDPSGLNLQGNPAENQESWTSGVSVGRGTPNLGVGTAVAGRSCPPLTLFPGSSLSSTALLNAMHEGSFPQAALVAGSGSSADEQHGGPPVRPSYNLPAGCTQVPISILVFHRRLTGRGSRRRSFMPAPALSGVSEDGASGPIPQSDFLSLGGPSEVFAEHAPMLLQPNGLYTEASNTESEREASQLQQSAEAVICDSHSKLESVMEKIQGQNPQESIGLVAEGSTDDNIHKYHQKAKRARTQITHSDKMDLPTQAVSACKEKTLTQIEMQIADAERTEAFKSEDAPAQKLKTRRRKHRPKVIREDRPAKKQMSTTSKEKPLNQKPKRKYVWKNRNPSSLEKCAEPFSDHSISRESRTTVRSSIASVRRRLQFEFGEHGVQRDQSSRTNSWYRNQEKPVNAESSLCSVTKSSVQVEHGQELHMENSPEGLFFGINSKLNKILDEYIHLPEAAPKPSEEIPLATSGHVSEELARKQDDVRHIHDHNERSGLITTKQNKKDLDLNYSNTNGFQMYCSASLLPEMDSTKGRMTKVSKMDKNQKRHYGGESSLAGTQSSIIMRTAAEMLAVYQACGIKKKRSARVRRNSFLSVMDLEKNTSQESTRLPRSCMEALYESSYIKFMTKKRSQKARLNSPNSIQPNIDQKNRFSSETIFSGGFNGLKRSEETFQKTLPQIPDDKRINLDIHCEVPVENSPNTSTPPYMDYLQGVTSKFRYFDLNTEQVHKTEMHLYQTMPSLSSLGATNYLPNALVPYVGGAVVPYQTQFHLVKKQRPRAKVDLDFETTRVWNLLMGKAADPVDGTDVDKERWWKQEREVFQGRANSFIARMRLVQGDRRFSPWKGSVVDSVVGVFLTQNVADHLSSSAYMALAASFPPGSDGNCNDGIAGQDNEEIISTSAVRDRGTFEFFYDGSRPDIGLNFEELSMACEKIHMEPKGNATVNELTKGENYSLHCKEPAGSLCDHETRIDHKAKSISDISLVELTARMKNLHATQFQTEISLSQSVVTSESILQPGLPLSSGMDHAPRNFVGGISDTASQQVGSNFDDGKSLTGNDVTANETEYHGIKAAATNNYVVDEPRIPSGSNMYPFFSATDCHQLDERNDIHVSSTSPNSSIGSASSNFKIGTIEENSSFFMPFDAHLAQMNGNMIAGTNVSSALASTELPVKLLHCCKRSCYEASEFQDHESLYATGGAIPETATKADDSTLKSGFASFNGLPDTAAQASKPKKPRTTSKKNSENFDWDKLRRQACGNYQMKERIFDRRDSVDWEAVRCADVQRISHAIRERGMNNVLAERIQKFLNRLVTDHGSIDLEWLRDVPPDSAKDYLLSIRGLGLKSVECVRLLTLHHLAFPVDTNVGRICVRLGWVPIQPLPESLQLHLLELYPVLETIQKYLWPRLCKLDQQTLYELHYQMITFGKVFCTKSKPNCNACPMRSECKHFASAFASARLALPSPQDKRLVNMSNQFDFQNGTMPTPHSTPLLQLEGSIHARDVHANNTNPIIEEPASPREEECRELLENDIEDFDEDTDEIPTIKLNMEAFAQNLENCIKESNKDFQSDDITKALVAISNEAASIPVPKLKNVHRLRTEHYVYELPDSHPLMQQLALDQREPDDPSPYLLAIWTPDELKDTREAPKPCCNPQTEGGLCSNEMCHNCVSERENQYRYVRGTVLVPCRTAMRGSFPLNGTYFQVNEVFADHSSSHNPINIPREQLWNLHRRMVYFGTSVPTIFKGLTTEEIQHCFWRGFVCVRGFDMETRAPRPLCPHFHLAASKLRRSSKTAATEQTH

>Zm_demethylase1

MQEELGQRMHVPHGINLCFSTSSELFQVESSIELGTAEVNPVPSEKLSANSQAVRDDAGAVEGIDMNGKPVQKPKRKKHRPKVIKEGQSAKLQKPKTPKPPKENGNQPTGKRKYVRKKGLSTPAKQIPSEGADTHTRAKPGIAQRCLDFDVEDQHGHLDLVSQTQETEIQTGPGDTQPSISGVERSNAQVSCHWGWGGTSSSIISADPIVDIQGLQADCIPKRVNFDLNNSMASQMPTNYSSRMDSSGQFFQFGLGEKVQTNQLLDYNCNLPARRVSHLSSSVDHMRHPLANFDQYISTSQVCTENSRRHYQMPSSSRISENRTAASQYTERVSMGGNFNPGACIGEGTVIKQMAQCYRLPESPLVPPKHNERDVMNGNLNEFSVYNDYLNFSSNSNYQTGAAFGFHDSPVYSDVLAMGKKREHNAISGHQISFDIDFVNSNSTRKFCIDDLLSTSSQTSYYPEACKRMRPENHSNQLNVTTGKLSSSSAFSGSLNTNNVSTINPGIGTLADIQRLMALEKSQASQQMIDFITSQNNMAGESTELAQQNNSDKGFVALHNKKFQSLTAQNIPLPDSTVNQSGESNILRNGIHQTQPWEITSRSHHSSDNFALPNKWSGYLTAGNTQLSSITVNPSTENYIQSNAIYQYQCLENVVAKVPVLSGTHNTSSQEGHNHSTAATNEHIRTTSEEVVRSFTQRASEPTTHGNYNLNSSRVTAEANSTEKPRKRGRPRKVVKPNGEPKERGTKGKQNVSHEKPTSQKGSHTDILKTNGISYASEPSTGITPRMAIVESKSSDQDKEIHGGVIPQATAISVDLLDGIIQKIKLLSISRPDNVVAEIPKDALVPYEGEFGALVAFEGKTKKNRSRAKVNIDPVTTMMWNLLMGPDMGDGAEGLDKDKEKWLDEERKVFRGRVDSFIARMHLVQGDRRFSRWKGSVVDSVVGVFLTQNVSDHLSSSAFMAVAAKFPAKPEVPEKPVAEMSHTPEQKDSCSCSGLFGDSIKLQGKMFIEEISDVRSLITTEDNEESNSNELIGSSAGYGVNHATGGCHVSYRKSLTESHENGLSGSVFPTTGFSSVVETEDGSLEDVISSQNSAVSSQNSPDYLFHRTDPIGSSSLQNFTEEGYIMRNISNGTGSSTDCSGFLPIQDPKGTLGLSEYYGHNPLLVSGVNKGVLLDLNRSYQPLHTSMPYVQNSESDFTGVSCFSHMDKSFHTGPNRVNLSSVTQSEASLYPTDPLQQDEFSPVIKQNFQPLYSSDKVSLFKEHCSYGNDFSRNKTEAAIMEPLVYSNPQELYTTSTEQMGVEQFQSGCGQQDNDVRVQTTSYERHQSSTLCGNQNSQLEILQGVASGSTQKFIDTQKSPSEVQQNGSKAKKVRGRPKTKTYDWDSLRKEVFSNGGDKQRNNDARDTVDWEAVRQAEVREISETIRERGMNNMLAERIKASLEFLNRLVTDHGGIDLEWLRDVPPDKAKDFLLSIRGLGLKSVECVRLLTLHHMAFPVDTNVGRICVRLGWVPLQPLPESLQLHLLEMYPMLEHIQKYLWPRLCKLDQRTLYELHYQMITFGKVFCTKSKPNCNSCPLRAECKHFASAFASARLALPAPEEKRLATSEDPNVVEFCHQTYINSGAVGELEWSANYPKHAVCGNLQPFIEEPLSPEPEPENVEAKDGAIEDFFNEDPDEIPTINLNIEEFTQNLKNYMQANHVEIEYADMSKALVAITPEAASIPTPKLKNVSRLRTEHQVYELPDSHPLLEGFEQREPDDPCPYLLSIWTPGKIIFFNPLFF

>Zm_demethylase2

MSAGRGDPNTPANRPLFLSAGVPNLGCHNVAAPAAAGNSGSIASQVLFSPCSTSVLNTIQEVPAPQSALLSPINRATEQGDVPCIQPSWNMPGGCTQVPINIIVFHRELTRRGSRPQLSPSATALPDVSEGASIRLAGSNFLSHGRTPSIVAGDMTTNSSQLATADKYNSEHRMNCLHDEASKTENGKGSTQLPQLATAIFRDSYNDLESVVPAATWETRVQHSQESTTCLAESTTDDNIHMYQSMQKRLKTQINKSEHTPLSAPTVPKEKTLTQIETQLASAEKTEMFRNEETPALKMKARKKKHRPKVIRENKPAKVQKPDSTPDGKSPNQKVKRSYVRKKRNLSSLENCSGPVSDQSISGATGVAARSRTSSVRRSLQFESKEQGLQGGHSSMANIHHHNYEKPGHAQSSFYSESEVQTGRVMQEGMENSPGELAFDMSRRLNKFLDEYIHLPDMLKPTQEVSAATSASFSTELAREEDNVGRTCKPDGKSKCSLFADERVVNPTIEGNKKDLELNYSDADGLVSSARSLPQMDPTRSQMGKVSEVENPTHHSNGSLPGTRDSVVLRAAAEMFAFFQAGAIKKKRSARLRRNSPFYTMDLKNNTLQASTRLPQPCMDALHRSSCIKFLTKKRSEKARPHCSSVIQPNDELKDRLSAGSTFYGATNGCKISEESSPNFSSQTLDNKRINVDTHCEVAEGRSTNTSTGPYMDYVQGVASKLKHLDLNTEQVQRTEMHLSLTTPAVISFEGTSGPSNALVPYGGVVMVPNERPLQLVKKQRPRAKVDLDFETTRVWNLLMGKSAESYGTDVEKERWWQQEREVFQSRANSFIARMRLVQGDRRFSPWKGSVVDSVVGVFLTQNVADHLSSSAYMALAASFPSRPVNNNCKDDGTTEDNKQTTRMSELVGEKSVFDLFYNGVRPDLEVGCEEVSMTCKKTHMEPTDNTRDSEFIEGETYSFDYKSTDESVCNHKGIGIEHKEQQLPDFSSAELTVSTELVQQIQIQKISSSQILTSETIQSRLSLSSEIPRNFVCGGSAAAYQQLGSNFDQGSSLTENDATASEIECHRLQMAAINDYGFGKPEIPSSSSMPFFLAVDPQQLKLRNETNVSSTSSNIPSDSASPNLKNGTDPLLMPFNSYMADWSSDKITYTTLNTPKISTELPVKLHHDKSSSFEAPNLKEHESVFATHEMTVEATRKEDEHTSKSSFTSYNGVPDTAAQASKPKKTRTTTAKNTENFDWEKLRRQACSEGQMKQRSFERRDSIDWEAVRCADVRRISHAIRERGMNNVLAERIQNFLNRLVRDHGSIDLEWLRYIPPDSAKDYLLSIRGLGLKSVECVRLLTLHHLAFPVDTNVGRICVRLGWVPIQPLPESLQLHLLELYPILETIQKYLWPRLCKLDQQTLYELHYQMITFGKVFCTKRQPNCNACPMRSECKHFASAFASARLALPAPQEESLVKLSNPFAFQNSSMHAMNSTHLPRLEGSIHSREFLPKNSEPIIEEPASPREERPPETMENDIEDFYEDGEIPTIKLNMEAFAQNLENCIKESNNELQSDDIAKALVAISTEAASIPVPKLKNVLRLRTEHYVYELPDAHPLLQQVKYCHGIKEITKTPKPCCDPQMGGDLCNNEMCHNCTAEKENQSRYVRGTILVPCRTAMRGSFPLNGTYFQVNEVFADHRSSHNPIHVEREMLWNLQRRMVFFGTSVPTIFKGLRTEEIQQCFWRGFVCVRGFDMETRAPRPLCPHLHIIARPKARKTAATEQVL

>Zm_demethylase3

MQEGLGQRMHVPHGINLCIATSSMPFQAQSFIEPSTGQVNPPVTSENLSANSQAVNDAGAVEGTDMNEKSVQKPKRKKHRPKVIKEGQSAKLQKPKTPKPPKENGNQPTGKRKYVRRKGLSAPAEQIPLCADTHTRAKPGVVQRCLDFDVEDQHGHLDLVPQTQETEIQTGPADTQPSISGVERSNVQISYHWGGTSSSISSVDPIAGIHGLQADCNSKRVSFDLNNSIVSQLPTNYSSPMDSSEQFFNFDLREEVRTNQLLPHLTSSVDHTRHPSVNFDQYISTSQDCPEKSPRHYQMVSSYRILENMTTASQYTERVSMGGNFNPEACMGEDAIIKQMAQSYRPLESPFVPPNHNERDMMNGNLNEFPVKNDYLKFSTNSNYQTGAGFDHCSPDYSDVLAMGTKREHDAISGHQISLGIDFINSNRTRKFCSDDPLSTSSQTSYCPQSCKRMRPEDHSNQLHGTMGKLSSASAFSDGLNTNKVSAMNPRIGTLADVQLLMALEKSKASQQMFEFDYVTSQNNMIHERTGLALQNIADKGFIALPNKQFPSFAAQNIPLPGSTVNQLGESNTLRNGVHQIQPWEITYRLHDSRDNFSLPGNTQFSNGTVNPSSENYIQSNAIHEHQCLENVVAKVPVLSETQNTSTQEGHNHCTAATTDEHIRTTSDEVVRSLTRRASQPTTNGNYNIKSSRVTAEANCTEKPRKRGRPRKVVNPNGEPKERGTKGKQNVGHAELISPKGSCADFLKTNGITYASESSTGITPRMSTVELKSSDQDKEIHGGAMPQATAASVDPLDGIIEKIKLLTINGPDKGVSEVPKNALVPYEGEFSALIAFEGKAKKSRPRAKVNIDPVTTMMWNLLMGPDMGDDAEGLDKDKEKWLDEERRVFRGRVDSFIARMHLVQGDRRFSRWKGSVVDSVVGVFLTQNVSDHLSSSAFMAVAAKFPVKIEVPKKPVAEMSHTPEQKDSCSGLFGDSIKLQGNIYIEEISDIKSLITTEDNEESNNNELIGNSAGNGVNCATGGCHVSYSKSLTGSHGNGLSGSVFPTTGFSGVVETEDGSLEDVISSQNSAVSSQNSPDYVVRRTDPIGSSSLQNFTEEGCIMRNMSNGTGISTDYTLFLPIQDPKGMLGLSEYCGLNPLPVSGVNKDMLLDLNRSYQPLHTYMPYDQNSESDFTGVSCLNHMGKSFHTGPDKVNLSSVTQSEASLYPIDPLEQGDFSPLIKQNFQPLHSSDEVPFFKEHSSCNDFLRNKTEAHFLEPLVCSNPQEVYTTSTDQMGAEQSQSGCGQKYNDARVQTASHERHQSSTLCDNQNSCSVVLQGVASDSTQKFVDTQKGPSKARQNGSKAKVRGRPKKKTDDWDSLRKKVLSNGGDKQRSHDARDTVDWEAVRQAEVREISETIRERGMNNMLAERIKEFLNRLVTDHGSIDLEWLRDVQPDKAKDFLLSIRGLGLKSVECVRLLTLHHMAFPVDTNVGRICVRLGWVPLQPLPESLQLHLLEMYPMLEHIQKYLWPRLCKLDQRTLYELHYQMITFGKVFCTKSKPNCNSCPMRAECKHFASAFASARLALPAPEEKCLVTLEDPNVVEFSHQTYINSGSVGQLEWSANYPKHAVSGNHQPIIEEPLSPECETENIEAHEGAIEDFFCEESDEIPTINLNIEEFTQNLKDYMQANNVEIEYADMSKALVAITPDAASIPTPKLKNVNRLRTEHQVYELPDSHPLLEGVKLHNRSMPPRHSVIQGRRVDYVEVQHALVATIYEKCRLRKSEEHFWQIPCRTAMRGSFPLNGTYFQVNEVFADHCSSQNPIDVPRSWIWDLPRRTVYFGTSVPTIFRGLTTEEIQRCFWRGFVCVRGFDRTVRAPRPLYARLHFPVSKVVRGKKPGAARAEE
