# Supplementary material for: Identification of DNA methyltransferases and demethylases in Solanum melongena L., and their transcription dynamics during fruit development and after salt and drought stresses
Source: PLoS One. 2019 Oct 9;14(10):e0223581. doi: 10.1371/journal.pone.0223581 (PMC6785084; doi:10.1371/journal.pone.0223581)
Supplement: S3 File — (DOCX) [file pone.0223581.s003.docx]

|  |  |  |
| --- | --- | --- |
| Gene name | **Left primer** | **Right primer** |
| SmelMET1 | CCTAGAGCATCTATGGCGCC | CACCTCCTTTGACACCTCG |
| SmelCMT2 | CTTTCCATGACCCAAAGCGC | AAGTGAGCAGGTGGAACGTC |
| SmelCMT3-like1 | AACTACCAAGCACGGATGGG | AAGGAAGAGCACCCCACATG |
| SmelCMT3 like 2 | AAGCCAGATCCGGTTGTAGC | AGCTGTTGTTCCTCTCGTGG |
| SmelDRM2 | TGTGGAGGCCTTTGATGGTG | TGTTTCTCCCAACCCACACC |
| SmelDRM3 | TGGGTTTCATCCGGTAGCTG | GTGGCCGTTTCCCTTTAAGC |
| SmelDemethylase_1 | ATGGCTGAGAGACGTTCCAC | CACTCTACGCTCTTCAGGCC |
| SmelDemethylase_2 | TCATCACCGACCACACCAAG | TGGCCGTGGTTTTCGTTTTC |
| SmelDemethylase_3 | TTCACCCTTGGCACCAGATG | TGCTGGGTGTCCTTGAAGTG |
| SmelDemethylase_4 | TGATGCCTGCCCAATGAGAG | CTGCTCAGAGGTGGCTTCTC |
| SmelDemethylase_5 | GTACTCGTGCGACTGGGTTG | GTGTGCATAATCGTGGCCAG |
| Actin | ACCACAGCTGAGCGAGAAAT | GACCATCGGGAAGCTCATAG |
