# Supplementary figures and images for: Identification of DNA methyltransferases and demethylases in Solanum melongena L., and their transcription dynamics during fruit development and after salt and drought stresses
Source: PLoS One. 2019 Oct 9;14(10):e0223581. doi: 10.1371/journal.pone.0223581 (PMC6785084; doi:10.1371/journal.pone.0223581)

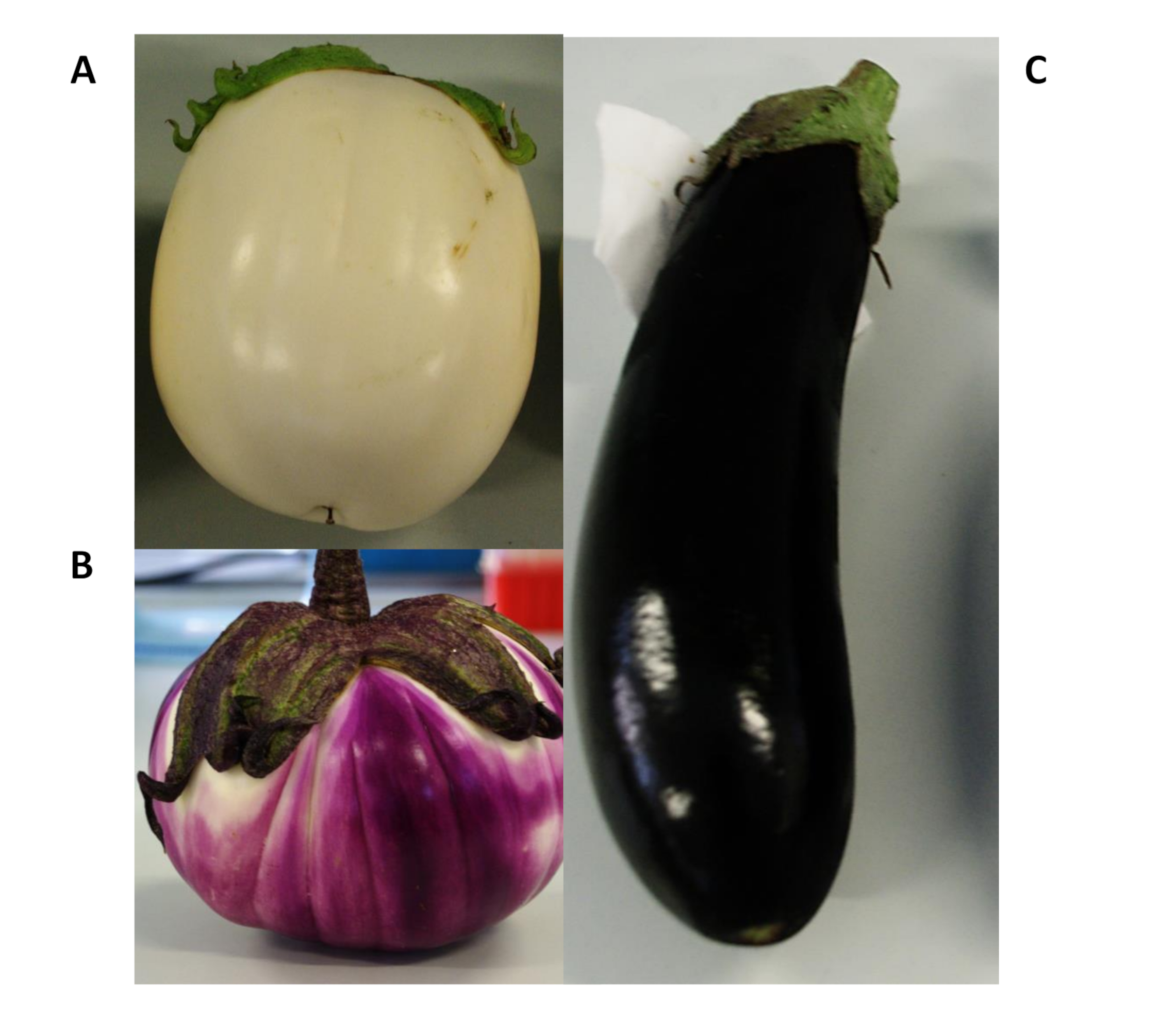

Supplement: S1 Fig — Fruits of Clara (a), Bella Roma (B) and Nite Lady (c) ~25 (stage 3, ripe fruits) days after anthesis. (TIFF) [file pone.0223581.s004.tiff]

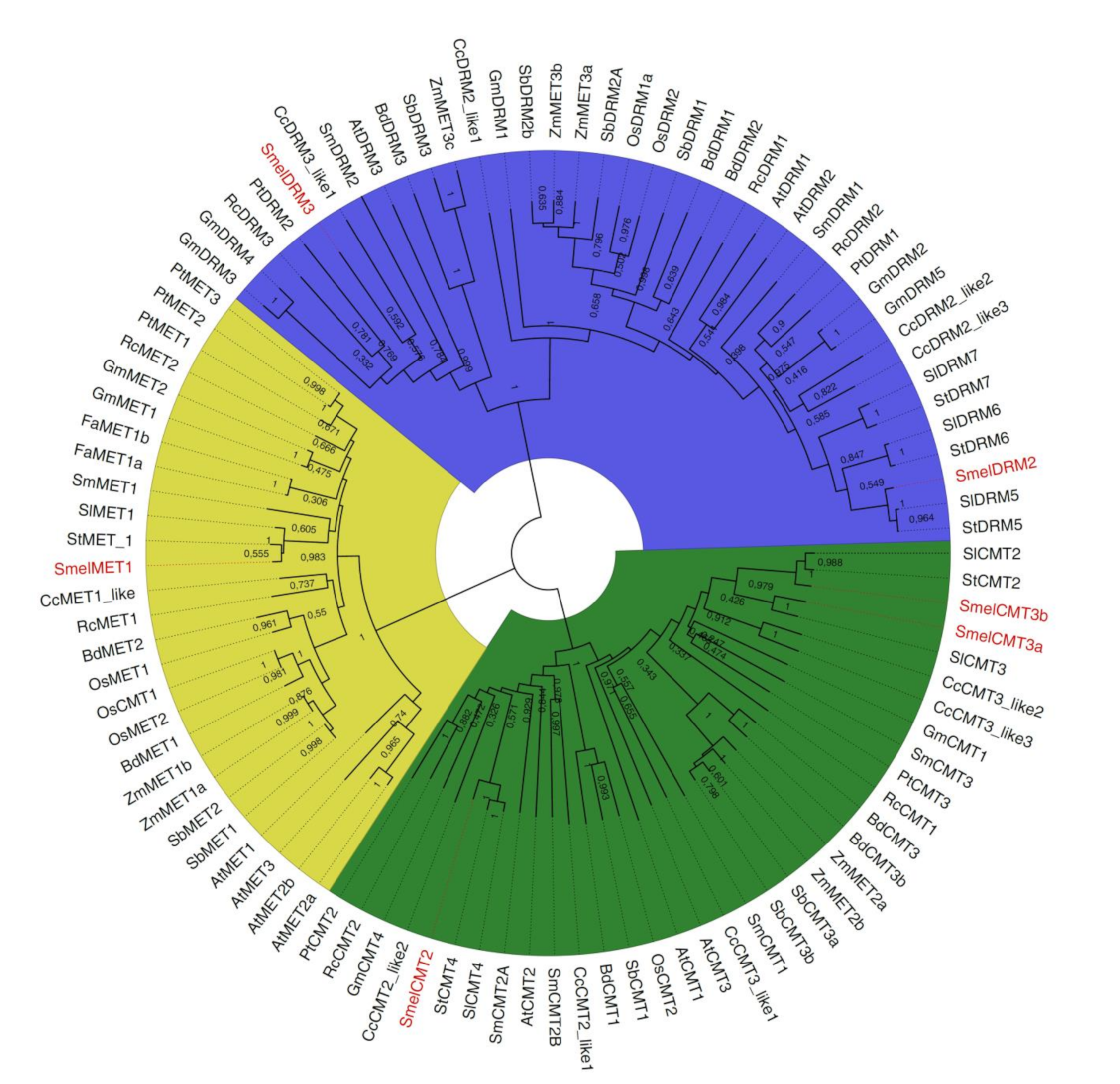

Supplement: S2 Fig — The unrooted, neighbour-joining tree was constructed by aligning the methylase domain of C5-MTase protein sequences contained in S1 File. The number at each node represents the bootstrap percentage value from 1,000 replicates. (TIFF) [file pone.0223581.s005.tiff]

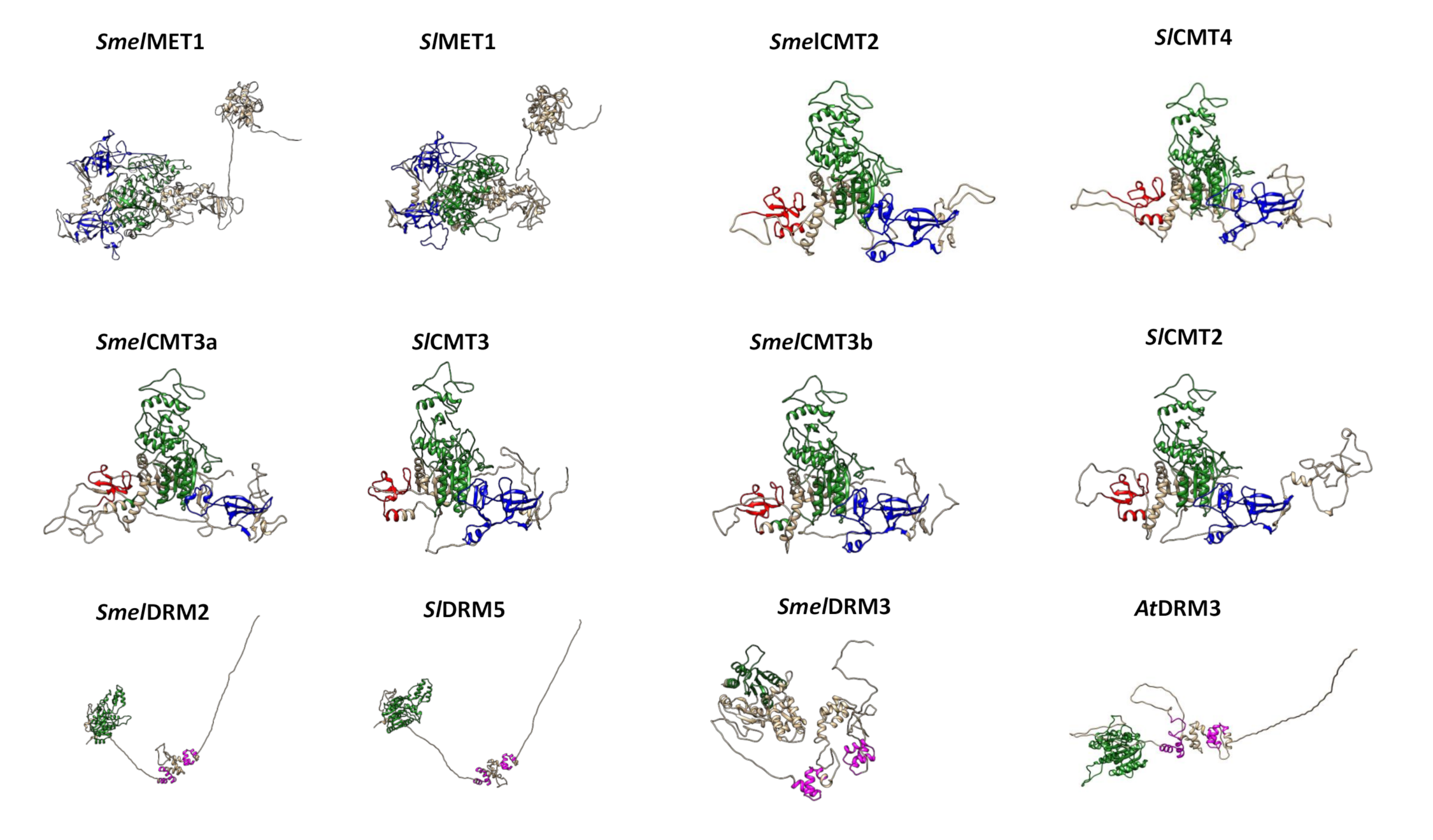

Supplement: S3 Fig — The three-dimensional structures of eggplant C5-MTases have been compared to those of S. lycopersicum, when possible, and of A. thaliana when a tomato ortholog was not available. The methylase domain is highlighted in green, the BAH domain in blue, the CHROMO domain in red and the UBA domain in magenta. (TIFF) [file pone.0223581.s006.tiff]

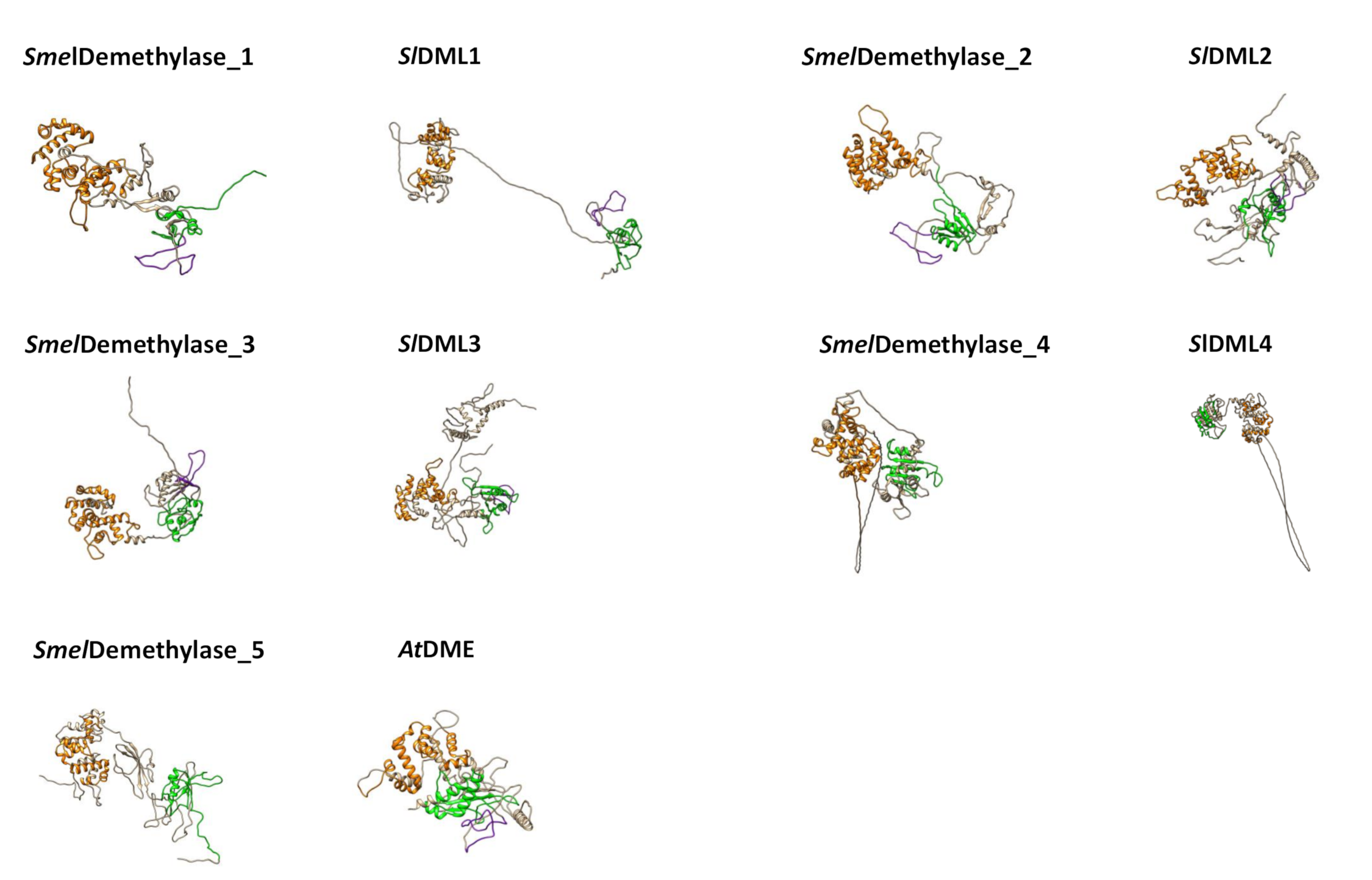

Supplement: S4 Fig — The three-dimensional structures of eggplant demethylases have been compared to those of S. lycopersicum, when possible, and of A. thaliana when a tomato ortholog was not available. The HhH-GPD domain is highlighted in orange, the Perm-CXXC domain in purple, and the RRM DME domain in green. (TIFF) [file pone.0223581.s007.tiff]
